# Azithromycin for community treatment of suspected COVID-19 in people at increased risk of an adverse clinical course in the UK (PRINCIPLE): a randomised, controlled, open-label, adaptive platform trial

**DOI:** 10.1016/S0140-6736(21)00461-X

**Published:** 2021-03-20

**Authors:** PRINCIPLE Trial Collaborative Group, Christopher C Butler, Jienchi Dorward, Ly-Mee Yu, Oghenekome Gbinigie, Gail Hayward, Benjamin R Saville, Oliver Van Hecke, Nick Berry, Michelle Detry, Christina Saunders, Mark Fitzgerald, Victoria Harris, Mahendra G Patel, Simon de Lusignan, Emma Ogburn, Philip H Evans, Nicholas PB Thomas, FD Richard Hobbs

## Abstract

**Background:**

Azithromycin, an antibiotic with potential antiviral and anti-inflammatory properties, has been used to treat COVID-19, but evidence from community randomised trials is lacking. We aimed to assess the effectiveness of azithromycin to treat suspected COVID-19 among people in the community who had an increased risk of complications.

**Methods:**

In this UK-based, primary care, open-label, multi-arm, adaptive platform randomised trial of interventions against COVID-19 in people at increased risk of an adverse clinical course (PRINCIPLE), we randomly assigned people aged 65 years and older, or 50 years and older with at least one comorbidity, who had been unwell for 14 days or less with suspected COVID-19, to usual care plus azithromycin 500 mg daily for three days, usual care plus other interventions, or usual care alone. The trial had two coprimary endpoints measured within 28 days from randomisation: time to first self-reported recovery, analysed using a Bayesian piecewise exponential, and hospital admission or death related to COVID-19, analysed using a Bayesian logistic regression model. Eligible participants with outcome data were included in the primary analysis, and those who received the allocated treatment were included in the safety analysis. The trial is registered with ISRCTN, ISRCTN86534580.

**Findings:**

The first participant was recruited to PRINCIPLE on April 2, 2020. The azithromycin group enrolled participants between May 22 and Nov 30, 2020, by which time 2265 participants had been randomly assigned, 540 to azithromycin plus usual care, 875 to usual care alone, and 850 to other interventions. 2120 (94%) of 2265 participants provided follow-up data and were included in the Bayesian primary analysis, 500 participants in the azithromycin plus usual care group, 823 in the usual care alone group, and 797 in other intervention groups. 402 (80%) of 500 participants in the azithromycin plus usual care group and 631 (77%) of 823 participants in the usual care alone group reported feeling recovered within 28 days. We found little evidence of a meaningful benefit in the azithromycin plus usual care group in time to first reported recovery versus usual care alone (hazard ratio 1·08, 95% Bayesian credibility interval [BCI] 0·95 to 1·23), equating to an estimated benefit in median time to first recovery of 0·94 days (95% BCI −0·56 to 2·43). The probability that there was a clinically meaningful benefit of at least 1·5 days in time to recovery was 0·23. 16 (3%) of 500 participants in the azithromycin plus usual care group and 28 (3%) of 823 participants in the usual care alone group were hospitalised (absolute benefit in percentage 0·3%, 95% BCI −1·7 to 2·2). There were no deaths in either study group. Safety outcomes were similar in both groups. Two (1%) of 455 participants in the azothromycin plus usual care group and four (1%) of 668 participants in the usual care alone group reported admission to hospital during the trial, not related to COVID-19.

**Interpretation:**

Our findings do not justify the routine use of azithromycin for reducing time to recovery or risk of hospitalisation for people with suspected COVID-19 in the community. These findings have important antibiotic stewardship implications during this pandemic, as inappropriate use of antibiotics leads to increased antimicrobial resistance, and there is evidence that azithromycin use increased during the pandemic in the UK.

**Funding:**

UK Research and Innovation and UK Department of Health and Social Care.

## Introduction

Identifying treatments that can be used to speed recovery and reduce hospitalisations due to COVID-19 in the community is critically important, particularly among older adults and people with comorbidities, who are at a high risk of adverse outcomes.^1^ Azithromycin, a licensed, widely available, cheap, and generally safe drug has been proposed as a treatment for COVID-19, with in-vitro studies suggesting activity against some viruses, including SARS-CoV-2.^2^, ^3^ Azithromycin might increase the pH of the Golgi network and recycling endosome,^4^ which could in turn interfere with intracellular SARS-CoV-2 activity and replication. The drug might also reduce levels of the enzyme furin;^4^ this could interfere with the ability of SARS-CoV-2 to enter cells, as the virus is believed to have a furin-like cleavage site in the spike protein.^5^ The ability of azithromycin to reduce the levels of proinflammatory cytokines, such as IL-6,^6^ could reduce the ability of SARS-CoV-2 infection to trigger a cytokine storm, along with associated tissue damage. Furthermore, some patients with viral respiratory illness might develop a secondary bacterial infection or present with a bacterial co-infection, which azithromycin could effectively treat. Azithromycin use in primary care has increased during the COVID-19 pandemic,^7^ which could contribute to antimicrobial resistance.^8^

**Research in context**

**Evidence before this study**

We searched PubMed on Jan 24, 2021, using the following search terms [(randomised OR trial) AND (azithromycin OR macrolide) AND (COVID* OR SARS-CoV-2 OR SARS-CoV)], with no language restrictions, and identified 243 results. Adding a filter for clinical trials limited the number of results to 16. Of these, we identified two randomised clinical trials with over 100 participants that provided some data on the effectiveness of azithromycin as a treatment for COVID-19 compared with control treatment or usual care. Additional searches of medRxiv and Google Scholar on Jan 24, 2021, using similar search terms with no language restrictions, identified a further randomised clinical trial of azithromycin. All identified trials were in hospitalised patients. A large randomised trial among patients hospitalised with COVID-19 in the UK found no difference in 28-day mortality, duration of hospital stay or treatment in hospital, or intensive care unit admissions among 2582 participants randomly assigned to receive azithromycin versus 5181 participants randomly assigned to receive usual care alone. A randomised trial in Brazil among 675 patients admitted to hospital with mild to moderate COVID-19 found no difference in clinical status by 15 days among patients randomly assigned to receive usual care plus azithromycin and hydroxychloroquine versus usual care plus hydroxychloroquine, versus usual care alone. A trial by the same group, among 447 patients admitted to hospital with severe COVID-19, found that patients randomly assigned to azithromycin plus hydroxychloroquine, versus hydroxychloroquine alone, had poorer clinical status at 15 days, although outcomes were more similar between the two groups by 29 days. We identified no randomised clinical trials of azithromycin as a treatment for COVID-19 in the community.

**Added value of this study**

All three randomised clinical trials that we identified were in hospital settings, and only one assessed azithromycin as a standalone therapy for COVID-19. To our knowledge, PRINCIPLE is the first randomised trial to assess the effectiveness and safety of azithromycin as a standalone treatment for patients with COVID-19 in the community. We found that azithromycin did not substantially improve time to recovery and found little evidence of an effect on admissions to hospital, when used in the community to treat COVID-19.

**Implications of all the available evidence**

Taken together, our findings plus the evidence to date suggest that azithromycin is not a sufficiently effective treatment to justify routine use for treatment of COVID-19, neither in the community nor in hospitals.

Randomised trials have found that azithromycin is not an effective treatment for patients who are admitted to hospital with COVID-19, either alone or in combination with hydroxychloroquine.^9^, ^10^, ^11^ However, there is a paucity of evidence regarding the effectiveness of azithromycin for treatment of suspected COVID-19 in the community, where earlier treatment might speed recovery and prevent hospital admissions. We aimed to assess the effectiveness of azithromycin to treat COVID-19 in the platform randomised trial of interventions against COVID-19 in older people (PRINCIPLE) study.

## Methods

### Study design and participants

PRINCIPLE is the UK National Urgent Public Health Priority open-label, multi-group, prospective, adaptive platform, randomised clinical trial in community care. A platform trial is an adaptive clinical trial in which multiple treatments for the same disease can be tested simultaneously. Participants are randomly assigned to either usual care or an intervention, where an intervention consists of the intervention active agent plus usual care. A master protocol defines prospective decision criteria to allow for stopping an intervention for futility, declaring an intervention superior, or adding a new intervention to be evaluated.^12^ Interventions currently or previously evaluated in PRINCIPLE include hydroxychloroquine, azithromycin, doxycycline, and inhaled budesonide. Here, we report outcomes for azithromycin, which was the second intervention included in the trial.

People in the community were eligible if they were aged 65 years and older, or 50 years and older with comorbidities, and with ongoing symptoms from PCR-confirmed or suspected COVID-19 (in accordance with the UK National Health Service [NHS] syndromic case definition of high temperature, a new, continuous cough, or a change in sense of smell or taste).^13^, ^14^ Symptoms must have started within the past 14 days. Comorbidities required for eligibility in those aged 50–65 years were as follows: known weakened immune system due to a serious illness or medication (eg, chemotherapy); known heart disease or a diagnosis of high blood pressure; known asthma or lung disease; known diabetes; known mild hepatic impairment; or known stroke or neurological problems. People were ineligible to be randomly assigned between azithromycin and usual care if they were already receiving acute antibiotics, or had a contraindication to azithromycin as identified in the summary of product characteristics and British National Formulary.

Initially, eligible individuals were recruited, screened, and enrolled through participating general practices. From May 17, 2020, people across the UK were able to enrol online or by telephone with study team support. After patients completed a baseline and screening questionnaire, a clinician or trained research nurse confirmed eligibility using the patient's primary care medical record, accessed remotely where necessary, before randomisation. Given the increased risk from COVID-19 among Black, Asian, and minority ethnic communities,^15^ we actively reached out to a range of religious and community organisations at national and regional levels to increase participation from diverse backgrounds.

The trial was approved by the UK Medicines and Healthcare products Regulatory Agency (MHRA) and the South Central—Berkshire Research Ethics Committee under the authority of the United Kingdom Ethics Committee Authority under the Medicines for Human Use (Clinical Trials) Regulations 2004 (ethics reference 20/SC/0158). Online informed consent was obtained from all participants, with the opportunity to discuss queries with the trial team by telephone. The trial is coordinated by the Oxford Primary Care Clinical Trials Unit, Nuffield Department of Primary Care Health Sciences at the University of Oxford (Oxford, UK), and conducted in accordance with the principles of the Declaration of Helsinki and the International Conference on Harmonization—Good Clinical Practice guidelines. The protocol, and details of how to participate, are available online.

### Randomisation and masking

Participants were randomly assigned using an in-house, secure, fully validated and compliant web-based randomisation system called Sortition (version 2.3). Initially, randomisation was fixed at 1:1 allocation between usual care plus azithromycin and usual care alone, with stratification by age (<65 years *vs* ≥65 years) and presence of comorbidities (yes *vs* no). After doxycycline was added to the trial on July 24, 2020, subsequent randomisation ratios were determined via response adaptive randomisation through regularly scheduled interim analyses. Details for implementing response adaptive randomisation were prespecified in the adaptive design report; the general idea is to allocate more participants to the intervention groups that have the best observed time to first recovery outcomes (appendix p 77). The trial team was masked to the randomisation ratios.

### Procedures

The intervention presented in this manuscript is oral azithromycin 500 mg once daily for three days plus usual care, compared with usual care alone. Usual care in the NHS for suspected COVID-19 in the community is largely supportive and focused on managing symptoms.^16^ Antibiotics are only recommended for use if bacterial pneumonia is suspected, in which case guidelines recommend doxycycline.^17^ Azithromycin was either prescribed or issued directly by the participant's general practitioner (GP), or issued centrally by the trial team and delivered to the participant. Azithromycin 500 mg is commonly used in primary care for bacterial respiratory infections and is similar to the dose used in early studies for COVID-19.^18^

The PRINCIPLE trial initially had two groups, with 1:1 allocation between usual care and hydroxychloroquine. The first participant was recruited on April 2, 2020, and the hydroxychloroquine group of the trial was closed by the UK MHRA on May 22, 2020, at which point azithromycin was introduced. Participants were followed up by responding to questions about their symptoms, antibiotic use, and use of health-care services in a daily, online diary for 28 days after random assignment, supplemented with telephone calls on days 2, 14, and 28. Participants were encouraged to nominate a friend, relative, or carer to be a study partner, who could help them provide follow-up data, and be contacted for information if the participant was unable to complete the daily diary themselves. We obtained consent to ascertain relevant outcome data from GP and hospital records about hospital assessments, admissions, intensive care treatment, and mechanical ventilation. We aimed to give all participants the opportunity to provide a self-swab test for SARS-CoV-2 infection, analysed by the Public Health England virology reference laboratory but, because of supply issues during the early stages of the pandemic, swabs were not available for all participants.

### Outcomes

The trial commenced with a single primary outcome: hospitalisation or death within 28 days. However, the proportions of people in the community requiring hospitalisation were much lower in the UK than seen in initial data from China, meaning a different outcome would be required to allow rapid evaluation of various interventions within reasonable sample sizes.^19^ The trial management and steering committees therefore recommended that the primary outcome be amended to include a measure of illness duration.^20^, ^21^ This change was approved by the research ethics committee and the MHRA on September 16, 2020, and implemented before any interim analyses were done. Thus, the trial has coprimary endpoints, as follows: time to first self-reported recovery within 28 days from random assignment, with time to recovery defined as the first instance that a participant reported feeling recovered (ascertained by answering the question, “Do you feel recovered today? ie, symptoms associated with illness are no longer a problem. Yes/No”); and hospital admission or death within 28 days of random assignment. Data for the time to recovery outcome were collected from the daily diary filled in by participants. Hospital admission and death data were collected from the daily diary, medical notes obtained either directly from GPs or via the Oxford Royal College of General Practice Research and Surveillance Centre,^22^ and also the Secondary Uses Service provided by the UK NHS Digital.^23^ These data were centrally processed by the trial data management team.

Secondary outcomes were a rating of how well participants felt (“How well are you feeling today? Please rate how you are feeling now using a scale of 1–10, where 1 is the worst you can imagine, and 10 is the best you can imagine”); time to sustained recovery (date participant first reported feeling recovered and subsequently remained well until 28 days after random assignment); time to initial alleviation of symptoms; time to sustained alleviation of symptoms (date participant first reported all symptoms as minor or none, with no subsequent increase in symptoms until 28 days after random assignment); time to initial reduction of severity of symptoms; contacts with health services; hospital assessment without admission; oxygen administration; intensive care unit admission; mechanical ventilation (components of the WHO ordinal scale); prescription of antibiotics other than the trial antibiotics; effects in those with a positive test for SARS-CoV-2 infection; and the WHO-5 Well-Being Index.^24^ The WHO-5 Well-Being Index includes five items relating to wellbeing measured on a five-point scale (appendix p 210). A total score is computed by summing the scores to the five individual questions to give a raw score ranging from 0 to 25, which is then multiplied by 4 to give the final score, with 0 representing the worst imaginable wellbeing and 100 representing the best imaginable wellbeing. We measured sustained recovery and sustained alleviation of symptoms because of the recurrent nature of COVID-19 illness.

Serious adverse events other than hospitalisation or death due to COVID-19 were assessed by the clinical team for relatedness to and whether they were expected with the trial treatment.

### Statistical analysis

For the primary outcome analyses, assuming a median time to first recovery of 9 days in the usual care alone group, around 400 participants per group (800 participants in total if only a single intervention *vs* usual care alone) would be required to provide 90% power for detecting a difference of 2 days in median time to first recovery (approximate hazard ratio [HR] 1·3). Additionally, around 1500 participants per group (3000 participants in total if only a single intervention *v*s usual care alone) would be required to provide 90% power for detecting a 50% reduction in the relative risk of hospitalisation or death, assuming a hospitalisation rate of 5% in the control group and 2·5% in the intervention group. These calculations are approximations that do not explicitly account for adaptations of the platform design. In the adaptive design report (appendix pp 68–80), we provide a more complete justification of sample size by simulating the operating characteristics of the adaptive design in multiple scenarios, which explicitly account for response adaptive randomisation, early stopping for futility or success, and multiple interventions.

The primary analysis population is defined as all eligible randomly assigned participants for whom data were available, with participants analysed according to the groups they were randomly allocated to, regardless of deviation from the protocol and irrespective of their COVID-19 status.

The first coprimary outcome, time to first recovery, was analysed using a Bayesian piecewise exponential model regressed on treatment and stratification covariates (age and comorbidity), and included parameters for time interval (0–7 days, 8–14 days, 15–21 days, and >21 days from random allocation). The second coprimary outcome, hospitalisation or death, was analysed using a Bayesian logistic regression model regressed on treatment and stratification covariates (age and comorbidity). We included these stratification covariates in the primary analysis as response adaptive randomisation increases the risk of imbalance on these variables. The coprimary outcomes were evaluated using a so-called gate-keeping strategy. For a given treatment, the hypothesis for the time to first recovery endpoint was evaluated first and, if the recovery null hypothesis was rejected, the hypothesis for the second coprimary endpoint of hospitalisation or death was evaluated. This gate-keeping strategy preserves the overall type I error of the primary endpoints without additional adjustments for multiple hypotheses. In the context of multiple interim analyses, the master protocol specified each null hypothesis to be rejected if the Bayesian posterior probability of superiority exceeded 0·99 for the time to recovery endpoint and 0·975 (via gate-keeping) for the hospitalisation or death endpoint. Based on trials of antibiotics for lower respiratory tract infection,^25^ a minimum of 1·5 days difference in median time to first self-reported recovery and 2% point difference in hospitalisation or mortality were considered to be clinically meaningful and were prespecified in the adaptive design report.

Per the prespecified protocol, the primary analysis model included all participants randomly allocated to azithromycin plus usual care, usual care alone, and other trial interventions, from the start of random assignment in the platform to the time of the last random allocation to azithromycin (Nov 30, 2020). Data were extracted from the database after all randomly allocated participants had the opportunity to complete 28 days follow-up. Because this population included participants randomly assigned to usual care alone before the azithromycin group was opened, the time to recovery model also includes parameters to adjust for temporal drift in the study population. This adjustment can capture temporal drift due to changes in SARS-CoV-2, usual care, or the pandemic situation, as well as changes in the inclusion or exclusion criteria over time, when including participants receiving non-concurrent usual care in the analyses.

We did sensitivity analyses of the primary outcomes on the concurrent randomisation analysis population, defined as all participants who were randomly assigned to azithromycin plus usual care, usual care alone, or other interventions only during the time interval when azithromycin was open to random allocation. Analyses of the coprimary outcomes for the concurrent randomisation analysis population were done using the same methods as described for the primary analysis population. Analysis of the secondary outcomes was done in the concurrent randomisation analysis population, but restricted to those in the azithromycin plus usual care and usual care alone groups only. Secondary time-to-event outcomes were analysed using Cox proportional hazard models, and binary outcomes were analysed using logistic regression, adjusting for comorbidity status, age, duration of illness, and eligibility for azithromycin at baseline. We did not adjust for baseline covariates for outcomes with low event rates. We did prespecified subgroup analyses of time to recovery for baseline severity score and duration of illness before random assignment, age, comorbidity, and SARS-CoV-2, using the concurrent randomisation analysis population to assess whether treatment effects varied across subgroups.

We also did secondary analyses restricted to participants who were positive for SARS-CoV-2, for the primary outcomes among the primary analysis population, and for the secondary outcomes in the concurrent randomisation analysis population. We compared serious adverse events in the as treated population (ie, the treatment that the participants received).

The master statistical analysis plan with full descriptions of definitions and analyses of outcomes was reviewed and signed off before the unblinding of results (appendix pp 168–207). An addendum to the plan was later added for further clarification of the analyses (pp 208–209). The adaptive design report details the analysis of the coprimary outcomes, including prespecified decisions at the interim analysis (appendix p 68). Because of concerns over trial integrity and confidentiality, we only present results corresponding to the primary analysis result and corresponding secondary outcomes and sensitivity for azithromycin, as randomisation and follow-up have been completed. Results from other trial groups will be presented in separate manuscripts when randomisation and follow-up is complete for each group. We did not explore the potential impact of missing data because of the high proportion of participants contributing to the analysis of primary outcomes (94%).

Analyses were done using R (version 3.6.0) and Stata (version 16.1). The trial is registered with ISRCTN, ISRCTN86534580.

### Role of the funding source

The funder of the study had no role in study design, data collection, data analysis, data interpretation, or writing of the report.

## Results

**Figure 1:**
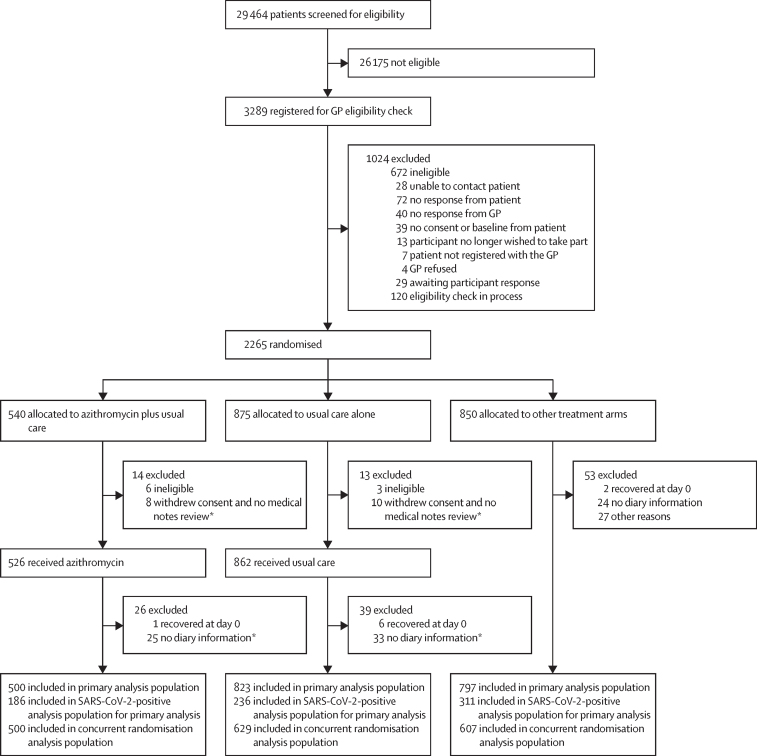
Trial profile GP=general practitioner. *Participants provided no diary information.

On Nov 30, 2020, after review of planned interim analyses by the Data Monitoring and Safety Committee, the PRINCIPLE Trial Steering Committee advised the Trial Management Group to stop random assignment of patients to the azithromycin group of the trial because the prespecified futility criterion was met. By this date, 2265 participants from 1460 GP practices were enrolled into PRINCIPLE across the UK (appendix p 210). 679 (30%) of 2265 participants were enrolled directly through 231 GP practices and 1586 (70%) participants via online or telephone contact with the study team. 540 participants were randomly allocated to azithromycin plus usual care, 875 to usual care alone, and 850 to other interventions. After exclusion of those who were ineligible, those who withdrew consent, and those without medical notes review and with no diary information available, 526 eligible participants were randomly assigned to receive azithromycin plus usual care, 862 to usual care alone, and 823 to other interventions (figure 1). 2120 (94%) of 2265 participants provided follow-up data and were included in the Bayesian primary analysis, 500 participants in the azithromycin plus usual care group, 823 in the usual care alone group, and 797 in other intervention groups. The concurrent randomisation analysis population included data from all participants randomly assigned to azithromycin plus usual care (n=500), and those concurrently assigned to usual care alone (n=629) for analysis of secondary outcomes, plus participants assigned to other interventions (n=607) for sensitivity analyses of coprimary outcomes (figure 1).

Characteristics of participants randomly assigned to azithromycin and concurrent controls were similar (table 1). The mean participant age was 60·7 years (SD 7·8), 1233 (88%) of 1388 participants had comorbidities, and the median duration of illness before randomisation was 6 days (IQR 4–10). 1148 (83%) of 1388 participants had a SARS-CoV-2 PCR result available, and 434 (31%) of 1388 participants had a positive result. 455 (87%) of 526 participants allocated to azithromycin plus usual care reported taking at least one dose of azithromycin and 374 (71%) took all three doses.

**Table 1:** Baseline characteristics of all eligible, randomly assigned participants by study group

		**Azithromycin plus usual care (n=526)**	Usual care alone*(n=862)	**Total (n=1388)**
Age (years)		60·9 (7·9)	60·5 (7·8)	60·7 (7·8)
Age by 5-year bands (years)				
	50–54	131 (25%)	236 (27%)	367 (26%)
	55–59	130 (25%)	219 (25%)	349 (25%)
	60–64	84 (16%)	123 (14%)	207 (15%)
	65–69	108 (21%)	153 (18%)	261 (19%)
	70–74	48 (9%)	90 (10%)	138 (10%)
	75–79	13 (2%)	30 (3%)	43 (3%)
	80–84	7 (1%)	8 (1%)	15 (1%)
	85–89	4 (1%)	2 (<1%)	6 (<1%)
	90–94	1 (<1%)	1 (<1%)	2 (<1%)
Sex				
	Female	301 (57%)	486 (56%)	787 (57%)
	Male	224 (43%)	375 (44%)	599 (43%)
	Missing data	1 (<1%)	1 (<1%)	2 (<1%)
Comorbidity				
	No	63 (12%)	102 (12%)	165 (12%)
	Yes	463 (88%)	760 (88%)	1223 (88%)
Ethnicity†				
	White	434 (83%)	700 (82%)	1134 (82%)
	Mixed background	10 (2%)	22 (3%)	32 (2%)
	South Asian	21 (4%)	34 (4%)	55 (4%)
	Black	2 (<1%)	5 (1%)	7 (1%)
	Other	12 (2%)	9 (1%)	21 (2%)
	Missing data	47 (9%)	92 (11%)	139 (10%)
Duration of illness before randomisation (days)		6 (4–9) [0–28]	6 (4–10) [0–63]‡	6 (4–10) [0–63]
Smoking status				
	Current smoker	62 (12%)	117 (14%)	179 (13%)
	Former smoker	187 (36%)	317 (37%)	504 (36%)
	Never smoker	264 (50%)	403 (47%)	667 (48%)
	Missing data	13 (2%)	25 (3%)	38 (3%)
Swab result within 28 days of randomisation				
	Negative	266 (51%)	439 (51%)	705 (51%)
	Positive	189 (36%)	245 (28%)	434 (31%)
	No result	2 (<1%)	7 (1%)	9 (1%)
	Missing data	69 (13%)	171 (20%)	240 (17%)
Asthma, chronic obstructive pulmonary disease, or lung disease		193 (37%)	329 (38%)	522 (38%)
Diabetes		90 (17%)	162 (19%)	252 (18%)
Heart problem§		86 (16%)	126 (15%)	212 (15%)
High blood pressure for which medication is being taken		209 (40%)	368 (43%)	577 (42%)
Liver disease		16 (3%)	23 (3%)	39 (3%)
Stroke or other neurological problem		31 (6%)	52 (6%)	83 (6%)
Taking angiotensin-converting enzyme inhibitor¶		103 (20%)	179 (21%)	282 (20%)
	Missing data	2 (<1%)	4 (<1%)	6 (<1%)
Fever at baseline				
	No problem	222 (42%)	372 (43%)	594 (43%)
	Minor problem	164 (31%)	300 (35%)	464 (33%)
	Moderate problem	122 (23%)	168 (19%)	290 (21%)
	Major problem	18 (3%)	22 (3%)	40 (3%)
Cough at baseline				
	No problem	94 (18%)	155 (18%)	249 (18%)
	Minor problem	217 (41%)	335 (39%)	552 (40%)
	Moderate problem	175 (33%)	317 (37%)	492 (35%)
	Major problem	40 (8%)	55 (6%)	95 (7%)
Shortness of breath at baseline				
	No problem	205 (39%)	271 (31%)	476 (34%)
	Minor problem	212 (40%)	380 (44%)	592 (43%)
	Moderate problem	92 (17%)	192 (22%)	284 (20%)
	Major problem	17 (3%)	19 (2%)	36 (3%)
Muscle ache at baseline				
	No problem	164 (31%)	266 (31%)	430 (31%)
	Minor problem	193 (37%)	335 (39%)	528 (38%)
	Moderate problem	134 (25%)	194 (23%)	328 (24%)
	Major problem	35 (7%)	67 (8%)	102 (7%)
Nausea at baseline				
	No problem	392 (75%)	646 (75%)	1038 (75%)
	Minor problem	96 (18%)	182 (21%)	278 (20%)
	Moderate problem	32 (6%)	27 (3%)	59 (4%)
	Major problem	6 (1%)	7 (1%)	13 (1%)
Feeling generally unwell (malaise) at baseline				
	No problem	31 (6%)	44 (5%)	75 (5%)
	Minor problem	211 (40%)	291 (34%)	502 (36%)
	Moderate problem	206 (39%)	263 (31%)	469 (34%)
	Major problem	53 (10%)	38 (4%)	91 (7%)
	Missing data	25 (5%)	226 (26%)	251 (18%)
Diarrhoea at baseline				
	No problem	356 (68%)	477 (55%)	833 (60%)
	Minor problem	94 (18%)	110 (13%)	204 (15%)
	Moderate problem	38 (7%)	39 (5%)	77 (6%)
	Major problem	13 (2%)	10 (1%)	23 (2%)
	Missing data	25 (5%)	226 (26%)	251 (18%)
Taken antibiotics since illness started		15 (3%)	37 (4%)	52 (4%)
	Missing data	1 (<1%)	1 (<1%)	2 (<1%)
Use of health-care services				
	General practitioner	150 (29%)	243 (28%)	393 (28%)
	Other primary care services	26 (5%)	53 (6%)	79 (6%)
	UK National Health Service 111	86 (16%)	160 (19%)	246 (18%)
	Accident and emergency department	6 (1%)	14 (2%)	20 (1%)
	Other health-care services	47 (9%)	78 (9%)	125 (9%)
WHO-5 Well-Being Index score		49·9 (25·4)	49·5 (24·2)	49·7 (24·7)
	Missing data	0	24 (3%)	24 (2%)

Data are mean (SD), n (%), or median (IQR) [range]. Severity of symptoms at baseline was self-reported.

* Includes participants randomly assigned to usual care alone before the azithromycin group opened.

† Data on ethnicity were collected retrospectively via medical notes review.

‡ One participant had a duration of illness of 63 days at baseline due to a long pause between screening and consent by the participant; this participant subsequently withdrew from the study and did not contribute any diaries or notes review and so was not included in the primary analysis.

§ eg, angina, heart attack, heart failure, atrial fibrillation, or valve problems.

¶ Such as ramipril, lisinopril, perindopril, captopril, or enalapril.

**Figure 2:**
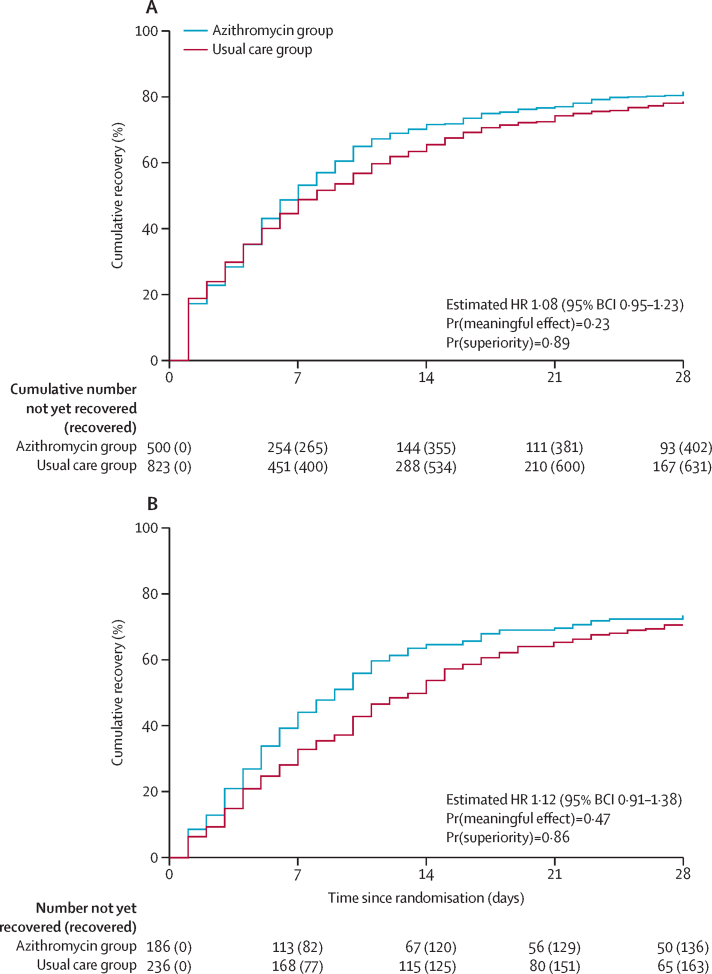
Summary and results of the time to first self-reported recovery (A) Primary analysis population. (B) SARS-CoV-2-positive analysis population. Pr(meaningful effect) is the Bayesian model-based estimated probability that the benefit in median time to recovery compared with usual care is at least 1·5 days. Pr(superiority) is the probability of superiority; treatment superiority is declared if Pr(superiority) ≥0·99 versus usual care. HR=hazard ratio. BCI=Bayesian credibility interval.

402 (80%) of 500 participants in the azithromycin plus usual care group and 631 (77%) of 823 participants in the usual care alone group reported feeling recovered within 28 days (table 2). Median time to first reported recovery for patients in the azithromycin plus usual care group was 7 days (IQR 3 to 17) and for patients in the usual care group was 8 days (2 to 23; figure 2; table 2). Based on the Bayesian primary analysis model, we found no evidence of a meaningful benefit in the azithromycin plus usual care group in time to first reported recovery versus usual care alone (HR 1·08, 95% Bayesian credibility interval [BCI] 0·95 to 1·23), equating to an estimated benefit in median time to first recovery of 0·94 days (95% BCI −0·56 to 2·43; figure 2). The probability that median time to recovery was shorter in the azithromycin plus usual care group compared with the usual care alone group (ie, probability of superiority) was 0·89 and did not meet the 0·99 threshold to declare superiority. The probability that there was a clinically meaningful benefit of at least 1·5 days in time to recovery was 0·23.

**Table 2:** Primary outcomes

		**Azithromycin plus usual care**	**Usual care alone**	**Estimated treatment effect (95% Bayseian credible interval)**	**Probability of meaningful effect**	**Probability of superiority**
**Primary outcomes (primary analysis population)**						
First reported recovery		402/500 (80%)	631/823 (77%)	..	..	..
	Time to first reported recovery (days)	7 (3 to 17)	8 (2 to 23)	1·08 (0·95 to 1·23)*	0·23*	0·89*
	Hospitalisation or death at 28 days	16/500 (3%)	28/823 (3%)	0·3% (−1·7 to 2·2)†	0·042†	0·64†
**Primary outcomes (SARS-CoV-2-positive analysis population)**						
First reported recovery		136/186 (73%)	163/236 (69%)	..	..	..
	Time to first reported recovery (days)	9 (4 to not reached)	13 (5 to not reached)	1·12 (0·91–1·38)*	0·47*	0·86*
	Hospitalisation or death at 28 days	11/186 (6%)	17/236 (7%)	1·6% (−3·1 to 6·2)†	0·43†	0·76†

Data are n/N (%) or median (IQR). HR=hazard ratio.

* Estimated HR derived from a Bayesian piecewise exponential model adjusted for age and comorbidity at baseline, with 95% Bayesian credible interval. HR >1 favours azithromycin.

† Estimated absolute benefit in percentage of hospitalisation or death derived from a Bayesian logistic regression model adjusted for age and comorbidity at baseline, with 95% Bayesian credible interval. A positive value favours azithromycin.

**Figure 3:**
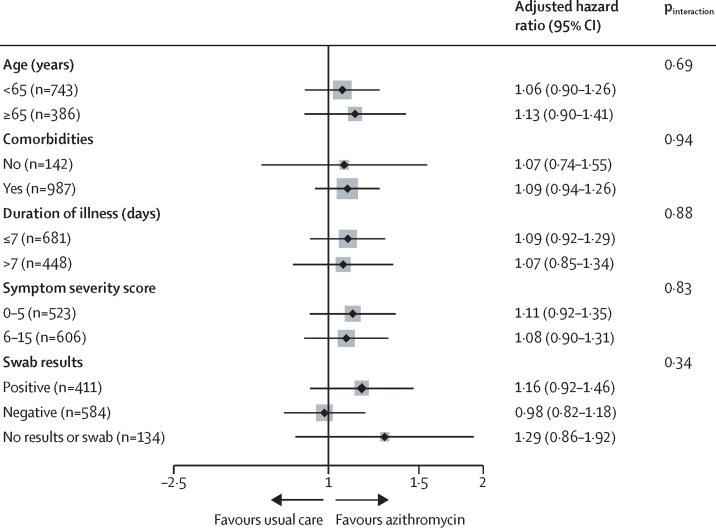
Subgroup analysis of time to recovery outcome (concurrent randomisation analysis population)

16 (3%) of 500 participants in the azithromycin plus usual care group and 28 (3%) of 823 participants in the usual care alone group were hospitalised (absolute benefit in percentage 0·3%, 95% BCI −1·7 to 2·2; table 2). There were no deaths in either study group. The probability that hospitalisations or deaths were lower in the azithromycin plus usual care group compared with the usual care alone group (probability of superiority) was 0·64, and was not formally analysed for significance due to the gate-keeping hypothesis structure. The probability that there was a reduction in hospitalisations or deaths of at least 2% (the predefined threshold of a clinically meaningful benefit) was 0·042. Results of both primary outcomes were consistent in participants with SARS-CoV-2 and the concurrent randomisation analysis population (table 2; figure 3).

Analysis of the secondary outcomes using the concurrent randomisation analysis population showed that there was no evidence of any difference between the two study groups in the daily score (1–10) of how well participants felt over 28 days (appendix p 217), nor the WHO wellbeing score at any of the follow-up timepoints, nor any of the hospitalisation secondary outcomes (table 3). Similarly, we found no evidence of treatment benefit in the azithromycin plus usual care group in time to first alleviation of symptoms, time to sustained alleviation of symptoms, and time to initial reduction of severity of symptoms (appendix p 218). More GP health-care service use was reported in the azithromycin plus usual care group compared with the usual care alone group (table 3). We found some evidence that sustained recovery from nausea and vomiting and diarrhoea was more rapid in the azithromycin plus usual care group compared with the usual care alone group (appendix p 218).

**Table 3:** Secondary outcomes

		**Azithromycin plus usual care (n=500)**	**Usual care alone (n=629)**	**Estimated treatment effect (95% CI)**	**p value**
Sustained recovery		317/500 (63%)	414/629 (66%)	..	..
Time to sustained recovery (days)		19 (7 to not reached)	20 (7 to not reached)	0·94 (0·81 to 1·09)*	0·39
Alleviation of all symptoms		401/420 (95%)	473/505 (94%)	..	..
Time to alleviation of all symptoms (days)		3 (1 to 7)	3 (1 to 7)	1·04 (0·91 to 1·19)*	0·57
Sustained alleviation of all symptoms		338/422 (80%)	425/510 (83%)	..	..
Time to sustained alleviation of all symptoms (days)		8 (3 to 27)	10 (3 to 24)	0·94 (0·81 to 1·09)*	0·40
Initial reduction of severity of symptoms		449/494 (91%)	554/622 (89%)	..	..
Time to initial reduction of severity of symptoms (days)		4 (2 to 10)	4 (1 to 11)	0·99 (0·88 to 1·13)*	0·91
Rating of how well participant feels (1 worst, 10 best)					
	Day 7	7·2 (1·8) [484]	7·1 (1·9) [620]	0·10 (−0·12 to 0·32)†	0·36
	Day 14	7·8 (1·8) [484]	7·7 (1·7) [613]	0·08 (−0·16 to 0·32)†	0·51
	Day 21	8·0 (1·7) [421]	8·0 (1·6) [539]	0·03 (−0·25 to 0·30)†	0·86
	Day 28	8·0 (1·7) [497]	8·3 (1·6) [612]	−0·15 (−0·46 to 0·16)†	0·33
Wellbeing (WHO-5 Well-Being Index score)					
	Day 14	45·3 (23·8) [472]	44·1 (24·1) [601]	0·61 (−1·89 to 3·11)†	0·63
	Day 28	52·9 (23·9) [474]	53·4 (24·3) [590]	−0·06 (−2·56 to 2·44)†	0·96
Self-reported contact with one or more health-care services		255/499 (51%)	323/628 (51%)	1·00 (0·89 to 1·12)‡	0·99
General practitioner reported contact with one or more health-care services		173/287 (60%)	200/387 (52%)	1·16 (1·01 to 1·32)‡	0·039
Prescription of antibiotics		20/271 (7%)	26/353 (7%)	1·00 (0·57 to 1·76)§	>0·99
Hospital assessment without admission		9/500 (2%)	11/629 (2%)	1·03 (0·43 to 2·46)§	>0·99
Oxygen administration		10/497 (2%)	15/625 (2%)	0·84 (0·38 to 1·85)§	0·69
Mechanical ventilation		2/496 (<1%)	5/625 (1%)	0·50 (0·10 to 2·59)§	0·47
Intensive care unit admission		3/495 (1%)	5/625 (1%)	0·76 (0·18 to 3·15)§	>0·99

Data are n/N (%), median (IQR), or mean (SD) [n]. All secondary outcome analyses were analysed in the concurrent randomisation analysis population but restricted to those in the azithromycin plus usual care and usual care alone groups. HR=hazard ratio.

* Estimated HR derived from a Cox proportional hazard model adjusted for age, comorbidities at baseline, duration of illness, and eligibility for azithromycin at baseline, with 95% CI.

† Mixed effect model adjusted for age, comorbidity, duration of illness, eligibility for azithromycin at baseline, and time. Participant was fitted as a random effect; WHO-5 Well-Being Index score was also adjusted for the score at baseline.

‡ Relative risk adjusted for age, comorbidities at baseline, duration of illness, and eligibility for azithromycin at baseline.

§ Unadjusted relative risk due to low event rate.

In subgroup analyses, we found no impact of the duration of illness before random assignment nor of the baseline illness severity score on the time to first feeling recovered (figure 3). Estimates of treatment benefit were similar for those younger than 65 years and aged 65 years and older, as well as between those with and without comorbidities (figure 3). In patients who tested SARS-CoV-2 positive and received azithromycin, we observed an estimated median benefit of 1·4 days (HR 1·12, 95% BCI 0·91–1·38; table 2) and a probability of benefit of 0·86, which was below the threshold for superiority of 0·99. Additional sensitivity analyses of interactions of swab results with time to first reported recovery in the concurrent randomisation analysis population supported these findings (appendix p 219). Results for the concurrent randomisation analysis population in SARS-CoV-2-positive participants for the secondary outcomes were similar to the concurrent randomisation analysis population results for all participants (appendix p 215).

One participant had side-effects from azithromycin and subsequently withdrew from the study. Two (1%) of 455 participants in the azithromycin plus usual care group and four (1%) of 668 participants in the usual care alone group reported admission to hospital, unrelated to COVID-19, during the trial (p>0·99).

## Discussion

In this trial of interventions for people with suspected COVID-19 within 14 days of symptom onset, and at increased risk of complications, azithromycin plus usual care did not substantially shorten the time to first self-reported recovery or decrease the risk of hospitalisation.

There are over 80 clinical trials of azithromycin for COVID-19 planned or underway,^3^ but few have reported results and none, to our knowledge, are in a community setting. Similar to our findings, several randomised trials among patients admitted to hospital have found that azithromycin was not effective as a treatment for COVID-19. Azithromycin has been evaluated as part of a hospital-based, platform, open-label randomised clinical trial of different COVID-19 treatments in the UK.^9^ 2582 patients were randomly assigned to azithromycin (500 mg once a day for 10 days, or until discharge if this occurred sooner) plus usual care and 5181 received usual care alone. Azithromycin did not improve the primary outcome of mortality at 28 days (22% in both groups; rate ratio 0·97, 95% CI 0·87–1·07; p=0·50). Similarly, the authors reported no difference in 28-day mortality between groups when the analyses were limited to patients with confirmed SARS-CoV-2 infection (rate ratio 0·95, 95% CI 0·86–1·06; p=0·38). There was no difference in the occurrence of new cardiac arrhythmias between groups. One serious adverse event attributed to azithromycin was reported (pseudomembranous colitis).

A Brazilian randomised clinical trial of hospitalised adults with known or suspected mild to moderate COVID-19 randomly assigned 667 patients to usual care alone (n=227), usual care plus hydroxychloroquine (n=221), or usual care plus hydroxychloroquine and azithromycin (500 mg once a day for 7 days; n=227).^10^ The primary outcome was clinical status at day 15, recorded on a 7-point ordinal scale from 1 (no longer in hospital and no limitation of activities) through to 7 (deceased). Among patients with confirmed COVID-19, the authors found that there was no difference in the odds of having poorer clinical status at day 15 between the groups (hydroxychloroquine plus azithromycin *vs* control odds ratio [OR] 0·99, 95% CI 0·57–1·73; p>0·99; hydroxychloroquine plus azithromycin *vs* hydroxychloroquine OR 0·82, 95% CI 0·47–1·43; p>0·99). More participants who received hydroxychloroquine plus azithromycin and hydroxychloroquine alone had adverse events compared with the usual care alone group (39·3%, 33·7%, and 22·6%, respectively). Prolongation of the corrected QT interval was most common in the hydroxychloroquine plus azithromycin group; however, the authors noted that participants in the usual care alone group were less likely to have electrocardiogram monitoring.

The same Brazilian study group conducted a trial of azithromycin 500 mg once daily for 10 days plus usual care versus usual care alone in 447 adult, hospitalised patients with severe COVID-19 (COALITION II).^11^ At the time of the trial, usual care for patients with severe COVID-19 was hydroxychloroquine 400 mg twice daily for 10 days. Thus, patients in the azithromycin group additionally received hydroxychloroquine. The authors reported no difference between the groups in the primary outcome of the odds of poorer clinical status at day 15, according to a 6-point ordinal scale of clinical status, among patients with confirmed COVID-19 (OR 1·36, 95% CI 0·94–1·97; p=0·11). The proportion of serious adverse events between the intervention and control groups were similar (42% *vs* 38%; p=0·35), and there was no difference in patients with a prolonged corrected QT interval between the intervention and control groups (20% *vs* 21%; p=0·66). These studies provide good evidence that azithromycin is not an effective treatment in hospitalised COVID-19 patients. Furthermore, there is no evidence of synergy between azithromycin and hydroxychloroquine in the context of COVID-19, as has previously been suggested.^18^, ^26^, ^27^

These data, in conjunction with our findings, suggest that despite potential antiviral and anti-inflammatory properties, azithromycin is not effective in treating COVID-19 without additional indications, even when used in the community and earlier in the course of the disease. A meta-analysis of 24 hospital-based studies found that bacterial co-infection (estimated from respiratory or blood isolates on presentation with COVID-19) was identified in 3·5% of patients (95% CI 0·4–6·7),^28^ suggesting that bacterial co-infection in the community is not common and, therefore, a beneficial effect through an antibacterial mechanism of action is unlikely in this setting.

Strengths of the PRINCIPLE trial include the evaluation of azithromycin as a standalone treatment in the community, with a focus on patients at a high risk of complications, and the use of 28-day patient-reported outcomes, which, in the case of hospitalisation and deaths, were confirmed by medical record review. Only around three-quarters of participants reported feeling recovered within 28 days, reflecting emerging evidence concerning the long-term nature of COVID-19 symptoms.^29^, ^30^ Furthermore, recovery is not always sustained and COVID-19 symptoms can recur or relapse,^30^ which we measured using the secondary outcomes of sustained recovery and symptom alleviation. We did not assess the potential effects of azithromycin on long-term outcomes beyond 28 days.

We included patients with no SARS-CoV-2 PCR results because this reflects many primary care settings, where timely testing might not be available. At the beginning of the trial, shortages of swabs and restricted community testing resulted in a high proportion of participants with no timely SARS-CoV-2 test result available. We do not know how many participants who did not have a test, or who tested negative with a self-swab, might have tested positive if the swab had been taken by a health-care professional or earlier in their illness. Increased test availability has meant that the prevalence of known SARS-CoV-2 infection in participants enrolling into PRINCIPLE is now much higher. However, in the present analysis, only 434 (31%) of 1388 participants with suspected COVID-19 had PCR-confirmed SARS-CoV-2 infection. In analyses of the primary outcomes restricted to this group, the estimated benefits of azithromycin remained below the predefined thresholds of clinically meaningful benefit. The low proportion of confirmed SARS-CoV-2 infections reflects the management of COVID-19 in UK primary care at the time of the study, when testing was not always available to many patients, and might reflect current practice in other community settings or low-income hospital settings, where access to timely SARS-CoV-2 diagnostic testing remains restricted and early empirical treatment might be used in symptomatic patients. This approach is supported by primary care data, which suggest that during the first wave of the pandemic in the UK, mortality was similar among people with PCR-confirmed SARS-CoV-2 infection and people with COVID-19 diagnosed clinically by GPs.^31^

Our trial was open label, as the study question concerned whether the addition of azithromycin to usual care was effective, rather than a comparison between azithromycin and placebo. In the context of a rapidly adapting platform trial, it would be logistically difficult to produce a placebo for the multiple drug treatments that are being added to PRINCIPLE as new evidence emerges. As participants were not masked to the intervention, self-reported outcomes might have been influenced by a placebo effect, although the effect of masking on estimates of effect has not been clearly shown.^32^ In our study, any potential placebo effect will have probably biased results towards a positive effect of azithromycin, meaning our finding of no effect on self-reported time to first recovery is robust to potential type II error. Our findings were similar for the hospitalisation or death outcome, which is less susceptible to ascertainment bias.

In conclusion, our findings show that azithromycin should not be used routinely to treat COVID-19 in the community in older adults, in the absence of additional indications. These findings have important antibiotic stewardship implications during this pandemic, as inappropriate use of antibiotics leads to increased antibiotic resistance, and there is evidence that azithromycin use increased during the pandemic in the UK.^7^ Using antibiotics to treat COVID-19 might also encourage patients to believe that antibiotics are an appropriate treatment for other viral respiratory infections, and our findings guide clinicians to avoid prescribing antibiotics to patients seeking treatment for COVID-19 in the absence of an additional indication. Finally, our findings highlight the importance of randomised controlled trials to assess medications during the COVID-19 pandemic and prevent the use of ineffective medications which, in the case of azithromycin, might contribute to other public health problems such as antimicrobial resistance.

## Data sharing

Data can be shared with qualifying researchers who submit a proposal with a valuable research question as assessed by a committee formed from the Trial Management Group, including senior statistical and clinical representation. A contract should be signed. Requests should be directed to principle@phc.ox.ac.uk.
