# Supplementary material for: Azithromycin for community treatment of suspected COVID-19 in people at increased risk of an adverse clinical course in the UK (PRINCIPLE): a randomised, controlled, open-label, adaptive platform trial
Source: Lancet. 2021 Mar 20;397(10279):1063–74. doi: 10.1016/S0140-6736(21)00461-X (PMC7972318; doi:10.1016/S0140-6736(21)00461-X)
Supplement: Supplementary appendix [file mmc1.pdf]

THE LANCET

Supplementary appendix

This appendix formed part of the original submission and has been peer reviewed. We post it as supplied by the authors.

Supplement to: PRINCIPLE Trial Collaborative Group. Azithromycin for community treatment of suspected COVID-19 in people at increased risk of an adverse clinical course in the UK (PRINCIPLE): a randomised, controlled, open-label, adaptive platform trial. *Lancet* 2021; published online March 4. http://dx.doi.org/10.1016/S0140-6736(21)00461-X.

Supplementary Appendix Content

Appendix 1: PRINICIPLE Master Protocol v6.3

Appendix 2: Adaptive Design Report v3.4

Appendix 3: Master Statistical Analysis Plan (and addendum) v1.1

Appendix 4: WHO-5 Wellbeing Index

Appendix 5: PRINCIPLE Trial Collaborative

Table S1: Baseline characteristics of all eligible, randomised participants by study arm (Concurrent Randomised Analysis population)

Table S2: Sensitivity analysis of primary and secondary outcomes

Figure S1: GP practices that have recruited at least one participant to PRINCIPLE (n=1406)

Figure S2: Summary and results of the time to first self-reported recovery for Concurrent Randomisation Analysis Population

Figure S3 Estimated mean and 95% confidence interval of daily rating of feeling well over the 28 days follow-up by treatment arm

(a) Concurrent Randomisation Analysis population but restricted to those in the azithromycin and usual care group only
(b) Concurrent Randomisation Analysis population for SARS-CoV-2 positive population

Figure S4: Effect of allocation to azithromycin on time to event outcomes Concurrent Randomisation Analysis population but restricted to those in the azithromycin and usual care group only

(a) Time to alleviations of all symptoms and alleviation of each symptom
(b) Time to sustained alleviation of all symptoms and alleviation of each symptom
(c) Time to initial reduction of severity of all symptoms and alleviation of each symptom

Figure S5: Effect of allocation to azithromycin on time to event outcomes (Concurrent Randomisation Analysis population for SARS-CoV-2 positive population)

(a) Time to alleviations of all symptoms and alleviation of each symptom
(b) Time to sustained alleviation of all symptoms and alleviation of each symptom
(c) Time to initial reduction of severity of all symptoms and alleviation of each symptom

Appendix 1

Trial Title: Platform Randomised trial of INterventions against COVID-19 In older peoPLE
Internal Reference Number / Short title: PRINCIPLE
Ethics Ref: 20/SC/0158
IRAS Project ID: 281958
EudraCT Number: 2020-001209-22
Date and Version No: 30th December 2020 version 6.3

**Chief Investigator and trial leader:**	Professor Chris Butler, Department of Primary Care Health Sciences University of Oxford
**Co-Principal Investigator and Co-trial lead:**	Prof Richard Hobbs, Department of Primary Care Health Sciences University of Oxford
**Co-Principal Investigators:**	Prof Simon de Lusignan, RCGP Research Surveillance Centre, University of Oxford<br><br>Prof Gail Hayward, Department of Primary Care Health Sciences University of Oxford
**Investigators:**	Dr Ly-Mee Yu, Primary Care Clinical Trials Unit, Department of Primary Care Health Sciences, University of Oxford<br><br>Dr Emma Ogburn, Primary Care Clinical Trials Unit, Department of Primary Care Health Sciences, University of Oxford<br><br>Dr Oliver Van Hecke, Department of Primary Care Health Sciences, University of Oxford<br><br>Ms Julie Allen, Primary Care Clinical Trials Unit, Department of Primary Care Health Sciences, University of Oxford<br><br>Dr Emily Bongard, Primary Care Clinical Trials Unit, Department of Primary Care Health Sciences, University of Oxford<br><br>Dr Hannah Swayze, Primary Care Clinical Trials Unit, Department of Primary Care Health Sciences, University of Oxford<br><br>Dr Sharon Tonner, Primary Care Clinical Trials Unit, Department of Primary Care Health Sciences, University of Oxford<br><br>Dr Nina Gobat, Department of Primary Care Health Sciences University of Oxford<br><br>Ben Saville, PhD, Berry Consultants, Texas, USA, & Department of Biostatistics, Vanderbilt University School of Medicine, Tennessee, USA<br><br>Prof Martin Llewellyn, Professor in Infectious Diseases, Medical Research Building, Room 1.08, BSMS, University of Sussex<br><br>Prof Stavros Petrou, Department of Primary Care Health Sciences University of Oxford

© Copyright: The University of Oxford and Oxford University Hospitals NHS Foundation Trust 2019

Dr Monique Andersson, Oxford University Hospital NHS Trust

Dr Susan Hopkins, Incident Director for COVID-19, Public Health England

Dr Sarah Tonkin Crine, Department of Primary Care Health Sciences, University of Oxford

Dr Aleksandra Borek, Department of Primary Care Health Sciences, University of Oxford

Dr James Ray, Oxford University Hospitals Emergency Medicine Consultant, NHS England Lead for Urgent and Emergency Care for Thames Valley and London

Prof. Mahendra G Patel, Honorary Visiting Professor of Pharmacy University of Bradford , Honorary Senior Lecturer Academic Unit Primary Care Medical School University of Sheffield

Sponsor: University of Oxford
Joint Research Office
1st floor, Boundary Brook House
Churchill Drive,
Headington
Oxford
OX3 7GB

Funder: UKRI/NIHR

Chief Investigator Signature:

Statistician Signature:

No potential conflict of interest

Confidentiality Statement

This document contains confidential information that must not be disclosed to anyone other than the Sponsor, the Investigator Team, HRA, host organisation, and members of the Research Ethics Committee and Regulatory Authorities unless authorised to do so.

See *supplementary material B* for **Key Trial Contacts**.

© Copyright: The University of Oxford and Oxford University Hospitals NHS Foundation Trust 2019

Platform Randomised trial of INterventions against COVID-19 In older peoPLE (PRINCIPLE): Overview

Background: There is an urgent need to identify effective treatments for SARS-CoV-2 infection, that reduce the need for hospital admission and reduce the time to recovery. We have established an open, adaptive, platform trial to evaluate treatments suitable for use in the community for treating COVID-like-illness that might prevent hospitalisation and/or death and help people recover sooner.

Eligibility and randomisation: This protocol describes a randomised trial for people in the community aged 65 and over, or 50 and over with comorbidity, with possible (in accordance with the United Kingdom's Chief Medical Officer's syndromic case definition) or confirmed SARS-CoV-2 infection. Participants are randomised to receive either usual care or a trial treatment (see appendices for details of all trial arms). Participants can take part in the study if they are eligible to be randomised to at least one intervention arm as well as the Usual Care arm.

Platform trial: A "platform trial" is a trial in which multiple treatments for the same disease are tested simultaneously. New interventions can be added or replace existing ones during the course of the trial in accordance with pre-specified criteria.

Response adaptive randomisation: The initial randomisation ratio is fixed 1:1 for a comparison between two trial arms, but the trial has the capability for these proportions to be altered according to participants' responses to interventions. Pre-specified decision criteria allow for dropping a treatment for futility, declaring a treatment superior, or adding a new treatment to be tested. If at any point a treatment is deemed superior to the usual care arm, the superior treatment may replace the usual care arm as the new standard of care. In the context of the COVID-19 pandemic, the trial may continue as long as the pandemic persists.

Outcomes: The trial has co-primary endpoints: 1) Time taken to self-reported recovery; and 2) hospitalisation and/or death. The main objective of the trial is to assess the effectiveness of the interventions in reducing time to recovery and in reducing the incidence of hospitalisation and/or death.

Key secondary outcomes include: Hospital assessment without admission; Oxygen administration; Intensive Care Unit admission; Mechanical ventilation (components of the WHO Clinical Progression Ordinal Scale); Duration of hospital admission; Duration of severe symptoms; Sustained recovery; Contacts with the health services; Consumption of antibiotics; Effects in those with a positive test for COVID-19 infection; WHO Well-being Index.

See *supplementary material C* for details of objectives and outcome measures.

Efficient study design: All enrolment (screening, informed consent, eligibility review and baseline data) and follow-up procedures (daily diary, hospitalisations and deaths) can be performed and captured online on the trial website or by telephone with a member of the trial team. Randomisation is online and automatic following eligibility confirmation. Participant packs and medications are sent from the central study team directly to the participant.

© Copyright: The University of Oxford and Oxford University Hospitals NHS Foundation Trust 2019

Data to be recorded: We will capture demographic features including ethnicity and care home residency at baseline. In the online daily diary (completed for 28 days)/ during telephone calls, participants or their Study Partners will rate the severity of symptoms, record contacts with the health services (including hospital admission), record medication use, and new infections in the household. The WHO-5 Wellbeing Index, a five-question instrument, will assess wellbeing at baseline and on days 14 and 28. Follow-up beyond 28 days after randomisation will be accessing electronic medical records and by participant questionnaire for information relevant to the longer term consequences of COVID-19.

Numbers to be randomised: The trial will continue until either superiority or futility is claimed for each intervention. We estimate that approximately 400 participants per arm (800 participants total if only a single intervention vs. Usual Care) will be required to provide 90% power for detecting an approximate difference of 2 days in median recovery time. We estimate that approximately 1500 participants per arm (3000 participants total if only a single intervention vs. usual care) will be required to provide 90% power for detecting a 50% reduction in the relative risk of hospitalisation and/or death.

To enquire about the trial, contact the PRINCIPLE Trial Team:

PRINCIPLE Trial
Nuffield Department of Primary Care Health Sciences
Radcliffe Primary Care
Radcliffe Observatory Quarter, Woodstock Road
Oxford
OX2 6GG

Email Address: principle@phc.ox.ac.uk

Tel: 0800 1385451
Website: www.principletrial.org

© Copyright: The University of Oxford and Oxford University Hospitals NHS Foundation Trust 2019

TABLE OF CONTENTS

1. BACKGROUND and RATIONALE 9
2. TRIAL DESIGN AND PROCEDURES 9
2.1 Participant Identification 9
2.1.1 Trial Participants 9
2.1.2 Inclusion Criteria 9
2.1.3 Exclusion Criteria 10
2.2 Trial procedures 10
2.2.1 Recruitment 10
2.2.2 Face to face 10
2.2.3 Remote recruitment 10
2.3 Screening 11
2.4 Informed Consent 12
2.5 Eligibility Assessment 13
2.6 Randomisation 13
2.7 Blinding and code-breaking 13
2.8 Baseline Assessments 13
2.9 Subsequent Visits 14
2.10 Qualitative Sub-study 14
2.11 Early Discontinuation/Withdrawal of Participants 15
2.12 Definition of End of Trial 15
3 TRIAL INTERVENTIONS 15
4 SAFETY REPORTING 15
4.1 Procedures for Reporting Adverse Events and Serious Adverse Events 16
4.1.1. Other events exempt from immediate reporting as SAEs 16
4.1.2. Procedure for immediate reporting of Serious Adverse Events 16
4.1.3 Expectedness and Causality 16
Assessment of Causality 17
4.2 SUSAR Reporting 17
4.3 Development Safety Update Reports 17
5 STATISTICS 17
5.1 Master Statistical Analysis Plan (M-SAP) 17
5.2 Open Adaptive Platform Trial 17
5.2.1 Co-Primary Endpoints & Analyses 18
5.2.2 Adaptive Design 19

© Copyright: The University of Oxford and Oxford University Hospitals NHS Foundation Trust 2019

5.2.3 Interim Analyses ... 19
5.2.4 Allocation & Response Adaptive Randomisation ... 20
5.2.5 Sample Size Justification ... 20
5.2.6 Virtual Trial Simulations ... 20
5.2.7 Procedure for Accounting for Missing, Unused, and Spurious Data ... 21
5.3 Primary Analysis Population ... 21
5.4 Procedures for Reporting Unplanned Deviation(s) from the Master Statistical Analysis Plan 21
5.5 Qualitative sub-study analysis ... 21
6 DATA MANAGEMENT ... 21
6.1 Source Data ... 21
6.2 Access to Data ... 22
6.3 Data Recording and Record Keeping ... 22
7 QUALITY ASSURANCE PROCEDURES ... 22
7.1 Risk assessment and Monitoring ... 22
7.2 Trial committees ... 23
8 PROTOCOL DEVIATIONS ... 23
9 SERIOUS BREACHES ... 23
10 ETHICAL AND REGULATORY CONSIDERATIONS ... 24
10.1 Declaration of Helsinki ... 24
10.2 Guidelines for Good Clinical Practice ... 24
10.3 Approvals ... 24
10.4 Other Ethical Considerations ... 24
10.5 Reporting ... 24
10.6 Transparency in Research ... 24
10.7 Participant Confidentiality ... 25
10.8 Expenses and Benefits ... 25
11 FINANCE AND INSURANCE ... 25
11.1 Funding ... 25
11.2 Insurance ... 25
11.3 Contractual arrangements ... 25
12 PUBLICATION POLICY ... 25
13 DEVELOPMENT OF A NEW PRODUCT/ PROCESS OR THE GENERATION OF INTELLECTUAL PROPERTY ... 26
14 ARCHIVING ... 26
21 REFERENCES ... 27
22 APPENDIX A: SCHEDULE OF PROCEDURES ... 31
23 APPENDIX B: AMENDMENT HISTORY ... 34

© Copyright: The University of Oxford and Oxford University Hospitals NHS Foundation Trust 2019

24 APPENDIX C: USUAL CARE ARM ... 37
1. Background and rationale ... 37
2. Changes to outcome measures ... 37
3. Detail of intervention ... 37
a. Investigational Medicinal Product (IMP) description ... 37
b. Storage of IMP ... 37
4. Safety reporting ... 37
25 APPENDIX D: USUAL CARE PLUS HYDROXYCHLOROQUINE ARM (DISCONTINUED) ... 38
1. Background and rationale ... 38
a. Evidence for potential Hydroxychloroquine benefits in COVID-19 ... 38
2. Eligibility criteria specifically related to hydroxychloroquine ... 42
3. Outcome measures related to hydroxychloroquine ... 43
4. Detail of intervention ... 43
a. Investigational Medicinal Product (IMP) description ... 43
b. Storage of IMP ... 43
c. SmPC precautions and concomitant medication ... 44
i. Precautions ... 44
ii. Concomitant medication ... 44
iii. Pregnancy and Breastfeeding ... 45
5. Safety reporting ... 45
26 APPENDIX E: USUAL CARE PLUS AZITHROMYCIN ARM ... 46
1. Background and rationale ... 46
a. Evidence for potential Azithromycin benefits in COVID-19 ... 46
b. Importance of treating CAP or CAP risk in the elderly or immuno-compromised ... 46
2 Changes to outcome measures ... 47
3 Eligibility criteria specifically related to azithromycin ... 47
4 Detail of intervention ... 47
a. Investigational Medicinal Product (IMP) description ... 48
b. Storage of IMP ... 48
c. SmPC precautions and concomitant medication ... 48
i. Precautions ... 48
ii. Concomitant medications ... 48
iii. Fertility, pregnancy and lactation ... 52
5 Safety reporting ... 52
26 APPENDIX F: USUAL CARE PLUS DOXYCYCLINE ARM ... 53
1. Background and rationale ... 53
a. Evidence for potential doxycycline benefits in COVID-19 ... 53

© Copyright: The University of Oxford and Oxford University Hospitals NHS Foundation Trust 2019

b. Importance of treating CAP or CAP risk in the elderly or immuno-compromised 53
2. Changes to outcome measures 54
3. Eligibility criteria specifically related to doxycycline 54
4. Detail of intervention 54
a. Investigational Medicinal Product (IMP) description 54
b. Storage of IMP 55
c. SmPC precautions and concomitant medication 55
i. Precautions 55
ii. Concomitant medications 55
5. Safety reporting 55
26 APPENDIX G: USUAL CARE PLUS INHALED CORTICOSTEROID (ICS) ARM 56
1. Background and rationale 56
a. Evidence for potential benefits of inhaled corticosteroids in COVID-19 illness............... 56
2. Changes to outcome measures 57
3. Eligibility criteria specifically related to ICS 57
4. Detail of intervention 57
a. Investigational Medicinal Product (IMP) description 57
b. Storage of IMP 57
c. SmPC precautions and concomitant medication 57
iii. Precautions 57
iv. Concomitant medications 57
5. Safety reporting 58
27. Supplementary Material 59
A. Abbreviations 59
B. Key Trial Contacts 60
C. Objectives and Outcome Measures 62
D. Adverse Events 64
Definitions 64
E. Data Recording and Record Keeping 65
F. Qualitative Sub-study 65

© Copyright: The University of Oxford and Oxford University Hospitals NHS Foundation Trust 2019

1. BACKGROUND and RATIONALE

We urgently need to know whether potential interventions for COVID-19-like-illness that are suitable for use in the community might help affected individuals recover more quickly and reduce the risk of hospitalisation and/or death. PRINCIPLE is a platform trial designed to efficiently evaluate potential treatments for people with COVID-19-like-illness, and who may be at higher risk of poorer outcomes. Eligible participants are those who meet the UK Chief Medical Officer's definition of COVID-19 illness, who are being managed in the community, and who are aged 50 and over with certain comorbidities, and those aged 65 and over (1-4).

The platform trial has the flexibility to allow additional interventions to be added in, or to replace existing interventions according to pre-specified criteria. If at any point a treatment is deemed superior to the usual care arm, the superior treatment may replace the usual care arm as the new standard of care. All approved intervention arms are outlined in Intervention Specific Appendices (ISAs).

The trial has co-primary endpoints: 1) Time taken to self-reported recovery; and 2) Hospitalisation and/or death. The main objective of the trial is to assess the effectiveness of the respective interventions in reducing time to recovery and in reducing the incidence of hospitalisation and/or death.

The primary analysis will be by "intention to treat", with secondary "intention to treat infected" analyses based on identified aetiology. Clinical data, and information from swab and blood tests, where available, will be used to classify participants according to aetiology.

2. TRIAL DESIGN AND PROCEDURES

PRINCIPLE is an open, prospective, individually randomised, platform, response adaptive, controlled clinical trial in community care.

2.1 Participant Identification

2.1.1 Trial Participants

The trial aims to include participants aged 50 and over with certain comorbidities, and those aged 65 and over with confirmed or possible COVID-19 who meet the case definition for COVID-19, and who are well enough to remain in the community. This definition can be found here: *https://www.gov.uk/government/publications/wuhan-novel-coronavirus-initial-investigation-of-possible-cases/investigation-and-initial-clinical-management-of-possible-cases-of-wuhan-novel-coronavirus-wn-cov-infection*

The study is for people who have ongoing symptoms.

2.1.2 Inclusion Criteria

- Participant, or their legal representative, is willing and able to give informed consent for participation in the study;
- Participant is willing to comply with all trial procedures;

© Copyright: The University of Oxford and Oxford University Hospitals NHS Foundation Trust 2019

- SARS-CoV-2 infection (suspected due to symptoms or laboratory confirmed). Onset of symptoms or a **positive test** for SARS-Co-V2 infection with symptoms of COVID-19 must be within the last 14 days.
- Age criteria: Patients aged ≥65, or Patients aged ≥50-64 years and meeting at least one of the following criteria:
- Known weakened immune system due to a serious illness or medication (e.g. chemotherapy);
- Known heart disease and/or a diagnosis of high blood pressure;
- Known asthma or lung disease;
- Known diabetes;
- Known mild hepatic impairment;
- Known stroke or neurological problem;
- Self-report obesity or body mass index ≥35 kg/m^2

2.1.3 Exclusion Criteria

- Patient currently admitted in hospital
- Almost recovered (generally much improved and symptoms now mild or almost absent)
- Judgement of the recruiting clinician deems ineligible.
- Previous randomisation to an arm of the PRINCIPLE trial

Additional exclusions specific to each intervention arm are listed in the ISAs. For participation, participants must be eligible to be randomised to at least one intervention arm as well as the Usual Care arm.

2.2 Trial procedures

2.2.1 Recruitment

Recruitment is possible through a variety of mechanisms:

2.2.2 Face to face

Attending clinicians, including research nurses or other health care professionals, at general medical practices, paramedic services, hospital emergency departments, clinical care hubs, Hospital at Home facilities, care of the elderly services, pharmacies, social care services, residential and nursing homes, or any health and social care facility, can facilitate recruitment into the trial. They can do this by discussing the study with potentially eligible participants, guiding them through informed consent procedures, collection of baseline data, completion of screening questions, collecting information for eligibility assessment, and randomising the participant. If required and appropriate, licensed prescribers may prescribe the medication appropriate to the group to which the participant is randomised. Alternatively, health care professionals may revert to the PC-CTU to complete the activity, including eligibility confirmation and issue of study medication and materials.

2.2.3 Remote recruitment

© Copyright: The University of Oxford and Oxford University Hospitals NHS Foundation Trust 2019

i) All Health, health related, and Social Care professionals will be able to give information verbally or via a trial text, email, poster, social media post, adverts, media release, leaflet or letter, to potential study participants and their study partners. They may also direct patients to the online study information and the study website.

ii) Potential participants may present directly to the study team via the website or by the study telephone contact. The study team can provide information about joining the trial and guide them through the consent and enrolment process.

iii) A General Practice may be contacted by a potential participant or the practice may contact patients, by text (or by letter), who may match the trial eligibility criteria, through running searches of their database. They will then direct patients to the trial enrolment website or seek verbal consent to be contacted by the trial team.

iv) NHS Digital will provide the PRINCIPLE trial with a daily list of contact details from the COVID-19 testing Pillar 2 data, for patients receiving a positive test result for SARS-Co-V2 infection, via a secure transfer system. NHS digital will apply an age filter to ensure only the details of those within the age range of the trial are passed on to PRINCIPLE. The trial team will make a limited number (maximum of 3) of attempts to telephone, text or email these patients to provide them with information about the trial, to invite them to consider taking part, and to enrol them if they provide full informed consent and are deemed eligible at screening.

Patient details will be provided in accordance with section 251 under the General Notice under the Health Service Control of Patient Information Regulations 2002, which applies only in England and Wales, providing patient information without consent for COVID-19 public health, surveillance and research purposes. The notice provides a temporary legal basis to avoid a breach of confidentiality for COVID-19 purposes.

For all recruitment models:

- Study Partner: at screening the potential participant will be asked to provide contact details for a Study Partner, to assist in completing trial procedures and to provide information on their behalf where necessary, but this is not a requirement for trial participation. However, it is strongly encouraged that participants who may be frailer and/or lack capacity to consent make use of a study partner to facilitate their participation. In addition to family member or friend, the study partner may also be a carer or other suitable person.
- Participants may be asked if they wish to enrol in additional studies that do not conflict with the main PRINCIPLE trial. Those who do not screen as eligible for PRINCIPLE may be alerted to the possibility of participating in other approved trials.

2.3 Screening

An online screening, eligibility and consent procedure is used. If online access is not possible, a member of the trial team collects this information during a telephone call. A trial free-phone number enables participants to contact the trial team for further information and study participation support. Participants are screened after they have read the PIS by completing an online eligibility questionnaire.

© Copyright: The University of Oxford and Oxford University Hospitals NHS Foundation Trust 2019

2.4 Informed Consent

If participants meet the screening criteria, they will be asked to provide informed consent and a screening trial ID number will be assigned to them. Remote, paperless online/telephone consent is required, and appropriate during the pandemic. Participants will be able to download their consent form, or it may be printed by the central study team and delivered to participants with their study materials if they so prefer.

Written and verbal versions of the PIS and ICF will be presented to participants detailing no less than: the exact nature of the trial; the known side-effects and risks involved in taking part. It will be clear that the participant is free to withdraw from the study at any time. A summary, pictorial PIS is available which can be read by those feeling very unwell, lack capacity or have low reading comprehension skills. Adequate time will be given to the participant to consider the information and to ask any questions about the trial before deciding whether to participate. After consent, the participant will enter online baseline information, including their address, contact details and those of a Study Partner.

Population groups such as care home residents have been amongst those hardest hit by the pandemic and therefore stand to benefit the most from any effective treatments. However, some care home residents lack capacity to consent to research themselves. If the recruiting clinician deems a care home resident lacks capacity to consent then a personal or professional legal representative (England and Wales only) will be asked to provide consent for those lacking capacity to consent for themselves. A personal legal representative is defined as a person not connected with the conduct of the trial who is suitable to act as the legal representative by virtue of their relationship with the adult. A professional legal representative may be a doctor responsible for the medical treatment of the adult if they are independent of the study, or a person nominated by the healthcare provider. In all instances, a personal legal representative will be sought first and a professional legal representative sought only if a personal legal representative cannot be identified. A professional legal representative will be sought in order not to deny access to research to older adults who may not have personal legal representatives. Only residents of care homes who lack capacity to consent will be recruited, adults who lack capacity to consent will not be recruited from the wider community. Residents who, in addition to their lack of capacity, have a quality of life which can reasonably be seen as not acceptable to them will not be recruited

The legal representative will be provided with information about the trial and made aware of the following:

- They are being asked to give consent on behalf of the incapacitated adult,
- They are free to decide whether they wish to make this decision or not, and
- They are being asked to consider what the adult would want, and to set aside their own personal views when making this decision.

© Copyright: The University of Oxford and Oxford University Hospitals NHS Foundation Trust 2019

2.5 Eligibility Assessment

Eligibility of those who have provided appropriate consent can be checked at study sites or centrally by a medically qualified clinician or a research nurse, who is suitably trained and experienced and has been delegated this responsibility, and who has appropriate access to the participant's summary care record or relevant medical information. If a participant's summary care record cannot be accessed centrally, the clinician/delegate will contact the participant's primary care medical practice for information relevant to confirming eligibility. Participants will not be randomised to an arm if an exclusion criterion to that arm applies to them, but will need to have no exclusions relevant to at least one intervention and the usual care arm.

2.6 Randomisation

Participants will be randomised using a fully validated and compliant web-based randomisation system called Sortition. Once deemed eligible, the clinician or a member of the trial team will randomise the participant, to one of the arms they are eligible for (at least two arms), automatically by Sortition. Full details of response adaptive randomisation are described in section 5.2.

The participant, legal representative if applicable, trial team and participant's GP will be notified electronically of the treatment allocation. If the participant does not have an email address, they will be notified by telephone. The research team may also send the GP or Care Home an email or letter via secure systems, containing personally identifiable data and treatment allocation.

2.7 Blinding and code-breaking

PRINCIPLE is an open-label trial. The participant, legal representative if applicable and the recruiting clinician will know the participant's allocation. Therefore, no unblinding or code breaking is required. However, those managing the data will be blind to participant allocation.

The trial team and recruiting clinicians will be blinded to emerging results. During the course of the trial, only those on the Data Safety and Monitoring Committee will have access to the unblinded interim results.

2.8 Baseline Assessments

Once randomised, study medication (if so randomised), and a participant pack will be sent to participants, from their general practice, study team, Public Health England (PHE) or other approved central service (or collected from a general practice or pharmacy). Participants may be offered a swab test as part of standard care. Where possible, and availability of sampling kits allows, one sample will be taken as close to study entry as possible to assess COVID-19 status and other viral aetiologies. While the aim is to have a swab result for all patients, where swabs are unavailable, patients may still participate and be included in the primary intention to treat analysis only.

© Copyright: The University of Oxford and Oxford University Hospitals NHS Foundation Trust 2019

2.9 Subsequent Visits

There is no requirement for participants to have a face-to-face visit as part of trial participation. All subsequent measurements consist of self-completed questionnaires online or through telephone calls, and primary care and/or hospital record searches. We will ascertain relevant data from primary care and/or hospital medical records about length of hospital stay, oxygen therapy, and ICU admission and ventilation, if applicable.

Participants will be sent a link to their online diary, which they will be asked to complete for 28 days. They will be asked to rate the severity of symptoms, record contacts with the health services (including hospital admission), record medication use and new infections in the household. It is becoming increasingly apparent the COVID-19 infection may have a considerable negative impact on well-being (14) and so the five questions of WHO-5, validated for measuring wellbeing over time, will be presented at baseline and on days 14 and 28. We will not ask for WHO-5 questions to be completed for participants who lack capacity. We will capture ethnicity and care home residency at baseline and day 28 (if missed at baseline).

All participants receive a call from the trial team on day 2/3 to confirm that they have received a participant pack, and trial medication (if randomised to a trial medication), and to explain that they should complete the daily diary for 28 days even if they feel better or their swab result is negative. The trial team calls participants/study partners on days 7, 14 and 28 if they do not have internet access or have *not* completed their diary for at least 2 consecutive days prior to the call. No more than six contact attempts will be made at each of these follow-up points.

We will seek consent from participants to contact them on a monthly basis for up to 12 months after enrolment (via email, text message or phone call) to collect information about any ongoing symptoms, hospitalisations and well-being. We will re-consent those already enrolled in the trial.

In addition to the swab being undertaken as part of the national RCGP RSC surveillance programme with PHE, trial participants will also be asked to consent to the trial team accessing a blood sample result. The study team will obtain the result from RCGP RSC/PHE.

The RCGP RSC will report to the central trial office at regular intervals about healthcare contacts in the participant's clinical records, as they are able to download this information centrally. This will be used as confirmation and a back-up for information obtained directly from study participants and other data sources outlined above. If obtaining data is not possible using this route, the GP surgery will be contacted to request a limited notes review. Participant records will be accessed up to twelve months following enrolment to ascertain follow up data from enrolment to day 28. Data will be collected as close to real time as possible; RCGP RSC, EMIS and NHS Digital and other sources of routinely collected data will be utilised if required To investigate the impact of trial interventions on the longer-term effects of COVID-19, we will use these data collection methods to follow-up participants, for up to 10 years.

2.10 Qualitative Sub-study

© Copyright: The University of Oxford and Oxford University Hospitals NHS Foundation Trust 2019

A qualitative sub-study will be nested within the trial, to capture data to understand how patients conceptualise their illness and how they respond to taking medication(s) as part of the trial. Once participants have completed the trial, we will interview their respective clinicians to explore their views of taking part in trials during a pandemic. Healthcare professionals will also be asked about their experiences of taking part in the trial. See *supplementary material E* for further details. Participants who lack capacity will not be invited to participate in the qualitative sub study.

2.11 Early Discontinuation/Withdrawal of Participants

Each participant, or their legal representative on the participant's behalf, has the right to withdraw from the study at any time. For those that lack capacity, expression of dissent in any form will be taken as an indication they do not wish to be included and they will be withdrawn. In addition, the Investigator may discontinue a participant from the study at any time if the Investigator considers it necessary for any reason including:

- Ineligibility (either arising during the study or retrospectively having been overlooked at screening)
- Withdrawal of consent

The reason for withdrawal will be recorded on the CRF. Data that has already been collected about the participant will be kept and used.

2.12 Definition of End of Trial

Last data capture of last participant, when: no further suitable interventions are available and/or COVID-19 is no longer prevalent. March 2022 has been decided as the formal end date at this stage, but this date may need to be amended depending on circumstances prevailing at the time.

3 TRIAL INTERVENTIONS

IMP information can be found in the relevant ISAs.

In general, re-packaging and issuing of medication can be completed by: the patient's registered GP surgery or treatment and assessment facility; an accredited licensed central facility; an online, community or hospital pharmacy, and The Primary Care Clinical Trials Unit (as approved by the MHRA). Distribution of trial packs to participants will be tracked via courier or call/text message. Clinicians can prescribe trial medications that can be issued in the community and pharmacies can issue medication to the patient by community pharmacy services 'on-line pharmacy' services, NHS volunteers, or it can be collected from the pharmacy by the participant or someone on their behalf.

To record presence of symptoms and severity, as well as adherence to trial treatment, participants will receive a daily email asking them to complete an online diary where they will record their symptoms and medicines use. If incomplete, the trial team will contact the participant and/or their Study Partner to obtain the data. A risk-adapted approach will be used for drug accountability. Accountability logs will be kept by PC-CTU when they ship drug.

4 SAFETY REPORTING

© Copyright: The University of Oxford and Oxford University Hospitals NHS Foundation Trust 2019

All symptoms, medication side-effects and SAEs will be collected from participant daily diaries, calls to participants/Study Partners, medical records, notes reviews and RCGP data downloads. SAE information will be analysed as part of the interim and whole trial analysis and will be reviewed at each Data Safety & Monitoring Committee meeting.

4.1 Procedures for Reporting Adverse Events and Serious Adverse Events

The severity of events will be assessed by participants in daily diaries on the following scale: minor problem/moderate problem/major problem. Serious Adverse Events (SAE), but not Adverse events (AE), will be assessed for causality and expectedness in the trial. A participant may voluntarily withdraw from treatment due to what he or she perceives as an intolerable AE.

Hospitalisation and/or death due to confirmed or possible SARS-Cov-2 infection is a primary outcome, we will collect this data using a risk-adapted approach and will not report such SAEs. SAEs other than hospitalisation or death due to COVID-19 must be reported by the person who has discovered the SAE or nominated delegate within 24 hours of becoming aware of the event. The sponsor or delegate will ensure it is reviewed by the CI or other delegated personnel for relatedness and expectedness as soon as possible taking into account the reporting time for a potential SUSAR according to the relevant competent authority. If the event has not resolved, at the 28 day time point the SAE will be reviewed again to see if resolution has occurred. If the event is considered ‘resolved’ or ‘resolving’ no further follow up is required. If not, the event must be followed up until such a time point.

See Appendix C for definitions of adverse events

4.1.1. Other events exempt from immediate reporting as SAEs

Hospitalisations will be defined as at least a one night admission to hospital. Hospitalisation for a pre-existing condition, including elective procedures planned prior to study entry, which has not worsened, does not constitute an SAE, and standard supportive care for the disease under study are not SAEs and do not require SAE reporting.

4.1.2. Procedure for immediate reporting of Serious Adverse Events

- Trial team will complete an SAE report form for all reportable SAEs.
- GP practice/trial team/RCGP will provide additional, missing or follow up information in a timely fashion.

The CI or delegate will review the SAE once reported, collect as much information and report to the Sponsor within the timeframe according to the PC-CTU SOPs.

4.1.3 Expectedness and Causality

For SAEs that require reporting, expectedness of SARs will be determined according to the relevant RSI section of the Summary of Product Characteristics/IB. The RSI will be the current Sponsor and MHRA approved version at the time of the event occurrence.

© Copyright: The University of Oxford and Oxford University Hospitals NHS Foundation Trust 2019

Assessment of Causality

The relationship of each serious adverse event to the trial medication must be determined by a medically qualified individual according to the following definitions:

- **Unrelated** – where an event is not considered to be related to the IMP
- **Possibly** – although a relationship to the IMP cannot be completely ruled out, the nature of the event, the underlying disease, concomitant medication or temporal relationship make other explanations possible.
- **Probably** – the temporal relationship and absence of a more likely explanation suggest the event could be related to the IMP.
- **Definitely** – the known effects of the IMP, its therapeutic class or based on challenge testing suggest that the IMP is the most likely cause.

All SAEs labelled possibly, probably or definitely will be considered as related to the IMP.

4.2 SUSAR Reporting

All SUSARs will be reported by the sponsor delegate to the relevant Competent Authority and to the REC and other parties as applicable. For fatal and life-threatening SUSARS, this will be done no later than seven calendar days after the Sponsor or delegate is first aware of the reaction. Any additional relevant information will be reported within eight calendar days of the initial report. All other SUSARs will be reported within 15 calendar days.

Principal Investigators will be informed of all SUSARs for the relevant IMP for all studies with the same Sponsor, whether or not the event occurred in the current trial.

4.3 Development Safety Update Reports

The DSUR will be developed and submitted annually on the anniversary date that the trial receives Clinical Trial Authorisation +60 days. Due to the nature of this trial and the importance of sharing the science of COVID-19 and the drug, internationally, we expect to produce reports to the UK Government and regulatory agency more frequently upon request.

5 STATISTICS

5.1 Master Statistical Analysis Plan (M-SAP)

Details of the statistical design and methods will be described in a Master Statistical Analysis Plan (M-SAP), in which an appendix to the M-SAP titled "Adaptive Design Report" (ADR) provides complete specifications for the primary analysis and pre-specified adaptive algorithm. In addition, the M-SAP will be accompanied by arm-specific appendices to describe any planned deviations from the M-SAP. A broad overview of the design and primary analyses is provided below.

5.2 Open Adaptive Platform Trial

PRINCIPLE is an open, adaptive, platform trial to evaluate emerging treatments for symptomatic COVID-19-like illness. A "platform trial" is a trial in which multiple treatments for the same

© Copyright: The University of Oxford and Oxford University Hospitals NHS Foundation Trust 2019

disease are tested simultaneously. The backbone of the trial is an adaptive clinical trial design. Pre-specified decision criteria allow for dropping a treatment for futility, declaring a treatment superior, or adding a new treatment to be tested. If at any point a treatment is deemed superior to the Usual Care arm, the superior treatment may replace the Usual Care arm as the new standard of care. Because the process of dropping and adding treatments may be on-going for an indefinite period of time, platform trials may be better conceived of as a process rather than a singular clinical trial. In the context of the COVID-19 pandemic, the trial may continue as long as the pandemic persists.

The PRINCIPLE trial will begin as a two arm, 1:1 randomised trial but will have the capability to add additional interventions over time. The evaluation of any new interventions will be governed by this master protocol and M-SAP (including adaptive algorithm and decision criteria), with any planned deviations from the master protocol and M-SAP to be specified in arm-specific appendices. The inclusion of any new interventions will require additional arm-specific appendices to the master protocol and M-SAP.

5.2.1 Co-Primary Endpoints & Analyses

There are two co-primary endpoints. The first co-primary endpoint is time to recovery from possible COVID-19 infection within 28 days from randomization, where time to recovery is defined as the first instance that a participant reports feeling recovered. The second co-primary endpoint is hospital admission or death related to possible or confirmed COVID-19 within 28 days from randomization. Unless otherwise specified in the ISAs, the co-primary outcomes will be evaluated using a “gate-keeping” strategy. For a given treatment, the hypothesis for the time to recovery endpoint will be evaluated first, and if the recovery null hypothesis is rejected, the hypothesis for the second co-primary endpoint of hospitalization/death will be evaluated. This gate-keeping strategy preserves the overall Type I error of the primary endpoints without additional adjustments for multiple hypotheses. In addition, the gate-keeping structure reflects the clinical belief that an intervention is unlikely to demonstrate benefit on the hospitalization/death endpoint without first demonstrating benefit on the time to recovery endpoint.

The primary outcome of time to recovery is defined as the first instance that a participant reports feeling recovered. The corresponding primary analysis for this outcome is a Bayesian piecewise exponential model, with time to recovery regressed on treatment and stratification covariates (age, comorbidity). Let θ_j denote the log hazards ratio comparing the hazards of recovery for participants in treatment group *j* versus participants in the Usual Care arm. A corresponding Bayesian posterior distribution will be derived for the estimated log hazards ratio. The first co-primary analysis for intervention *j* will test the following hypothesis:

$$H_{10}: \theta_j \leq 0$$
$$H_{11}: \theta_j > 0$$

If the Bayesian posterior probability of superiority (a log hazards ratio greater than 0 corresponding to quicker recovery) for a treatment versus Usual Care is sufficiently large (e.g. ≥ 0.99), the null hypothesis will be rejected and the intervention will be deemed superior to Usual Care with respect to time to recovery. The exact threshold of the superiority decision criterion (e.g. 0.99) will be determined *a priori* via simulation to control the one-sided Type I error of the

© Copyright: The University of Oxford and Oxford University Hospitals NHS Foundation Trust 2019

study at approximately 0.025, and will be specified in the Adaptive Design Report (Appendix to the M-SAP). The Adaptive Design Report will also specify appropriate methodology for the primary analysis when the Usual Care arm is replaced by a superior treatment, and for when the comparison of a treatment versus Usual Care includes non-concurrent randomisations.

The second co-primary endpoint is hospital admission or death due to possible SARS-CoV-2 infection. The corresponding analysis will be a Bayesian generalised linear model of hospitalisation/death regressed on treatment and stratification covariates (age, comorbidity). Let δ_j denote the log odds ratio comparing the odds of hospitalisation/death for persons in treatment group *j* versus persons in the Usual Care arm. A corresponding Bayesian posterior distribution will be derived for the estimated log odds ratio. If the first co-primary endpoint hypothesis (for time to recovery) is rejected for intervention *j*, the second co-primary hypothesis for intervention *j* be tested:

$$H_{20}: \delta_j \leq 0$$
$$H_{21}: \delta_j > 0$$

If the Bayesian posterior probability of superiority on hospitalization/death for a treatment versus Usual Care is sufficiently large (e.g. ≥ 0.99), the null hypothesis will be rejected and the intervention will be deemed superior to Usual Care with respect to hospitalization/death. The exact threshold of the superiority decision criterion (e.g. 0.99) will be determined *a priori* via simulation to control the one-sided Type I error of the study at approximately 0.025, and will be specified in the M-SAP.

5.2.2 Adaptive Design

The pre-specified design will allow adaptations to the trial based on the observed co-primary endpoint data. These adaptations include the declaration of success or futility of an intervention at an interim analysis, the addition or removal of treatment arms, and changes in the randomisation probabilities. Adaptations will occur at a given interim analysis if pre-specified conditions are satisfied. The adaptive algorithm will be documented in the Adaptive Design Report, including pre-specified criteria for decisions regarding futility or effectiveness of interventions and/or replacing interventions in the trial.

5.2.3 Interim Analyses

Precise timing of the first interim analysis and frequency of subsequent interim analyses will be specified in the Adaptive Design Report, based on both simulations and logistical considerations. At each interim analysis, all enrolled intervention arms will be evaluated for success and futility on both co-primary endpoints using the Bayesian primary analyses. These interim analyses will maintain the gate-keeping sequential order by first evaluating the hypothesis for time to recovery, and if the recovery endpoint null hypothesis is rejected, subsequently evaluating the hypothesis for hospitalisation and/or death. If the Bayesian posterior probability of superiority of a given intervention versus Usual Care is sufficiently large for a given endpoint (e.g. ≥ 0.99) within the gate-keeping structure, superiority will be declared versus Usual Care with respect to that endpoint.

© Copyright: The University of Oxford and Oxford University Hospitals NHS Foundation Trust 2019

If the Bayesian posterior probability of a clinically meaningful treatment effect is sufficiently small (e.g. < 0.01) for the first co-primary endpoint (time to recovery), the intervention arm may be dropped from the study for futility. If there are no other intervention arms available, the trial may be suspended; otherwise accrual continues to the remaining treatment arms. The exact futility thresholds will be pre-specified in the Adaptive Design Report and determined via simulation.

5.2.4 Allocation & Response Adaptive Randomisation

Initially, randomisation will be fixed 1:1 for a comparison between two trial arms, with stratification by age (less than 65, greater than or equal to 65), and comorbidity (yes/no). If a second experimental intervention arm is added to the study, randomisation allocation will be modified and the additional intervention will be included in the interim analyses (with evaluation for success and futility) as detailed in the Adaptive Design Report. If there are at least 3 arms (2 intervention arms plus Usual Care) in the study, each interim analysis may incorporate modified randomisation probabilities via response adaptive randomisation (RAR). Full details for implementing RAR will be provided in the Adaptive Design Report; the general idea is to allocate more participants to the intervention arms that have the best observed outcomes.

5.2.5 Sample Size Justification

Given the open perpetual trial structure, the trial does not have a finite ending based on sample size. Rather, the trial will continue until either superiority or futility is claimed for each intervention, or until the pandemic expires in the population. We estimate that approximately 400 participants per arm (800 participants total if only a single intervention vs. Usual Care) will be required to provide 90% power for detecting a hazard ratio of 1.3 (approximate difference of 2 days in median recovery time). This calculation is based on the assumption of an exponential distribution for time to recovery with a median of 9 days in the Usual Care arm, with some adjustments for missing data and multiple interim analyses. On average, we expect fewer participants to be required when there is a large treatment benefit or complete lack of benefit. For example, if the true hazard ratio is 1.5 (3 day benefit in median time to recovery), on average only 150 subjects per arm are required to provide sufficient power. The primary advantage of the adaptive design is the ability to adapt the sample size to the observed data, thus addressing the primary hypothesis as quickly and as efficiently as possible.

We estimate that approximately 1500 participants per arm (3000 participants total if only a single intervention vs. usual care) will be required to provide 90% power for detecting a 50% reduction in the relative risk of hospitalisation and/or death. This calculation is based on the assumption of an underlying 5% combined hospitalisation and/or death rate in the Usual Care arm, with an intervention lowering the hospitalisation and/or death rate to 2.5%, with some adjustments for the multiple interim analyses. We expect fewer participants to be required to detect a 50% reduction if the event rate in the Usual Care arm is greater than 5%.

5.2.6 Virtual Trial Simulations

Because of the adaptive platform trial structure, there exists no simple formula(s) to calculate power and Type I error (false positive rate). Hence, virtual trial simulations will be used to fully characterize and quantify the power and Type I error of the design. These simulations will be

© Copyright: The University of Oxford and Oxford University Hospitals NHS Foundation Trust 2019

conducted prior to the first interim analysis (with results described in the Adaptive Design Report), and will be used to optimize the adaptive decision criterion and RAR parameters. The simulations will include a comprehensive evaluation of trial performance across a wide range of assumptions (e.g. underlying distribution of outcome in Usual Care arm, treatment effect, accrual rates, etc.). This will include summaries regarding the number of subjects required to make a success or futility conclusions for each intervention. Complete details of the simulations will be provided in the Adaptive Design Report.

5.2.7 Procedure for Accounting for Missing, Unused, and Spurious Data.

Full details of handling missing data will be specified in the M-SAP.

5.3 Primary Analysis Population

The primary analysis population is defined as all randomised participants according to the groups they were randomly allocated to, regardless of deviation from protocol and irrespective of their COVID-19 status. Secondary analyses will conduct the primary analysis on the subset of participants with confirmed COVID-19.

5.4 Procedures for Reporting Unplanned Deviation(s) from the Master Statistical Analysis Plan

Analyses will be carried out in accordance with the M-SAP and corresponding appendices. Any additional analysis that is not specified in the M-SAP/appendices or any unplanned deviation(s) from the M-SAP/appendices will be specified in the Statistical Report. Reasons for these changes will be documented and authorised by the Chief Investigator.

5.5 Qualitative sub-study analysis

Audio-recordings of interviews will be transcribed verbatim and transcripts analysed using thematic analysis. Patient and HCP interviews transcripts will be analysed separately but findings will be compared and triangulated if deemed appropriate. Thematic analysis allows the research team to take a pragmatic approach to data collection, remaining grounded in the data but ensuring that the analysis answers the research objectives. NVivo software will be used to assist with the organisation and coding of data. Codes will be compared with one another to create categories, grouping similar codes together. A thematic framework will be developed to code all data and represent key themes for both sets of interviews.

6 DATA MANAGEMENT

The data management aspects of the study are summarised here with details fully described in the Data Management Plan.

6.1 Source Data

Source documents are where data are first recorded. These include, but are not limited to, hospital records (from which medical history and previous and concurrent medication may be summarised into the CRF), clinical and office charts, laboratory and pharmacy records, diaries, microfiches, radiographs, and correspondence.

© Copyright: The University of Oxford and Oxford University Hospitals NHS Foundation Trust 2019

If a participant fails to complete data online and after six attempts at contacting the participant/Study Partner, the RCGP RSC may be utilised to obtain missing data. Data collected will include participant identifiable information and will be accessed at the University of Oxford according to PC-CTU Information Governance policies and GDPR. Data will only be held for the duration it is required, this will be reviewed annually.

CRF entries will be considered source data if the CRF is the site of the original recording (e.g. there is no other written or electronic record of data). All documents will be stored safely in confidential conditions. On all study-specific documents, other than the signed consent, the participant will be referred to by the study participant number/code, not by name.

6.2 Access to Data

Direct access will be granted to authorised representatives from the Sponsor and host institution for monitoring and/or audit of the study to ensure compliance with regulations.

6.3 Data Recording and Record Keeping

A CTU data manager will oversee the receiving, entering, cleaning, querying, analysing and storing all data that accrues from the study by designated persons. The Investigators will maintain appropriate medical and research records for this trial, in compliance with the requirements of the Medicines for Human Use (Clinical Trial) Regulations 2004, ICH E6 GCP and regulatory and institutional requirements for the protection of confidentiality of volunteers. The Chief Investigator, Principal Investigator, Co-Investigators, clinical team, including Clinical Research Nurses, and other authorised members of the trial team will have access to records. The Investigators will permit authorised representatives of the sponsor, and regulatory agencies to examine (and when required by applicable law, to copy) clinical records for the purposes of quality assurance reviews, audits and evaluation of the study safety and progress. The software used for the trial is described in *supplementary material D*.

7 QUALITY ASSURANCE PROCEDURES

The study will be conducted in accordance with the current approved protocol, GCP, relevant regulations and PC-CTU Standard Operating Procedures. All PIs, coordinating centre staff and site staff will receive training in trial procedures according to GCP where required. Regular monitoring will be performed according to GCP using a risk-based approach. Data will be evaluated for compliance with the protocol and accuracy in relation to source documents where possible.

The PC-CTU Trial Management Group will be responsible for the monitoring of all aspects of the trial's conduct and progress and will ensure that the protocol is adhered to and that appropriate action is taken to safeguard participants and the quality of the trial itself. The TMG will be comprised of individuals responsible for the trial's day to day management and will meet regularly throughout the course of the trial.

7.1 Risk assessment and Monitoring

© Copyright: The University of Oxford and Oxford University Hospitals NHS Foundation Trust 2019

A risk assessment and monitoring plan will be prepared before the study opens and will be reviewed as necessary over the course of the study to reflect significant changes to the protocol or outcomes of monitoring activities. Monitoring will be performed by the PC-CTU Quality Assurance Manager or delegate. The level of monitoring required will be informed by the risk assessment.

7.2 Trial committees

The responsibilities of each group are as follows:

- Data Monitoring and Safety Committee (DMSC) - to review the data at each interim analysis as described in the Statistical Analysis section, as the updates to the randomisation scheme occur in order to ensure that the process is working correctly and to review and monitor the accruing data to ensure the rights, safety and wellbeing of the trial participants.
- Trial Steering Committee (TSC) - the Trial Steering Committee ensure the rights, safety and wellbeing of the trial participants. They will make recommendations about how the study is operating, any ethical or safety issues and any data being produced from other relevant studies that might impact the trial.
- Trial Management Group (TMG) - is responsible for the day-to-day running of the trial, including monitoring all aspects of the trial and ensuring that the protocol is being adhered to. It will include Co-Investigators and will meet weekly in the first instance.
- A core project team (PT) from within the TMG will meet daily as required for daily operational decision making.

8 PROTOCOL DEVIATIONS

A study related deviation is a departure from the ethically approved study protocol or other study document or process (e.g. consent process or administration of study intervention) or from Good Clinical Practice (GCP) or any applicable regulatory requirements. Any deviations from the protocol will be documented in a protocol deviation form and filed in the study master file.

A PC-CTU SOP is in place describing the procedure for identifying non-compliances, escalation to the central team and assessment of whether a non-compliance /deviation may be a potential Serious Breach.

9 SERIOUS BREACHES

A “serious breach” is a breach of the protocol or of the conditions or principles of Good Clinical Practice which is likely to affect to a significant degree –

(a) the safety or physical or mental integrity of the trial subjects; or
(b) the scientific value of the research.

In the event that a serious breach is suspected the Sponsor must be contacted within one working day. In collaboration with the CI, the serious breach will be reviewed by the Sponsor and, if appropriate, the Sponsor will report it to the approving REC committee and the relevant NHS host organisation within seven calendar days.

© Copyright: The University of Oxford and Oxford University Hospitals NHS Foundation Trust 2019

10 ETHICAL AND REGULATORY CONSIDERATIONS

10.1 Declaration of Helsinki

The Investigator will ensure that this study is conducted in accordance with the principles of the Declaration of Helsinki.

10.2 Guidelines for Good Clinical Practice

The Investigator will ensure that this trial is conducted in accordance with relevant regulations and with Good Clinical Practice.

10.3 Approvals

Following Sponsor approval, the protocol, informed consent form, participant information sheets and any proposed informing material will be submitted to an appropriate Research Ethics Committee (REC), regulatory authorities, and host institution(s) for written approval. The PI and coordinating centres for each country will ensure and confirm correct regulatory approvals are gained prior to recruitment.

The Investigator will submit and, where necessary, obtain approval from the above parties for all substantial amendments to the original approved documents.

10.4 Other Ethical Considerations

If a particular arm is deemed futile and dropped, no further participants will be randomised to this arm and anyone who is currently on this arm will be informed it has been dropped.
Once a particular intervention has been declared superior and effective, that will become the comparator arm (i.e. standard care).

The vast majority of participant's, due to their co-morbidities, will be exempt from prescription charges. All participants will receive a £20 voucher to cover any prescriptions and other expenses they may incur as a consequence of study participation.

Participants who lack capacity to consent for themselves will only be recruited after consultation with their legal representative. Any sign of dissent in any form from the participant who lacks consent will be taken as an indication they do not wish to be involved and they will be withdrawn. Only residents of care homes who lack capacity to consent will be recruited, adults who lack capacity to consent will not be recruited from the wider community.

10.5 Reporting

The CI shall submit once a year throughout the clinical trial, or on request, an Annual Progress Report to the REC, HRA (where required), host organisation, funder (where required) and Sponsor. In addition, an End of Trial notification and final report will be submitted to the MHRA, the REC, host organisation and Sponsor.

10.6 Transparency in Research

Prior to the recruitment of the first participant, the trial will have been registered on a publicly accessible database. Results will be uploaded to the European Clinical Trial (EudraCT) Database

© Copyright: The University of Oxford and Oxford University Hospitals NHS Foundation Trust 2019

within 12 months of the end of trial declaration by the CI or their delegate. Where the trial has been registered on multiple public platforms, the trial information will be kept up to date during the trial, and the CI or their delegate will upload results to all those public registries within 12 months of the end of the trial declaration.

10.7 Participant Confidentiality

The study will comply with the General Data Protection Regulation (GDPR) and Data Protection Act 2018, which require data to be de-identified as soon as it is practical to do so. The processing of the personal data of participants will be minimised by making use of a unique participant study number only on all study documents and any electronic database(s). All documents will be stored securely and only accessible by study staff and authorised personnel. The study staff will safeguard the privacy of participants' personal data.

10.8 Expenses and Benefits

All participants will be reimbursed with a £20 voucher, to cover the payment of a prescription, should they incur this as a result of study participation, and a token of recognition of giving their time and contribution to the study. The vast majority of participants will not have to pay a prescription change, should a prescription be issued as a result of trial participation. Most people with the co-morbidities outlines and in the age-range required for eligibility, are not required to pay for prescriptions. Participants who complete a telephone interview as part of the qualitative sub-study will be reimbursed with a (second) £20 voucher for their time to participate.

11 FINANCE AND INSURANCE

11.1 Funding

The study is funded by the UKRI/NIHR via an MRC call.

11.2 Insurance

The University has a specialist insurance policy in place, which would operate in the event of any participant suffering harm as a result of their involvement in the research (Newline Underwriting Management Ltd, at Lloyd's of London). NHS indemnity operates in respect of the clinical treatment that is provided.

11.3 Contractual arrangements

Appropriate contractual arrangements will be put in place with all third parties.

12 PUBLICATION POLICY

The Investigators (those listed on the protocol and others to be decided at publication) will be involved in reviewing drafts of the manuscripts, abstracts, press releases and any other publications arising from the study. Authors will acknowledge the study funders. Authorship will be determined in accordance with the ICMJE guidelines and other contributors will be acknowledged.

© Copyright: The University of Oxford and Oxford University Hospitals NHS Foundation Trust 2019

13 DEVELOPMENT OF A NEW PRODUCT/ PROCESS OR THE GENERATION OF INTELLECTUAL PROPERTY

Ownership of IP generated by employees of the University vests in the University. The University will ensure appropriate arrangements are in place as regards any new IP arising from the trial.

14 ARCHIVING

Archiving will be done according to the UOXF PC-CTU SOP and study specific working instructions.

© Copyright: The University of Oxford and Oxford University Hospitals NHS Foundation Trust 2019

21 REFERENCES

1. Livingston E, Bucher K. Coronavirus Disease 2019 (COVID-19) in Italy. JAMA. 2020.
2. Wu Z, McGoogan JM. Characteristics of and Important Lessons From the Coronavirus Disease 2019 (COVID-19) Outbreak in China: Summary of a Report of 72314 Cases From the Chinese Center for Disease Control and Prevention. JAMA. 2020.
3. Paules CI, Marston HD, Fauci AS. Coronavirus Infections-More Than Just the Common Cold. JAMA. 2020.
4. Zhou F, Yu T, Du R, Fan G, Liu Y, Liu Z, et al. Clinical course and risk factors for mortality of adult inpatients with COVID-19 in Wuhan, China: a retrospective cohort study. Lancet. 2020.
5. Butler CC, van der Velden AW, Bongard E, Saville BR, Holmes J, Coenen S, et al. Oseltamivir plus usual care versus usual care for influenza-like illness in primary care: an open-label, pragmatic, randomised controlled trial. The Lancet. 2020;395(10217):42-52.
6. Butler CC, Coenen S, Saville BR, Cook J, van der Velden A, Homes J, et al. A trial like ALIC(4)E: why design a platform, response-adaptive, open, randomised controlled trial of antivirals for influenza-like illness? ERJ Open Res. 2018;4(2).
7. Butler CC, Connor JT, Lewis RJ, Broglio K, Saville BR, Cook J, et al. Answering patient-centred questions efficiently: Response-adaptive platform trials in primary care. British Journal of General Practice. 2018;68(671):294-5.
8. Remuzzi A, Remuzzi G. COVID-19 and Italy: what next? Lancet. 2020.
9. Neil M Ferguson DL, Gemma Nedjati-Gilani, Natsuko Imai, Kylie Ainslie, Marc Baguelin, Sangeeta Bhatia, Adhiratha Boonyasiri, Zulma Cucunubá, Gina Cuomo-Dannenburg, Amy Dighe, Ilaria Dorigatti, Han Fu, Katy Gaythorpe, Will Green, Arran Hamlet, Wes Hinsley, Lucy C Okell, Sabine van Elsland, Hayley Thompson, Robert Verity, Erik Volz, Haowei Wang, Yuanrong Wang, Patrick GT Walker, Caroline Walters, Peter Winskill, Charles Whittaker, Christl A Donnelly, Steven Riley, Azra C Ghani. Impact of non-pharmaceutical interventions (NPIs) to reduce COVID-19 mortality and healthcare demand 2020 [Available from: https://www.imperial.ac.uk/media/imperial-college/medicine/sph/ide/gida-fellowships/Imperial-College-COVID19-NPI-modelling-16-03-2020.pdf.
10. Kucharski AJ, Russell TW, Diamond C, Liu Y, Edmunds J, Funk S, et al. Early dynamics of transmission and control of COVID-19: a mathematical modelling study. Lancet Infect Dis. 2020.
11. Thorpe KE, Zwarenstein M, Oxman AD, Treweek S, Furberg CD, Altman DG, et al. A pragmatic-explanatory continuum indicator summary (PRECIS): a tool to help trial designers. J Clin Epidemiol. 2009;62(5):464-75.
12. Anand R, Norrie J, Bradley JM, McAuley DF, Clarke M. Fool's gold? Why blinded trials are not always best. BMJ. 2020;368:l6228.
13. Moustgaard H, Clayton GL, Jones HE, Boutron I, Jorgensen L, Laursen DRT, et al. Impact of blinding on estimated treatment effects in randomised clinical trials: meta-epidemiological study. BMJ. 2020;368:l6802.
14. Topp CW, Ostergaard SD, Sondergaard S, Bech P. The WHO-5 Well-Being Index: a systematic review of the literature. Psychother Psychosom. 2015;84(3):167-76.
15. Wang M, Cao R, Zhang L, Yang X, Liu J, Xu M, et al. Remdesivir and chloroquine effectively inhibit the recently emerged novel coronavirus (2019-nCoV) in vitro. Cell Res. 2020;30(3):269-71.
16. Devaux CA, Rolain JM, Colson P, Raoult D. New insights on the antiviral effects of chloroquine against coronavirus: what to expect for COVID-19? Int J Antimicrob Agents. 2020:105938.
17. Colson P, Rolain JM, Lagier JC, Brouqui P, Raoult D. Chloroquine and hydroxychloroquine as available weapons to fight COVID-19. Int J Antimicrob Agents. 2020:105932.
18. Gao J, Tian Z, Yang X. Breakthrough: Chloroquine phosphate has shown apparent efficacy in treatment of COVID-19 associated pneumonia in clinical studies. Biosci Trends. 2020.
19. Colson P, Rolain JM, Raoult D. Chloroquine for the 2019 novel coronavirus SARS-CoV-2. Int J Antimicrob Agents. 2020;55(3):105923.
20. Mackenzie AH. Dose refinements in long-term therapy of rheumatoid arthritis with antimalarials. Am J Med. 1983;75(1A):40-5.

© Copyright: The University of Oxford and Oxford University Hospitals NHS Foundation Trust 2019

21. Yao X, Ye F, Zhang M, Cui C, Huang B, Niu P, et al. In Vitro Antiviral Activity and Projection of Optimized Dosing Design of Hydroxychloroquine for the Treatment of Severe Acute Respiratory Syndrome Coronavirus 2 (SARS-CoV-2). Clin Infect Dis. 2020.
22. Mahevas M, Tran VT, Roumier M, Chabrol A, Paule R, Guillaud C, et al. Clinical efficacy of hydroxychloroquine in patients with covid-19 pneumonia who require oxygen: observational comparative study using routine care data. BMJ. 2020;369:m1844.
23. Tang W, Cao Z, Han M, Wang Z, Chen J, Sun W, et al. Hydroxychloroquine in patients with mainly mild to moderate coronavirus disease 2019: open label, randomised controlled trial. BMJ. 2020;369:m1849.
24. Mehra MR, Desai SS, Ruschitzka F, Patel AN. Hydroxychloroquine or chloroquine with or without a macrolide for treatment of COVID-19: a multinational registry analysis. Lancet. 2020.
25. Geleris J, Sun Y, Platt J, Zucker J, Baldwin M, Hripcsak G, et al. Observational Study of Hydroxychloroquine in Hospitalized Patients with Covid-19. N Engl J Med. 2020.
26. Boulware DR, Pullen MF, Bangdiwala AS, Pastick KA, Lofgren SM, Okafor EC, et al. A Randomized Trial of Hydroxychloroquine as Postexposure Prophylaxis for Covid-19. N Engl J Med. 2020.
27. Chen J, Liu D, Liu L, Liu P, Xu Q, Xia L, et al. [A pilot study of hydroxychloroquine in treatment of patients with moderate COVID-19]. Zhejiang Da Xue Xue Bao Yi Xue Ban. 2020;49(2):215-9.
28. Gautret P, Lagier JC, Parola P, Hoang VT, Meddeb L, Mailhe M, et al. Hydroxychloroquine and azithromycin as a treatment of COVID-19: results of an open-label non-randomized clinical trial. Int J Antimicrob Agents. 2020:105949.
29. Retallack H, Di Lullo E, Arias C, Knopp KA, Laurie MT, Sandoval-Espinosa C, et al. Zika virus cell tropism in the developing human brain and inhibition by azithromycin. Proc Natl Acad Sci U S A. 2016;113(50):14408-13.
30. E Bosseboeuf MA, T Nhan, JJ de Pina, JM Rolain, D Raoult, et al. **Azithromycin inhibits the replication of Zika virus.** J Antivirals Antiretrovirals. 2018;10:6-11.
31. Madrid PB, Panchal RG, Warren TK, Shurtleff AC, Endsley AN, Green CE, et al. Evaluation of Ebola Virus Inhibitors for Drug Repurposing. ACS Infect Dis. 2015;1(7):317-26.
32. Bacharier LB, Guilbert TW, Mauger DT, Boehmer S, Beigelman A, Fitzpatrick AM, et al. Early Administration of Azithromycin and Prevention of Severe Lower Respiratory Tract Illnesses in Preschool Children With a History of Such Illnesses: A Randomized Clinical Trial. JAMA. 2015;314(19):2034-44.
33. Villarino AV, Kanno Y, O'Shea JJ. Mechanisms and consequences of Jak-STAT signaling in the immune system. Nat Immunol. 2017;18(4):374-84.
34. Hodge S, Hodge G, Holmes M, Jersmann H, Reynolds PN. Increased CD8 T-cell granzyme B in COPD is suppressed by treatment with low-dose azithromycin. Respirology. 2015;20(1):95-100.
35. Hodge S, Hodge G, Jersmann H, Matthews G, Ahern J, Holmes M, et al. Azithromycin improves macrophage phagocytic function and expression of mannose receptor in chronic obstructive pulmonary disease. Am J Respir Crit Care Med. 2008;178(2):139-48.
36. Hodge S, Reynolds PN. Low-dose azithromycin improves phagocytosis of bacteria by both alveolar and monocyte-derived macrophages in chronic obstructive pulmonary disease subjects. Respirology. 2012;17(5):802-7.
37. Baines KJ, Wright TK, Gibson PG, Powell H, Hansbro PM, Simpson JL. Azithromycin treatment modifies airway and blood gene expression networks in neutrophilic COPD. ERJ Open Res. 2018;4(4).
38. Mandell LA, Wunderink RG, Anzueto A, Bartlett JG, Campbell GD, Dean NC, et al. Infectious Diseases Society of America/American Thoracic Society consensus guidelines on the management of community-acquired pneumonia in adults. Clin Infect Dis. 2007;44 Suppl 2:S27-72.
39. Kawai S, Ochi M, Nakagawa T, Goto H. Antimicrobial therapy in community-acquired pneumonia among emergency patients in a university hospital in Japan. J Infect Chemother. 2004;10(6):352-8.
40. Gleason PP, Meehan TP, Fine JM, Galusha DH, Fine MJ. Associations between initial antimicrobial therapy and medical outcomes for hospitalized elderly patients with pneumonia. Arch Intern Med. 1999;159(21):2562-72.
41. Wu C, Liu Y, Yang Y, Zhang P, Zhong W, Wang Y, et al. Analysis of therapeutic targets for SARS-CoV-2 and discovery of potential drugs by computational methods. Acta Pharm Sin B. 2020.

© Copyright: The University of Oxford and Oxford University Hospitals NHS Foundation Trust 2019

42. Kim HS, Luo L, Pflugfelder SC, Li DQ. Doxycycline inhibits TGF-beta1-induced MMP-9 via Smad and MAPK pathways in human corneal epithelial cells. Invest Ophthalmol Vis Sci. 2005;46(3):840-8.
43. Clemens DL, Duryee MJ, Sarmiento C, Chiou A, McGowan JD, Hunter CD, et al. Novel Antioxidant Properties of Doxycycline. Int J Mol Sci. 2018;19(12).
44. Hsu AT, Barrett CD, DeBusk GM, Ellson CD, Gautam S, Talmor DS, et al. Kinetics and Role of Plasma Matrix Metalloproteinase-9 Expression in Acute Lung Injury and the Acute Respiratory Distress Syndrome. Shock. 2015;44(2):128-36.
45. NICE. COVID-19 rapid guideline: managing suspected or confirmed pneumonia in adults in the community. NICE; 2020.
46. The RECOVERY Collaborative Group. Dexamethasone in Hospitalized Patients with Covid-19 Preliminary Report. *New England Journal of Medicine* 2020 doi: 10.1056/NEJMoa2021436
47. The WHO Rapid Evidence Appraisal for COVID-19 Therapies Working Group. Association Between. Administration of Systemic Corticosteroids and Mortality Among Critically Ill Patients With COVID-19: A Meta-analysis. *JAMA* 2020 doi: 10.1001/jama.2020.17023

© Copyright: The University of Oxford and Oxford University Hospitals NHS Foundation Trust 2019

© Copyright: The University of Oxford and Oxford University Hospitals NHS Foundation Trust 2019

22 APPENDIX A: SCHEDULE OF PROCEDURES

**Procedures**	**Participant contacts**							
	**Visit timing Day 0**	**Day 0**	**Day 0**	**Day 0**	**Daily Day 1-28 incl**	**Day 28-12 months (monthly contact)**	**Day 29-12mths**	**Up to 10 years**
	**Screening completed by participant online/phone**	**Eligibility completed by participant online/phone**	**Baseline completed by participant online/phone**	**Eligibility completed by Clinician online/phone**	**Symptom Diaries completed by participant online/phone**	**Contacted by study team if consent provided**	**Retrospective data collection by study team**	**By data extraction from clinical records**
Informed consent	X	X	X	X	X			
Demographics	X	X	X				X	
Medical history	X	X	X	X			X	
Swab as part of the RCGP RSC/PHE national surveillance programme	When available, preferably by self-swabbing at study entry							
Concomitant medications		X					X	
Eligibility assessment	X	X						

© Copyright: The University of Oxford and Oxford University Hospitals NHS Foundation Trust 2019

Randomisation				X				
Dispensing of trial drugs				X	X			
Questionnaire					X	X		
WHO 5 Well Being Index	X				Day 14 and day 28	X		
Telephone interview (for subset of patient participants)					X			
Compliance					X			
Adverse event assessments					X*		X	
Optional SARS-CoV-2 blood test as part of the RCGP RSC/PHE national surveillance programme							X	
Evidence of sequalae and health care utilisation						X		X

© Copyright: The University of Oxford and Oxford University Hospitals NHS Foundation Trust 2019

* Patient reported AEs will not be assessed by a clinician. The only exception is AEs collected from the hydroxychloroquine group. Participants in this group, will receive a telephone call on day 7 from the trial team to collect any information about cardiovascular Adverse Events (please see hydroxychloroquine appendix). Such events will be assessed by a clinician.

© Copyright: The University of Oxford and Oxford University Hospitals NHS Foundation Trust 2019

23 APPENDIX B: AMENDMENT HISTORY

Amendment No.	Protocol Version No.	Date issued	Author(s) of changes	Details of Changes made
1 (SA1)	1.1		Emma Ogburn; Chris Butler; Gail Hayward	Inclusion criteria: change 'known heart disease' to 'Known heart disease and/or hypertension'; Exclusion criteria: exclude patients taking the following drugs: penicillamine, amiodarone, ciclosporin, chloroquine.<br>Update section 9.6 to include vision changes and lowering of blood sugar.<br>Update change in Funder and update Investigator list to reflect UKRI funder bid.
2 (SA2)	2.0		Emma Ogburn; Chris Butler; Gail Hayward, Hannah Swayze	Inclusion of TSC; central facility to distribute patient packs; addition of third arm; update of secondary outcomes to include WHO wellbeing questions; qualitative sub study; sign posting to other RCGP RSC study; eligibility confirmation by research nurse.
3 (SA3)	2.1		Hannah Swayze; Chris Butler; Emma Ogburn; Gail Hayward	Trial rationale; secondary outcomes to include blood test; 14 days of covid-19 symptoms; call to participant at day 2; poster
4 (SA4)	*2.1*		*No changes to the protocol*	
5 (SA5)	3.0		Hannah Swayze; Chris Butler; Emma Ogburn; Gail Hayward	Updated Azithromycin information; broadening of inclusion criteria; first interim analysis; primary analysis details; care home materials; administrative and typographical updates; study partner letter; recruitment via social media, care homes and pharmacies; GPs prescribe trial medication; eligibility to at least one intervention arm as well as the

© Copyright: The University of Oxford and Oxford University Hospitals NHS Foundation Trust 2019

				Usual Care arm; ICF may be sent to participants.
6 (SA6)	4.0		Chris Butler; Emma Ogburn; Gail Hayward; Ben Saville; Ly-Mee Yu; Hannah Swayze	Updating inclusion criteria; updating the rationale and evidence for safety of hydroxychloroquine; inclusion of a new arm, doxycycline; AE reporting for hydroxychloroquine arm; typographical clarifications.
7 (NS1)	4.0		*No changes to the protocol*	
8 (SA7)	5.0		Chris Butler; Emma Ogburn; Ben Saville; Ly-Mee Yu; Hannah Swayze	Including a second primary outcome, time to recovery, change to sample size estimation, new eligibility criteria: obesity, formatting changes, blood test process.
9 (SA8)	5.0		*No changes to the protocol*	
10 (SA9)	5.0		*No changes to the protocol*	
11 (NS2)	5.0		*No changes to the protocol*	
12 (SA10)	6.0		Chris Butler; Emma Ogburn; Hannah Swayze	Addition of inhaled corticosteroid treatment arm, enrolment to additional trials, long-term follow-up, access to NHS Digital Pillar 2 test data, removal of investigators, additional trial contact with participants for up to 12 months, changes to objectives/outcomes/ time-points, removal of sampling from study
13 (NS3)	6.1		Sharon Tonner	Removal of patient already taking a treatment arm medication as an exclusion
14 (NS4)	6.1		*No changes to the protocol*	
15 (SA11)	6.2		Sharon Tonner, Hannah Swayze	Inclusion of patients who lack capacity to consent, discontinuation of azithromycin arm

Lists details of all protocol amendments whenever a new version of the protocol is produced.

© Copyright: The University of Oxford and Oxford University Hospitals NHS Foundation Trust 2019

Protocol amendments must be submitted to the Sponsor for approval prior to submission to the REC committee, HRA (where required) or MHRA.

© Copyright: The University of Oxford and Oxford University Hospitals NHS Foundation Trust 2019

24 APPENDIX C: USUAL CARE ARM

1. Background and rationale

COVID-19 disproportionately affects people over 50 years old with comorbidities and those over 65 years old. The disease causes considerable morbidity and mortality in this population group in particular, and is having a devastating effect on people's health, and society in the UK and internationally.(1-3, 9) So far, there are no specific treatments for COVID-19 that have been proven in rigorous clinical trials to be effective and that can be used in the community. Clinicians managing possible COVID-19 in the community will make clinical judgements about best treatment based on the clinical situation, but care is usually supportive to begin with, unless patients deteriorate and require hospital admission https://www.nice.org.uk/guidance/ng163). The National Institute for Health and Care Excellence does not recommend the immediate use of antibiotics unless there are signs of pneumonia (https://www.nice.org.uk/guidance/ng163).

This Usual Care arm will follow current NHS care provision, and provides a control against which the effect of new interventions that are added to usual care can be assessed. If a new trial intervention plus usual care is found to be superior to usual care alone, then the usual care alone arm will be dropped, and the intervention that is found to be most effective will become the standard of care within the trial.

2. Changes to outcome measures

None

3. Detail of intervention

Participants randomised to the usual care arm will receive usual clinical care as per NHS care delivery practice.

a. Investigational Medicinal Product (IMP) description

Not applicable

b. Storage of IMP

Not applicable

4. Safety reporting

Mechanisms for safety reporting are outlined in the trial protocol.

© Copyright: The University of Oxford and Oxford University Hospitals NHS Foundation Trust 2019

25 APPENDIX D: USUAL CARE PLUS HYDROXYCHLOROQUINE ARM (DISCONTINUED)

1. Background and rationale

a. Evidence for potential Hydroxychloroquine benefits in COVID-19

A candidate intervention for COVID-19, a drug called hydroxychloroquine, has become available following early evaluation in some studies in China.(15, 16) Hydroxychloroquine is a hydroxylated version of the drug chloroquine.(16, 17) Both agents are commonly in use as anti-malarials, and are used in a variety of auto-immune diseases. They have received significant recent interest as potential modifiers of disease activity in COVID-19. (16, 18, 19) Hydroxychloroquine is already available within the NHS on prescription for other indications, and has a generally benign safety profile.(20) Chloroquine is available to buy in the UK over the counter in some formulations and is used as antimalarial prophylaxis and treatment.

Chloroquine is known to block virus infection by increasing endosomal pH required for virus/cell fusion, as well as interfering with the glycosylation of cellular receptors of SARS-CoV.(5) Besides its antiviral activity, chloroquine has an immune-modulating activity, which may synergistically enhance its antiviral effect *in vivo*.(17) Chloroquine is widely distributed in the whole body, including lungs, after oral administration.(16) The EC_{90} value of chloroquine against the 2019-nCoV in Vero E6 cells was 6.90 µM in one study (15) which can be clinically achievable as demonstrated in the plasma of rheumatoid arthritis patients who received 500 mg administration.(20)

Hydroxychloroquine has been found to be effective against intracellular micro-organisms including malaria and intracellular bacteria *Coxiella burnetii* and *Tropheryma Whipplei*.(17) Both chloroquine and hydroxychloroquine have been shown to have *in vitro* antiviral activity against SARS coronavirus in a number of studies.(17) Most recently activity against SARSCOV2 was shown to be greater for hydroxychloroquine than chloroquine (21).

Key publications that have relevance to the safety and rationale for use of hydroxychloroquine in the PRINCIPLE Trial:

1. *The Mahévas study* was an observational study that assessed whether hydroxychloroquine reduced the need for transfer to ICU in patients already sick enough to be hospitalised.(22) It focussed on sicker patients with hypoxic pneumonia, some requiring ITU care. It did not find a difference in transfers to ICU. So the question and population in the Mahevas study are very different compared to PRINCIPLE. Most importantly, unlike PRINCIPLE, the Mahevas study is ***not a randomised clinical tri***al. Numbers were relatively small (n=181), and it is at high risk of bias due to the observational design.

Regarding safety, those receiving hydroxychloroquine were prescribed 600mg per day, whereas the dose in the PRINCIPLE trial is 400mg per day; 18% of those who received hydroxychloroquine in the Mahévas study were also on azithromycin (which can be arrhythmogenic), and this combination is not possible in PRINCIPLE because of the additive risk. Moreover, PRINCIPLE excludes several other drug combinations that could be arrhythmogenic. In the Mahevas study, eight patients (10%) who were taking hydroxychloroquine experienced electrocardiographic changes that required discontinuation of hydroxychloroquine. Critically, those in the control

© Copyright: The University of Oxford and Oxford University Hospitals NHS Foundation Trust 2019

group did not have ECGs done, so we don't know if there was indeed a difference between groups, and we cannot therefore attribute the ECG changes to hydroxychloroquine. COVID-19 itself, or drug interactions, may well have been underlying reasons. The authors state, "Although hydroxychloroquine is considered safe in the context of systemic lupus erythematosus, these adverse events might be explained by the use of high dose hydroxychloroquine in patients older than 75 years with renal impairment and frequent drug interactions. We cannot rule out the possibility that these cardiac effects attributed to hydroxychloroquine were caused by COVID-19, especially given electrocardiograms were unavailable during follow-up in the control group."

2. *The Tang study* was a hospital-based, randomised study and included 150 patients; randomisation was done using sealed envelopes.(23) The trial found no difference in the proportion of patients with two sequential negative swab results.

Regarding safety,75 participants received hydroxychloroquine 1200 mg daily for 3 days and then 800 mg for either 2 or 3 weeks. Again, the dose used in this study was much higher that the dose being used in PRINCIPLE (initially three times, and subsequently twice as high as PRINCIPLE). However, 63% and 64% of patients in the hydroxychloroquine and control groups respectively also received other antiviral agents. In PRINCIPLE, we are not evaluating the combination of antiviral agents and hydroxychloroquine. Importantly, this study did not find evidence of cardiac arrhythmias associated with hydroxychloroquine use. The authors sate, "Events of cardiac arrhythmia, such as prolonged QT interval were not observed in our trial, possibly because of the relatively mild to moderate disease of patients investigated or the short term period of follow-up."

3. *The Mehra study* published in the Lancet on 22.05.2020 reported an association between hydroxychloroquine use and cardiac events and mortality amongst patients hospitalised with COVID-19.(24) The *observational* study design is inherently susceptible to bias, the study data integrity has been queried given the homogeneity of the baseline characteristics, the adequacy of the adjustment for confounders cannot be assessed from the published methods, and the registries used are in a different patient population compared to PRINCIPLE. Patients were much sicker and more advanced in the illness than in PRINCIPLE. The authors themselves state that "*Randomised clinical trials will be required before any conclusion can be reached regarding benefit or harm of these agents (hydroxychloroquine and chloroquine) in COVID-19 patients*." The authors also state "These data do not apply to the use of any treatment regimen used in the ambulatory, out-of-hospital setting." This study has proved hugely controversial on social media, with a number of methodological and data integrity concerns already raised, for example:

1. There were inadequate adjustments for known and measured confounders (disease severity, temporal effects, site effects, dose used).
2. The authors have not adhered to standard practices in the machine learning and statistics community. They have not released their code or data. There is no data/code sharing and availability statement in the paper. The Lancet was among the many signatories on the Wellcome statement on data sharing for COVID 19 studies.
3. There was no ethics review.
4. There was no mention of the countries or hospitals that contributed to the data source, no acknowledgments to their contributions. A request to the authors for information on the contributing centres was denied.
5. Data from Australia are not compatible with government reports (too many cases for

© Copyright: The University of Oxford and Oxford University Hospitals NHS Foundation Trust 2019

just five hospitals, more in-hospital deaths than had occurred in the entire country during the study period). Surgisphere (the data company) have since claimed this was an error of classification.

6. Data from Africa indicate over 40% of all COVID-19 cases and deaths in the continent occurred in *Surgisphere*-associated hospitals which had sophisticated electronic patient data recording, and patient monitoring able to detect and record "non-sustained [at least 6 secs] or sustained ventricular tachycardia or ventricular fibrillation". This seems unlikely.
7. Unusually small reported variances in baseline variables, interventions and outcomes between continents
8. Mean daily doses of hydroxychloroquine that are 100 mg higher than FDA recommendations, whilst 66% of the data are from North American hospitals.
9. Implausible ratios of chloroquine to hydroxychloroquine use in some continents.
10. The tight 95% confidence intervals reported for the hazard ratios are unlikely. For instance, for the Australian data this would need about double the numbers of recorded deaths that were reported in the paper.

This paper has now been retracted, and the data cannot be verified.

4. ***The Geleris study*** was an observational study of 1,376 consecutive COVID-19 patients at a New York hospital to determine whether hydroxychloroquine use was associated with intubation or death, as a primary composite outcome.(25) 811 (58.9%) of these patients received hydroxychloroquine. The authors excluded patients who were intubated, died, or who were transferred to another facility within 24 hours after presentation to the emergency department from the analyses. A propensity score matching model (C-statistic of 0.81) was used to ensure that groups were similar at baseline.

Regarding safety, multivariable adjusted analyses with inverse probability weighting revealed no significant association between treatment with hydroxychloroquine and intubation or death (HR 1.04 (95% CI 0.82 – 1.32)). Whilst the patient population in this study is different to that of PRINCIPLE, it is interesting that the findings contrast with those of a recent Lancet study published by Mehra *et al*. One possible reason for the difference is that patients receiving interventions like hydroxychloroquine in the study by Mehra *et al* were sicker than those in the study's control group. This may have arisen through use of crude measures to account for baseline disease severity (qSOFA score and SpO2 < 94%) in their propensity score matching model, and may also explain the big differences seen in patients requiring mechanical ventilation between controls (7.7%) and those in intervention groups (20-21.6%).

5. ***Boulware*** and colleagues conducted a Covid-19 postexposure prophylaxis, placebo controlled randomised trial of hydroxychloroquine in 821 asymptomatic patients; 11.8% of those taking hydroxychloroquine vs 14.3 of those taking placebo experienced a new illness compatible with COVID-19 (absolute difference -2.4%) but this difference was not statistically significant, indicating no evidence of benefit from the hydroxychloroquine. (26)

Regarding safety, while side effects were more common with hydroxychloroquine than with placebo (40.1% vs. 16.8%), no serious adverse reactions were reported.

© Copyright: The University of Oxford and Oxford University Hospitals NHS Foundation Trust 2019

Earlier studies of hydroxychloroquine for COVID-19

1. *Chen* and colleagues conducted a *randomised* controlled trial to test the effectiveness of hydroxychloroquine in 30 adult patients who tested positive for COVID-19 in China.(27) Patients in the treatment group received 400mg of hydroxychloroquine for 5 days, while the control group received usual care. The result of a nasopharyngeal swab on Day 7 was used as the primary outcome. The intention- to- treat analysis revealed that the treatment group did not differ from the control group in the number of patients testing negative for COVID-19 on Day 7 (13 versus 14 patients), nor the duration of illness (all P>0.05).

***Regarding safety*,** the authors report three adverse events in the control group (one patient with abnormal liver function and anaemia, and one patient with abnormal renal function), and four adverse events in the treatment group (two patients with diarrhoea, one with lethargy, and one patient with abnormal liver function tests), which the authors argue were not linked to treatment with HCQ. One patient in the treatment group deteriorated significantly and thus HCQ was stopped on Day 4 of the treatment. This study was under-powered according to their own calculations.

2. Gautret and colleagues presented the results of an open- label, non-randomised trial with 36 patients diagnosed with COVID-19 in French hospitals.(28) Six participants were asymptomatic, 22 had upper respiratory tract infection symptoms, and eight had lower respiratory tract infection symptoms. The twenty patients in the treatment group received HCQ 200mg three times a day for 10 days. Patients declining to take part in the study and not meeting the inclusion criteria were assigned to the control group and received usual care. Six of the patients in the treatment group additionally received azithromycin to prevent bacterial superinfection. The primary outcome was SARS- CoV-2 carriage at Day 6 on nasopharyngeal swabs. Patients treated with hydroxychloroquine were significantly more likely to test negative for SARS- CoV-2 on Day 6 compared with controls (70% versus 12.5% virologically cured, p<0.001). All patients treated with hydroxychloroquine and azithromycin tested negative on Day 6.

***Regarding safety*,** the authors did not report any safety data, stating that this would follow in a subsequent publication. Aside from a lack of adverse event reporting, there are many problems with the study methodology including the non-randomized design, under-powered sample size, lack of intention-to-treat analysis, and absence of medium to long-term follow-up data.

3. Chen and colleagues conducted a *randomised* clinical trial of adult patients admitted to hospital with confirmed COVID-19.(7) Sixty two patients were randomly assigned to usual care (n=31) or hydroxychloroquine (200 mg BD) for five days in addition to usual care (n=31). The authors report that there were ‘significant differences’ in time to clinical recovery (TTCR) between the two groups, with TTCR defined as the return of body temperature and cough relief, maintained for more than 72 hours. They also report that all four patients who ‘progressed to severe disease’ were in the control group. The reporting of empirical data by the authors is limited and unclear. They did not include a power calculation, but presumably this study was under-powered to detect differences between groups. No medium to long-term follow-up data is presented.

Regarding safety, the authors report that two mild adverse events occurred (a rash and a headache), both of which were in patients receiving hydroxychloroquine. No patients receiving usual care experienced adverse events.

© Copyright: The University of Oxford and Oxford University Hospitals NHS Foundation Trust 2019

In summary
The large scale hospital based Recovery trial has recently announced that they found no benefit from hydroxychloroquine (as yet unpublished). No safety concerns have been reported by the Principle Trial. A post exposure prophylaxis study found no benefit from hydroxychloroquine, but also found no safety concerns. These studies address a different research question and focus on different patient populations in comparison to the Principle Trial. Evidence about early treatment of COPVID-19 in the community is urgently needed: the potential application of the findings of the PRINCIPLE Trial of community treatment is considerable, and the 'reach' of the study is now nation-wide. Our study population are patients in the community and our trial question is about early treatment. Outcome data from studies with sicker hospitalised patients may not apply to our study population

A key, controversial observational study (Mehra et al) reported that those taking hydroxychloroquine had worse outcomes and suffered more cardiac events than those not taking hydroxychloroquine. However, major doubts have been expressed about the data integrity of this study and insufficient detail in the paper to judge the adequacy of the methods employed to adjust for the inevitable confounders in an observational study. Hydroxychloroquine is not a licensed drug for treating COVID-19. Patients doing well are therefore less likely to be prescribed this drug. When a patient is causing their clinical team more concern or their condition is deteriorating, the chances of them being prescribed hydroxychloroquine will be greater. Adjustment for potential confounders has been inadequate in the observational studies. Critically, these studies cannot adjust for the clinician's sense of how the patient is faring over time. The Mehra study has been retracted and can't be relied upon.

The deficiencies and differences in all of these studies highlight the need for well-conducted, adequately powered randomised clinical trials, to provide definitive evidence of the safety and effectiveness of hydroxychloroquine for the early community treatment COVID-19 illness. PRINCIPLE will assess whether hydroxychloroquine is safe and effective if given earlier in the course of illness and in patients with milder symptoms not requiring hospital admission.

2. Eligibility criteria specifically related to hydroxychloroquine

Inclusion criteria:

Exclusion criteria:

- Pregnancy;
- Breastfeeding;
- Known severe hepatic impairment;
- Known severe renal impairment;
- Known porphyria;
- Type 1 diabetes or insulin dependent Type 2 Diabetes mellitus ;
- Known G6PD deficiency;
- Known myasthenia gravis;
- Known severe psoriasis;
- Known severe neurological disorders (especially those with a history of epilepsy—may lower seizure threshold)
- Previous adverse reaction to, or currently taking, hydroxychloroquine or chloroquine

© Copyright: The University of Oxford and Oxford University Hospitals NHS Foundation Trust 2019

Patients currently taking the following drugs: penicillamine, amiodarone, ciclosporin, digoxin: the following antimicrobials; azithromycin, clarithromycin, erythromycin, ciprofloxacin, levofloxacin, moxifloxacin, ketoconazole, itraconazole, or mefloquine: the following antidepressants; amitriptyline, citalopram, desipramine, escitalopram, imipramine, doxepin, fluoxetine, wellbutrin, venlafaxine; the following antipsychotics or mood stabilizers; haloperidol, droperidol, lithium, quetiapine, thioridazine, ziprasidone: methadone: sumatriptan, zolmitriptan

- Known congenital or documented QT prolongation
- Known retinal disease

3. Outcome measures related to hydroxychloroquine

There are no outcome measures related specifically to this usual care plus hydroxychloroquine arm

4. Detail of intervention

Participants randomised to the usual care plus hydroxychloroquine arm will receive usual clinical care as per NHS guidelines, plus a course of oral hydroxychloroquine 200mg twice daily for seven days.

a. Investigational Medicinal Product (IMP) description

Hydroxychloroquine sulphate 200 milligram (mg) tablets. The tablets are for oral administration. One tablet (200mg) hydroxychloroquine to be taken twice daily for 7 days by mouth (14 tablets in total).
Special instructions: Each dose should be taken with a meal or glass of milk. Antacids may reduce absorption of hydroxychloroquine so it is advised that a 4-hour interval be observed between taking hydroxychloroquine and an antacid.
This is the standard therapeutic dose for its normal indication in adults which is for the treatment of rheumatoid arthritis, discoid and systemic lupus erythematosus, and dermatological conditions caused or aggravated by sunlight.

The Marketing Authorisation holder is Zentiva Pharma UK Limited Guildford Surrey GU1 4YS United Kingdom. Marketing authorisation number is PL 17780/0748.

b. Storage of IMP

: Stored at room temperature in locked cupboards in restricted access rooms in the Nuffield Department of Primary Care Health Sciences; in locked cupboards in restricted access rooms in GP Practices; in Pharmacies.

For hydroxychloroquine, GP practices can order a supply of trial medication from Public Health England using the existing ImmForm process. GPs will be provided with an envelope by the trial team which will be labelled appropriately for trial medication, and they will add the patient's details to this label. This pack, containing instructions on using the medication will be provided to the patient or their representative.

© Copyright: The University of Oxford and Oxford University Hospitals NHS Foundation Trust 2019

c. SmPC precautions and concomitant medication

Hydroxychloroquine: Hydroxychloroquine will be used for short-term use (7 days) in this trial. The SmPC and precautions listed below focus on longer term chronic use.

i. Precautions

Hydroxychloroquine might lower blood sugar levels in some people. If participants develop these symptoms, they will be advised in the Patient Information documents to eat something sweet and seek clinical advice if the symptoms continue.

Hydroxychloroquine occasionally causes blurred vision, which typically resolves once the medication is stopped. Participants will be advised via the Participant Information documents that if they develop any problems with vision, they should stop taking the medication immediately, seek clinical advice, and not drive or operate any heavy machinery.

ii. Concomitant medication

Hydroxychloroquine sulfate has been reported to increase plasma digoxin levels. Serum digoxin levels should be closely monitored in participants receiving concomitant treatment.

Hydroxychloroquine sulfate may also be subject to several of the known interactions of chloroquine even though specific reports have not appeared. These include: potentiation of its direct blocking action at the neuromuscular junction by aminoglycoside antibiotics; inhibition of its metabolism by cimetidine which may increase plasma concentration of the antimalarial; antagonism of effect of neostigmine and pyridostigmine; reduction of the antibody response to primary immunisation with intradermal human diploid-cell rabies vaccine.

As with chloroquine, antacids may reduce absorption of hydroxychloroquine so it is advised that a four hour interval be observed between hydroxychloroquine and antacid dosaging.

As hydroxychloroquine may enhance the effects of a hypoglycaemic treatment, a decrease in doses of insulin or antidiabetic drugs may be required.

Halofantrine prolongs the QT interval and should not be administered with other drugs that have the potential to induce cardiac arrhythmias, including hydroxychloroquine. Also, there may be an increased risk of inducing ventricular arrhythmias if hydroxychloroquine is used concomitantly with other arrhythmogenic drugs, such as amiodarone and moxifloxacin.

An increased plasma ciclosporin level was reported when ciclosporin and hydroxychloroquine were co-administered.

Hydroxychloroquine can lower the convulsive threshold. Co-administration of hydroxychloroquine with other antimalarials known to lower the convulsion threshold (e.g. mefloquine) may increase the risk of convulsions. Also, the activity of anti-epileptic drugs might be impaired if co-administered with hydroxychloroquine. In a single-dose interaction study, chloroquine has been reported to reduce the bioavailability of praziquantel. It is not known if there is a similar effect when hydroxychloroquine and praziquantel are co-administered. Per extrapolation, due to the similarities in structure and pharmacokinetic parameters between hydroxychloroquine and chloroquine, a similar effect may be expected for hydroxychloroquine.

© Copyright: The University of Oxford and Oxford University Hospitals NHS Foundation Trust 2019

There is a theoretical risk of inhibition of intra-cellular α-galactosidase activity when hydroxychloroquine is co-administered with agalsidase.

iii. Pregnancy and Breastfeeding

A moderate amount of data on pregnant women (between 300 – 1000 pregnancy outcomes), including prospective studies in long-term use with large exposure, have not observed a significant increased risk of congenital malformations or poor pregnancy outcomes. Hydroxychloroquine crosses the placenta. Only limited non-clinical data are available for hydroxychloroquine, data on chloroquine have shown developmental toxicity at high supratherapeutic doses and a potential risk of genotoxicity in some test systems. Therefore, hydroxychloroquine sulfate should be avoided in pregnancy except when, in the judgement of the physician, the individual potential benefits outweigh the potential hazards. Careful consideration should be given to using hydroxychloroquine during lactation, since it has been shown to be excreted in small amounts in human breast milk, and it is known that infants are extremely sensitive to the toxic effects of 4-aminoquinolines.
Pregnancy and breastfeeding are exclusion criteria for the hydroxychloroquine arm of the PRINCIPLE trial.

5. Safety reporting

Hydroxychloroquine: has a well-documented safety profile and is a commonly used medication in a primary care setting (see above).

Common symptoms of hydroxychloroquine include abdominal pain; appetite decreased; diarrhoea; emotional lability; headache; nausea; skin reactions; vision disorders; and vomiting.
Mechanisms for safety reporting are outlined in the trial protocol.

We will call all participants randomised to hydroxychloroquine on day 7 to ask about cardiovascular AEs. Our team of clinicians will review any AEs relating to cardiovascular symptoms from the day 7 call, and assess whether these may be related to hydroxychloroquine. If AEs are thought to be related and it's deemed necessary by the assessing clinician, the participant's GP will be contacted to arrange a face-to-face visit for further clinical evaluation.

© Copyright: The University of Oxford and Oxford University Hospitals NHS Foundation Trust 2019

26 APPENDIX E: USUAL CARE PLUS AZITHROMYCIN ARM (DISCONTINUED)

1. Background and rationale

a. Evidence for potential Azithromycin benefits in COVID-19

Atypical macrolides, especially Azithromycin, have activities that may be beneficial in the treatment of COVID-19 patients, and especially those in the at-risk or age range of the PRINCIPLE trial.

Firstly, Azithromycin appears to have some anti-viral mechanisms. In COVID-19, Azithromycin appears to inhibit viral replication and therefore reduces shedding. In the small open observational trial of Gautret *et al* the addition of azithromycin to hydroxychloroquine (HCQ) (at 200 tds for 10 days) in 6 of the 14 HCQ subjects of the total 36 COVID-19 patients in the study significantly reduced viral shedding at 3 days to 15% (one subject) versus 70% in the HCQ arm and 95% in the indirect control arm, with no shedding at 6 days in the combination arm versus 50% and 90% respectively.(28) Azithromycin was also used in some Chinese observational and interventional studies.

Azithromycin has also been shown to be active ***in vitro*** against Zika and Ebola viruses,(29-31) and to prevent severe respiratory tract infections when administrated to patients suffering viral infection.(32) Inhibition of viral infections by azithromycin may be linked to its suppressive effect on the production of viral interferon.(33) Longer term administration of low dose azithromycin in COPD has been shown to suppress proinflammatory cytokine production, potentiate macrophage phagocytosis and anti-inflammatory cytokine expression.(34-36) Azithromycin use is also associated with a decrease in the expression of human HLA (human leukocyte antigen) complex molecules in the respiratory tract, including HLA-A, HLA-B, HLA-DPA1, HLA-DRA, HLA-DRB4.(37)

b. Importance of treating CAP or CAP risk in the elderly or immuno-compromised

An important secondary pathway to severe illness and death with COVID-19 may be secondary infection and sepsis in the immune-compromised state, especially secondary community or hospital acquired pneumonia. Older people are more susceptible to pneumonia because of comorbidities, a weakened immune system and are therefore more likely to die.(38) The onset of pneumonia in the elderly can often be rapid, and for severe pneumonia, the prognosis is poor: as many as one in five will die.(38) Severe pneumonia is more prevalent the older you are and in those with more serious underlying diseases.(39) The leading cause of death is respiratory insufficiency. Death has been shown to increase in those not responding to initial antimicrobials, and consequently, the initial selection of the agent is important.

Common causative organisms in the elderly admitted to the hospital with pneumonia include *Haemophilus influenza, Staphylococcus aureus, Streptococcus pneumoniae,* and *Mycoplasma pneumoniae*. In severe pneumonia, *S. aureus, Klebsiella pneumoniae*, and *Pseudomonas aeruginosa* have been identified as common causative organisms. Older patients often have polymicrobial infections, which may be a factor in non-responders. Assessment of 12,945 US

© Copyright: The University of Oxford and Oxford University Hospitals NHS Foundation Trust 2019

Medicare inpatients over 65 with pneumonia found that initial treatment with a second-generation cephalosporin plus macrolide ([HR, 0.71; 95% CI, 0.52-0.96), a non-pseudomonas third-generation cephalosporin plus a macrolide (HR, 0.74; 0.60-0.92), or a fluoroquinolone alone (HR, 0.64; 0.43-0.94) was associated with lower 30-day mortality.(40)

For CAP management NICE guidance currently recommends Amoxycillin 500mg tds combined with Clarithromycin 500mg bd for 5 days or, in penicillin sensitive, Clarithromycin 500mg bd for 5 days or Doxycycline 200mg stat then 100mg daily for the next 4 days. They also recommend starting therapy within 4 hours. The identification of the early stages of pneumonia in older patients can prove challenging since traditional symptoms and signs, including fever, may be lacking.

Azithromycin will have at least as broad a spectrum of action as clarithromycin in terms of bacterial infections and the additional potential anti-viral activity which has not been observed for other macrolides like Clarithromycin. It will also cover atypical organisms.

2 Changes to outcome measures

The addition of this usual care plus azithromycin arm will not require any changes to outcome measures

3 Eligibility criteria specifically related to azithromycin

Inclusion criteria: No changes

Exclusion criteria:

- Pregnancy
- Breastfeeding
- Known severe hepatic impairment;
- Known severe renal impairment;
- Known myasthenia gravis;
- Previous adverse reaction to, or currently taking, azithromycin or other macrolides or ketolides
- Patients taking the following drugs: hydroxychloroquine or chloroquine, sotalol, amiodarone, ciclosporin, digoxin, bromocriptine, cabergoline, ergotamine, ergometrine, methysergide or any ergot derivatives.
- Already taking antibiotics for an acute condition
- Known congenital or documented QT prolongation
- Known allergy to soya or peanut due to the risk of hypersensitivity reactions

4 Detail of intervention

Participants randomised to the usual care plus azithromycin arm will receive usual clinical care as per NHS guidelines, plus a course of oral azithromycin 500mg daily for three days. We will use the IMP distribution methods described in the protocol to deliver IMP to participants.

© Copyright: The University of Oxford and Oxford University Hospitals NHS Foundation Trust 2019

a. Investigational Medicinal Product (IMP) description

Azithromycin 250mg capsules. Participants in this arm will take 500 mg (two capsules) once daily for 3 days. The capsules are for oral administration.

Special instructions:
Azithromycin must be taken at least 1 hour before or 2 hours after antacids as this affects overall bioavailability. Azithromycin must be taken at least 1 hour before or 2 hours after food.

The marketing authorisation holder is: Teva UK Limited, Brampton Road, Hampden Park, Eastbourne, East Sussex, BN22 9AG, UK.
Marketing authorisation number: PL 00289/1570

b. Storage of IMP

Azithromycin: Stored at room temperature in locked cupboards in restricted access rooms in the Nuffield Department of Primary Care Health Sciences; in locked cupboards in restricted access rooms in GP Practices; in Pharmacies.

c. SmPC precautions and concomitant medication

i. Precautions

Azithromycin is a commonly prescribed antibiotic with an established safety profile. The SmPC advises caution using azithromycin in the following conditions:
Elderly people with proarrhythmic conditions due to the risk of developing cardiac arrhythmia and torsades de pointes including patients with congenital or documented QT prolongation; receiving treatment with other active substances known to prolong QT interval such as anti-arrhythmics (e.g. amiodarone and sotalol), cisapride, and fluoroquinolones such as moxifloxacin and levofloxacin; known hypokalaemia and hypomagnesaemia; significant hepatic or renal impairment; patients with neurological or psychiatric disorders; myasthenia gravis. Azithromycin as other with the use of nearly all antibacterial agents, alters the normal flora of the colon leading to overgrowth of Clostridium difficile which can lead to Clostridium difficile associated diarrhoea.

ii. Concomitant medications

Effects of other medicinal products on azithromycin:

Antacids

In a pharmacokinetic study investigating the effects of simultaneous administration of antacids and azithromycin, no effect on overall bioavailability was seen, although the peak serum concentrations were reduced by approximately 25%. In patients receiving both azithromycin and antacids, the medicinal products should not be taken simultaneously. Azithromycin must be taken at least 1 hour before or 2 hours after the antacids.

Co-administration of azithromycin prolonged-release granules for oral suspension with a single 20 ml dose of co-magaldrox (aluminium hydroxide and magnesium hydroxide) did not affect the rate and extent of azithromycin absorption.

Co-administration of a 600 mg single dose of azithromycin and 400 mg efavirenz daily for 7 days did not result in any clinically significant pharmacokinetic interactions.

© Copyright: The University of Oxford and Oxford University Hospitals NHS Foundation Trust 2019

Fluconazole

Co-administration of a single dose of 1200 mg azithromycin did not alter the pharmacokinetics of a single dose of 800 mg fluconazole. Total exposure and half-life of azithromycin were unchanged by the coadministration of fluconazole, however, a clinically insignificant decrease in C_{max} (18%) of azithromycin was observed.

Nelfinavir

Co-administration of azithromycin (1200 mg) and nelfinavir at steady state (750 mg three times daily) resulted in increased azithromycin concentrations. No clinically significant adverse effects were observed and no dose adjustment is required.

Rifabutin

Coadministration of azithromycin and rifabutin did not affect the serum concentrations of either medicinal product.

Neutropenia was observed in subjects receiving concomitant treatment of azithromycin and rifabutin. Although neutropenia has been associated with the use of rifabutin, a causal relationship to combination with azithromycin has not been established.

Terfenadine

Pharmacokinetic studies have reported no evidence of an interaction between azithromycin and terfenadine. There have been rare cases reported where the possibility of such an interaction could not be entirely excluded; however, there was no specific evidence that such an interaction had occurred.

Cimetidine

In a pharmacokinetic study investigating the effects of a single dose of cimetidine, given 2 hours before azithromycin, on the pharmacokinetics of azithromycin, no alteration of azithromycin pharmacokinetics was seen.

Effect of azithromycin on other medicinal products:
Ergotamine derivatives

Due to the theoretical possibility of ergotism, the concurrent use of azithromycin with ergot derivatives is not recommended.

Digoxin and colchicine (P-gp substrates)

Concomitant administration of macrolide antibiotics, including azithromycin, with P-glycoprotein substrates such as digoxin and colchicine, has been reported to result in increased serum levels of the P-glycoprotein substrate. Therefore, if azithromycin and P-gp substrates such as digoxin are administered concomitantly, the possibility of elevated serum concentrations of the substrate should be considered.

Coumarin-Type Oral Anticoagulants

In a pharmacokinetic interaction study, azithromycin did not alter the anticoagulant effect of a single 15-mg dose of warfarin administered to healthy volunteers. There have been reports received in the post-marketing period of potentiated anticoagulation subsequent to co-administration of azithromycin and coumarin-type oral anticoagulants. Although a causal relationship has not been established, consideration should be given to the frequency of

© Copyright: The University of Oxford and Oxford University Hospitals NHS Foundation Trust 2019

monitoring prothrombin time when azithromycin is used in patients receiving coumarin-type oral anticoagulants.

Cyclosporin

In a pharmacokinetic study with healthy volunteers that were administered a 500 mg/day oral dose of azithromycin for 3 days and were then administered a single 10 mg/kg oral dose of cyclosporin, the resulting cyclosporin C_{max} and AUC_{0-5} were found to be significantly elevated. Consequently, caution should be exercised before considering concurrent administration of these drugs. If coadministration of these drugs is necessary, cyclosporin levels should be monitored and the dose adjusted accordingly.

Theophylline

There is no evidence of a clinically significant pharmacokinetic interaction when azithromycin and theophylline are co-administered to healthy volunteers. As interactions of other macrolides with theophylline have been reported, alertness to signs that indicate a rise in theophylline levels is advised.

Trimethoprim/sulfamethoxazole

Coadministration of trimethoprim/sulfamethoxazole DS (160 mg/800 mg) for 7 days with azithromycin 1200 mg on Day 7 had no significant effect on peak concentrations total exposure or urinary excretion of either trimethoprim or sulfamethoxazole. Azithromycin serum concentrations were similar to those seen in other studies.

Zidovudine

Single 1000 mg doses and multiple 1200 mg or 600 mg doses of azithromycin had little effect on the plasma pharmacokinetics or urinary excretion of zidovudine or its glucuronide metabolite. However, administration of azithromycin increased the concentrations of phosphorylated zidovudine, the clinically active metabolite, in peripheral blood mononuclear cells. The clinical significance of this finding is unclear, but it may be of benefit to patients.

Azithromycin does not interact significantly with the hepatic cytochrome P450 system. It is not believed to undergo the pharmacokinetic drug interactions as seen with erythromycin and other macrolides. Hepatic cytochrome P450 induction or inactivation via cytochrome-metabolite complex does not occur with azithromycin.

Astemizole, alfentanil

There are no known data on interactions with astemizole or alfentanil. Caution is advised in the co-administration of these medicines with azithromycin because of the known enhancing effect of these medicines when used concurrently with the macrolid antibiotic erythromycin.

Atorvastatin

Coadministration of atorvastatin (10 mg daily) and azithromycin (500 mg daily) did not alter the plasma concentrations of atorvastatin (based on a HMG CoA-reductase inhibition assay).

However, post-marketing cases of rhabdomyolysis in patients receiving azithromycin with statins have been reported.

Carbamazepine

© Copyright: The University of Oxford and Oxford University Hospitals NHS Foundation Trust 2019

In a pharmacokinetic interaction study in healthy volunteers, no significant effect was observed on the plasma levels of carbamazepine or its active metabolite in patients receiving concomitant azithromycin.

Cisapride

Cisapride is metabolized in the liver by the enzyme CYP 3A4. Because macrolides inhibit this enzyme, concomitant administration of cisapride may cause the increase of QT interval prolongation, ventricular arrhythmias and torsades de pointes.

Cetirizine

In healthy volunteers, coadministration of a 5-day regimen of azithromycin with cetirizine 20 mg at steady-state resulted in no pharmacokinetic interaction and no significant changes in the QT interval.

Didanosins (Dideoxyinosine)

Coadministration of 1200 mg/day azithromycin with 400 mg/day didanosine in 6 HIV-positive subjects did not appear to affect the steady-state pharmacokinetics of didanosine as compared with placebo.

Efavirenz

Coadministration of a 600 mg single dose of azithromycin and 400 mg efavirenz daily for 7 days did not result in any clinically significant pharmacokinetic interactions.

Indinavir

Coadministration of a single dose of 1200 mg azithromycin had no statistically significant effect on the pharmacokinetics of indinavir administered as 800 mg three times daily for 5 days.

Methylprednisolone

In a pharmacokinetic interaction study in healthy volunteers, azithromycin had no significant effect on the pharmacokinetics of methylprednisolone.

Midazolam

In healthy volunteers, coadministration of azithromycin 500 mg/day for 3 days did not cause clinically significant changes in the pharmacokinetics and pharmacodynamics of a single 15 mg dose of midazolam.

Sildenafil

In normal healthy male volunteers, there was no evidence of an effect of azithromycin (500 mg daily for 3 days) on the AUC and C_{max} of sildenafil or its major circulating metabolite.

Triazolam

In 14 healthy volunteers, coadministration of azithromycin 500 mg on Day 1 and 250 mg on Day 2 with 0.125 mg triazolam on Day 2 had no significant effect on any of the pharmacokinetic variables for triazolam compared to triazolam and placebo.

© Copyright: The University of Oxford and Oxford University Hospitals NHS Foundation Trust 2019

iii. Fertility, pregnancy and lactation

Pregnancy

There are no adequate data from the use of azithromycin in pregnant women. In reproduction toxicity studies in animals azithromycin was shown to pass the placenta, but no teratogenic effects were observed. The safety of azithromycin has not been confirmed with regard to the use of the active substance during pregnancy. Therefore azithromycin should only be used during pregnancy if the benefit outweighs the risk.

5 Safety reporting

Mechanisms for safety reporting are outlined in the protocol. In brief, we will collect symptoms and side effects of azithromycin from symptom diaries and participant telephone calls.

Common symptoms of azithromycin include diarrhoea, abdominal pain, nausea and flatulence. It may also cause headache, dizziness, insomnia, altered taste, pins and needles, changes in vision or hearing, rash, itching, joint pains or fatigue.

© Copyright: The University of Oxford and Oxford University Hospitals NHS Foundation Trust 2019

26 APPENDIX F: USUAL CARE PLUS DOXYCYCLINE ARM

1. Background and rationale

a. Evidence for potential doxycycline benefits in COVID-19

Doxycycline may be beneficial in the treatment of COVID-19 patients, and especially those in the at-risk or age range of the PRINCIPLE trial.
The rationale for testing doxycycline is based on three reasons:
Firstly, doxycycline may have direct antiviral activity against SARS-CoV-2 based on computer modelling. Analysing all the proteins encoded by SARS-CoV-2 genes and then predicting potential targets by performing target-based virtual ligand screening, doxycycline ranked in the group of compounds with the highest binding affinity to 3CLpro (3-chymotrypsin-like protease). 3CLpro is the main protease in SARS- CoV-2 which is critical in the life-cycle of the virus (41).

Secondly, doxycycline has known anti-inflammatory effects in various human diseases by inhibiting mitogen-activated protein kinase (MAPK) and SMAD pathways (42), as well as potent antioxidant properties(43). Doxycycline reduces the hyperinflammation associated with severe COVID-19 by antagonising metalloproteinases such as MMP9 that are linked with lung injury, including SARS and ARDS(44).

Lastly, from extensive experience in other infectious diseases, doxycycline has broad antimicrobial activity and is efficacious against a broad spectrum of bacteria including atypical bacteria and other pathogens including intracellular plasmodia, chlamydia, rickettsia, and RNA viruses like Dengue fever and chikungunya.

b. Importance of treating CAP or CAP risk in the elderly or immuno-compromised

An important secondary pathway to severe illness and death with COVID-19 may be secondary infection and sepsis in the immune-compromised state, especially secondary community or hospital acquired pneumonia. Older people are more susceptible to pneumonia because of comorbidities, a weakened immune system and are therefore more likely to die.(38) The onset of pneumonia in the elderly can often be rapid, and for severe pneumonia, the prognosis is poor: as many as one in five will die.(38) Severe pneumonia is more prevalent the older you are and in those with more serious underlying diseases.(39) The leading cause of death is respiratory insufficiency. Death has been shown to increase in those not responding to initial antimicrobials, and consequently, the initial selection of the agent is important. Common causative organisms in the elderly admitted to the hospital with pneumonia include *Haemophilus influenza, Staphylococcus aureus, Streptococcus pneumoniae* and less commonly, atypical organisms, such as *Mycoplasma pneumoniae* and *Klebsiella pneumoniae*. All these organisms fall under doxycycline's antimicrobial spectrum.

We are aware that currently NICE, in their COVID-19 rapid guideline, advocates that clinicians offer oral doxycycline for treatment of suspected pneumonia in people who can or wish to be

© Copyright: The University of Oxford and Oxford University Hospitals NHS Foundation Trust 2019

treated in the community if: the likely cause is bacterial or; it is unclear whether the cause is bacterial or viral and symptoms are more concerning or; they are at high risk of complications (older or frail patients, pre-existing comorbidity or have a history of severe illness following previous lung infection).(45) Doxycycline will have at least as broad a spectrum of action as azithromycin in terms of bacterial infections with the potential anti-viral and anti-inflammatory effects.

Doxycycline for acute cough and community acquired pneumonia is recommended in the British National Formulary at a dose of Doxycycline 200mg stat then 100mg daily for the next 4 days. However, its use in COVID-19 is not proven and therefore important to address in this trial. Given the potential anti-inflammatory properties of doxycycline, we will use a slightly extended 7 day course.

2. Changes to outcome measures

The addition of this usual care plus doxycycline arm will not require any changes to outcome measures

3. Eligibility criteria specifically related to doxycycline

Inclusion criteria: No changes
Exclusion criteria:

- Pregnancy
- Breastfeeding
- Myasthenia gravis
- Systemic lupus erythematosus
- Previous adverse reaction to, or currently taking, doxycycline or other tetracyclines
- Sucrose intolerance (i.e. rare hereditary problems of fructose intolerance, glucose galactose malabsorption or sucrose-isomaltase insufficiency)
- Already taking antibiotics for an acute condition
- Patients taking the following drugs: ciclosporin, retinoids (acitretin, alitretinoin, isotretinoin, tretinoin), methotrexate, ergotamine, methoxyflurane, lithium.

4. Detail of intervention

Participants randomised to the usual care plus doxycycline arm will receive usual clinical care as per NHS guidelines, plus a course of oral doxycycline for 7 days. We will use the IMP distribution methods described in the protocol to deliver IMP to participants.

a. Investigational Medicinal Product (IMP) description

Doxycycline 100mg capsules. Participants in this arm will take 200mg on the first day (as a single dose or in divided doses with a twelve hour interval) followed by 100mg a day for 6 days (7 day course in total). The capsules are for oral administration.

© Copyright: The University of Oxford and Oxford University Hospitals NHS Foundation Trust 2019

Special instructions:
Capsules should be swallowed whole with plenty of fluid, while sitting or standing. Capsules should be taken during meals, well before going to bed. Due to the risk of photosensitivity, patients should be advised to avoid exposure to sunlight or sun lamps.

The marketing authorisation holder is:

Accord-UK Ltd (Trading style: Accord), Whiddon Valley, Barnstaple, Devon, EX32 8NS
Marketing authorisation number: PL 0142/0407

b. Storage of IMP

Doxycycline: Stored at room temperature in locked cupboards in restricted access rooms in the Nuffield Department of Primary Care Health Sciences; in locked cupboards in restricted access rooms in GP Practices; in Pharmacies.

c. SmPC precautions and concomitant medication

i. Precautions

Doxycycline is a commonly prescribed antibiotic with an established safety profile. The SmPC states that in elderly patients “doxycycline may be prescribed in the usual dose with no special precautions. No dosage adjustment is necessary in the presence of renal impairment”.

ii. Concomitant medications

Warfarin
There have been reports of prolonged prothrombin time in patients taking warfarin and doxycycline. Tetracyclines depress plasma prothrombin activity and reduced dosage of concomitant anti-coagulants may be necessary

5. Safety reporting

Mechanisms for safety reporting are outlined in the protocol. In brief, we will collect symptoms and side effects from symptom diaries and participant telephone calls.

Common side effects of doxycycline include: Angioedema; diarrhoea; headache; Henoch-Schönlein purpura; hypersensitivity; nausea/vomiting; pericarditis; skin and photosensitivity reaction; dyspnoea; hypotension; peripheral oedema; tachycardia.

© Copyright: The University of Oxford and Oxford University Hospitals NHS Foundation Trust 2019

26 APPENDIX G: USUAL CARE PLUS INHALED CORTICOSTEROID (ICS) ARM

1. Background and rationale

a. Evidence for potential benefits of inhaled corticosteroids in COVID-19 illness

Inhaled corticosteroids (ICS) are a commonly prescribed class of medication throughout the world. They are reasonably cheap and have been used widely for the last 60 years. The inhaled action and type2 pneumocyte target of COVID make ICS a potential therapeutic agent in COVID-19[1]. They have been shown to be very effective in improving asthma and COPD care over the long term, where the recommendation is that most, if not all, patients with asthma should be prescribed an inhaled corticosteroid[2,3] and up to 90% of patients with COPD in the UK are prescribed ICS[4]. The rationale of ICS is to reduce the inflammatory process that underlies exacerbations, which can be triggered by viruses in asthma and COPD. Systemic corticosteroids have been found to be effective at reducing mortality amongst hospitalised patients with COVID-19 [46, 47], but it is not known whether pre-hospital treatment with ICS is also beneficial.

Further evidence is as described below:

Evidence from the ARDS literature
ICS in patients at risk of acute respiratory distress syndrome (ARDS) have been shown to improve physiology and reduce inflammatory markers[5]. In patients admitted to hospital at risk of ARDS or acute lung injury, there was an almost 50% reduction of ARDS in patients that were using ICS pre-admission, even controlling for covariates such as age, gender and chronic respiratory disease[6]. Moreover, this ICS effect can also be seen to improve pulmonary physiology[7].

Potential mechanism of efficacy
Recently published *in vitro* data suggest a role for ICS inhibition of coronavirus replication in infected epithelial cells[8], whilst there is an indication that there is accelerated hyperinflammation at the onset of SARS-CoV-2 infection[9], which potentially can be modified by anti-inflammatory therapy. This suggests a plausible mechanism for ICS efficacy against COVID-19 in which ICS has a dual role: firstly, toning down the inflammatory “runaway train” (ARDS-like) response affecting a minority of COVID-19 patients; and secondly, inhibiting viral replication. It has long been known that the ICS effect on epithelial cells is as a direct consequence of gene transcription[10], and investigation of gene expression of ACE2 and TMPRSS2 in the sputum of asthmatic patients has very recently demonstrated lower expression of these key receptors in the presence of ICS[11]. Furthermore, ICS attenuates expression of the ACE2 receptor in human and murine in vitro and in vivo models[12]. This is of relevance as the SARS-CoV-2 mechanism of action is upon direct action of the ACE2 receptor, a receptor highly expressed on epithelial cells in the oral mucosa and type 2 alveolar cells and the serine protease TMPRSS2 for SARS-CoV-2 spike protein priming[13,14]. Furthermore, there is experimental evidence that inhaled corticosteroids inhibit coronavirus replication in vitro[15,16]. SARS-CoV-2 binds to cells via the angiotensin converting enzyme 2 (ACE2) receptor. ACE2 is highly expressed on epithelial cells in the oral mucosa and type 2 alveolar epithelial cells. The use of inhaled corticosteroids as a therapy suggests it would target the cells of interest. Furthermore, the primary action of the inhaled steroids is on the type 2 pneumocytes where viral replication is going to be at its most, where we know that ACE2 receptor expression is high.

© Copyright: The University of Oxford and Oxford University Hospitals NHS Foundation Trust 2019

2. Changes to outcome measures

The addition of this arm will not require any changes to outcome measures.

3. Eligibility criteria specifically related to ICS

Inclusion criteria: No changes

Exclusion criteria:

- A known allergy to inhaled corticosteroids
- Any known contraindication to inhaled corticosteroids (as per SmPC, *patients with rare hereditary problems of galactose intolerance, the Lapp lactase deficiency or glucose-galactose malabsorption should not take this medicine. Lactose, the excipient in the product, contains small amounts of milk proteins and can therefore cause allergic reactions*).
- Patient currently prescribed inhaled or systemic corticosteroids
- Unable to administer inhaler

4. Detail of intervention

Participants randomised to the usual care plus ICS arm will receive usual clinical care as per NHS guidelines, plus inhaled corticosteroids for 14 days. We will use the IMP distribution methods described in the protocol to deliver IMP to participants.

a. Investigational Medicinal Product (IMP) description

The IMP is the inhaled corticosteroid budesonide (dose 400mcg, Pulmicort turbohaler®). Inhaled budesonide comes in a polyethylene container consisting of a white cover screwed onto a brown bottom plate. Inside this is the inhaler with its main parts: a mouthpiece, a dosing mechanism and a substance store. The device will have 50 actuations of 400mcg/actuation. This product has marketing authorisation in the UK (PL 17901/0164) and is manufactured by AstraZeneca UK Ltd, 600 Capability Green, Luton, LU1 3LU, UK. This IMP will be taken as 2 puffs twice a day for 14 days.

b. Storage of IMP

Stored at room temperature in locked cupboards in restricted access rooms in the Nuffield Department of Primary Care Health Sciences; in locked cupboards in restricted access rooms in GP practices; in Pharmacies

c. SmPC precautions and concomitant medication

iii. Precautions

Budesonide is a commonly prescribed inhaled steroid with an established safety profile.

iv. Concomitant medications

Largely, there is no restriction to concomitant medications using inhaled budesonide. The SmPC states that concomitant treatment with ketoconazole, HIV protease inhibitors or other

© Copyright: The University of Oxford and Oxford University Hospitals NHS Foundation Trust 2019

potent CYP3A inhibitors may increase systemic budesonide levels, but that this is of little clinical significance for a short term treatment of 2 weeks, which is the duration of IMP use in the trial.

5. Safety reporting

Mechanisms for safety reporting are outlined in the protocol. In brief, we will collect symptoms and side effects from symptom diaries and participant telephone calls.

Common and/or potential side effects from IMP include:

- Cough immediately after inhaling
- Mouth and throat pain
- Hoarse voice
- Oral candidiasis (thrush)

These are all reversible upon ceasing IMP

© Copyright: The University of Oxford and Oxford University Hospitals NHS Foundation Trust 2019

27. Supplementary Material

A. Abbreviations

AE	Adverse event
AR	Adverse reaction
CI	Chief Investigator
CRF	Case Report Form
CT	Clinical Trials
CTA	Clinical Trials Authorisation
CTRG	Clinical Trials and Research Governance
DMSC	Data Monitoring Committee / Data Monitoring and Safety Committee
DSUR	Development Safety Update Report
GCP	Good Clinical Practice
GP	General Practitioner
HRA	Health Research Authority
HCP	Healthcare professional
IB	Investigators Brochure
ICF	Informed Consent Form
ICH	International Conference on Harmonisation
IMP	Investigational Medicinal Product
MHRA	Medicines and Healthcare products Regulatory Agency
NHS	National Health Service
NIHR	National Institute of Health Research
RES	Research Ethics Service
PHE	Public Health England
PI	Principal Investigator
PIL	Participant/ Patient Information Leaflet
R&D	NHS Trust Research and Development Department
RCGP RSC	Royal College of General Practitioners Research Surveillance Centre
REC	Research Ethics Committee
RSI	Reference Safety Information
SAE	Serious Adverse Event
SAR	Serious Adverse Reaction
SDV	Source Data Verification
SMPC	Summary of Medicinal Product Characteristics
SOP	Standard Operating Procedure
TSC	Trial Steering Committee
SUSAR	Suspected Unexpected Serious Adverse Reactions
TMF	Trial Master File

© Copyright: The University of Oxford and Oxford University Hospitals NHS Foundation Trust 2019

B. Key Trial Contacts

**Chief Investigator**	Professor Chris Butler<br>Nuffield Department of Primary Care Health Sciences<br>Gibson Building<br>Radcliffe Observatory Quarter<br>Woodstock Road<br>Oxford<br>OX2 6GG<br>christopher.butler@phc.ox.ac.uk
**Sponsor**	Joint Research Office<br>1st floor, Boundary Brook House<br>Churchill Drive,<br>Headington<br>Oxford OX3 7GB<br>ctrg@admin.ox.ac.uk<br>Tel: +44 (0)1865572224<br>Fax: +44 (0)1865572228
**Funder(s)**	UKRI/NIHR
**Clinical Trials Unit**	Primary Care Clinical Trials Unit,<br>Nuffield Department of Primary Care Health Sciences<br>Radcliffe Observatory Quarter<br>Woodstock Road<br>Oxford<br>OX2 6GG<br>principle@phc.ox.ac.uk<br>01865 289296
**Statistician**	Ben Saville,<br>Berry Consultants,<br>Austin, Texas, USA,<br>And<br>Department of Biostatistics,<br>Vanderbilt University School of Medicine,<br>Nashville, Tennessee,<br>USA.<br><br>Dr Ly-Mee Yu<br>Primary Care Clinical Trials Unit,<br>Nuffield Department of Primary Care Health Sciences<br>Radcliffe Observatory Quarter<br>Woodstock Road<br>Oxford<br>OX2 6GG
**Committees**	**DMSC Chair:**<br>Prof. Deborah Ashby<br>Chair in Medical Statistics and Clinical Trials<br>Director of the School of Public Health<br>Imperial College London Faculty of Medicine, School of Public Health,<br>153 Medical School<br>St Mary's Campus<br>Imperial College London<br>deborah.ashby@imperial.ac.uk

© Copyright: The University of Oxford and Oxford University Hospitals NHS Foundation Trust 2019

	(0)20 7594 8704<br><br>**DMSC Members:** Prof Simon Gates<br>Cancer Research Clinical Trials Unit (CRCTU)<br>Institute of Cancer and Genomic Sciences<br>University of Birmingham<br>Edgbaston<br>Birmingham<br>B15 2TT<br>S.Gates@bham.ac.uk<br><br>Prof Gordon Taylor<br>College House,<br>University of Exeter, St Luke's Campus,<br>Heavitree Road,<br>Exeter, EX1 2LU, UK<br>g.j.taylor@exeter.ac.uk<br><br>Prof Nick Francis<br>Primary Care and Population Science,<br>University of Southampton, Southampton, UK.<br>Nick.Francis@soton.ac.uk
	**TSC Chair**<br>Prof Paul Little,<br>Primary Care and Population Science,<br>University of Southampton, Southampton, UK.<br>P.Little@soton.ac.uk<br><br>**TSC Members**<br>Prof Philip Hannaford,<br>NHS Professor of Primary Care<br>University of Aberdeen, Aberdeen, UK<br>p.hannaford@abdn.ac.uk<br><br>Prof Matt Sydes,<br>Professor of Clinical Trials & Methodology,<br>MRC Clinical Trials Unit, University College of London, London, UK<br>m.sydes@ucl.ac.uk<br><br>*PPI representatives*<br>Ms Carol Green<br>Mr Tim Mustill

© Copyright: The University of Oxford and Oxford University Hospitals NHS Foundation Trust 2019

C. Objectives and Outcome Measures

	Objectives	Outcome Measures	Timepoint (s)
Primary	To assess the effectiveness of trial treatments in reducing<br>1) Time to recovery, for patients aged ≥50 years with comorbidity, and aged ≥65 with or without comorbidity and possible COVID-19 during a time of prevalent COVID-19 disease, and<br>2) Hospitalisation and/or death.	1) Time to recovery, defined as the first instance that a participant reports feeling recovered from possible COVID-19, and<br>2) Hospitalisation and/or death	Within 28 days of randomisation<br>Patient report, Study Partner report, medical records, Daily online symptom scores
Secondary	To explore whether trial treatment reduces<br>1) Patient-reported illness severity<br>2) Duration of severe symptoms and symptom recurrence<br>3) Contacts with the health services<br>4) Consumption of antibiotics<br>5) Hospital assessment without admission<br>6) Oxygen administration<br>7) Intensive Care Unit admission<br>8) Mechanical ventilation<br>9) Duration of hospital admission<br>10) Negative effects on well being<br>11) New infections in household<br>12) To determine if effects are specific	1-2. Patient reports daily and monthly (after 28 days) symptoms.<br>3. Contacts with health services reported by patients and/or captured by reports of patients' medical records if the practice is a member of the RCGP RSC network<br>4. Bi-weekly reports from participants' primary care medical records<br>5-9. Patient report/carer report/medical record in primary and secondary care<br>9<br>10. WHO-5 Well Being Index<br>11. Reports of new infections in the household (from daily questionnaire)	Daily online symptom scores.<br>Telephone call or text on days 2, 7, 14 and 28 and once a month for 12 months if data is not obtained through the online diary.<br><br>GP notes review if available through Oxford RCGP RSC network; otherwise, other sources of routinely collected data after 28 days. Medical notes review for up to 10 years.<br><br>HES/ONS/EMIS/Medical record data linkage after 28 days if patients have been assessed in hospital<br><br>Swab result from medical records, the supporting laboratory

© Copyright: The University of Oxford and Oxford University Hospitals NHS Foundation Trust 2019

	to those with a positive test for SARS-CoV-2	12. Swab test results will indicate an "Intention to Treat Infected" group within the overall cohort for sub analysis. Blood test results on recovery (optional) for evidence of historic COVID-19	and/or convalescent blood test result for evidence of historic COVID-19<br><br>WHO 5 Well Being Index at baseline, day 14, and day 28 and monthly for up to 12 months, either via online diary or telephone
Qualitative sub-study	1. To explore patients' experiences of consulting, being tested and taking (trial) medication for possible COVID-19.<br>2. To explore healthcare professionals' views of taking part in research during pandemics.	1. Telephone interviews with patients.<br>2. Telephone interviews with healthcare professionals.	1. After 28 days.<br>2. Once practice has completed recruitment.
Intervention(s)	All trial interventions are detailed in the Appendices. Further interventions may be added or replaced during the course of the trial, subject to suitable interventions becoming available and all necessary approvals being obtained.		
Comparator	In the first instance, this will be a two-arm trial, with the intervention arm being usual care plus a trial drug and the comparator being usual care. There will be no placebo control in this study. Additional arms may be added as the trial progresses. These will be detailed in the Appendices. If an intervention arm is shown to be superior, then this will become the new standard of care. However, the primary analysis of subsequent interventions will correspond to the comparison versus the original Usual Care arm.		

© Copyright: The University of Oxford and Oxford University Hospitals NHS Foundation Trust 2019

D. Adverse Events

Definitions

Adverse Event (AE)	Any untoward medical occurrence in a participant to whom a medicinal product has been administered, including occurrences which are not necessarily caused by or related to that product.
Adverse Reaction (AR)	An untoward and unintended response in a participant to an investigational medicinal product which is related to any dose administered to that participant.<br>The phrase "response to an investigational medicinal product" means that a causal relationship between a trial medication and an AE is at least a reasonable possibility, i.e. the relationship cannot be ruled out.<br>All cases judged by either the reporting medically qualified professional or the Sponsor as having a reasonable suspected causal relationship to the trial medication qualify as adverse reactions.
Serious Adverse Event (SAE)	A serious adverse event is any untoward medical occurrence that:<br>• results in death<br>• is life-threatening<br>• requires inpatient hospitalisation or prolongation of existing hospitalisation<br>• results in persistent or significant disability/incapacity<br>• consists of a congenital anomaly or birth defect*.<br>Other ‘important medical events’ may also be considered a serious adverse event when, based upon appropriate medical judgement, the event may jeopardise the participant and may require medical or surgical intervention to prevent one of the outcomes listed above.<br>NOTE: The term "life-threatening" in the definition of "serious" refers to an event in which the participant was at risk of death at the time of the event; it does not refer to an event which hypothetically might have caused death if it were more severe.<br>*NOTE: Pregnancy is not, in itself an SAE. In the event that a participant or his/her partner becomes pregnant whilst taking part in a clinical trial or during a stage where the foetus could have been exposed to the medicinal product (in the case of the active substance or one of its metabolites having a long half-life) the pregnancy should be followed up by the investigator until delivery for congenital abnormality or birth defect, at which point it would fall within the definition of “serious”.

© Copyright: The University of Oxford and Oxford University Hospitals NHS Foundation Trust 2019

Serious Adverse Reaction (SAR)	An adverse event that is both serious and, in the opinion of the reporting Investigator, believed with reasonable probability to be due to one of the trial treatments, based on the information provided.
Suspected Unexpected Serious Adverse Reaction (SUSAR)	A serious adverse reaction, the nature and severity of which is not consistent with the Reference Safety Information for the medicinal product in question set out:<br>• in the case of a product with a marketing authorisation, in the approved summary of product characteristics (SmPC) for that product<br>• in the case of any other investigational medicinal product, in the approved investigator's brochure (IB) relating to the trial in question.

NB: To avoid confusion or misunderstanding the difference between the terms "serious" and "severe", the following note of clarification is provided: "Severe" is often used to describe intensity of a specific event, which <u>may</u> be of relatively minor medical significance. "Seriousness"

E. Data Recording and Record Keeping

The data will be entered into the CRFs in an electronic format by the participant, Trial Partner or trial team (using OpenClinica™ database via Sentry). OpenClinica™ is stored on a secure server – data will be entered in a web browser and then transferred to the OpenClinica Database by encrypted (Https) transfer. OpenClinica™ meets FDA part 11B standards. This includes safety data, laboratory data and outcome data. Safety data will also be collected through electronic diaries which are stored on a secure server.

Sentry is an online secure data entry system developed in-house at PC-CTU and hosted at Oxford. It is designed to collect sensitive data, such as participant and Trial Partner contact details, and securely retain them separate form a trial's clinical data. Sentry can also act as a central participant portal to manage online eligibility, eConsent and ePRO - acting as an intermediary between the participant and the clinical databases. Sentry is accessed via a secure HTTPS connection and all stored sensitive data is encrypted at rest to AES-256 standards. Participant and Trial Partner data will be kept and stored securely for as long as it's required by the study and reviewed on annual basis.

F. Qualitative Sub-study

With consent, participants will be contacted for a telephone interview within three months after they complete their day 28 follow up. The researcher will provide study information over the telephone and the Interview Patient PIS, and ICF will be available on the study website and emailed to participants if requested.

© Copyright: The University of Oxford and Oxford University Hospitals NHS Foundation Trust 2019

Once a practice has completed patient recruitment and one of their patients has been interviewed, we may ask 1-2 healthcare professionals who would be willing to share their experiences of taking part in the trial. Healthcare professionals will include clinicians and non-clinicians with the main criteria for inclusion in interviews being that HCP participants should have carried out trial activities in their practice. Potential HCP participants will be contacted in person or by email by the practice contact. They will be provided with the Interview HCP Invitation Email, Interview HCP PIS and Interview HCP ICF by email.

Patients recruited to both the intervention and usual care arms will be purposively sampled across the recruiting period with approximately 15-20 patients in each arm (30-40 interviews in total). We will seek to obtain maximum variation in age and symptom severity (as reported in daily diary at baseline). When the research team receives responses from HCPs, they will collect basic demographics to purposively select participants based on practice location, practice size, practice patient recruitment and job role. We aim to complete 20-25 interviews with HCPs.

All participants will only be required to take part in a single interview. Patient participant interviews will follow a semi-structured topic guide (Interview Patient Topic Guide) and ask about reasons for consulting and illness perceptions prior to the consultation, experiences of the consultation, the COVID-19 testing process (if applicable, and result if the participant has been notified) and medication adherence. The topic guide will be informed by the Common Sense Model which describes how people perceive and cope with symptoms of illness.
HCP interviews will follow the Interview HCP Topic Guide and will ask about experiences of carrying out trial activities, recruiting patients and the work required to set up a clinical trial during a pandemic.

Interviews with patient participants are expected to last approximately 30-45 minutes and interviews with HCPs are expected to last 15-30 minutes.

Data Collection:
Each interview will be audio-recorded with the participant’s permission. Recordings will allow verbatim transcription of interviews. Transcription will be completed by an independent transcription company. Once transcribed and transcripts are checked, audio-recordings will be deleted. Transcripts will be labelled with a unique participant number and will omit any identifiable data either identifying the participant or their general practice.

© Copyright: The University of Oxford and Oxford University Hospitals NHS Foundation Trust 2019

Berry Consultants
Statistical Innovation

Appendix 2

PRINCIPLE
Adaptive Design Report
Version 3.4

Ben Saville, Ph.D.
Nick Berry, Ph.D.

November 6, 2020

Contents

1 Trial Overview **3**

2 Co-primary Hypotheses **3**
2.1 Time to Recovery . . . 3
2.2 Hospital Admission or Death . . . 4

3 Co-primary Analyses **4**
3.1 Time to Recovery Analysis Model . . . 4
3.1.1 Adjustment for Temporal Changes . . . 5
3.2 Hospitalization/Death Analysis Model . . . 6
3.3 Sensitivity Analyses . . . 6
3.4 Secondary Analyses . . . 7

4 Adaptive Design **7**
4.1 Interim Analyses for Superiority & Futility . . . 7
4.1.1 Interim Superiority . . . 8
4.1.2 Interim Futility . . . 8
4.1.3 Maximum Sample Size per Arm . . . 9
4.2 Additional Treatment Arms . . . 9
4.3 Allocation . . . 9
4.3.1 Response Adaptive Randomization . . . 10
4.3.2 Adding Interventions . . . 11
4.3.3 Comparing Interventions . . . 11
4.3.4 Standard of Care . . . 11
4.3.5 Combination Interventions . . . 11
4.3.6 Arm Suspension . . . 11
4.3.7 Eligibility Exclusions by Intervention . . . 12
4.4 Sample Size Justification . . . 12

5 Simulated Operating Characteristics **12**
5.1 Simulation Scenarios . . . 13
5.2 Operating Characteristics Base Scenarios (Three Interventions) . . . 13
5.3 OCs Additional Scenarios: Null Effect Hospitalization . . . 34
5.4 OCs Additional Scenarios: Modified Time to Recovery Rate for Usual Care Arm . . . 40
5.5 OCs Additional Scenarios: Fast Accrual . . . 50

6 Simulated Example Trials **57**
6.1 Simulated Example Trials: Three Interventions . . . 57
6.2 Example 1 . . . 58
6.3 Example 2 . . . 66
6.4 Example 3 . . . 74
6.5 Example 4 . . . 88
6.6 Computational Details . . . 100

Berry Consultants
Statistical Innovation

1 Trial Overview

PRINCIPLE is an open, adaptive, platform trial to evaluate emerging treatments of the novel COVID-19 virus in participants aged ≥ 65 years or ≥ 50 years with comorbidity or in high risk subgroups. The purpose of this document is to define the primary analysis and adaptive design of the PRINCIPLE trial. Complete details of all planned analyses are described in the Master Statistical Analysis Plan (M-SAP).

A "platform trial" is a trial in which multiple treatments for the same disease are tested simultaneously. The backbone of the trial is an adaptive clinical trial design. Pre-specified decision criteria allow for dropping a treatment for futility, declaring a treatment superior, or adding a new treatment to be tested. If at any point a treatment is deemed superior to Usual Care for both co-primary endpoints, the superior treatment will replace Usual Care as the new standard of care. Because the process of dropping and adding treatments may be on-going for an indefinite period of time, platform trials may be better conceived of as a process rather than a singular clinical trial. In the context of the COVID-19 pandemic, the trial may continue as long as the pandemic persists.

The PRINCIPLE trial will begin as a 1:1 randomized trial of Hydroxychloroquine versus Usual Care and will have the capability to add additional interventions over time. The evaluation of any new interventions will be governed by the master protocol, including adaptive and decision criteria. In addition, the inclusion of any new interventions will require supplementary appendices to the protocol and M-SAP.

2 Co-primary Hypotheses

The trial has two co-primary endpoints. The first co-primary endpoint is time to recovery from suspected COVID-19 infection within 28 days from randomization, where time to recovery is defined as the first instance that a participant reports feeling recovered. The second co-primary endpoint is hospital admission or death related to suspected COVID-19 infection within 28 days from randomization.

Unless otherwise specified in the intervention-specific appendices, the co-primary outcomes will be analysed using a "gate-keeping" strategy. For a given treatment, time to recovery will be analysed first, and if the first null hypothesis is rejected, the second co-primary endpoint of hospitalization/death will be subsequently analysed. This gate-keeping strategy preserves the overall Type I error of the primary endpoints without additional adjustments for multiple hypotheses. In addition, the gate-keeping structure reflects the clinical belief that an intervention is unlikely to demonstrate benefit on the hospitalization/death endpoint without first demonstrating benefit on the time to recovery endpoint.

2.1 Time to Recovery

The first primary analysis is a Bayesian piecewise exponential of time to recovery regressed on treatment and stratification covariates (age, comorbidity or high risk). Let θ_j for $j > 0$ denote the log hazard ratio for time to recovery for persons on intervention j versus the Usual Care arm ($j = 0$), where $\theta_j > 0$ corresponds to faster recovery. Based on a Bayesian posterior distribution of θ_j, the primary analysis for intervention j will test the following hypothesis:

$$\begin{aligned} H_0 &: \theta_j \leq 0 \\ H_1 &: \theta_j > 0 \end{aligned} \tag{1}$$

If the Bayesian posterior probability of beneficial treatment effect (ψ_{1j} in equation 2) is greater than or equal to 0.99,

$$\psi_{1j} = \Pr(\theta_j > 0) \tag{2}$$

the null hypothesis will be rejected and the intervention will be deemed superior to Usual Care. The decision criteria controls the one-sided Type I error of each intervention at approximately 0.025.

2.2 Hospital Admission or Death

The second co-primary analysis is a Bayesian generalized linear model of the primary outcome regressed on treatment and stratification covariates (age, comorbidity or high risk). Let p_j denotes the probability of hospitalization/death for persons in treatment group j, where j=0 denotes the Usual Care arm and $j = 1$ denotes an intervention arm. A Bayesian posterior distribution will be derived for the estimated difference in probability of hospitalization/death between treatment groups. Let δ_j denote the log odds ratio of hospitalization/death comparing intervention j to Usual Care. The primary analysis for intervention j will test the following hypothesis:

$$\begin{aligned} H_0 &: \delta_j \geq 0 \\ H_1 &: \delta_j < 0 \end{aligned} \tag{3}$$

If the Bayesian posterior probability of beneficial treatment effect ψ_{2j} is greater than or equal to 0.975, with ψ_{2j} given in (4),

$$\psi_{2j} = \Pr(\delta_j < 0) \tag{4}$$

the null hypothesis will be rejected and the intervention will be deemed superior to Usual Care with respect to Hospitalization/Death. Note the decision criterion is lower than the recovery endpoint decision criterion (due to the gate-keeping structure), and controls the one-sided Type I error of each intervention at approximately 0.025 for plausible scenarios.

The primary analysis population is defined as all randomized participants for whom data are available, and are analysed according to the groups they are randomized to. Secondary analyses will conduct the primary analysis on the subset of participants with confirmed COVID-19.

3 Co-primary Analyses

3.1 Time to Recovery Analysis Model

Let T_{ij} be the progression event time (in days) for time to recovery for the ith subject in arm j, where an event represents a positive outcome for the subject. We model the event times as piecewise exponential:

$$T_{ij} \sim PE(\lambda_{ij1}, \lambda_{ij2}, \lambda_{ij3}, \lambda_{ij4}), \tag{5}$$

where $\boldsymbol{\Lambda} = (\lambda_{ij1}, \lambda_{ij2}, \lambda_{ij3}, \lambda_{ij4})$ represents the set of hazard rates (events per day per subject) within each time segment s corresponding to subject i. The hazard rate for subject i on treatment j within time segment s is given by Equation (6),

$$\lambda_{ijs} = \exp(\gamma_s + \theta_j + \boldsymbol{x}_i'\boldsymbol{\beta} + \eta_{t(i)}), \tag{6}$$

where γ_s is the log hazard rate corresponding to time segment s, θ_j is the log hazard ratio for treatment relative to Usual Care, $\boldsymbol{x}_i$ is a vector of stratification covariates (age, comorbidity or high risk), $\boldsymbol{\beta}$ the corresponding vector of covariate parameters, and $\eta_{(i)}$ is a time drift parameter described in Section (3.1.1).

The log hazard rate corresponding to time segment s is given by equation (7),

$$\gamma_s = \alpha_1 + \alpha_2 I(s = 2) + \alpha_3 I(s = 3) + \alpha_4 I(s = 4) \tag{7}$$

where I() is an indicator function equal to 1 if the condition is satisfied and 0 otherwise, α_1 is the log hazard rate corresponding to time segment one (0-7 days), α_2 is the increment in log hazards for time segment two (8-14 days), α_3 is the increment in log hazards for time segment three (15-21 days), and α_4 is the increment in log hazards for time segment four (> 21 days). The prior distributions corresponding to the time segment parameters are given by equation (8),

$$\begin{aligned} \alpha_1 &\sim N(-2.3, 0.3^2) \\ \alpha_s &\sim N(0, 0.3^2) \text{ for } s > 1 \end{aligned} \tag{8}$$

where α_1 is centered at a hazard rate of 0.10, with increments in the log hazards centered at 0 for subsequent time segments, all with weakly informative variance.

For a time to recovery endpoint, treatment hazard ratios greater than one indicate treatment benefit. The log hazard ratio for treatment has the weak informative prior

$$\theta_j \sim N(0, 0.3^2), \tag{9}$$

and is assumed to be constant over time. The weak informative prior for the log hazard ratio places the prior mass of the HR between 0.5 and 2.0, which in line with clinical expectations for potential therapies, and also will be quickly overwhelmed with accruing data. A similar prior distribution is used for the covariate parameters given by equation (10),

$$\beta_k \sim N(0, 0.3^2) \tag{10}$$

where the covariate parameters are also assumed to be constant over time. Subjects with values of time to recovery equal to 0 days will be excluded from the analysis. Subjects with values for time to recovery greater than 28 days will be censored at 28 days. Subjects with incomplete diary data will be censored at the last date of entry. However, subjects who are censored due to death before first recovery will be censored at 28 days.

3.1.1 Adjustment for Temporal Changes

In the COVID-19 pandemic setting, there is a risk of temporal changes in the time to recovery, for example due to changes in virus prevalence, strain severity, or clinical care. Hence treatment arm comparisons based on non-concurrently randomized participants (e.g. due to staggered entry) can provide misleading results if temporal changes are not appropriately accounted for. In addition, the primary analysis for each intervention arm is based on the comparison of the intervention versus Usual Care (see exception for combination arms in Section 4.3.5). This is true even when an intervention replaces the Usual Care arm as the new standard of care. The rationale for comparing each arm to Usual Care, even when a superior arm has replaced Usual Care as the new standard of care, is because in a pandemic setting we are very interested in finding additional interventions that are superior to Usual Care (e.g. a "second best" intervention). This would be important if there are global supply shortages for the new standard of care. Hence adjustment for potential temporal changes is essential to the primary analysis. The temporal adjustment is made possible due to the overlapping enrollment across the respective treatment arms.

Bayesian methods are used to smooth the estimates across time intervals over the course of the trial. More specifically, we define 2-week time intervals from the start of the trial, and count backwards from the interval at the time of the data cut ($t = 1$) to the start of the trial, i.e. "walking backwards" in time. Let $\eta_{t(i)}$ denote the time offset parameter corresponding to the time of randomization for subject i. We set $\eta_1 = \eta_2 = 0$ corresponding to $t = 1$ and $t = 2$, i.e. for subjects randomized in the most recent two intervals. For every previous 2-week interval, the time parameter is modeled with the following Bayesian second order normal dynamic linear model (NDLM):

$$\eta_t \sim N(2\eta_{t-1} - \eta_{t-2}, \tau_\eta^2), \text{ for } t \geq 3 \tag{11}$$

The drift parameter τ_η^2 specifies the degree of smoothing over the time intervals, for which the following hyperprior distribution is used,

$$\tau_\eta^2 \sim \text{InvGamma}(a = 2, b = 0.0125) \tag{12}$$

and the inverse gamma (InvGamma) distribution is given by:

$$p(x) = \frac{b^a e^{-b/x}}{x^{a+1}\Gamma(a)} \tag{13}$$

This hyperprior distribution for τ_η^2 is equivalent to 4 intervals of data with τ_η centered at 0.079, i.e. a prior that time interval effects have small changes from one interval to the next. The individual time interval effects will be heavily shaped by the data from patients within the intervals. If there are insufficient numbers of patients within a given interval, time intervals will be collapsed as necessary to ensure stable model estimates.

Although the temporal adjustment in the primary analysis model is pre-specified, the unblinded statistical analysis committee may make adjustments to the temporal components as needed (e.g. change in prior distributions or length of intervals) to provide stable estimates of model parameters.

3.2 Hospitalization/Death Analysis Model

We define the second co-primary Bayesian analysis model for hospital admission or death related to suspected COVID-19. Among participants who have the opportunity to complete 28 days of follow-up, let Y_{ij} be the primary outcome measured at 28 days for subject i on arm j, with $j = 0$ denoting the Usual Care (control) and j denotes an intervention arm. We model the primary outcome as

$$\begin{aligned} \text{logit}(p_{ij}) &= \gamma_0 + \delta_j z_{ij} + \boldsymbol{x}'_{ij}\boldsymbol{\beta} \\ Y_{ij} &\sim \text{Bernoulli}(p_{ij}) \end{aligned} \tag{14}$$

where p_{ij} is the probability of hospital admission/death for patient i on arm j; γ_0 is the log odds of hospitalization for the Usual Care arm (at reference levels of covariates); z_j is an indicator variable equal to 1 if subject i is randomized to intervention j (for $j > 0$) and 0 otherwise; δ_j is the log odds ratio of hospitalization/death comparing intervention j to Usual Care; $\boldsymbol{x}_{ij}$ is a vector of stratification covariates (age, comorbidity or high risk) specific to subject i and arm j; and $\boldsymbol{\beta}$ is a vector of corresponding covariate log odds ratios. The priors are given by:

$$\begin{aligned} \gamma_0 &\sim N(0, 2^2) \\ \delta_j &\sim N(0, 2^2) \text{ for } j \geq 1 \\ \beta_k &\sim N(0, 2^2) \text{ for } k = 1, 2, \ldots \end{aligned} \tag{15}$$

which are non-informative (disperse) prior distributions on the logit scale, and are expected to be overwhelmed with increasing number of events. If the number of events are insufficient to provide stable model estimates, the primary analysis model may be modified by removing the stratification covariates from the model. Note there is no adjustment for drift over time for this endpoint, as the number of hospitalization/death events is not expected to be sufficient to robustly model temporal change.

3.3 Sensitivity Analyses

Upon conclusion of the study or intervention (and at interims as needed), the following sensitivity analyses are planned with respect to the primary outcome

- A separate stand-alone analysis will be conducted for each completed intervention, in which each intervention is compared Usual Care using only concurrent randomizations. These analyses will take the same form as the primary analysis models but may require modified priors/parameters for the temporal adjustment. For some interventions (e.g. hydroxychloroquine), temporal adjustment may not be necessary for this sensitivity analysis.

- Subjects with missing data for a given endpoint will not contribute data to the respective primary analysis. We plan the following sensitivity analyses to address missing data due to loss of follow-up:
  - Analyses will be conducted comparing data of persons with and without complete 28-days follow-up. Bayesian multiple imputation strategies of the primary analysis will be considered if there are characteristics or outcomes found to be associated with the primary endpoint.

3.4 Secondary Analyses

Upon completion of each intervention (e.g. futility), the following secondary analyses may be conducted (details provided in M-SAP):

- The co-primary analyses will be replicated for the "intent to treat infected" (ITT-I) population, i.e. comparing time to recovery between treatment groups, among individuals who test positive for COVID-19.
- Comparison of symptoms (per daily diary) between treatment groups
- Other secondary analyses listed in the M-SAP

Because other interventions may still be accruing information and using Usual Care subjects in their respective analyses, caution should be used in reporting and/or publishing results of an intervention, and will be limited to the analyses pre-specified in the M-SAP. Further guidance is provided in the PRINCIPLE Data Sharing/Access Policy.

4 Adaptive Design

The pre-specified design will allow adaptations to the trial based on the observed data. These adaptations include the declaration of superiority or futility of an arm at an interim analysis, the addition or removal of treatment arms, and changes in the randomization probabilities. Adaptations will occur at a given interim analysis if pre-specified conditions are satisfied. This adaptive design report was written while the trial was randomizing participants, but was finalized prior to conducting the first interim analysis.

4.1 Interim Analyses for Superiority & Futility

The timing of the first interim analysis will be determined by the trial management group (TMG) and statistical analysis committee (SAC), and will include logistical and operational considerations. Subsequent interim analyses will be conducted on a monthly basis provided the accrual rate is is approximately 25 or less participants per week. If accrual increases to approximately 50 participants per week, interims may be conducted every 2 weeks. If accrual increases above approximately 100 participants per week, interims may be conducted weekly. The TMG and SAC will monitor accrual rates and will have flexibility to adapt the frequency of the interim analysis to the observed accrual rate, with the goal of interim analyses occurring approximately every 100 completed observations. At each interim analysis, all enrolled intervention arms will be evaluated for superiority using the Bayesian interim primary analysis, provided that the intervention arm has at least 100 randomized participants with the opportunity to complete 28-days of follow-up for the primary endpoint. In addition, at each interim analysis all enrolled intervention arms will be evaluated for futility using the Bayesian interim primary analysis, provided that the intervention arm has at least 75 randomized participants with the opportunity to complete 28-days of follow-up for the primary endpoint.

4.1.1 Interim Superiority

If the Bayesian posterior probability of superiority of a given intervention over Usual Care is greater than or equal to 0.99 for the recovery endpoint, and greater than or equal to 0.975 for the hospitalization endpoint, superiority versus Usual Care will be declared on both endpoints, in which the superior arm will replace the Usual Care arm as the new standard of care. If a second intervention is found to be superior to the new standard of care on both endpoints, the second intervention will replace the existing standard of care. However, the primary analysis of each intervention arm (with exception for combination arms; see Section 4.3.5) will always be versus the Usual Care arm, even if participants are no longer being randomized to Usual Care.

If a decision of superiority is made for an intervention, additional enrollment and/or follow-up may continue on the randomized participants for that intervention, but any additional analyses or comparisons versus Usual Care will be considered secondary or sensitivity/exploratory analyses.

If superiority of an intervention is achieved for both primary endpoints, the DMSC will inform the Trial Steering Committee that superiority has been obtained on both endpoints, with details on size of treatment effect and probability of superiority obtained for each endpoint, and will recommend that the Trial Steering Committee (TSC) disclose the results to the Trial Management Group (TMG).

If the Bayesian posterior probability of superiority is achieved for the first co-primary endpoint (time to recovery) but not the second (hospitalization/death), the PRINCIPLE trial will continue randomizing to the Usual Care arm with allocation specified in Section (4.3.1). The DMSC will inform the TSC that superiority has been obtained on the first co-primary endpoint, with details on size of treatment effect and probability of superiority for both co-primary endpoints. The TSC will decide whether that information should be shared with the TMG based on guidelines detailed in the PRINCIPLE Data Sharing/Access Policy. In addition, interim results may be published by the TMG for a given intervention while the platform trial continues according to the PRINCIPLE Data Sharing/Access Policy.

4.1.2 Interim Futility

We define a futility rule based on the estimated median days of time to recovery. Note the primary analysis model in equation (6) produces a single additive treatment effect for each intervention on the log-hazard scale. Using the exponential framework of the model, we convert the treatment effect from a log-hazards scale to a median time to recovery, producing an estimated difference in the median days to recovery between each intervention and Usual Care. However, the treatment difference on the median time to recovery becomes approximately multiplicative rather than additive, and depends on the underlying control rate of recovery in the Usual Care population. This implies that the estimated treatment effect in median days recovery depends on covariates and on time interval (Section 3.1.1). For example, a hazard ratio of 1.2 corresponds to approximately 1.3 days benefit for an underlying Usual Care median of 8 days, but corresponds to a 2 day benefit for an underlying Usual Care median of 12 days. In order to define a futility rule using median recovery benefit, we use the underlying Usual Care median recovery for the most recent time interval, and estimate a difference in the population-averaged medians across the subgroups (based on equation 6).

Let ζ_j be the model-based difference in population-averaged median days to recovery for intervention j versus Usual Care based on equation 6 using above assumptions, i.e. the median recovery benefit in days of treatment j. Let ω_{1j} be the model-based Bayesian posterior probability of a clinically meaningful treatment effect of the recovery endpoint, defined as the probability that ζ_j is at least 1.5 days (equation 16). If the probability of a meaningful effect on recovery, ω_{1j}, is less than or equal to 0.05,

$$\omega_{1j} = \Pr(\zeta_j \geq 1.50) \tag{16}$$

intervention j will be dropped from the study for futility. If there are no other intervention arms available, the trial will be suspended; otherwise accrual continues to the remaining treatment arms.

If an intervention is superior for the first co-primary endpoint, a Bayesian posterior probability of a clinically meaningful treatment effect will be calculated for the second co-primary endpoint (hospitalization). This probability, ω_{2j}, is defined as the probability that the absolute reduction in hospitalization rate for intervention j relative to Usual Care is at least 0.02 (i.e. two percentage points) as given by equation 17. Because the primary analysis model produces a single additive treatment effect on the log odds scale, the treatment on the effect on the proportion scale becomes a multiplicative effect and depends on covariates of the Usual Care arm. Hence we define ω_{2j} using the difference in population-averaged hospitalization rates based on model 14.

If ω_{2j} is less than or equal to 0.05,

$$\omega_{2j} = \Pr(p_0 - p_j \geq 0.02) \tag{17}$$

the comparison versus Usual Care for hospitalization will be deemed futile, where p_j and p_0 are the model-based proportions of hospitalizations for intervention j and Usual Care (respectively) based on the second co-primary analysis model (equation 14). However, randomization will continue to intervention j as described in Section (4.3.1).

All futility thresholds are non-binding, meaning that the SAC and DMSC may choose to override futility decisions if they mutually agree it is in the best interests of the trial. For example, suppose there is only a single active intervention and a futility threshold is met, despite there being some evidence of a small treatment benefit. This is possible given the aggressive futility rule that is meant to find treatments with larger benefit. Rather than suspend the trial due to lack of active interventions, it may be desirable to continue randomization to the remaining intervention despite the futility threshold being met, with the TSC being informed that they should consider adding additional interventions as soon as possible.

4.1.3 Maximum Sample Size per Arm

There is no cap on sample size per arm; hence interventions will remain active in the trial unless futility criteria are met or accrual is halted for budgetary or other considerations.

4.2 Additional Treatment Arms

At any point in the trial (per recommendations of the Trial Steering Committee), the trial management group may elect to add an additional intervention to the trial. The evaluation of any new interventions will be governed by the master protocol, including adaptive and decision criteria. In addition, the inclusion of any new interventions will require supplementary appendices to the protocol and M-SAP. This includes updated simulations describing the revised operating characteristics of the trial with the additional arm.

4.3 Allocation

At the start of the trial, randomization was fixed 1:1 to hydroxychloroquine (HCQ) versus Usual Care, with stratification by age (less than 65, greater than or equal to 65), and comorbidity or high risk (yes/no). Randomization was suspended to HCQ on May 23, 2020 per the Medicine and Health Care Products Regulatory Agency (MHRA), at which time the trial began randomizing 1:1 to Azithromycin versus Usual Care. Protocol Amendment 6 included the addition of the intervention Doxycycline, which is began enrolling around the end of July 2020. This adaptive design report was finalized prior to conducting the first interim analysis, which is expected to accomplish the following tasks:

1. Evaluate primary hypotheses for Hydroxychloroquine vs. Usual Care

- Due to the sudden halt in accrual to hydroxychloroquine, there is only a single analysis evaluating hydroxychloroquine benefit versus Usual Care. Hence the posterior probability thresholds for hydroxychloroquine versus Usual Care equal 0.975 for BOTH of the co-primary endpoints. In addition, the analysis of hydroxychloroquine will include all subjects in the Usual Care arm up until the time of the interim analysis.

2. Evaluate early superiority/futility for all active interventions with a sufficient number of participants with the opportunity to complete 28 days of follow-up (per Section 4.1)

3. Determine subsequent randomization probabilities of active interventions

Subsequent interim analyses will evaluate superiority/futility of all active interventions with a sufficient number of participants with the opportunity to complete 28 days of follow-up (per Section 4.1), as well as determine subsequent randomization probabilities for all active interventions. An active intervention is defined as an intervention currently randomizing participants. Note Hydroxychloroquine will remain an inactive treatment regardless of the first interim results.

When there is more than one active intervention in the trial, the allocation probability to Usual Care will be set to $1/Z$, where Z is the total number of active arms in the trial (e.g., $Z = 3$ for Usual Care and two active interventions).

4.3.1 Response Adaptive Randomization

Prior to the first interim analysis, randomization will be set to $1/Z$ for each of the intervention arms. Response adaptive randomization (RAR) will be activated at the time of the first interim analysis if there at least two active interventions in the trial. When RAR is activated, the Usual Care arm will continue to receive a fixed allocation of $1/Z$. The remaining $(Z - 1)/Z$ allocation probability will be divided among the intervention arms based on interim RAR probabilities. The purpose of RAR is to allocate more participants to the intervention arms with the best observed outcomes (relative to Usual Care).

For example, if there are 3 active arms (2 active intervention arms and Usual Care), the Usual Care allocation will be fixed at 1/3, and the remaining 2/3 allocation will be split among the two intervention arms via response adaptive randomization. The RAR probabilities (q_j) for the intervention arms will be proportional to the Bayesian posterior probability that a given intervention is superior to Usual Care (ψ_{1j}) with respect to the first co-primary endpoint (time to recovery); see equation (2). The calculation of ψ_{1j} is based on the MCMC posterior samples from the Bayesian interim primary analysis. The randomization probabilities q_j of the intervention arms at a given interim are normalized so that they sum to $(Z - 1)/Z$.

If superiority is achieved for a given intervention for both co-primary endpoints, the superior intervention will replace the Usual Care arm and will receive a fixed $1/Z$ allocation thereafter if if there are no other interventions that have achieved superiority on either endpoint. If superiority is achieved for a given intervention, say intervention X, with respect to the first co-primary endpoint (time to recovery) but not the second co-primary endpoint (hospitalization/death), intervention X will be considered part of the "standard of care" (SOC) along with Usual Care. In this setting, intervention X and Usual Care will be both continue randomization, in which they make up a single standard of care arm receiving a total allocation probability of $1/Z^*$. The value Z^* replaces the value Z, and is equal to 1 plus the number of active interventions that have not achieved superiority on the recovery endpoint, such that the composite SOC is considered a single arm with respect to allocation. The allocation within the composite SOC will be shared equally among the arms with SOC designation.

For example, suppose there are 3 active interventions versus Usual Care, and intervention X is superior to Usual Care for the recovery endpoint but not the hospitalization endpoint. The standard

of care now refers to both intervention X and Usual Care, and the number of arms in the trial Z^* equals 3, referring to a composite SOC (composed of intervention X and Usual Care) and 2 active interventions. The two SOC arms will receive a total of $1/(2Z^*) = 1/6$ allocation each, and the remaining 2/3 allocation is assigned to the other 2 active interventions via RAR.

4.3.2 Adding Interventions

When a new intervention is added, the RAR allocation for the new arm will be bounded between $1/(2Z)$ and 1/2 for the first 4 weeks, after which this condition is removed. The remaining allocation will be given to the standard of care (fixed $1/Z$) and the other interventions, proportional to probability of superiority on time to recovery versus Usual Care (ψ_{1j}).

4.3.3 Comparing Interventions

Although the primary analysis compares each intervention versus Usual Care, the primary analysis model also enables pairwise comparisons between interventions. These pairwise comparisons will be conducted at each interim analysis. If an intervention is superior to another intervention per criterion of Bayesian posterior probability of superiority ≥ 0.99 for recovery and ≥ 0.975 for hospitalization, the inferior intervention will be dropped from the trial, regardless of how the intervention compares to Usual Care. Upon completion of enrollment for two interventions, the same criteria will be evaluated, i.e. a Bayesian posterior probability ≥ 0.99 and ≥ 0.975 for recovery and hospitalization, respectively, for one intervention versus another intervention will indicate pairwise superiority for each co-primary endpoint, with an identical gate-keeping sequential order of hypotheses for a given pairwise comparison.

4.3.4 Standard of Care

Standard of care (SOC) will initially refer to the Usual Care arm. However, if the Usual Care arm is replaced at any point by a superior intervention (due to superiority on both co-primary endpoints), standard of care would then refer to the superior intervention. If an intervention is superior to Usual Care on the first co-primary endpoint (time to recovery) but not the second (hospitalization/death), both the Usual Care arm and intervention arm will be considered standard of care arms.

4.3.5 Combination Interventions

A combination intervention is defined as a combination of one of the existing interventions plus another treatment. The primary analysis for the combination arm will be as specified in Section (3), except when the stand-alone component of the combination has been determined to be superior to Usual Care for a given primary endpoint. In such settings, the primary analysis of the combination arm will be versus the standalone component arm. For example, if azithromycin is superior to Usual Care on the first co-primary endpoint (recovery) only, and a new combination enters the trial as a combination of azithromycin plus intervention X, the primary analysis of the combination will be to evaluate superiority versus azithromycin for the first co-primary endpoint, and superiority versus Usual Care for the second co-primary endpoint. If azithromycin is superior to Usual Care on both co-primary endpoints, the primary analysis of the combination will evaluate superiority versus azithromycin for both co-primary endpoints. Note the combination arm will use the same gate-keeping strategy for evaluating the two co-primary endpoints regardless of the comparison group.

4.3.6 Arm Suspension

If the randomization probability $q_j < 0.05$ for any intervention j at a given interim, the intervention j is suspended from the trial, which is done by setting the randomization probability q_j equal to 0 until the next interim analysis. If the next interim analysis no longer meets this threshold for a previously suspended dose, randomization to the intervention may then resume according to the

algorithm. If at any point in the trial there only exist two active arms, randomization will be fixed at 1:1.

4.3.7 Eligibility Exclusions by Intervention

A participant is eligible for randomization provided the participant meets the eligibility criteria for at least two arms, one of which includes a standard of care arm. If such a participant is ineligible for one of the interventions, the randomization probabilities will be re-normalized (i.e. probabilities sum to 1.0) among the remaining arms prior to randomization. Eligibility status will be recorded for each participant as (eligible, ineligible, or unknown) for each of the treatment arms. If the number of participants who are known to eligible for one active intervention but ineligible for another active intervention becomes substantial, the primary analysis model may be modified to to include eligibility status as a covariate. However, the number of participants that this applies to is expected to be small ($\sim 5\%$); hence formal adjustment would likely lead to instability in model parameters, particularly with treatment effect parameters and temporal adjustment included in the model.

4.4 Sample Size Justification

Given the open perpetual trial structure, the trial does not have a finite ending based on sample size. Rather, the trial will continue until either superiority or futility is claimed for an intervention, or until the pandemic expires in the population. We estimate that approximately 400 participants per arm (800 participants total if only a single intervention vs. usual care) will be required to provide 90% power for detecting a difference of 2 days in median recovery time. This calculation is based on the assumption of an exponential distribution for time to recovery with a median of 9 days in the Usual Care group, with some adjustments for missing data and multiple interim analyses. Alternative assumptions are explored in the virtual trial simulations

On average, we expect fewer participants to be required when there is a large treatment benefit or complete lack of benefit. For example, if the true benefit is a 3 day benefit in median time to recovery (6 days intervention vs. 9 days Usual Care), on average only 155 subjects per arm are required to provide sufficient power. The primary advantage of the adaptive design is the ability to adapt the sample size to the observed data, thus addressing the primary hypothesis as quickly and as efficiently as possible.

In addition, we estimate that approximately 1500 participants per arm (3000 participants total if only a single intervention vs. usual care) will be required to provide 90% power for detecting a 50% reduction in the relative risk of hospitalization/death. This calculation is based on the assumption of an underlying 5% combined hospitalization/death rate in the Usual Care arm, with an intervention lowering the hospitalization/death rate to 2.5%, with some adjustments for the multiple interim analyses. We expect fewer participants to be required to detect a 50% reduction if the event rate in the Usual Care arm is greater than 5%.

For a more accurate sample size justification (via simulation) of the adaptive platform trial design, we refer to Section 5.

5 Simulated Operating Characteristics

Because of the adaptive platform trial structure, there exists no simple formula(s) to calculate power and Type I error (false positive rate). Hence, virtual trial simulations are used to fully characterize and quantify the power and Type I error of the design. The simulations include a comprehensive evaluation of trial performance across a wide range of assumptions (e.g. underlying distribution of outcome in control arm, treatment effect, accrual rates, etc.). This includes summaries regarding the number of subjects required to make a superiority or futility conclusions for each intervention.

5.1 Simulation Scenarios

The time to recovery is simulated using an exponential distribution with rate parameter λ. Based on current knowledge, we expect $\lambda_0 = \log(2)/9$ for the Usual Care arm (corresponding to a median of 9 days), but also consider lower and higher values of λ corresponding to a median of 7 and 11 days. We explore different scenarios for the hazard ratio reduction proportion hospitalized relative to control; these are given by HR=(1, 1.10, 1.20, 1.30). We also explore different scenarios for the treatment effects on hospitalization rates, where the aggregate hospitalization rate is assumed to be 0.025. This is done by imposing treatment effect sizes in absolute percentage decrease relative to the Usual Care arm equal to the null (0), small (1%), medium (2%), or large (3%) treatment effects while enforcing that the average hospitalization rate for the simulated arms (including Usual Care) equals 0.025.

At the time of writing this adaptive design report, the trial has been enrolling for approximately 6 months. Hence, our simulations include the observed accrual over the first 6 months (20-70 participants per week), followed by expected accrual thereafter based on a Poisson process. Given the uncertainty in accrual due to the state of the COVID pandemic, we consider maximum accrual rates of 30 ("expected") and 75 ("fast") subjects per week.

Given the large number of potential scenarios based on the factors listed above, simulations are conducted on a base set of scenarios with a median time to recovery of 9 days, hazard ratios of 1.0, 1.1, 1.2, and 1.3 for time to recovery, and hospitalization treatment effects of null (0%), small (1%), medium(2%), or large(3%). We then explore additional scenarios for a small subset of the base scenarios, where we 1) cross the null hospitalization scenarios with various treatment effects for the recovery endpoint; 2) cross different SOC median time to recovery rates with various treatment effects for the recovery endpoint; and 3) simulate faster accrual of 75 participants per week with bi-weekly interim analyses.

5.2 Operating Characteristics Base Scenarios (Three Interventions)

This section explores the simulated trial operating characteristics (OCs) for the first three interventions (Hydroxychloroquine, azithromycin, doxycycline) versus Usual Care using a base set of scenarios. Given simulated virtual trials, the adaptive algorithm is applied at each interim update to conduct a virtual trial exactly as described. Various summaries are captured, including the average sample size, average trial duration (in weeks), the probability of stopping accrual for early futility or superiority, and the total probability of success, where success is defined as claiming superiority versus Usual Care. Note "power" is calculated as the proportion of simulated trials that claim superiority on the primary endpoint for scenarios with a treatment benefit for a given endpoint, and Type I error is calculated as the proportion of simulated trials that claim superiority on the primary endpoint for scenarios with no treatment benefit for a given endpoint. This is done for all combinations of accrual, treatment effect, and the assumed median time to recovery in the Usual Care arm.

Principle OCs

Berry Consultants

10/26/2020

These operating characteristics are based on 1000 simulations per scenario across a variety of assumed treatment effects for experimental arms in the trial. The true number of patients randomized per day from April 1, 2020 to October 19, 2020. After this period is over, the simulations assume a 50 patient per week accrual rate. In absence of a definitive rule, these simulations conduct an interim every 28 days. In addition, the true day of enrollment stopping of HCQ is used as well as the days for opening enrollment to the Azithromycin and Doxycycline arms. As such, up to at least October 19, 2020 (day 199) of the trial, the enrollment rates to each arm in these simulations are expected to align closely with the true enrollment rates to the arms in the trial.

Median TTR	Hazard Ratio			Power for TTR				Hospitalization Rate Benefit (%)			Power for TTR & Hosp		
SOC	HCQ	Azith	Doxy	SOC	HCQ	Azith	Doxy	HCQ	Azith	Doxy	HCQ	Azith	Doxy
9	1.0	1.0	1.0	0.952	0.017	0.031	0.022	0	0	0	0.000	0.001	0.000
9	1.0	1.0	1.1	0.623	0.016	0.022	0.370	0	0	1	0.000	0.000	0.065
9	1.0	1.0	1.2	0.117	0.014	0.018	0.883	0	0	2	0.000	0.000	0.496
9	1.0	1.0	1.3	0.011	0.013	0.012	0.989	0	0	3	0.000	0.000	0.952
9	1.0	1.1	1.0	0.592	0.014	0.401	0.030	0	1	0	0.000	0.079	0.000
9	1.0	1.1	1.2	0.108	0.013	0.383	0.878	0	1	2	0.000	0.070	0.459
9	1.0	1.1	1.3	0.013	0.014	0.238	0.986	0	1	3	0.000	0.032	0.883
9	1.0	1.2	1.0	0.096	0.011	0.904	0.016	0	2	0	0.000	0.510	0.000
9	1.0	1.2	1.1	0.085	0.012	0.900	0.326	0	2	1	0.000	0.487	0.052
9	1.0	1.2	1.2	0.037	0.015	0.879	0.865	0	2	2	0.000	0.422	0.409
9	1.0	1.2	1.3	0.007	0.012	0.798	0.986	0	2	3	0.000	0.342	0.816
9	1.0	1.3	1.0	0.013	0.015	0.987	0.012	0	3	0	0.000	0.923	0.002
9	1.0	1.3	1.1	0.015	0.015	0.985	0.183	0	3	1	0.000	0.867	0.022
9	1.0	1.3	1.2	0.003	0.015	0.987	0.741	0	3	2	0.000	0.820	0.325
9	1.0	1.3	1.3	0.000	0.015	0.978	0.972	0	3	3	0.000	0.755	0.760
9	1.1	1.1	1.1	0.451	0.180	0.412	0.357	1	1	1	0.012	0.078	0.068
9	1.2	1.2	1.2	0.042	0.540	0.883	0.848	2	2	2	0.117	0.378	0.349
9	1.3	1.3	1.3	0.003	0.839	0.970	0.947	3	3	3	0.406	0.636	0.633

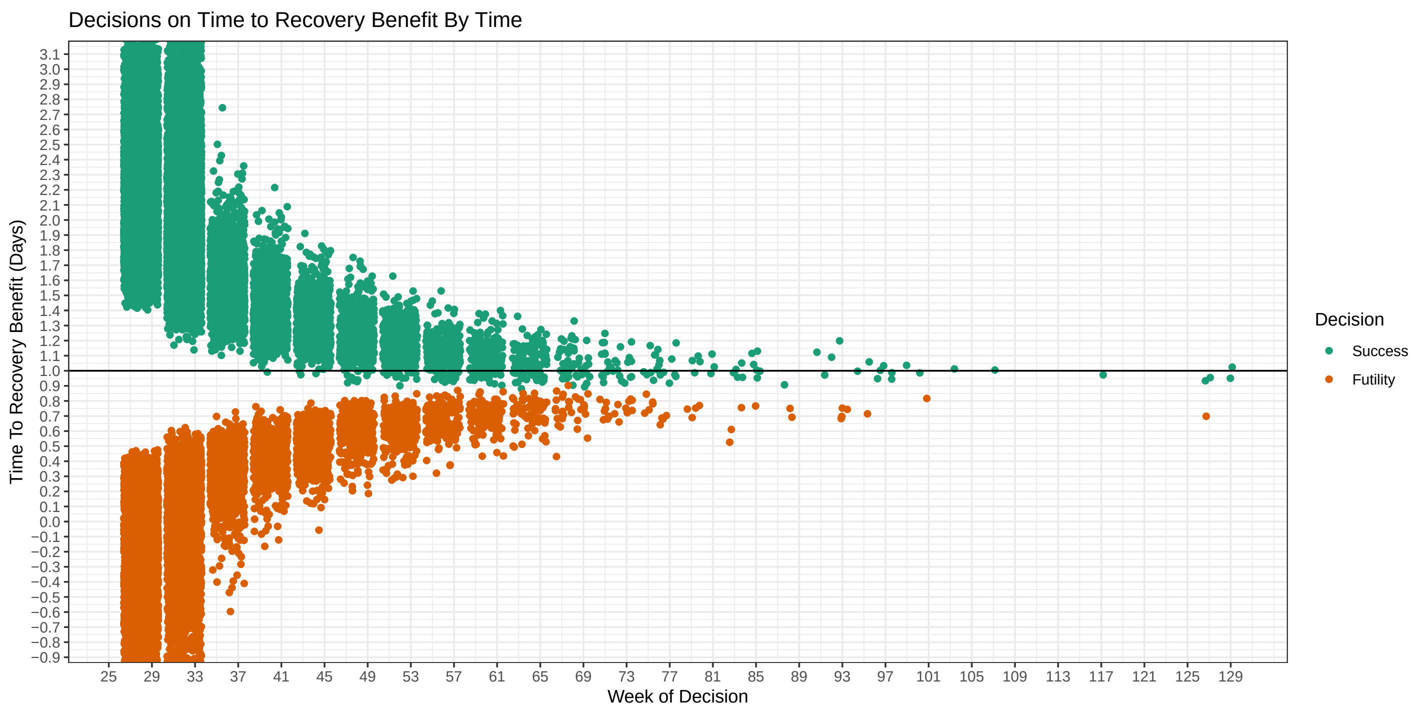

Decisions on Time to Recovery Benefit By Time
Time To Recovery Benefit (Days)
Week of Decision
Decision
Success
Futility

Hazard Ratios: HCQ: 1 Azith: 1 Doxy: 1

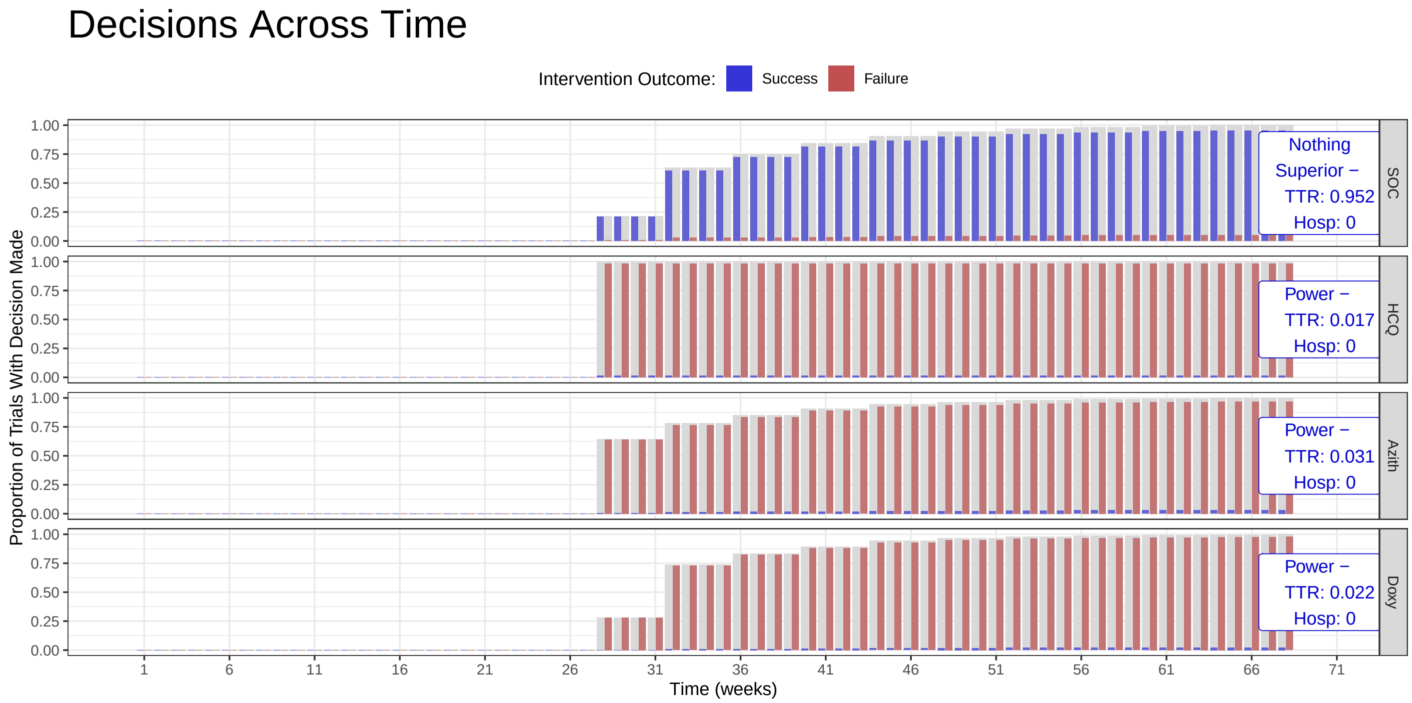

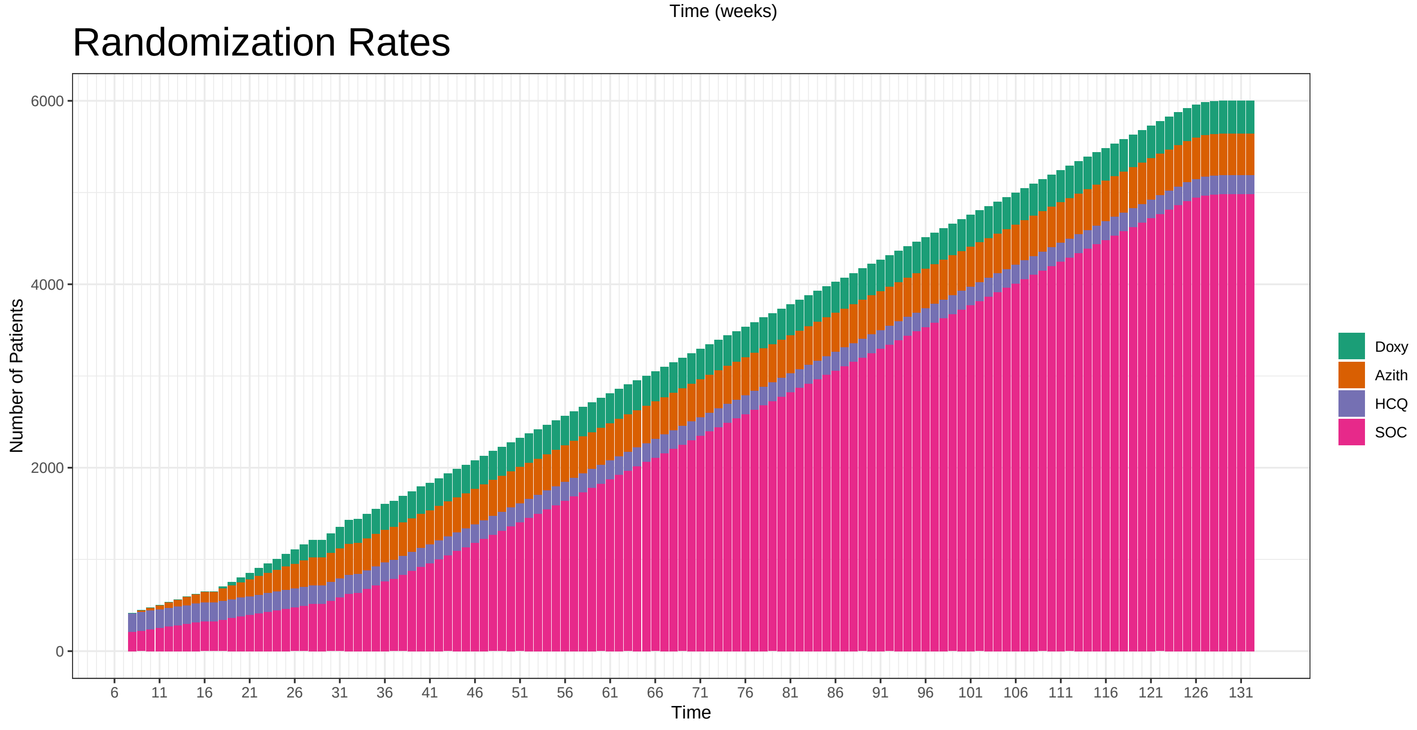

Hazard Ratios: HCQ: 1 Azith: 1 Doxy: 1.1

Decisions Across Time

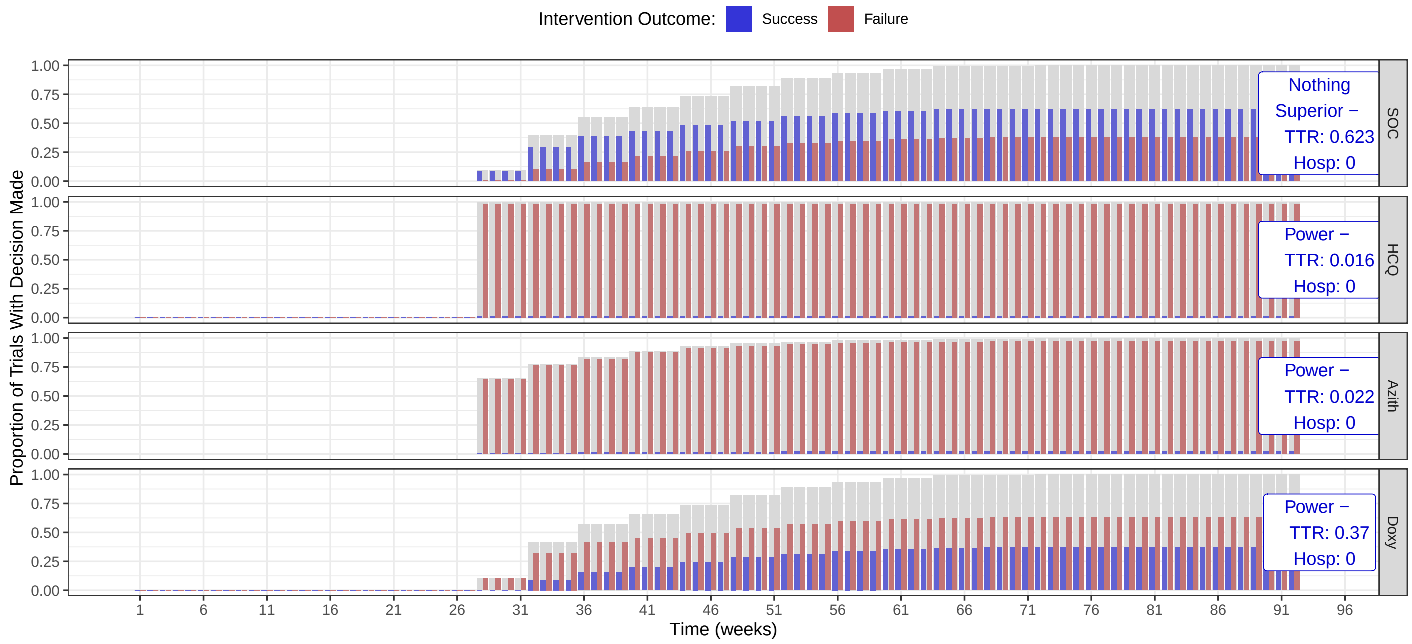

Randomization Rates

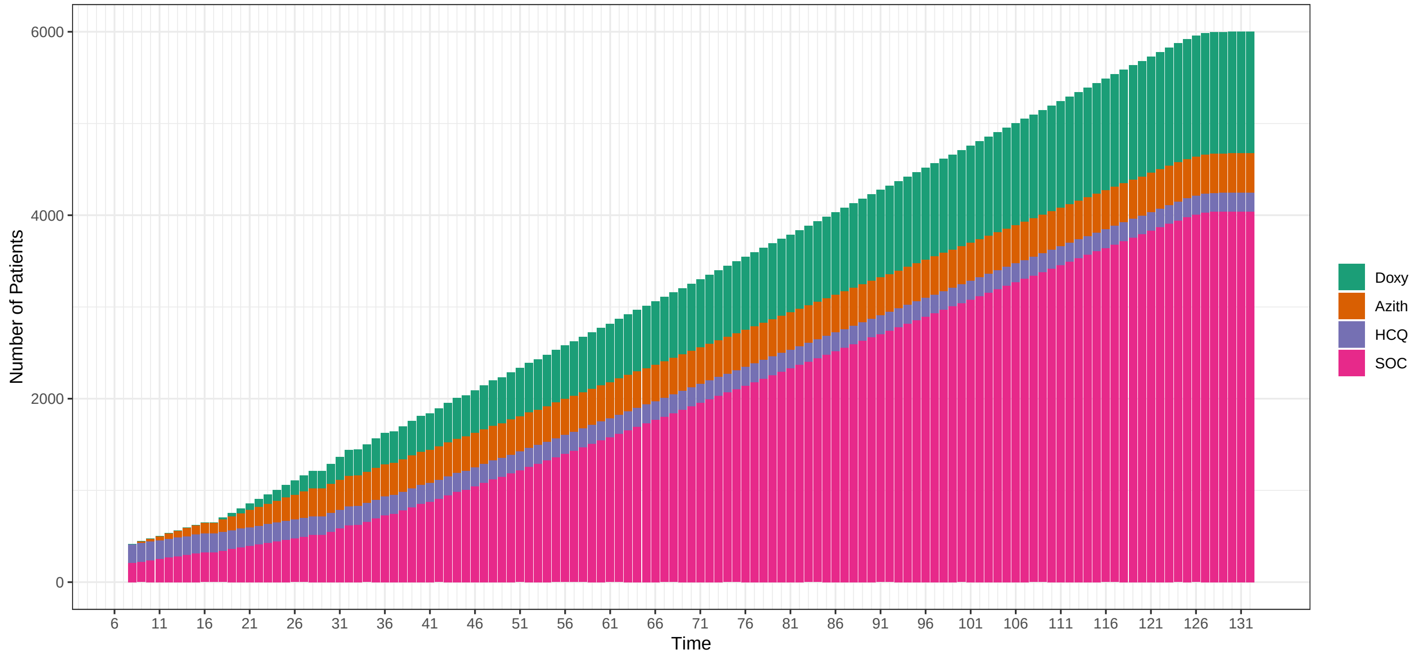

Hazard Ratios: HCQ: 1 Azith: 1 Doxy: 1.2

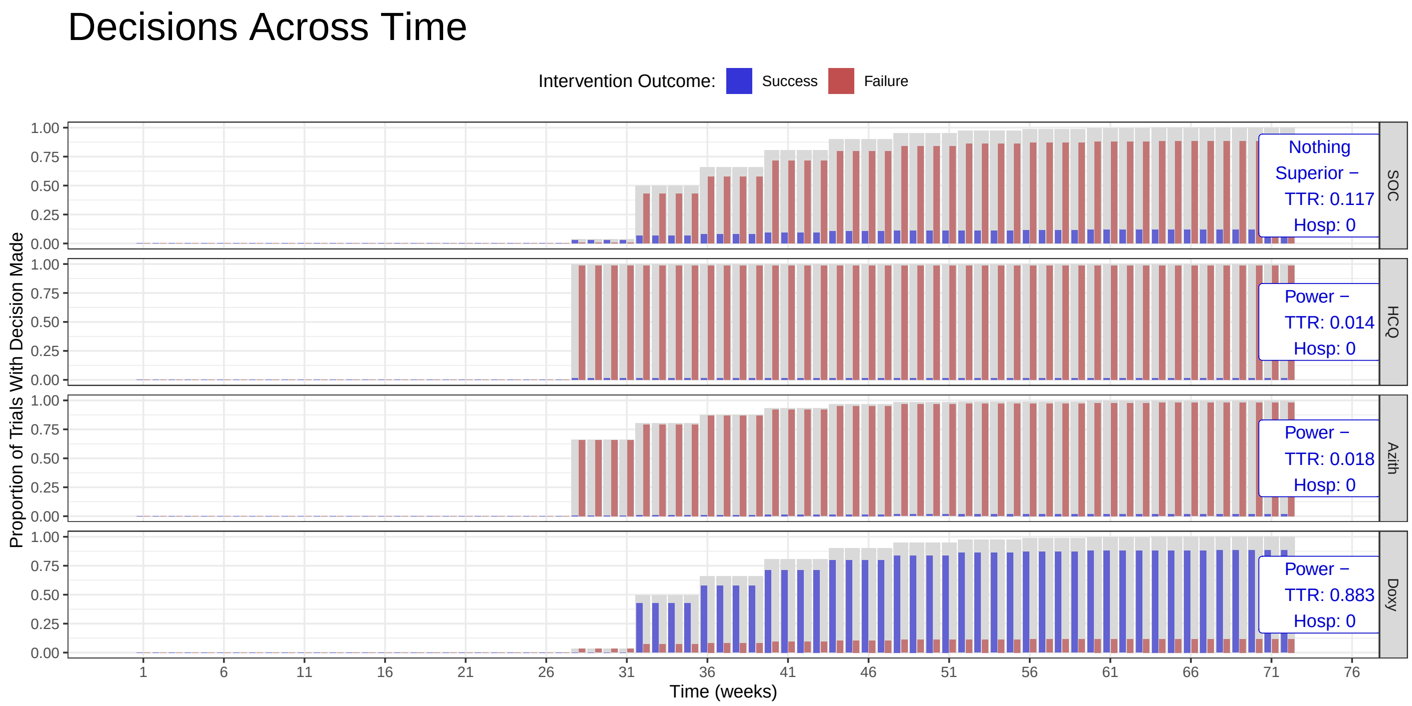

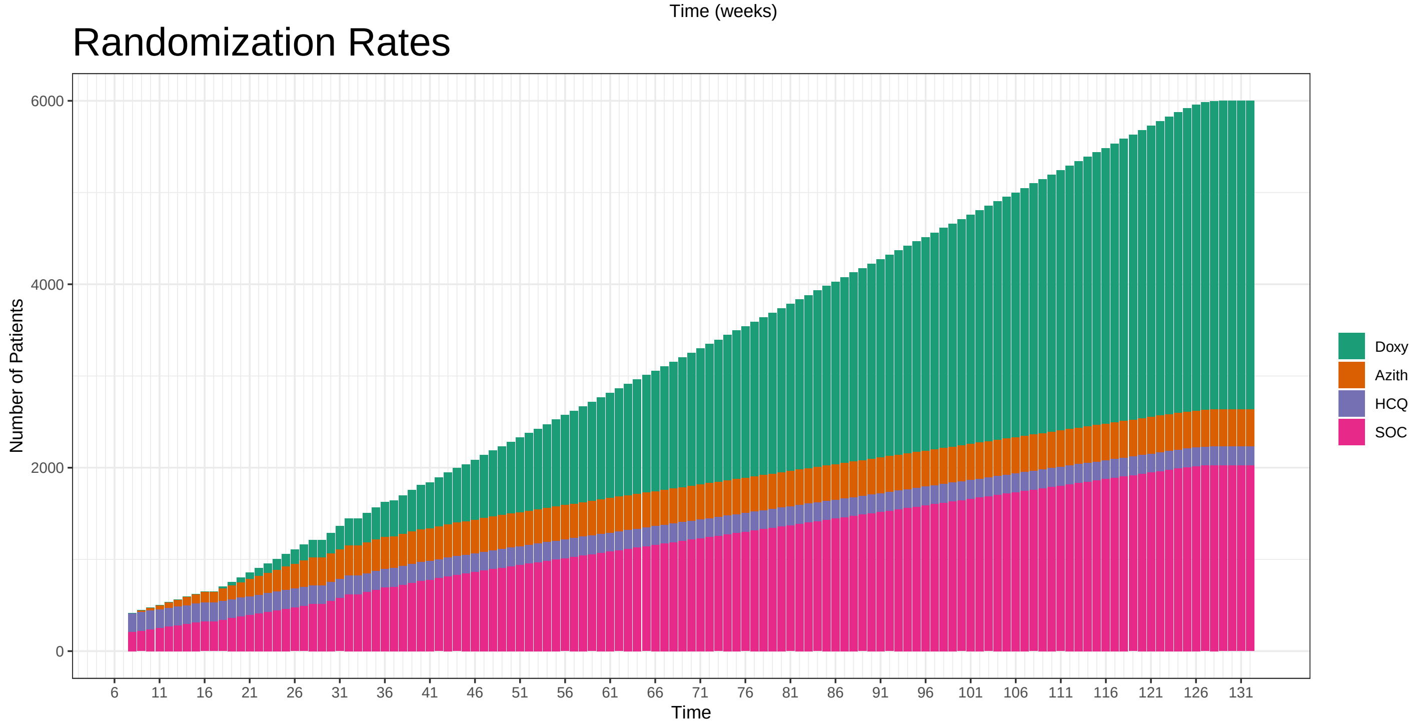

Hazard Ratios: HCQ: 1 Azith: 1 Doxy: 1.3

Decisions Across Time

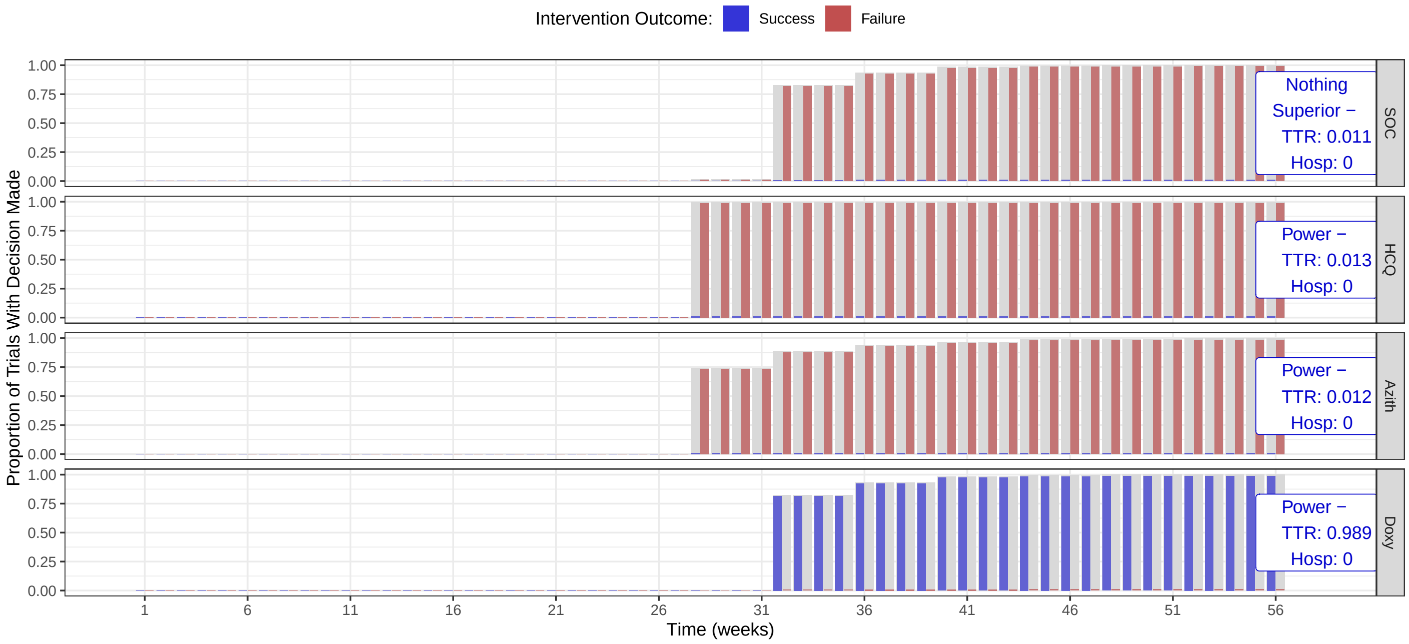

Randomization Rates

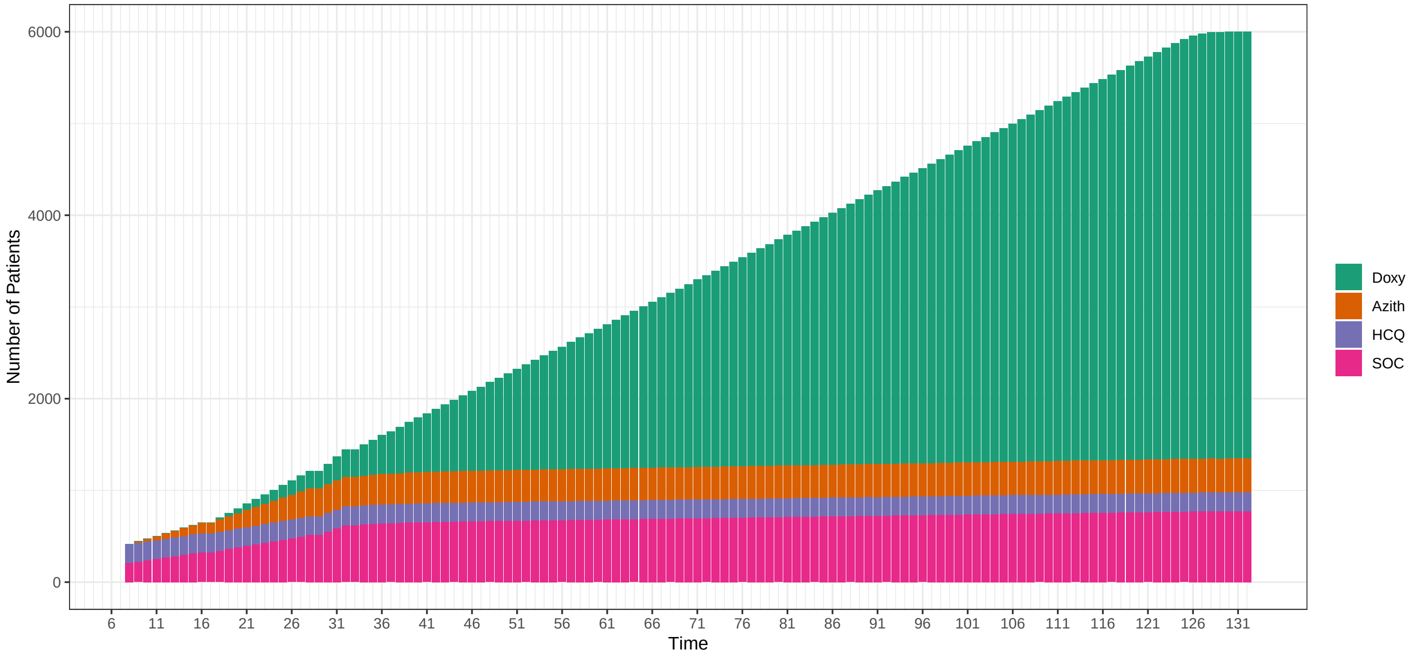

Hazard Ratios: HCQ: 1 Azith: 1.1 Doxy: 1

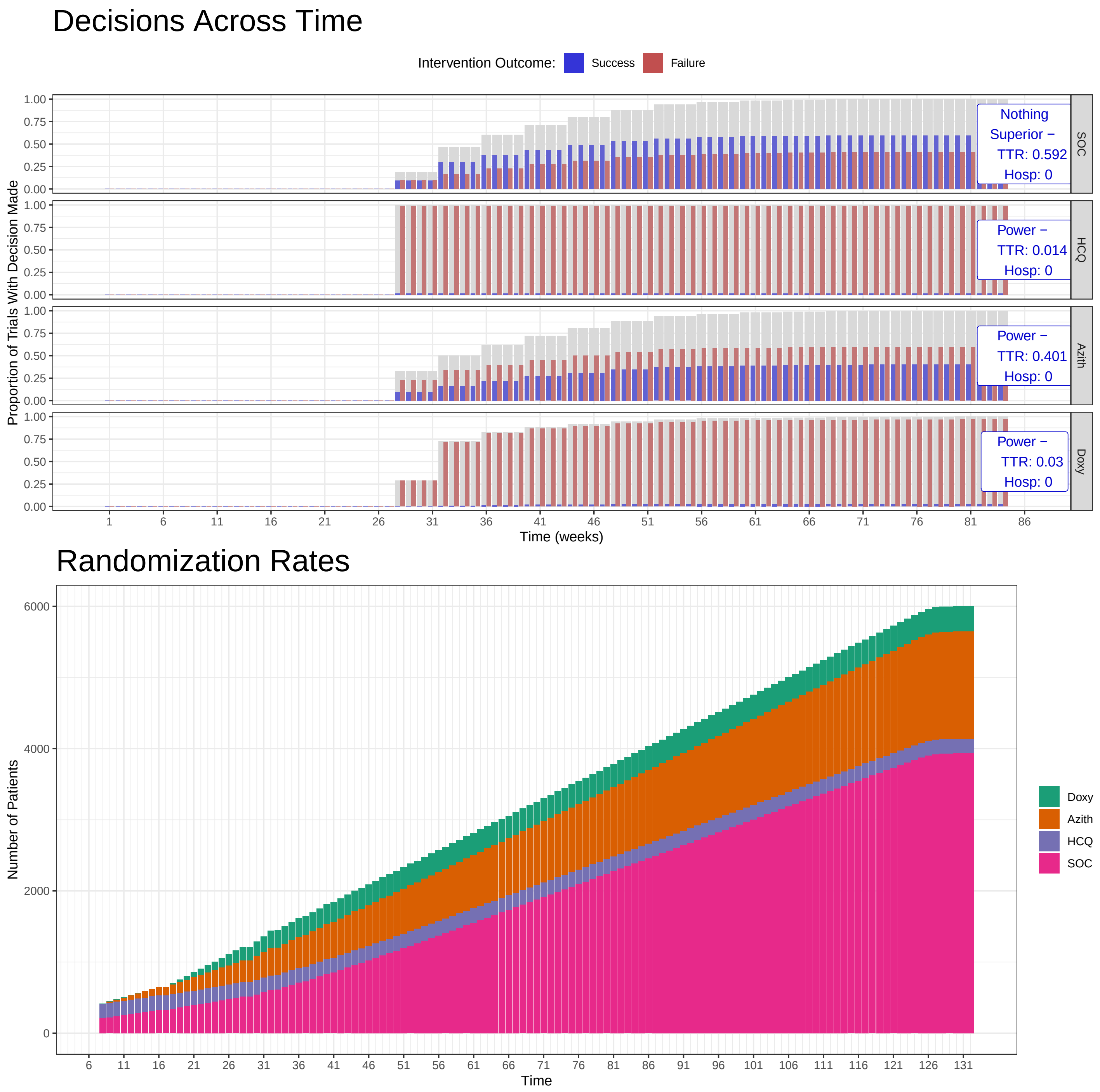

Hazard Ratios: HCQ: 1 Azith: 1.1 Doxy: 1.2

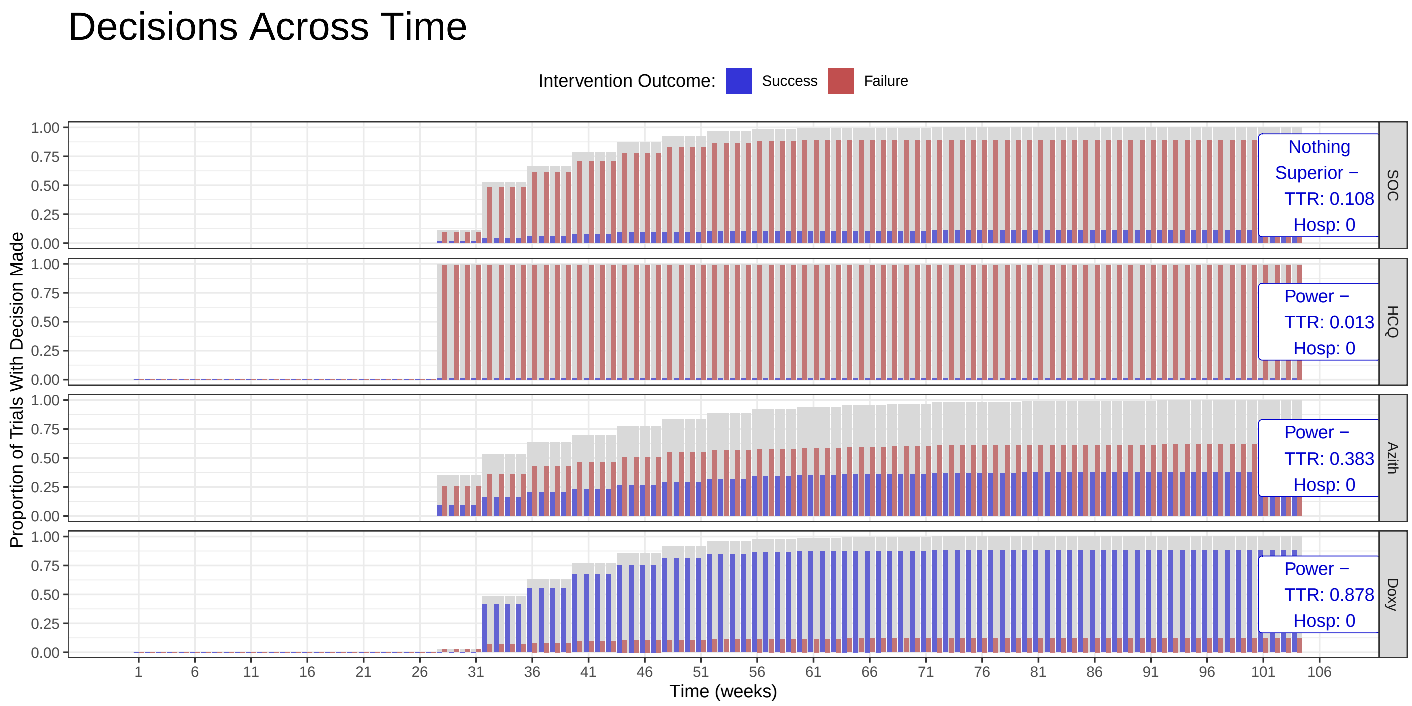

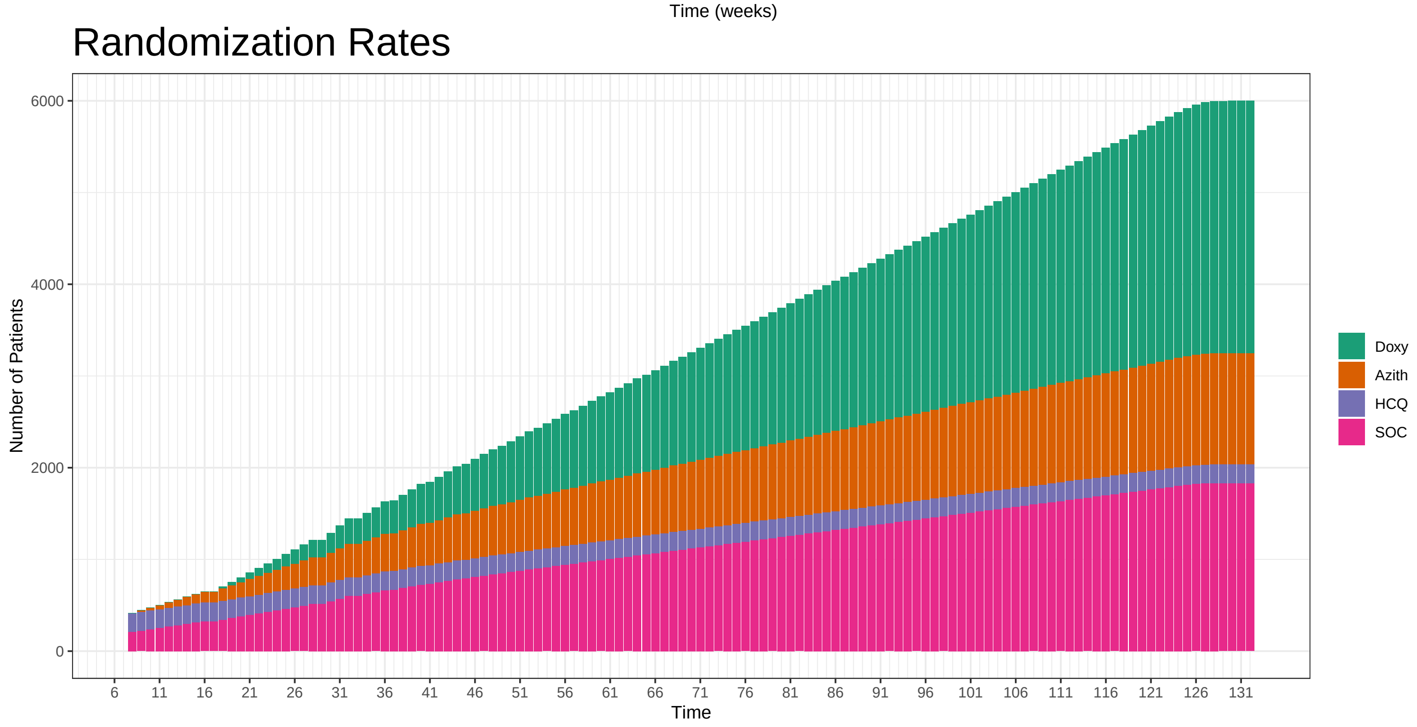

Hazard Ratios: HCQ: 1 Azith: 1.1 Doxy: 1.3

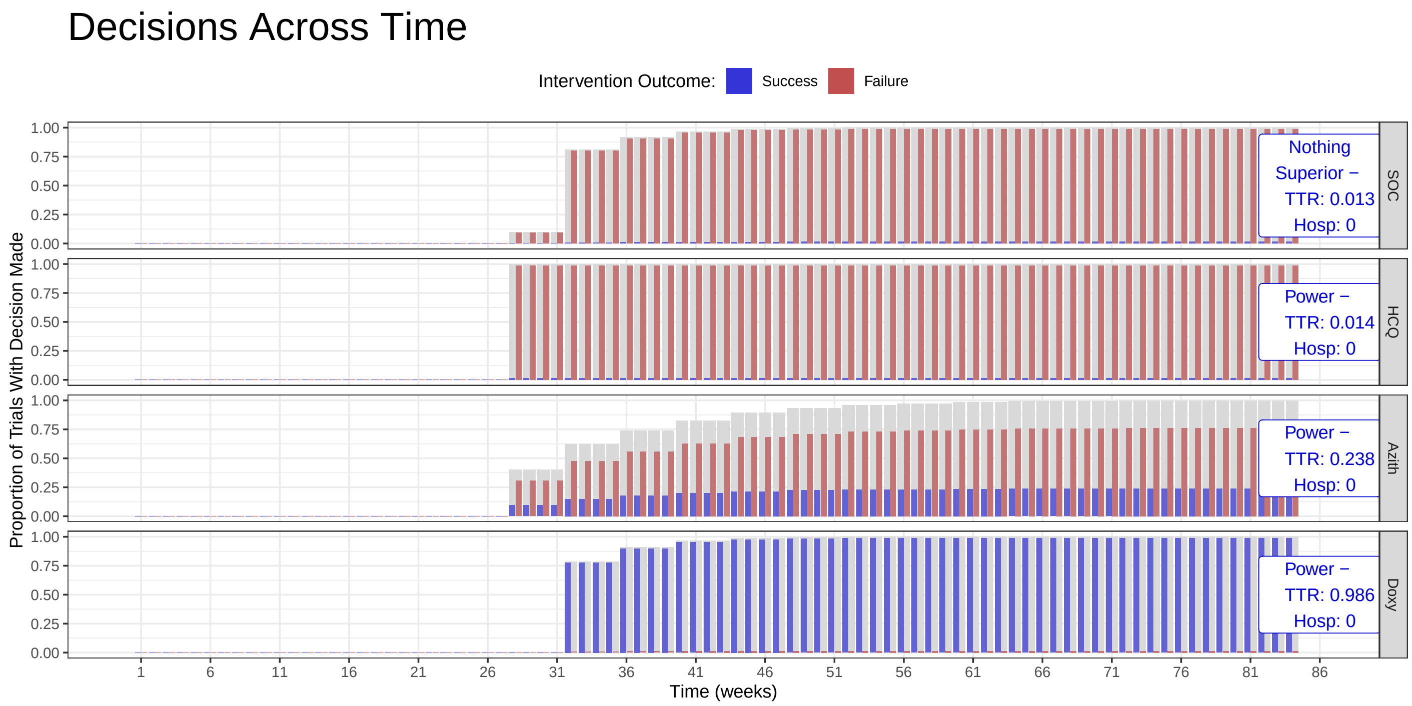

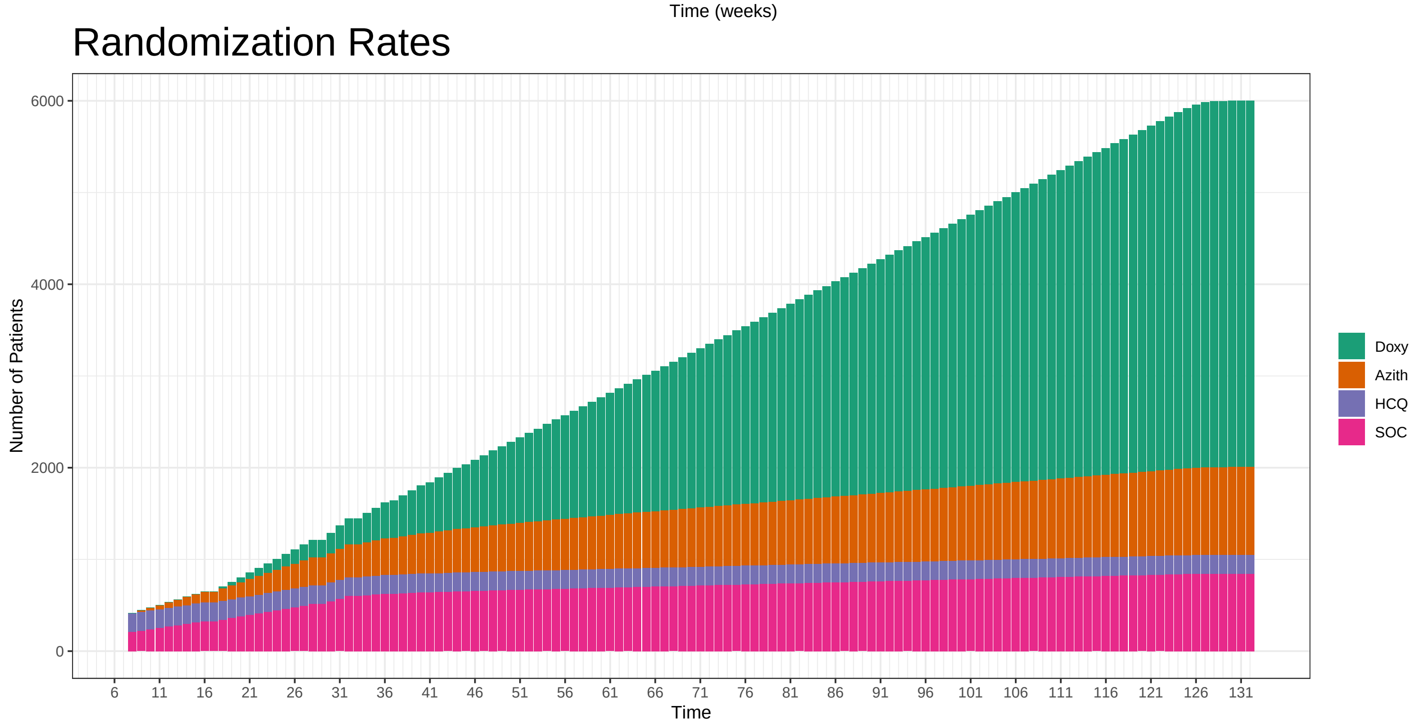

Hazard Ratios: HCQ: 1 Azith: 1.2 Doxy: 1

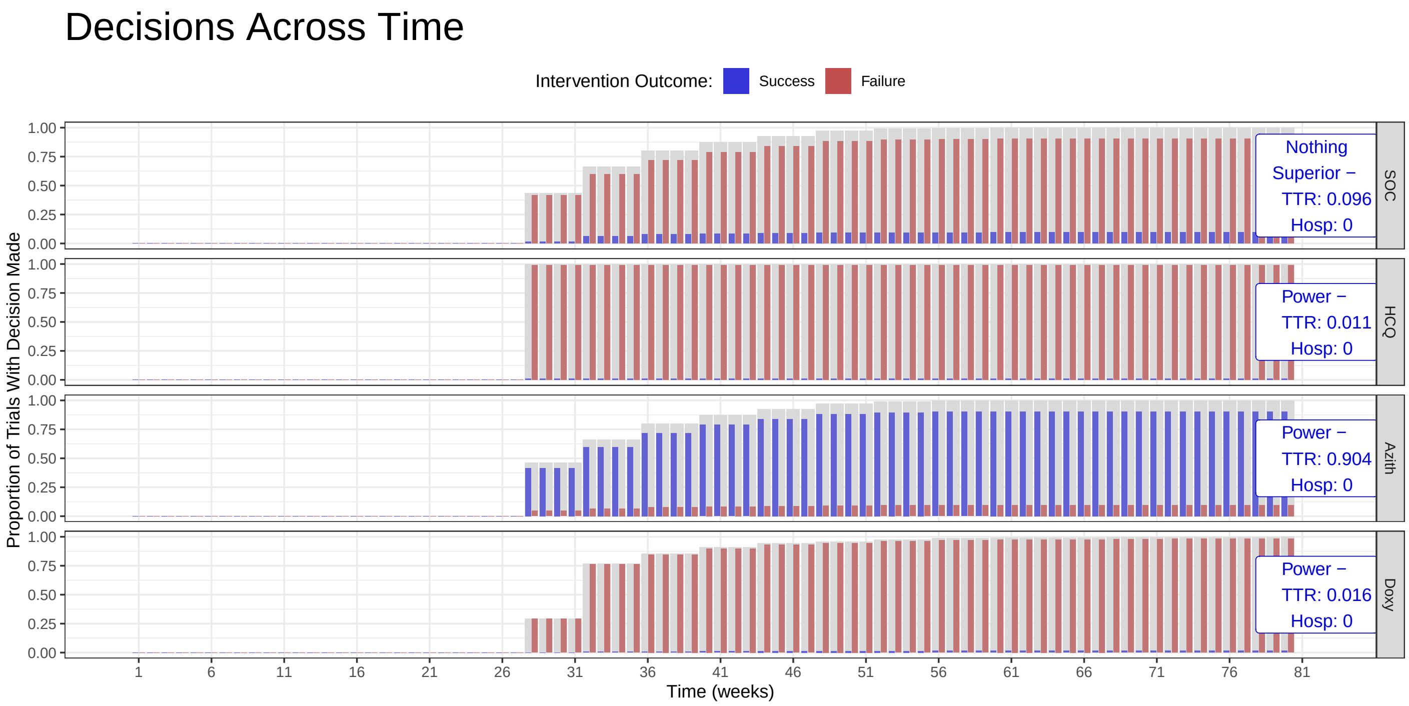

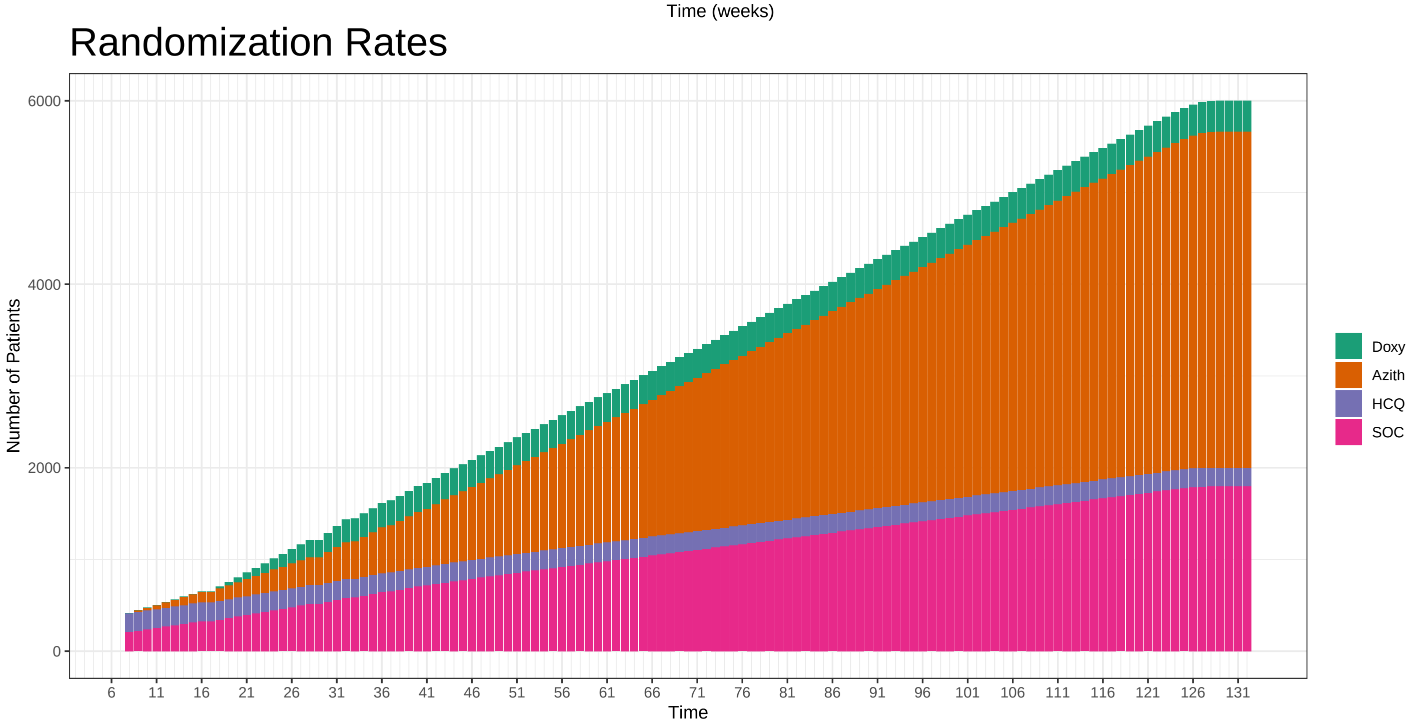

Hazard Ratios: HCQ: 1 Azith: 1.2 Doxy: 1.1

Decisions Across Time

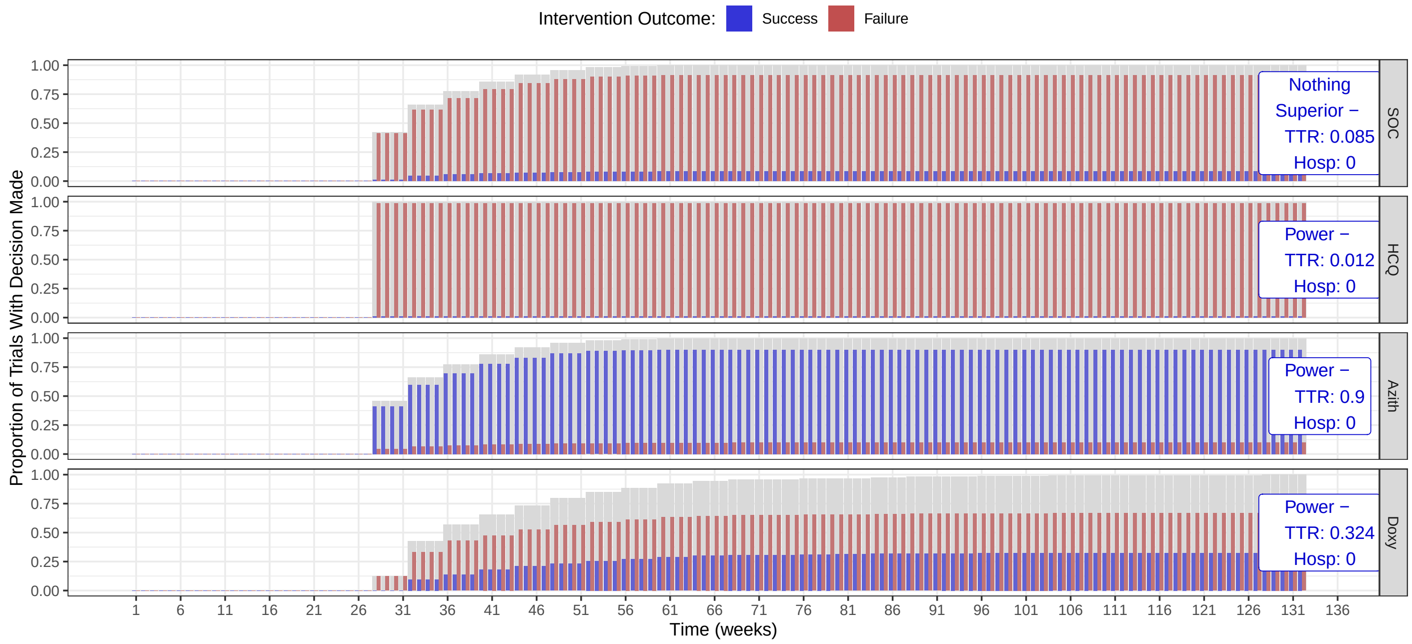

Randomization Rates

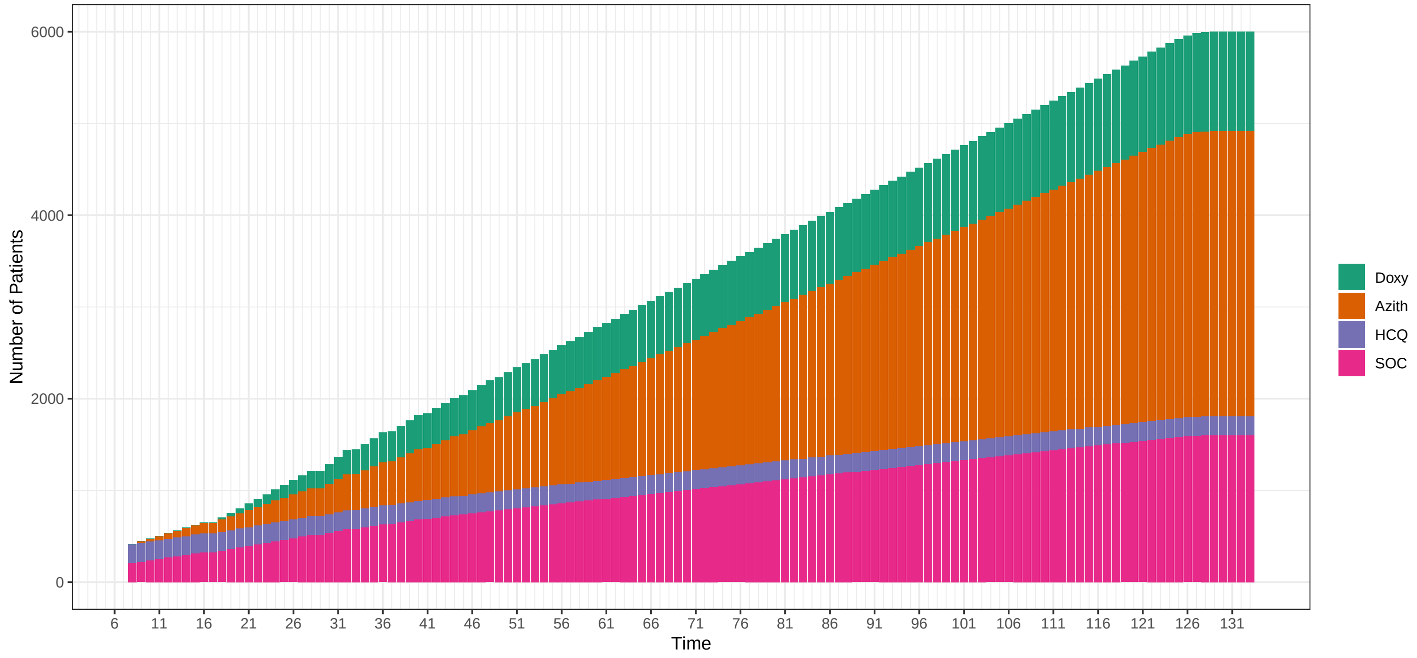

Hazard Ratios: HCQ: 1 Azith: 1.2 Doxy: 1.2

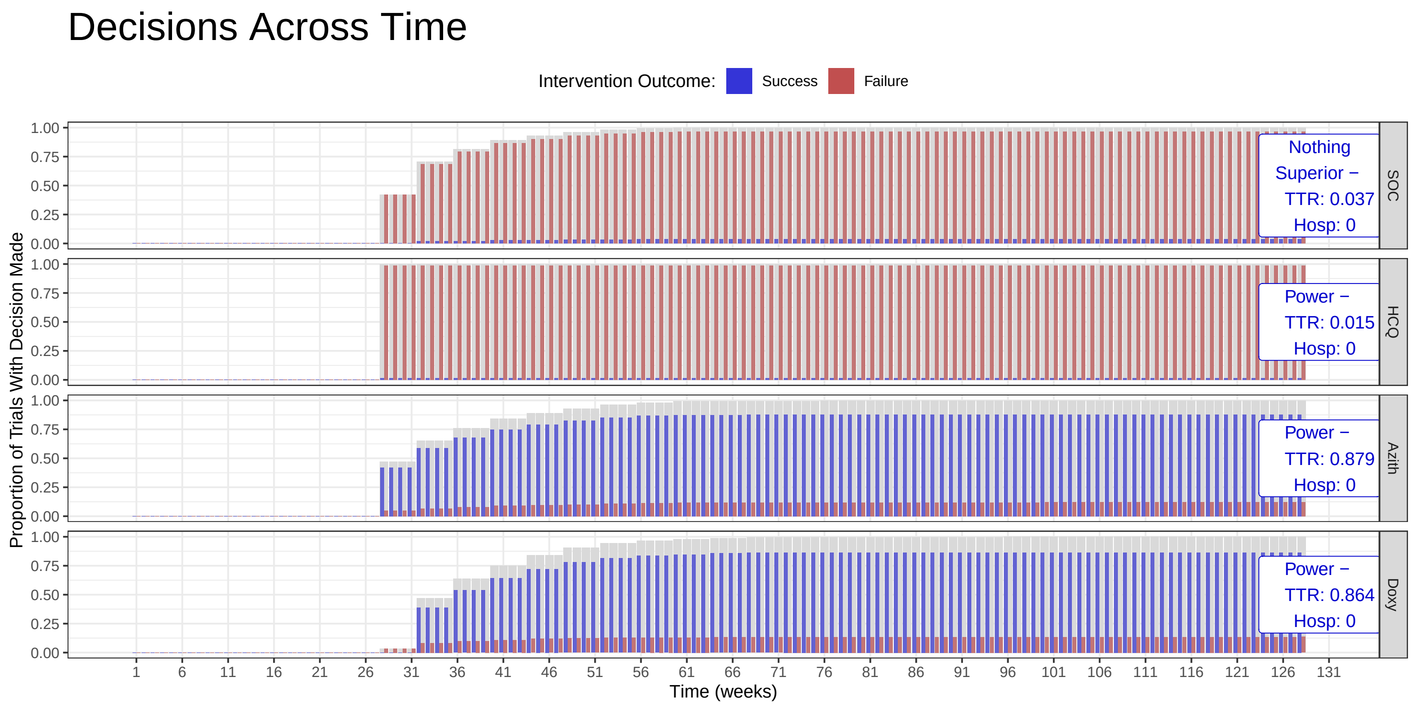

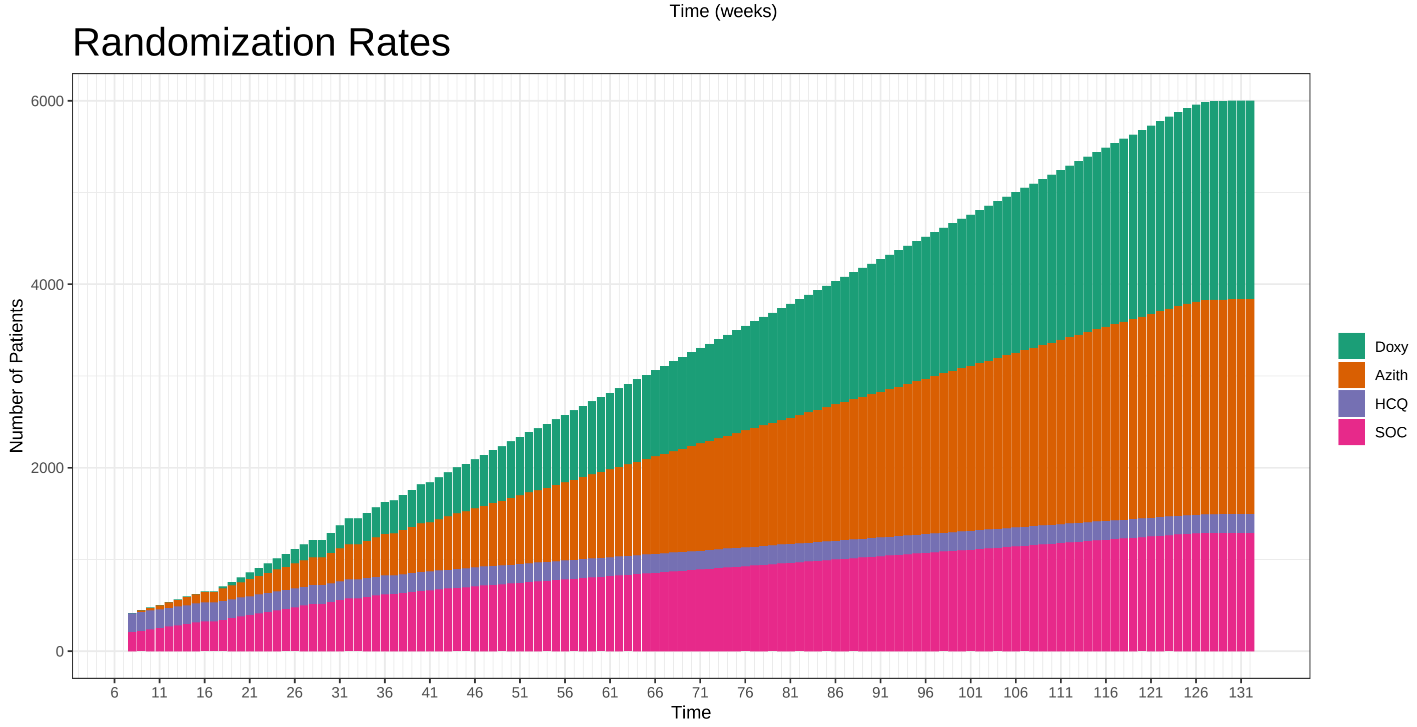

Hazard Ratios: HCQ: 1 Azith: 1.2 Doxy: 1.3

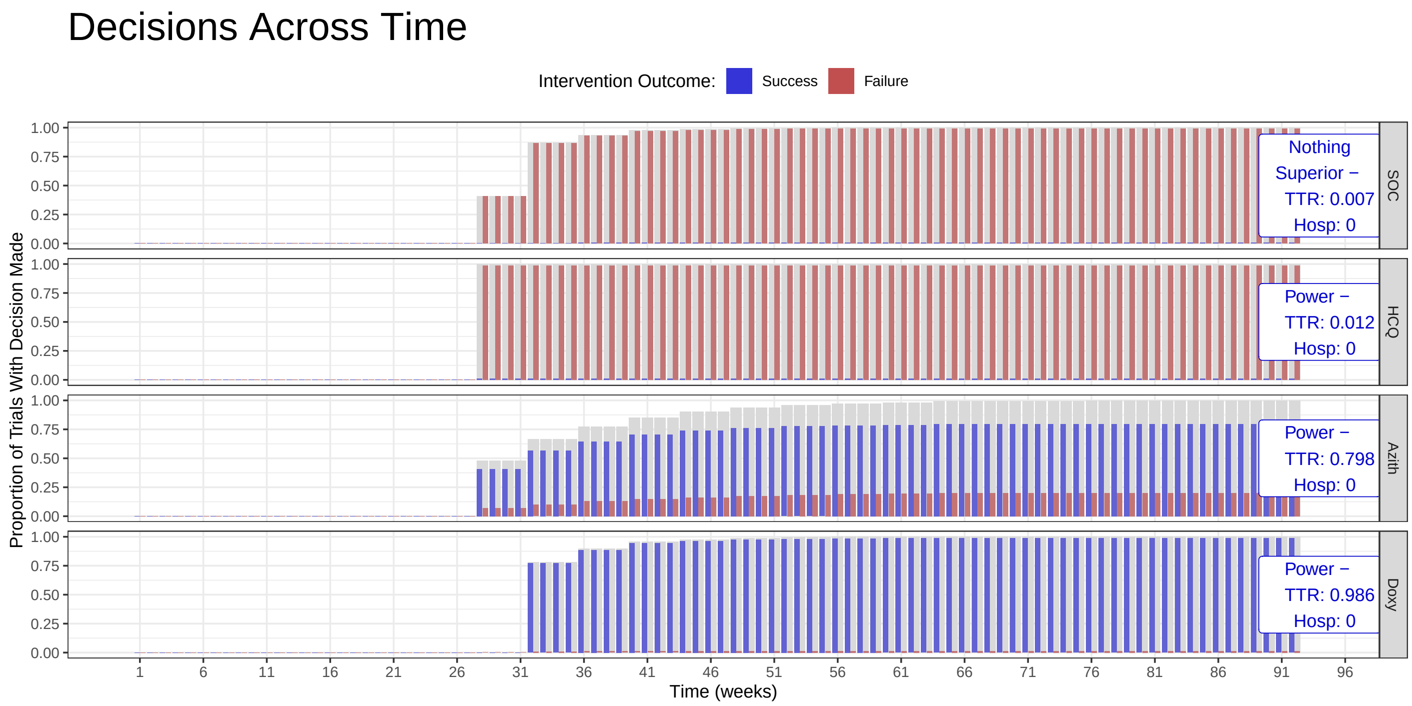

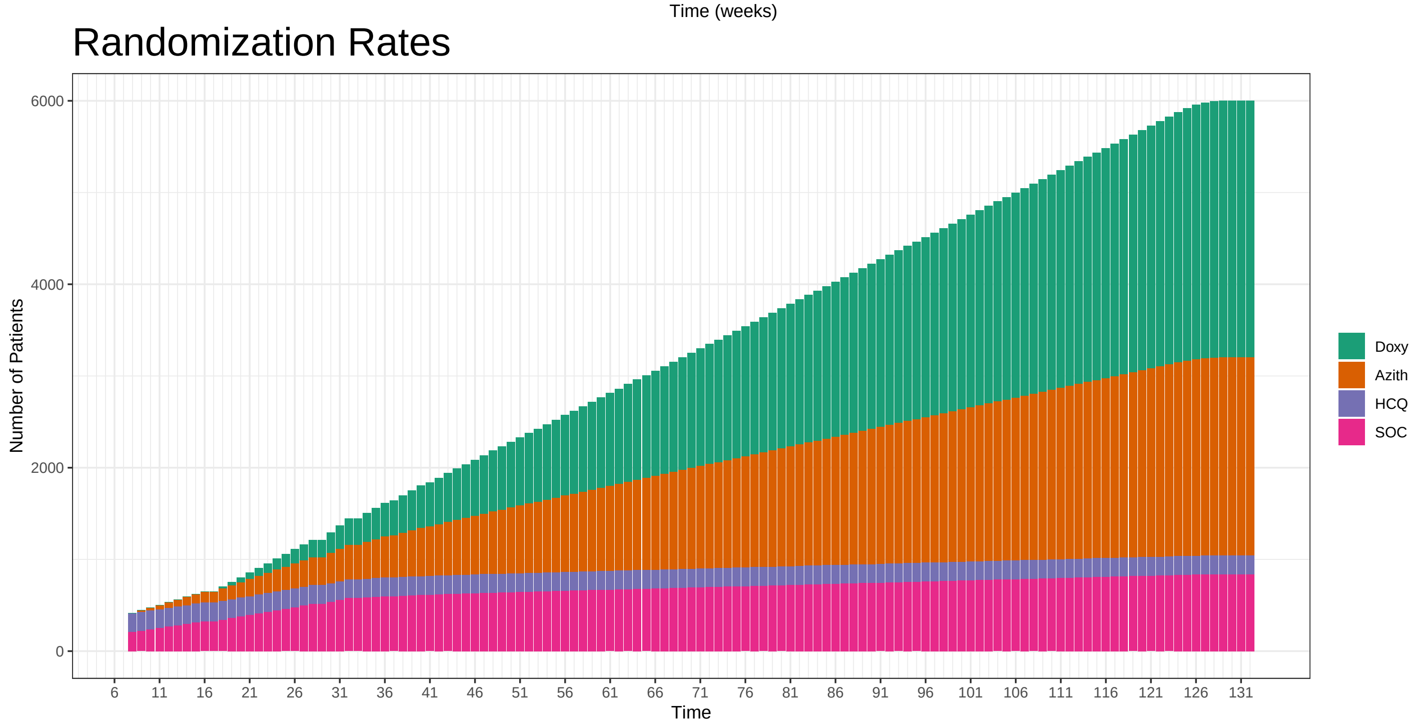

Hazard Ratios: HCQ: 1 Azith: 1.3 Doxy: 1

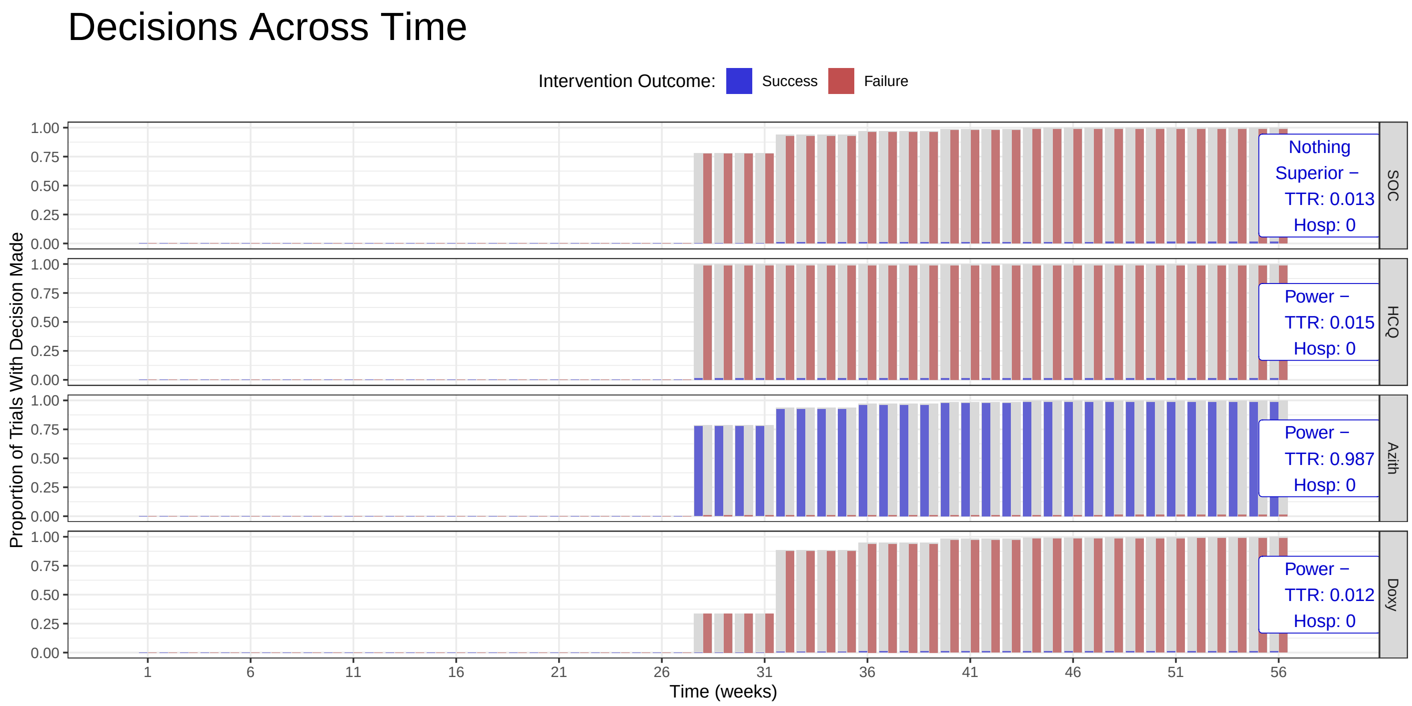

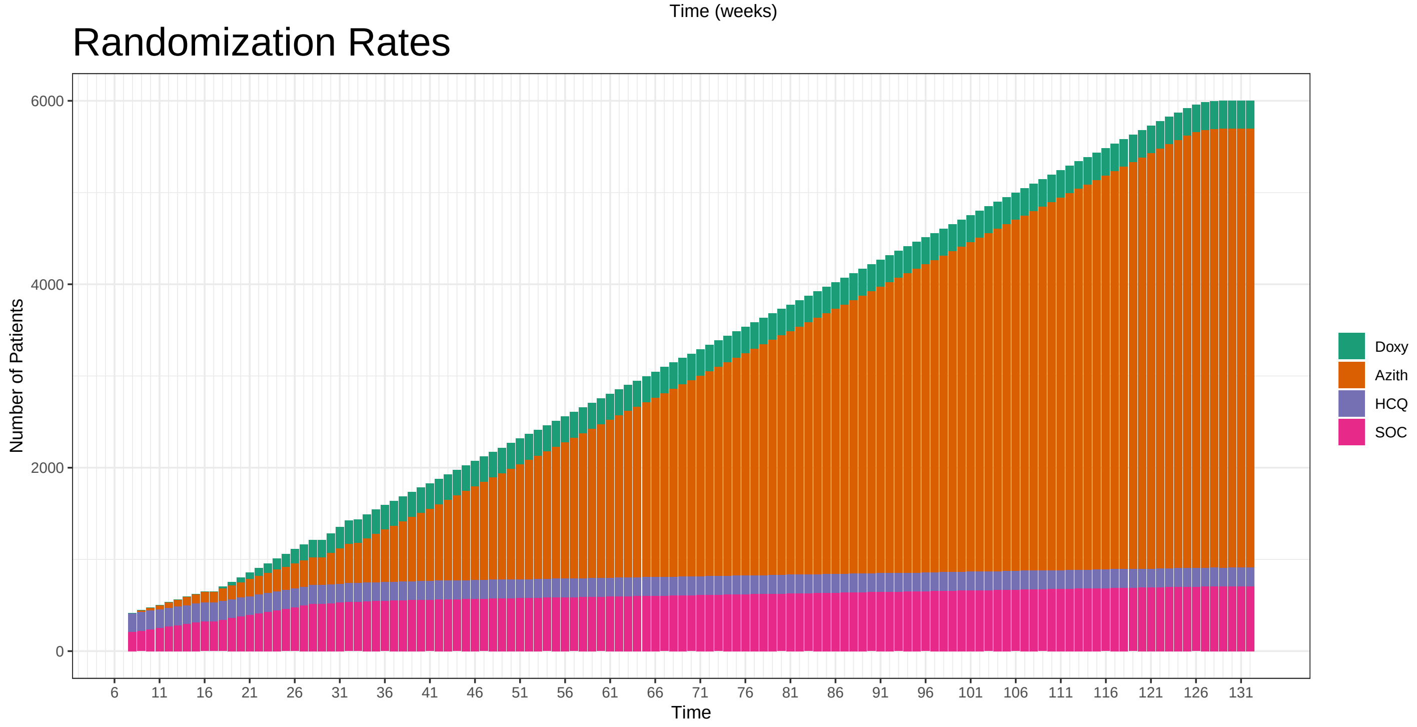

Hazard Ratios: HCQ: 1 Azith: 1.3 Doxy: 1.1

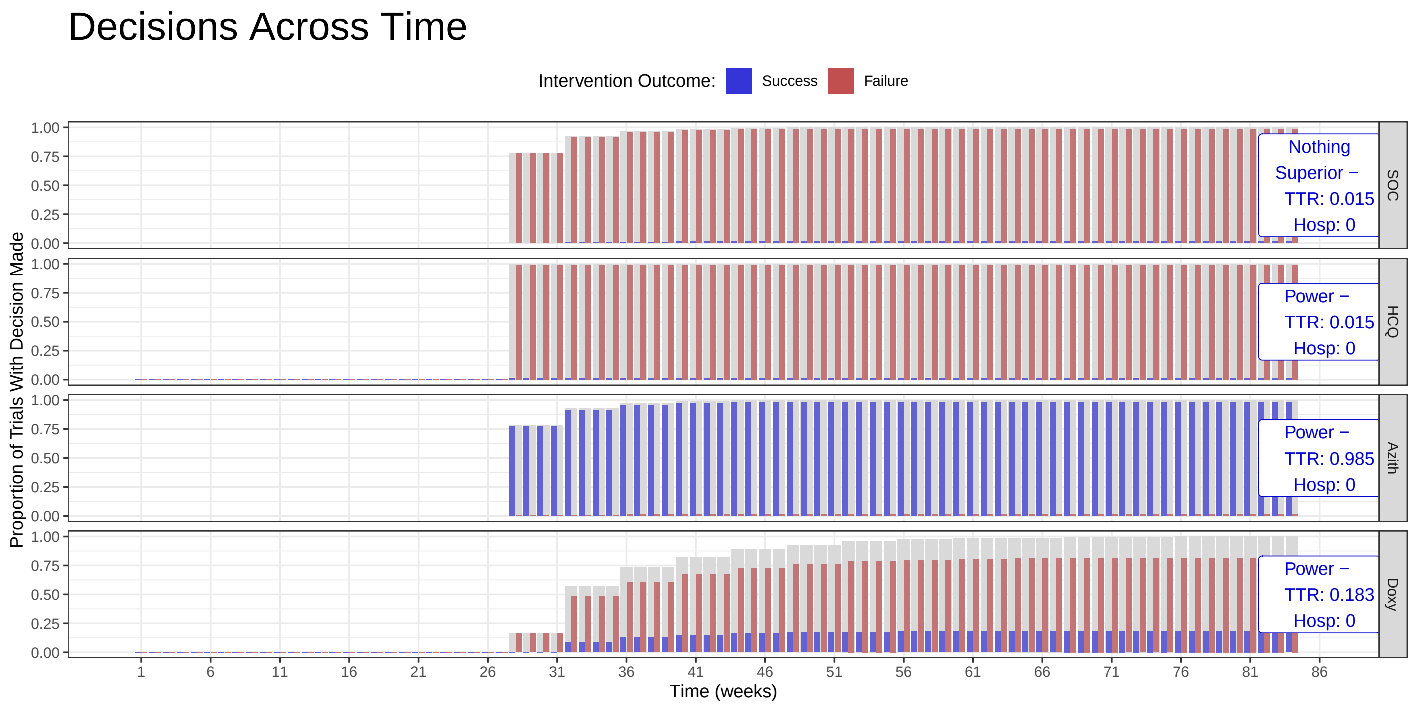

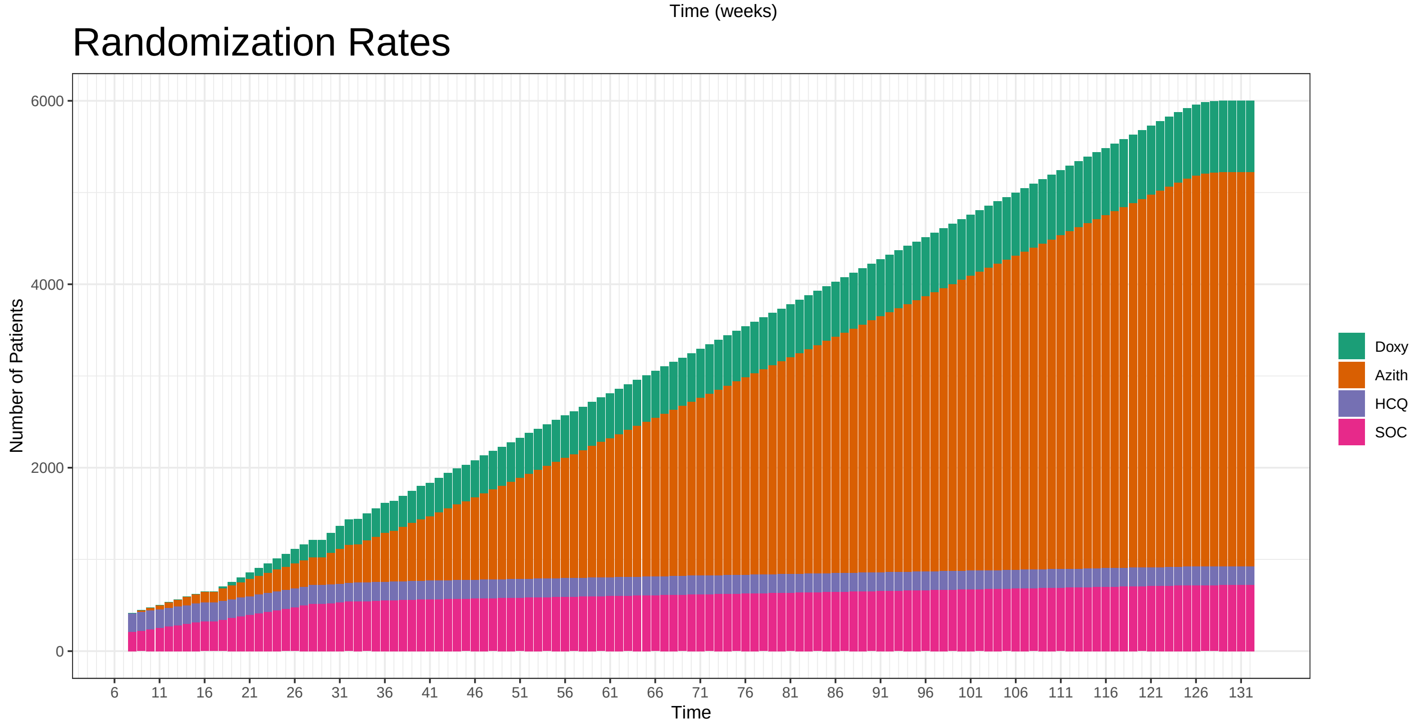

Hazard Ratios: HCQ: 1 Azith: 1.3 Doxy: 1.2

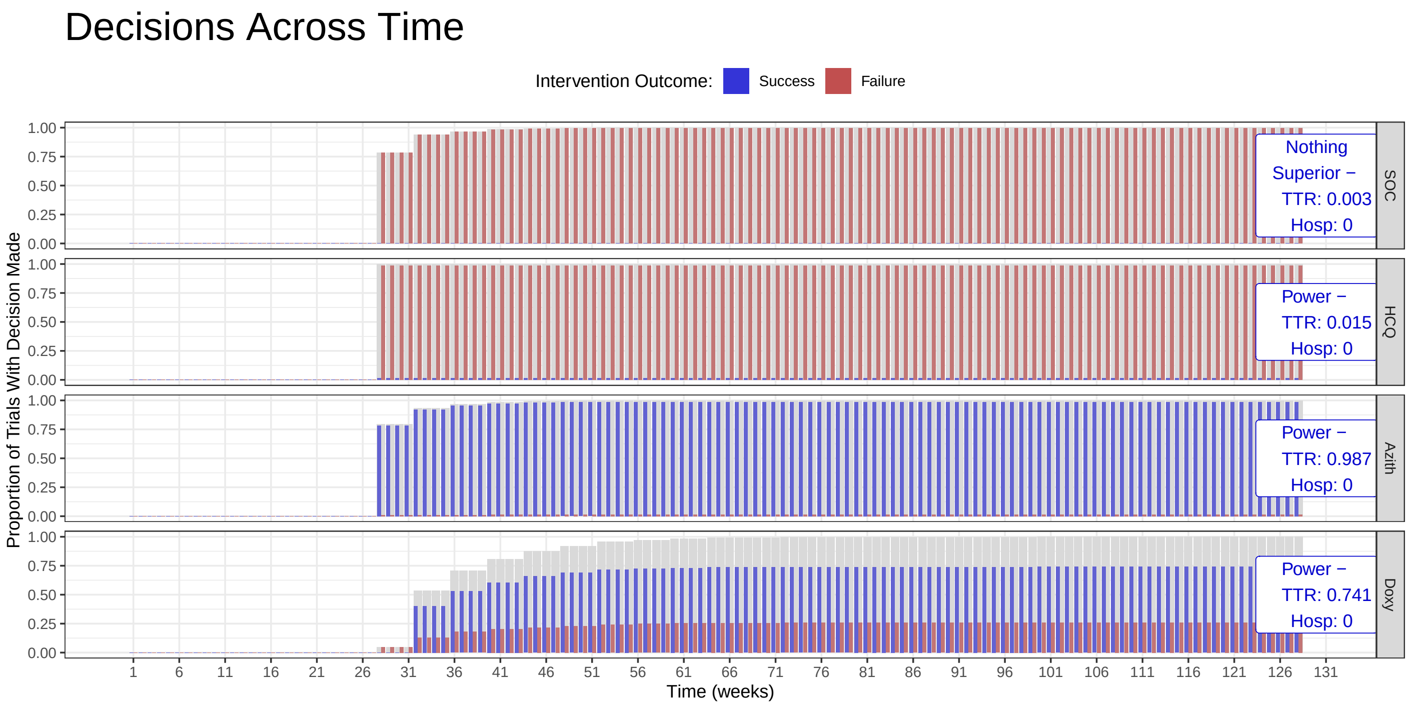

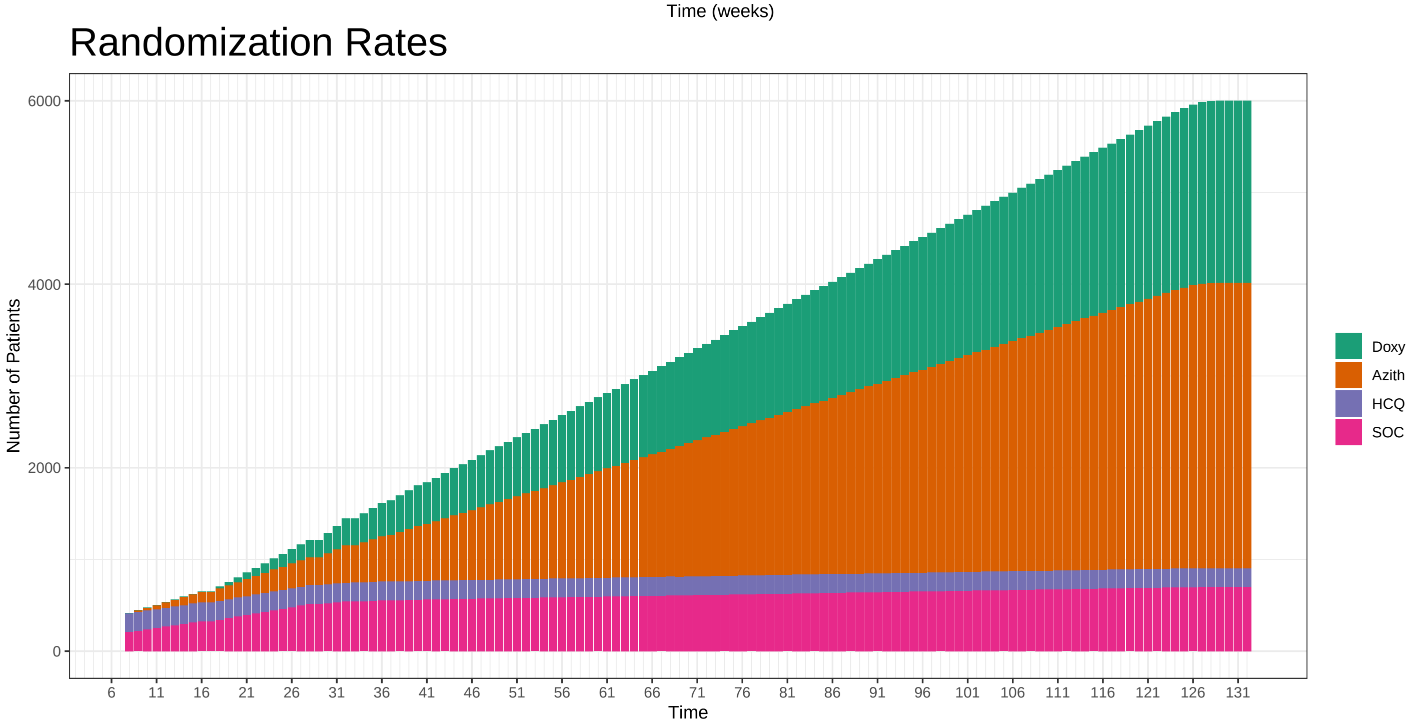

Hazard Ratios: HCQ: 1 Azith: 1.3 Doxy: 1.3

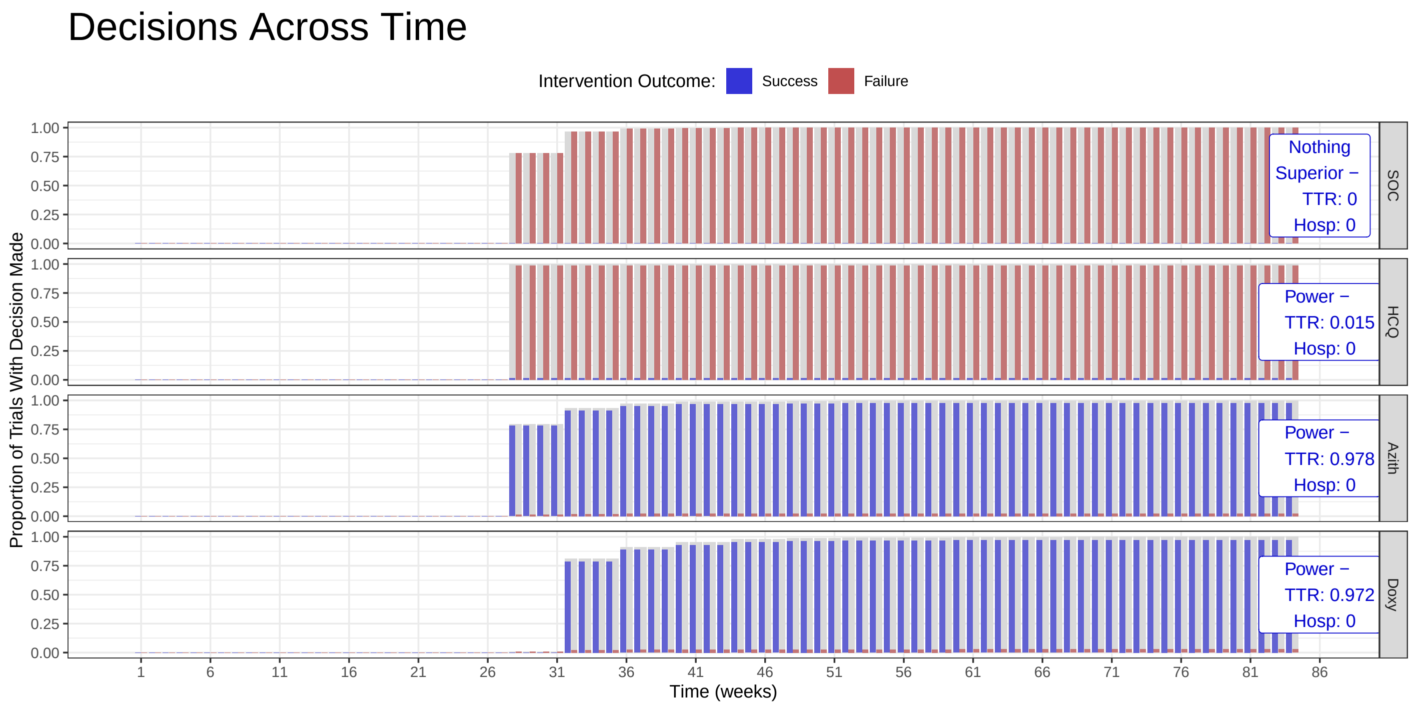

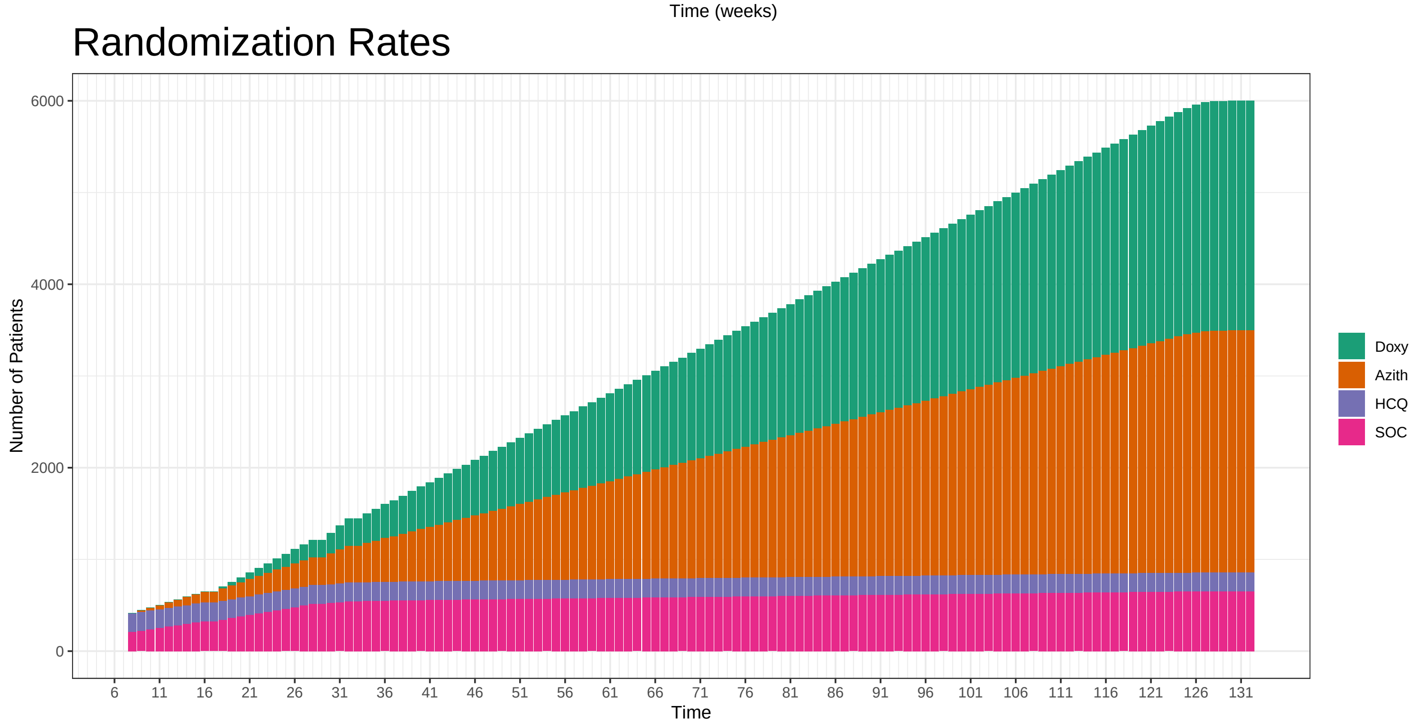

Hazard Ratios: HCQ: 1.1 Azith: 1.1 Doxy: 1.1

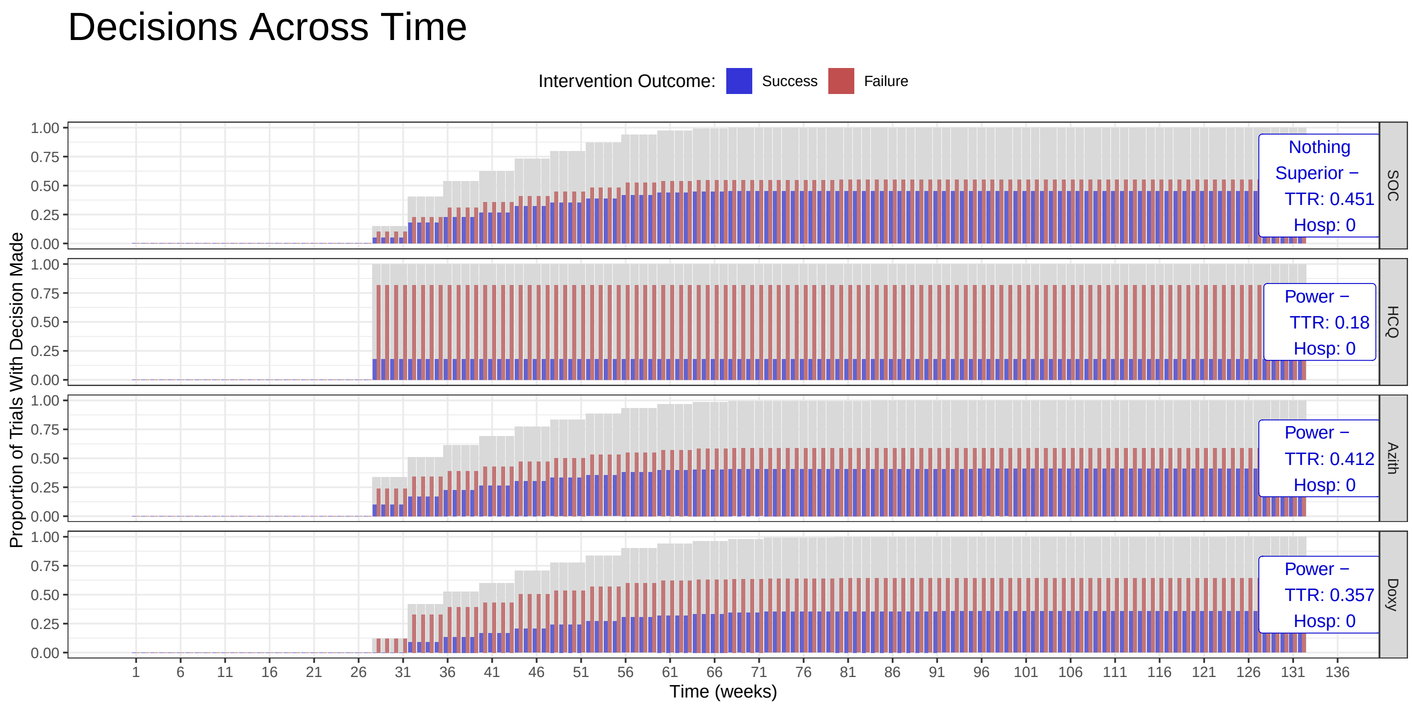

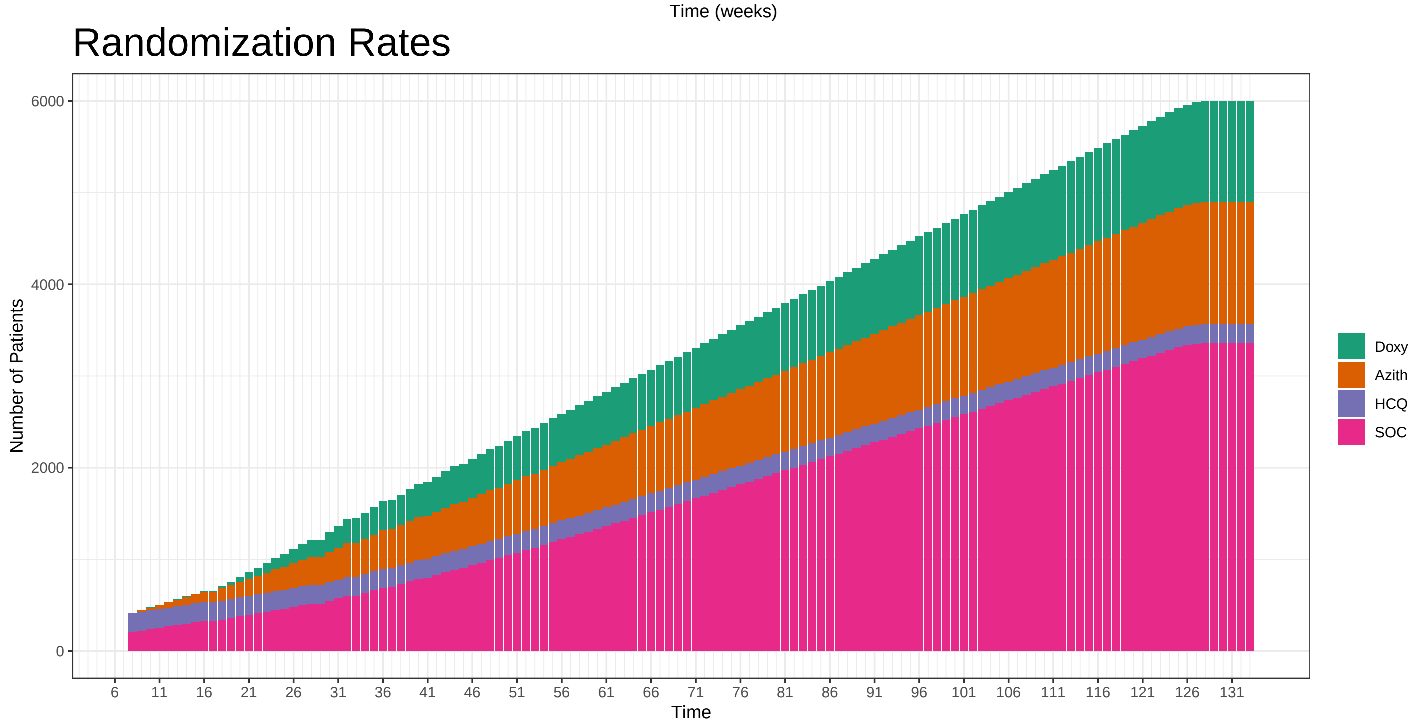

Hazard Ratios: HCQ: 1.2 Azith: 1.2 Doxy: 1.2

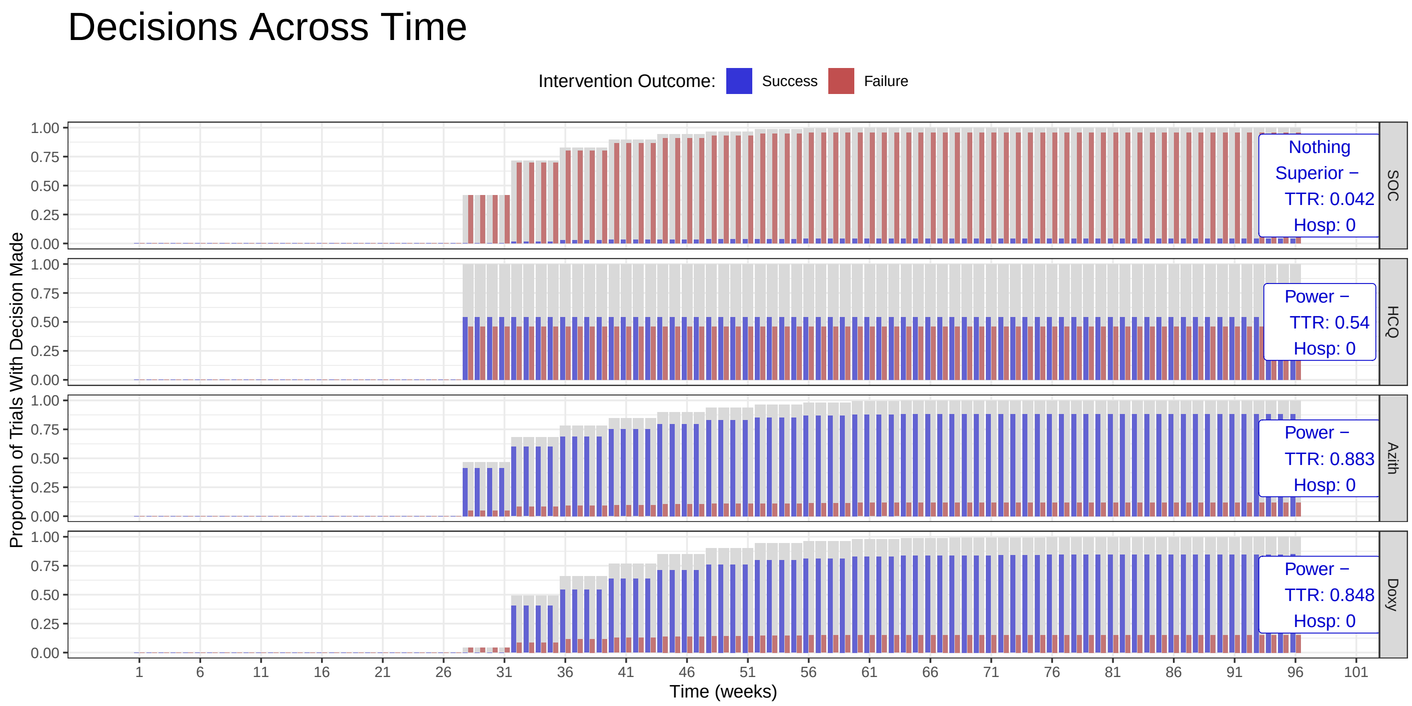

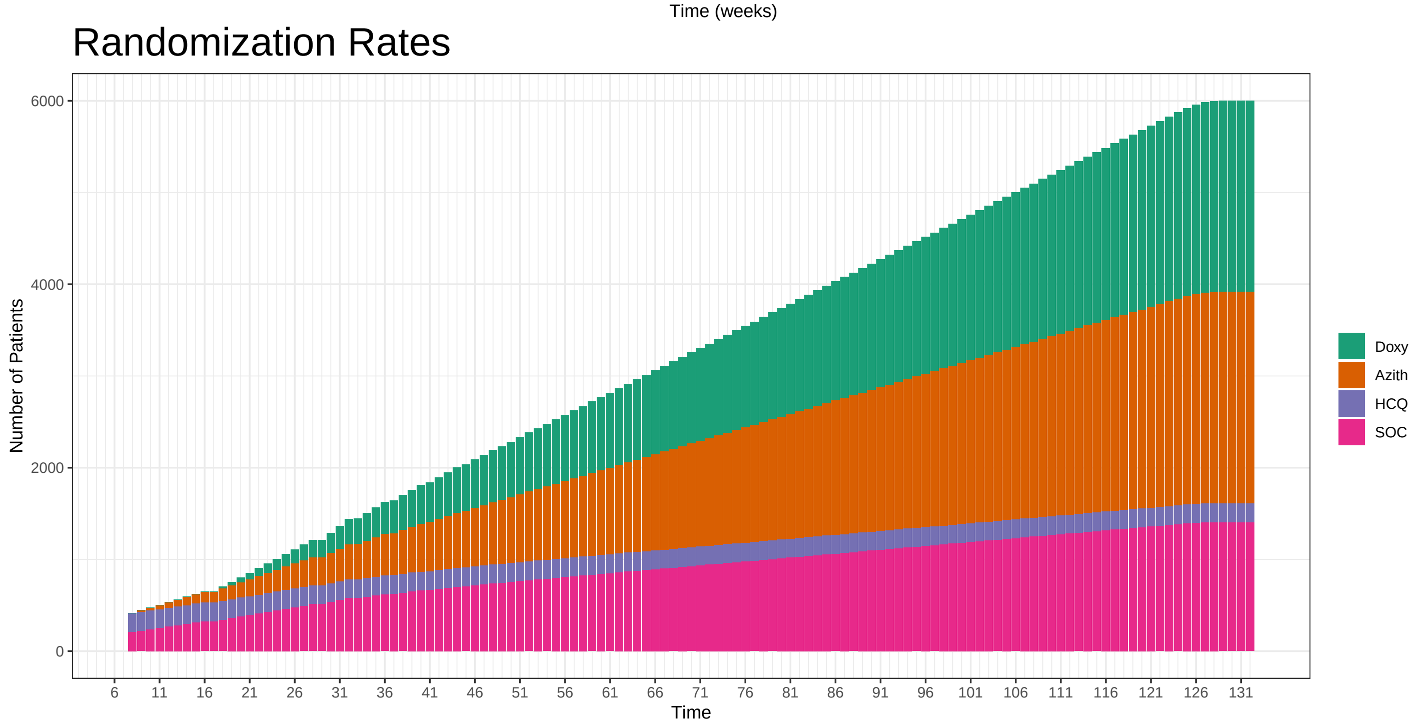

Hazard Ratios: HCQ: 1.3 Azith: 1.3 Doxy: 1.3

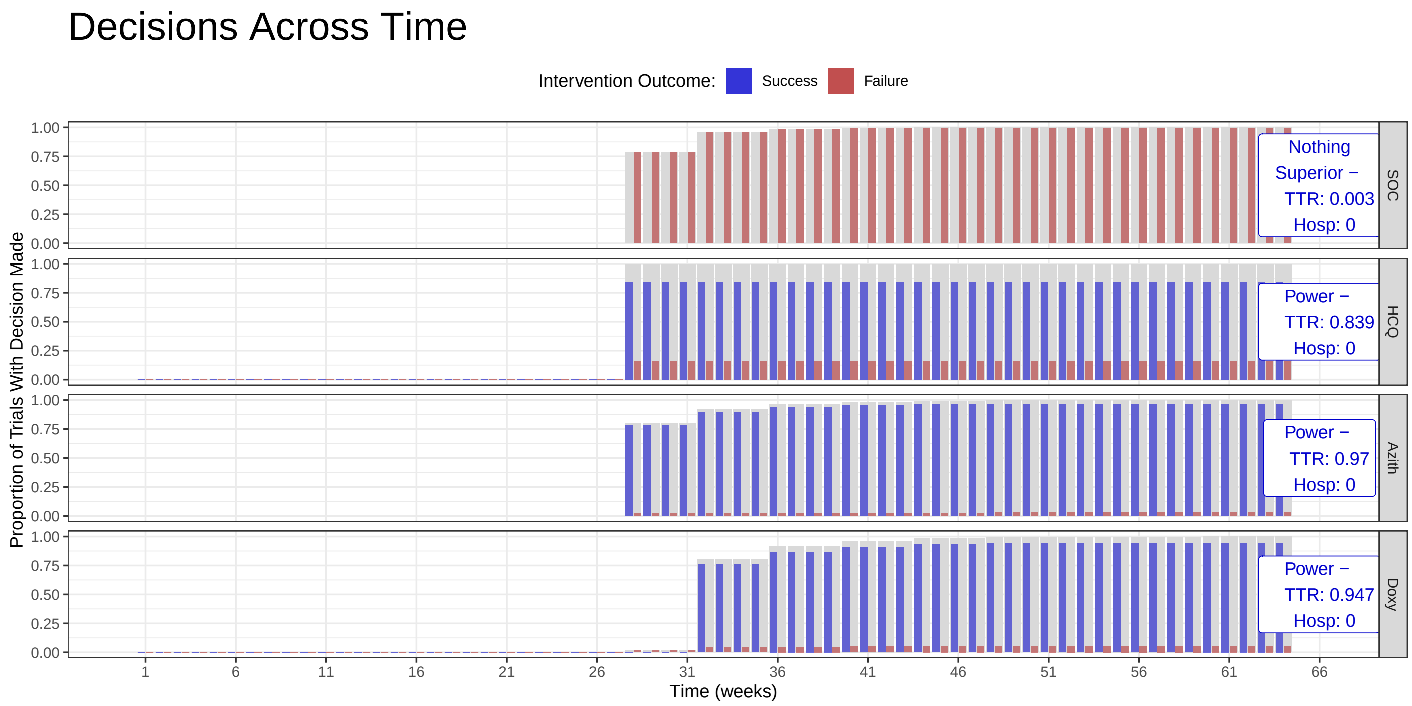

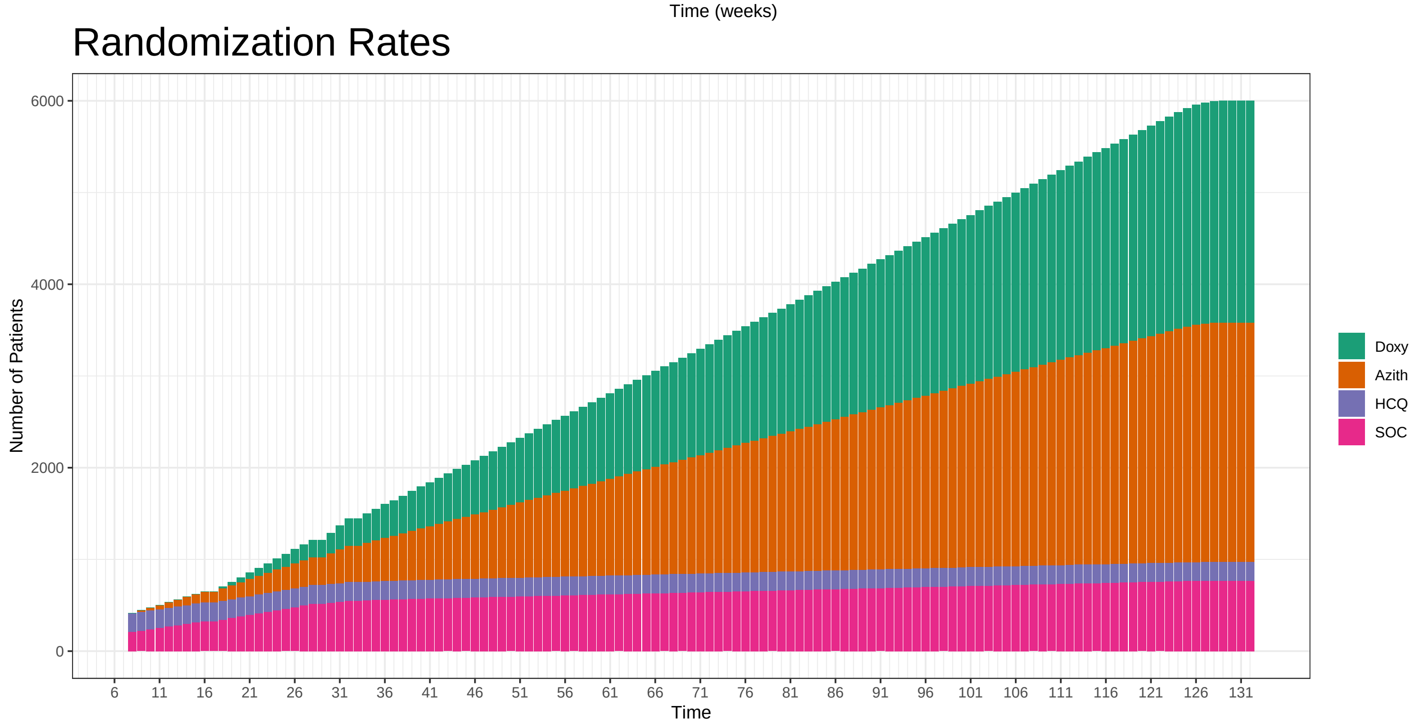

5.3 OCs Additional Scenarios: Null Effect Hospitalization

Principle OCs

Berry Consultants

10/26/2020

These operating characteristics are based on 1000 simulations per scenario across a variety of assumed treatment effects for experimental arms in the trial. The true number of patients randomized per day from April 1, 2020 to October 19, 2020. After this period is over, the simulations assume a 50 patient per week accrual rate. In absence of a definitive rule, these simulations conduct an interim every 28 days. In addition, the true day of enrollment stopping of HCQ is used as well as the days for opening enrollment to the Azithromycin and Doxycycline arms. As such, up to at least October 19, 2020 (day 199) of the trial, the enrollment rates to each arm in these simulations are expected to align closely with the true enrollment rates to the arms in the trial.

Median TTR	Hazard Ratio			Power for TTR				Hospitalization Rate Benefit (%)			Power for TTR & Hosp		
SOC	HCQ	Azith	Doxy	SOC	HCQ	Azith	Doxy	HCQ	Azith	Doxy	HCQ	Azith	Doxy
9	1.1	1.1	1.1	0.416	0.179	0.428	0.398	0	0	0	0.002	0.008	0.012
9	1.2	1.2	1.2	0.035	0.540	0.879	0.857	0	0	0	0.008	0.019	0.016
9	1.3	1.3	1.3	0.002	0.839	0.975	0.959	0	0	0	0.012	0.019	0.029

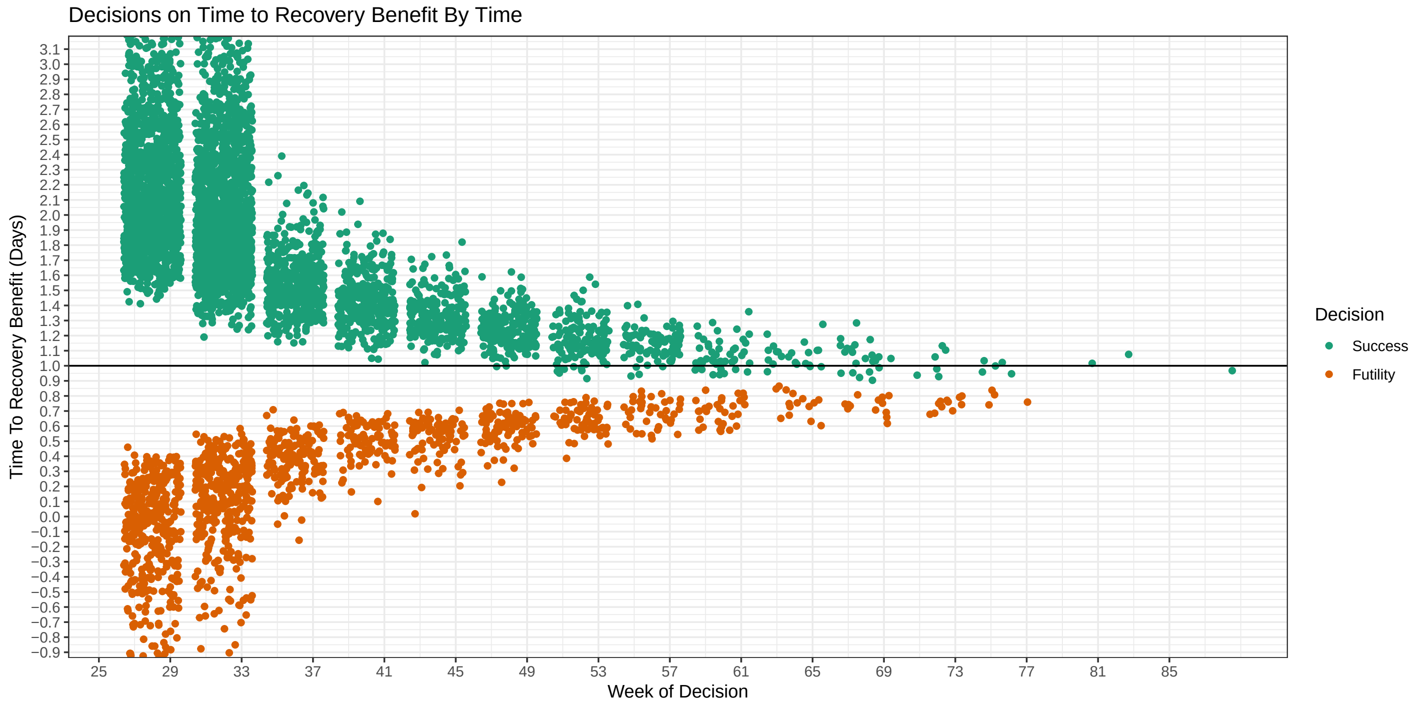

Decisions on Time to Recovery Benefit By Time
Time To Recovery Benefit (Days)
3.1
3.0
2.9
2.8
2.7
2.6
2.5
2.4
2.3
2.2
2.1
2.0
1.9
1.8
1.7
1.6
1.5
1.4
1.3
1.2
1.1
1.0
0.9
0.8
0.7
0.6
0.5
0.4
0.3
0.2
0.1
0.0
−0.1
−0.2
−0.3
−0.4
−0.5
−0.6
−0.7
−0.8
−0.9
25
29
33
37
41
45
49
53
57
61
65
69
73
77
81
85
Week of Decision
Decision
Success
Futility

Hazard Ratios: HCQ: 1.1 Azith: 1.1 Doxy: 1.1

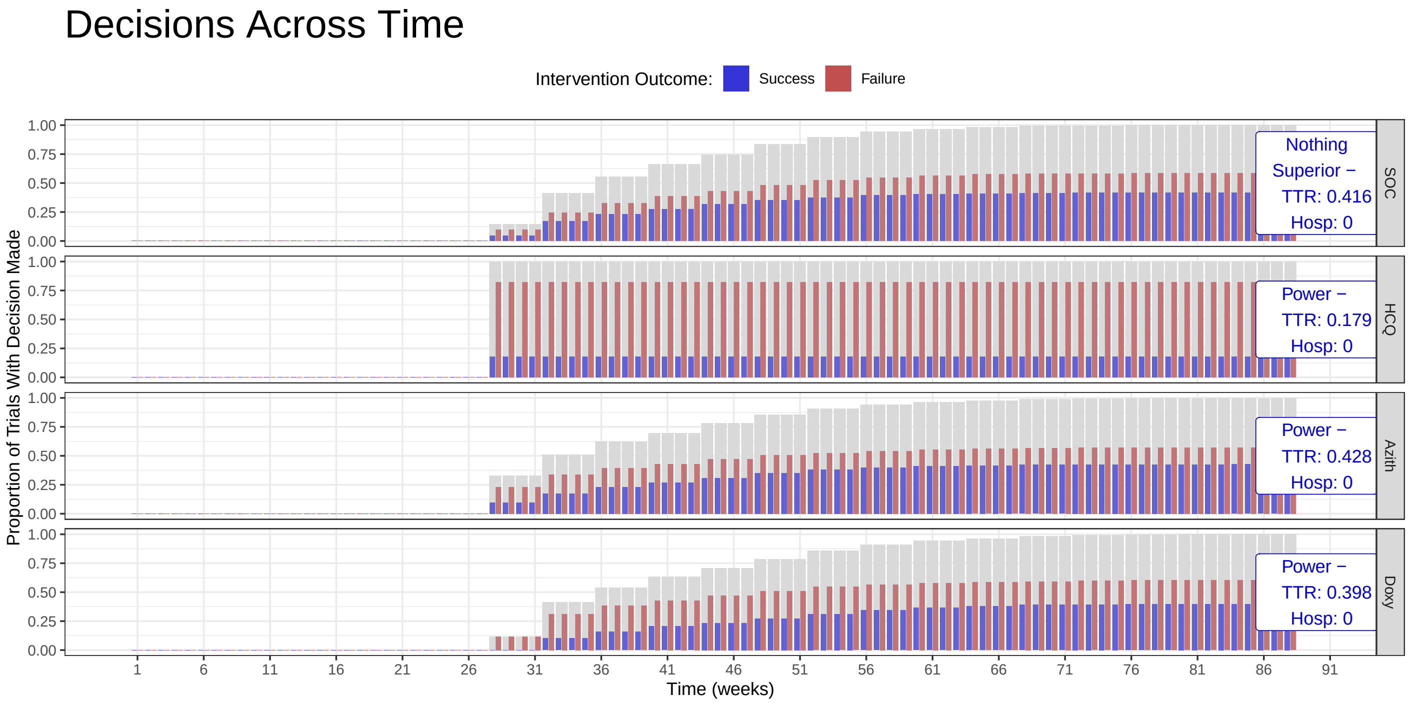

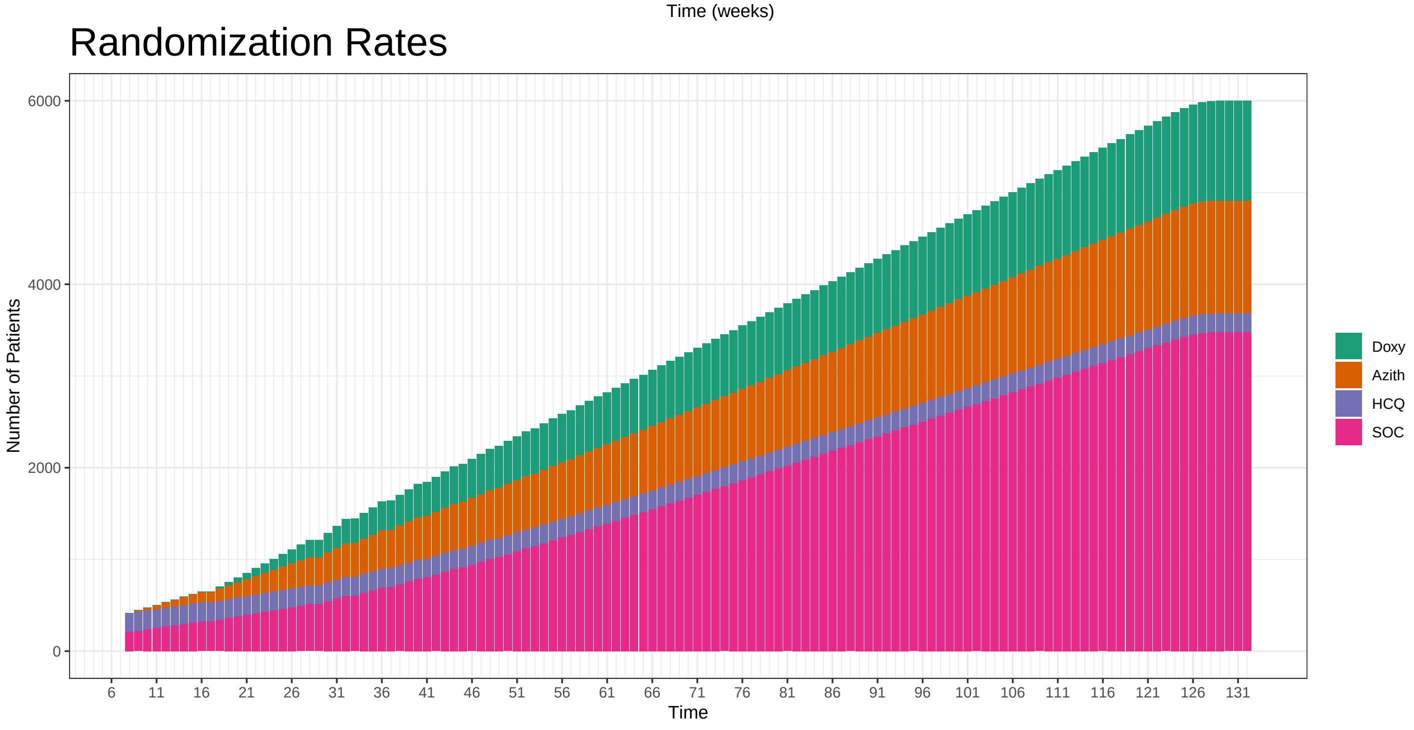

Hazard Ratios: HCQ: 1.2 Azith: 1.2 Doxy: 1.2

Decisions Across Time

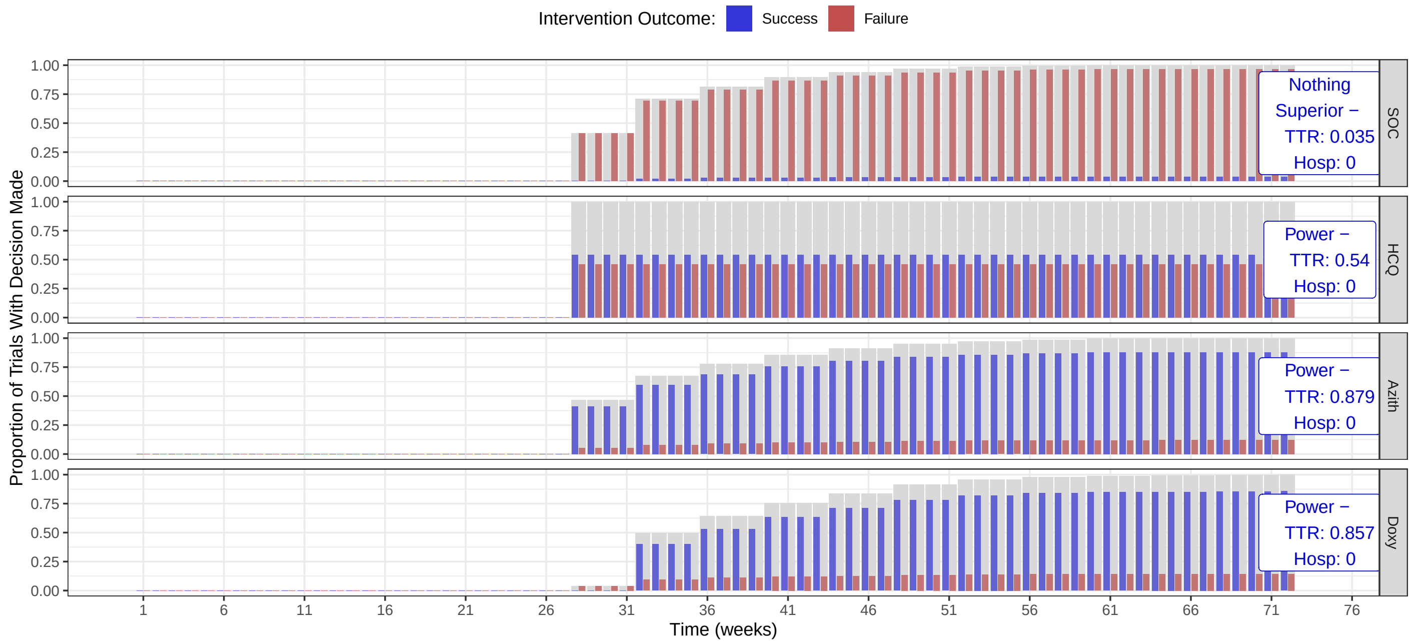

Randomization Rates

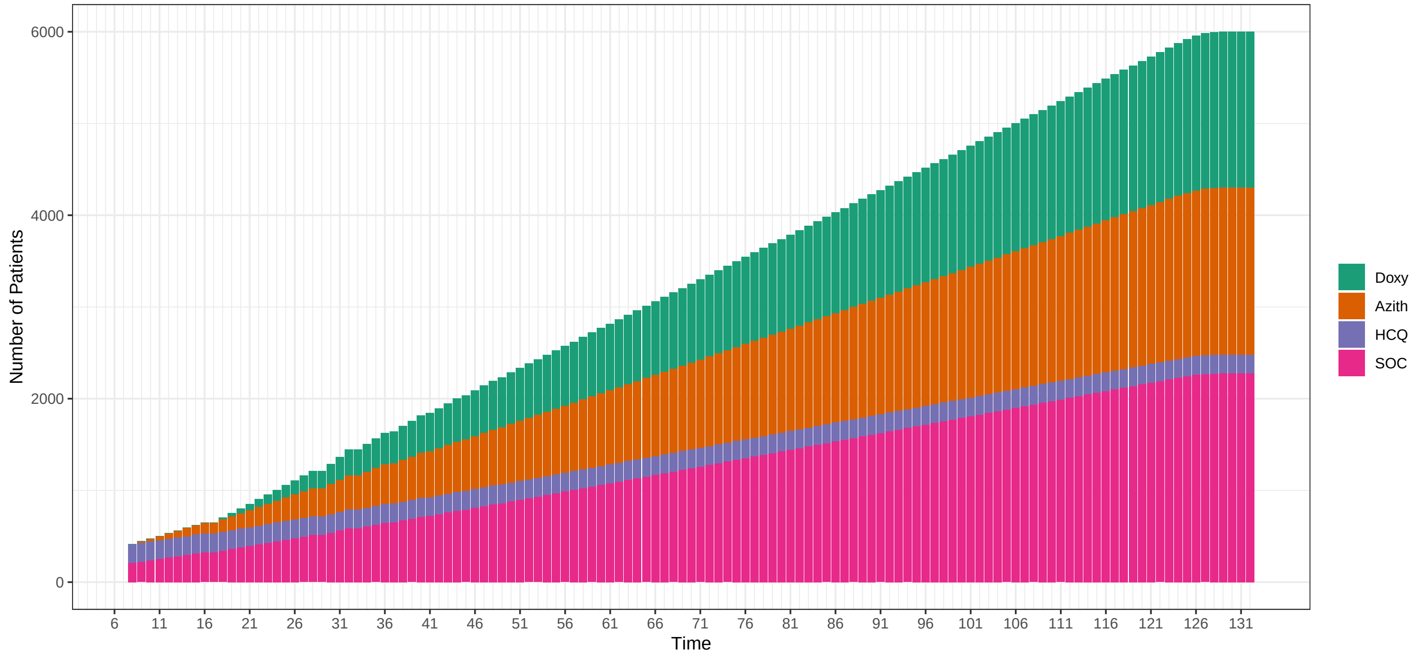

Hazard Ratios: HCQ: 1.3 Azith: 1.3 Doxy: 1.3

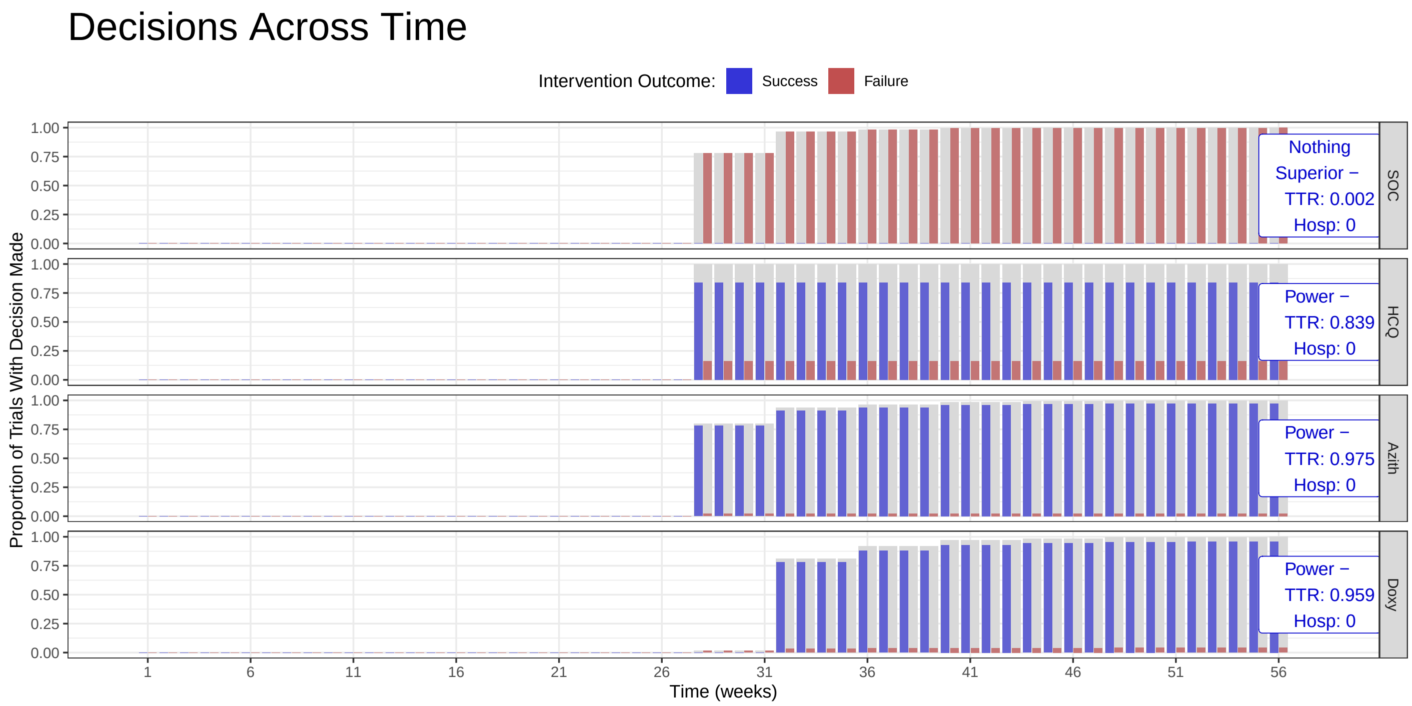

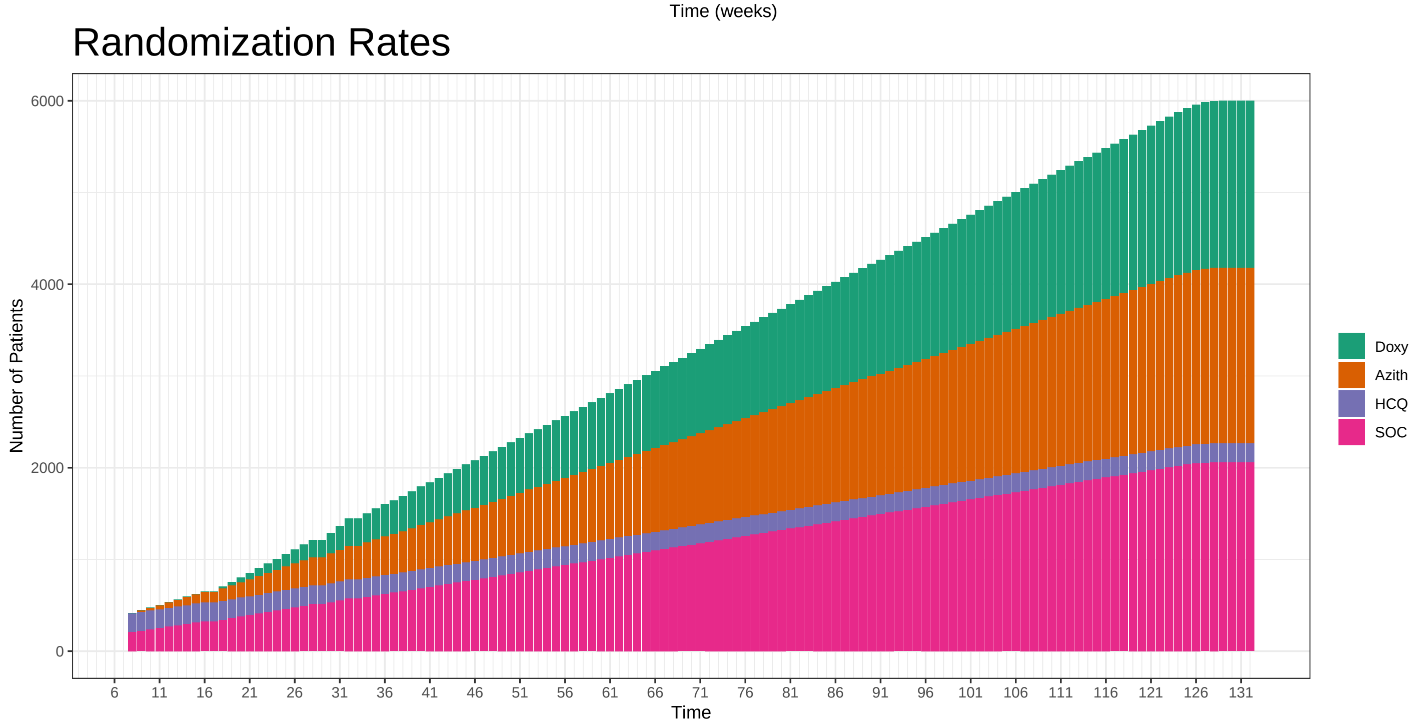

5.4 OCs Additional Scenarios: Modified Time to Recovery Rate for Usual Care Arm

Principle OCs

Berry Consultants

10/26/2020

These operating characteristics are based on 1000 simulations per scenario across a variety of assumed treatment effects for experimental arms in the trial. The true number of patients randomized per day from April 1, 2020 to October 19, 2020. After this period is over, the simulations assume a 50 patient per week accrual rate. In absence of a definitive rule, these simulations conduct an interim every 28 days. In addition, the true day of enrollment stopping of HCQ is used as well as the days for opening enrollment to the Azithromycin and Doxycycline arms. As such, up to at least October 19, 2020 (day 199) of the trial, the enrollment rates to each arm in these simulations are expected to align closely with the true enrollment rates to the arms in the trial.

Median TTR	Hazard Ratio			Power for TTR				Hospitalization Rate Benefit (%)			Power for TTR & Hosp		
SOC	HCQ	Azith	Doxy	SOC	HCQ	Azith	Doxy	HCQ	Azith	Doxy	HCQ	Azith	Doxy
7	1	1	1.0	0.979	0.009	0.012	0.010	0	0	0	0	0	0.000
7	1	1	1.1	0.768	0.009	0.019	0.221	0	0	1	0	0	0.033
7	1	1	1.2	0.317	0.011	0.010	0.683	0	0	2	0	0	0.343
7	1	1	1.3	0.059	0.007	0.007	0.941	0	0	3	0	0	0.905
11	1	1	1.0	0.940	0.017	0.036	0.030	0	0	0	0	0	0.000
11	1	1	1.1	0.467	0.015	0.038	0.522	0	0	1	0	0	0.105
11	1	1	1.2	0.057	0.015	0.014	0.943	0	0	2	0	0	0.578
11	1	1	1.3	0.003	0.015	0.013	0.997	0	0	3	0	0	0.963

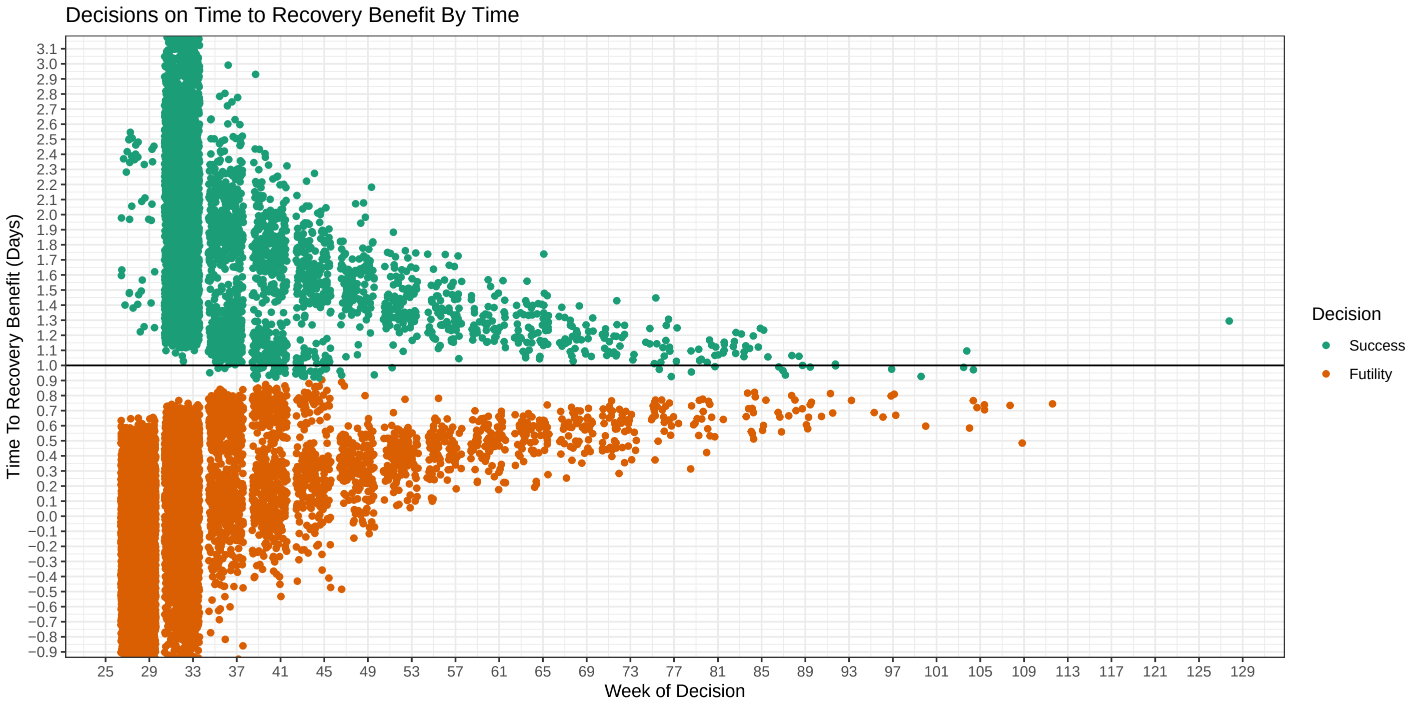

Hazard Ratios: HCQ: 1 Azith: 1 Doxy: 1

Decisions Across Time

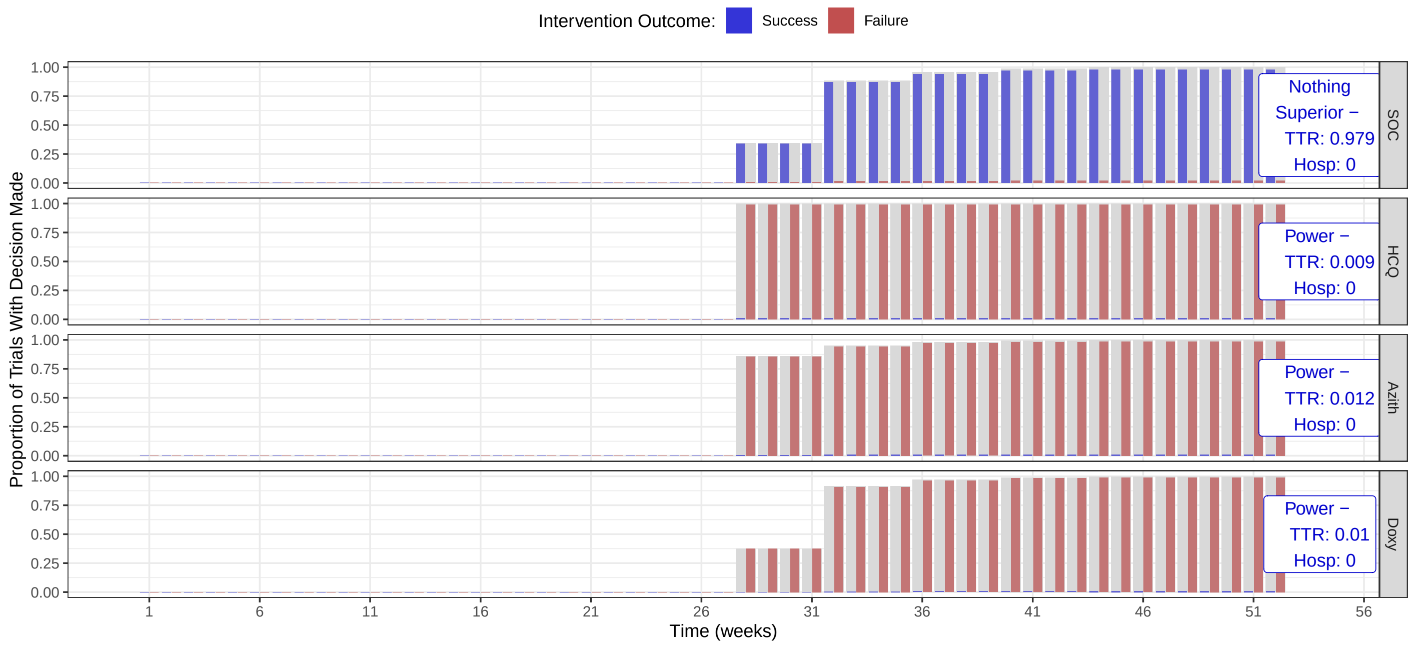

Randomization Rates

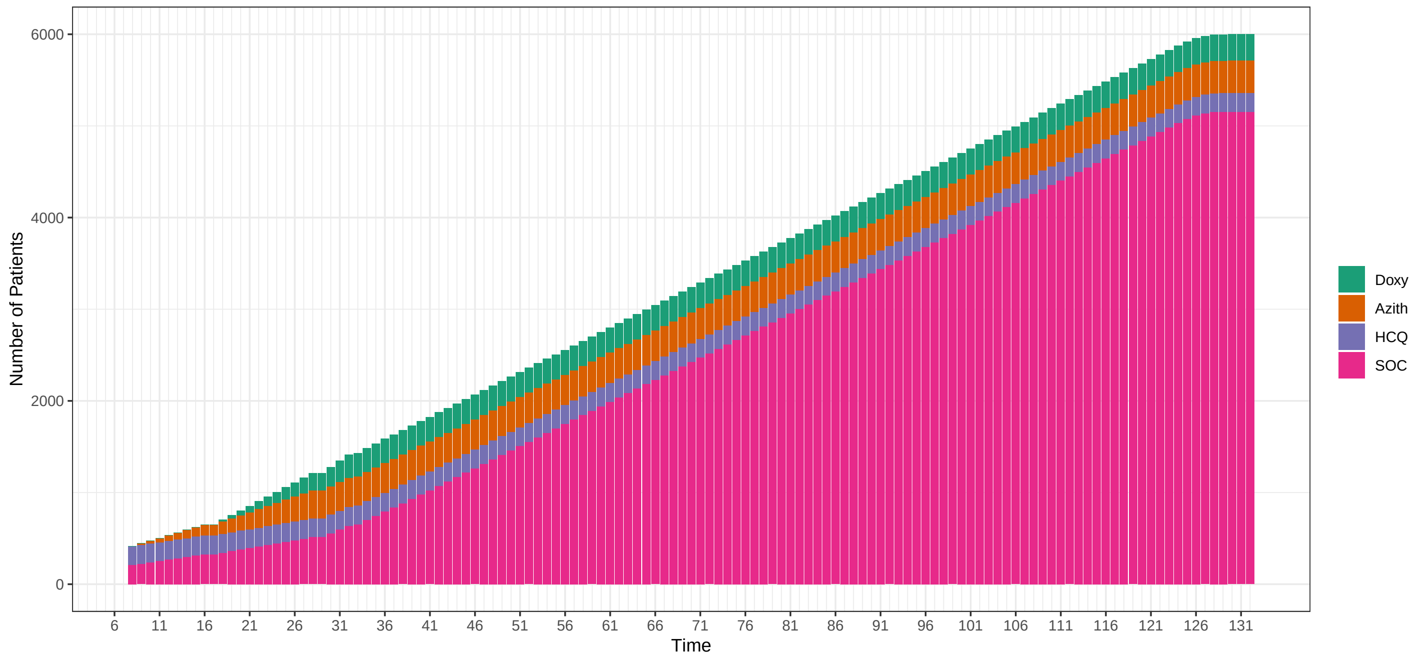

Hazard Ratios: HCQ: 1 Azith: 1 Doxy: 1.1

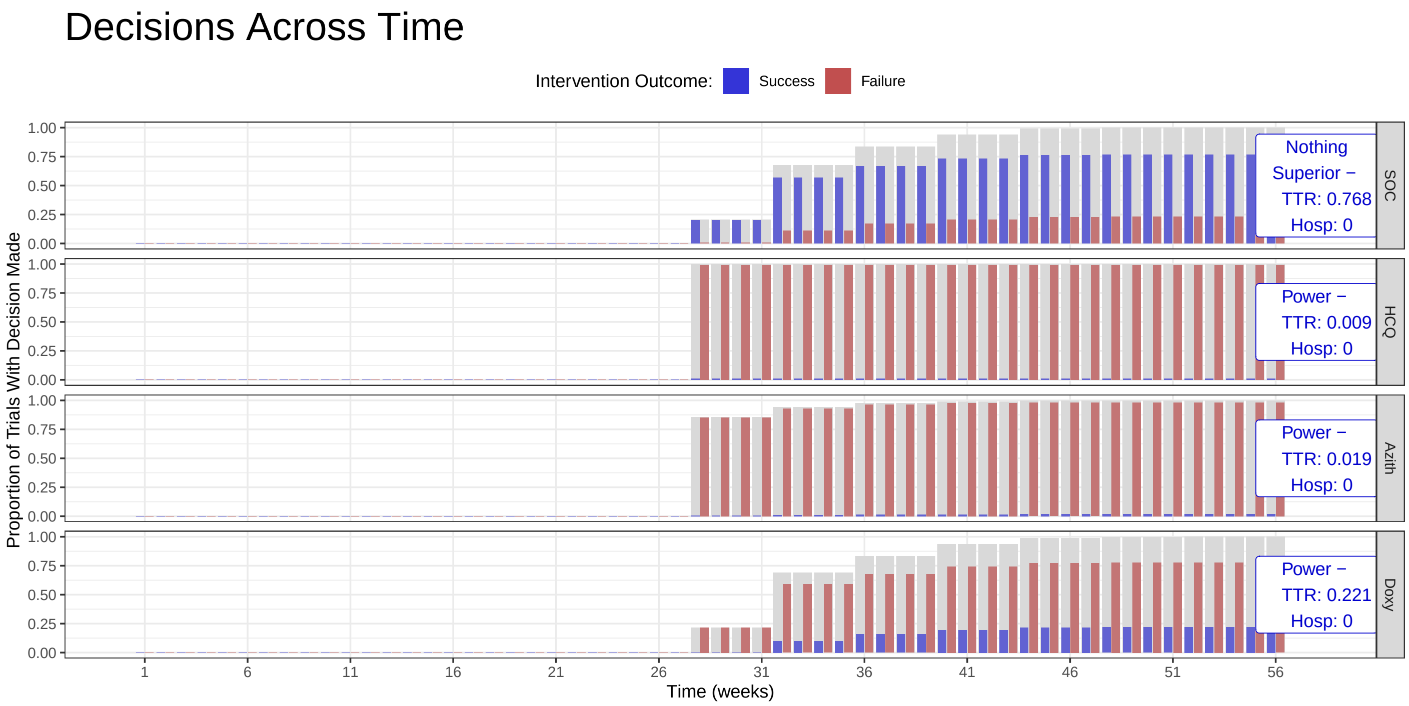

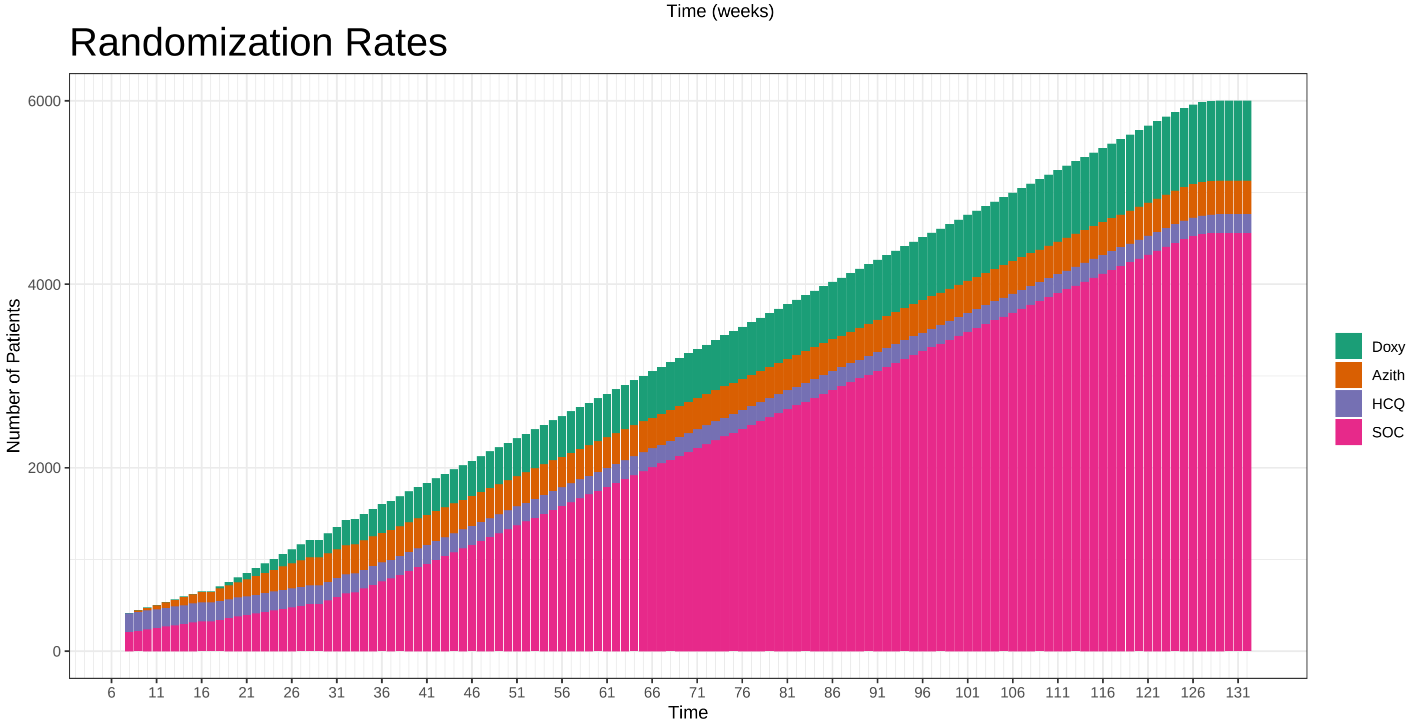

Hazard Ratios: HCQ: 1 Azith: 1 Doxy: 1.2

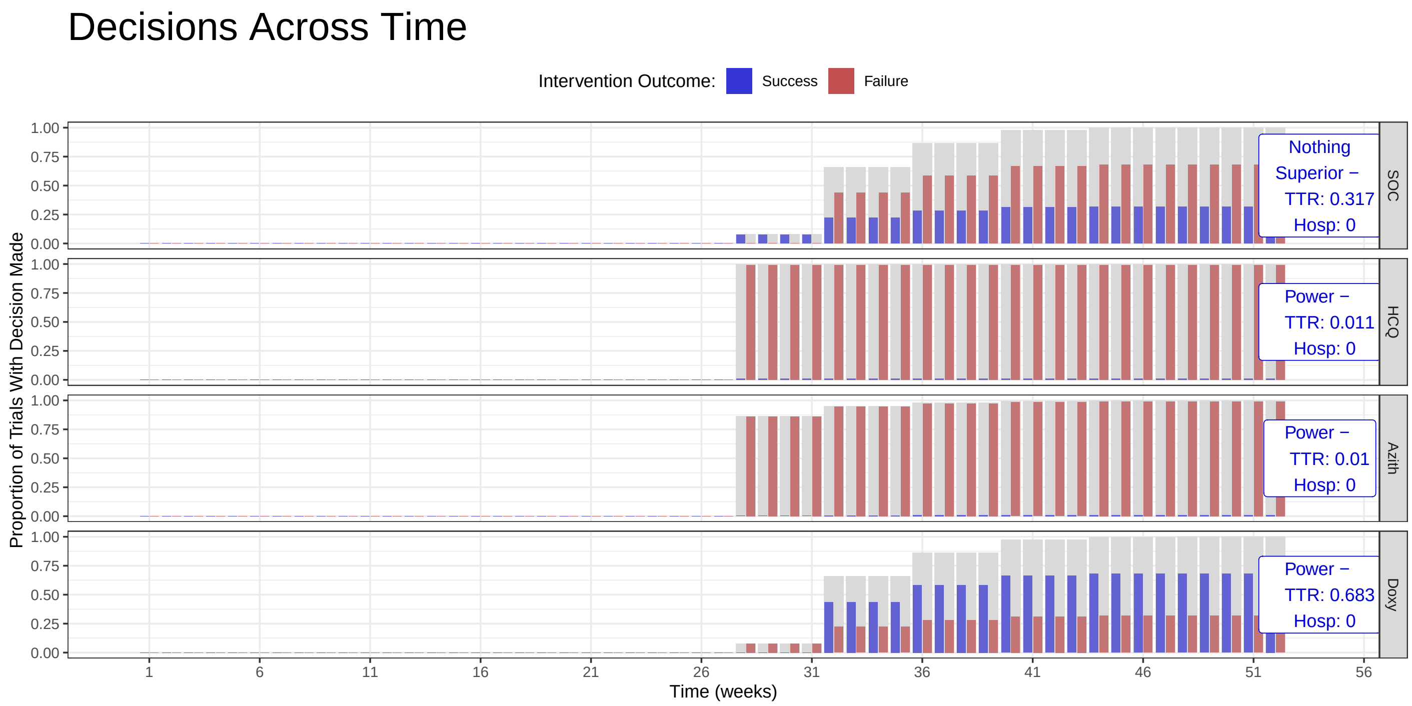

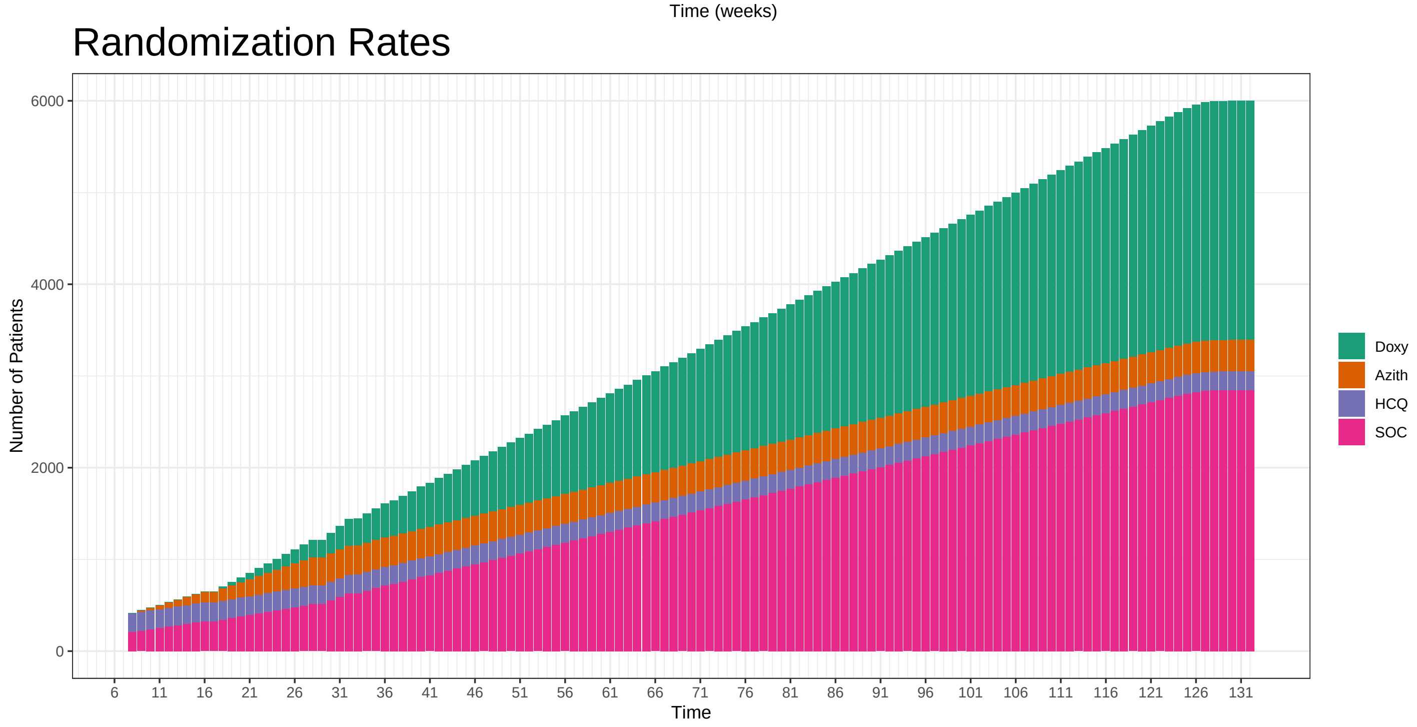

Hazard Ratios: HCQ: 1 Azith: 1 Doxy: 1.3

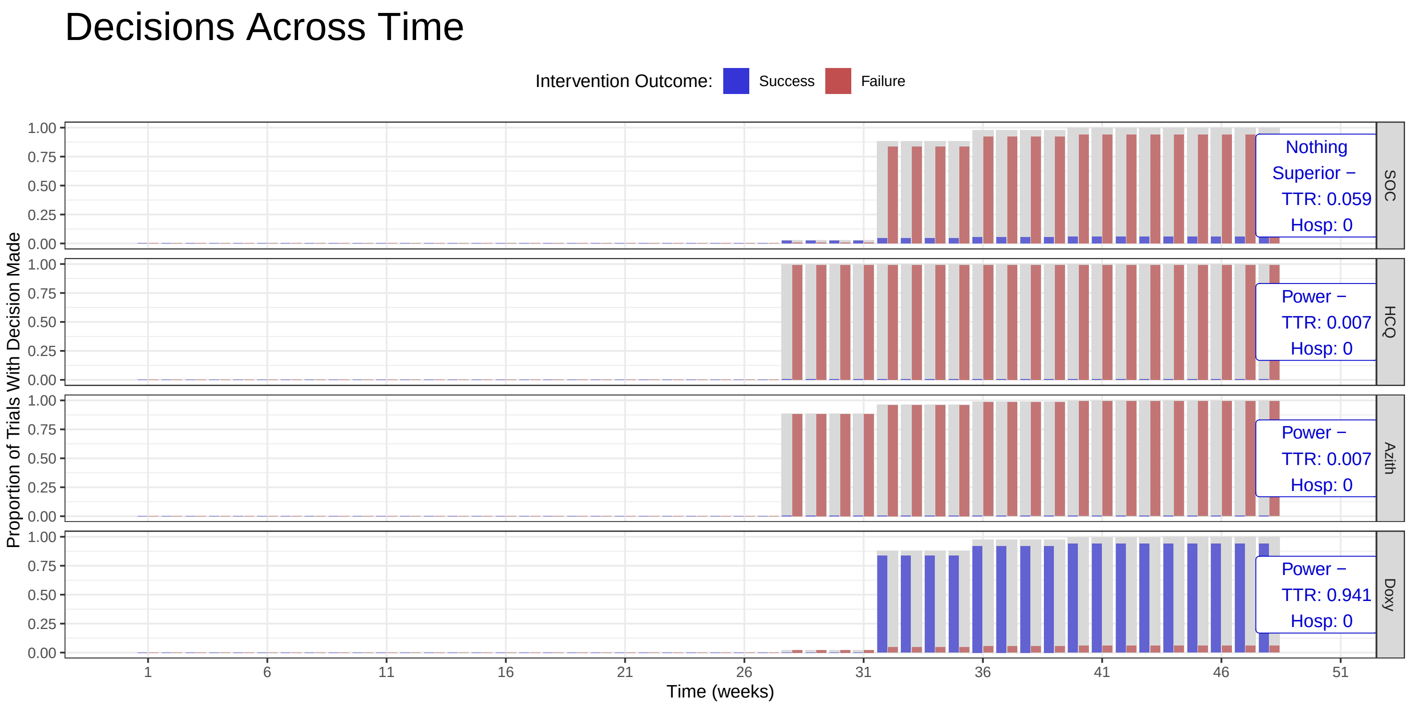

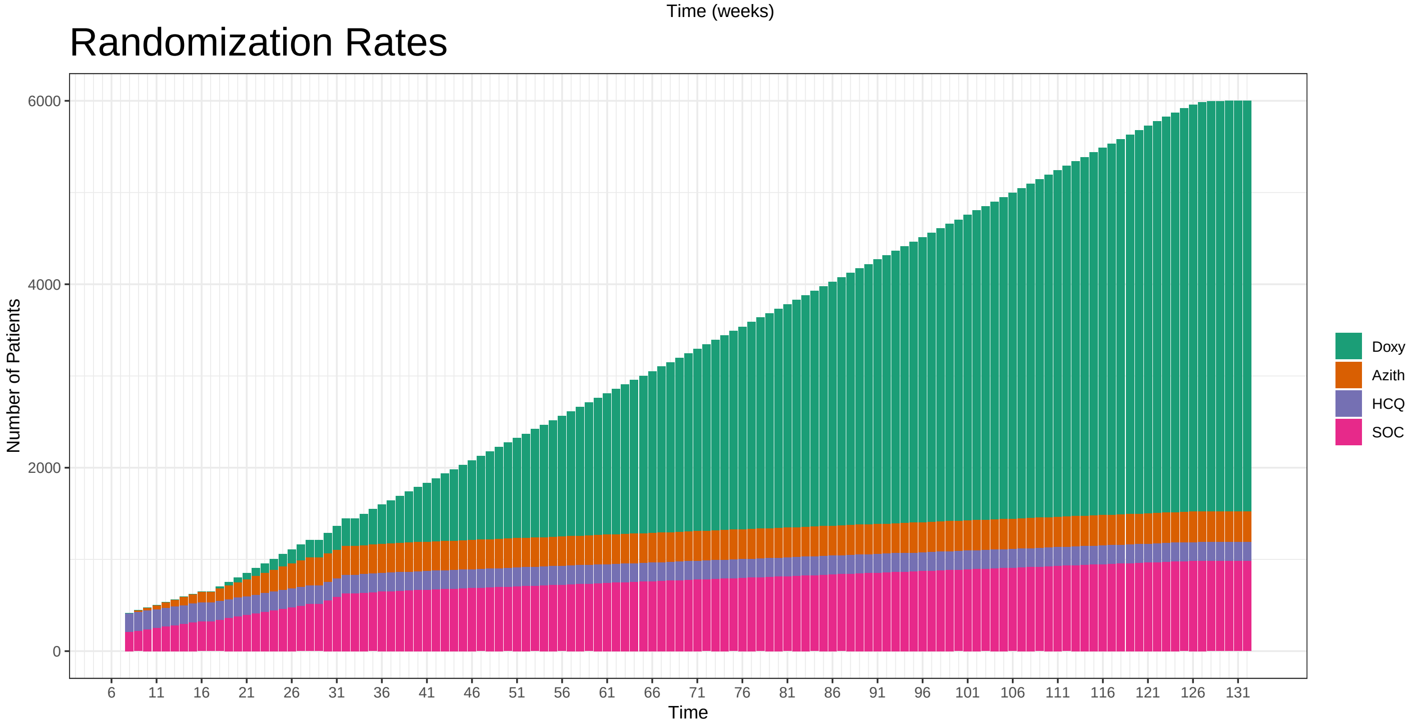

Hazard Ratios: HCQ: 1 Azith: 1 Doxy: 1

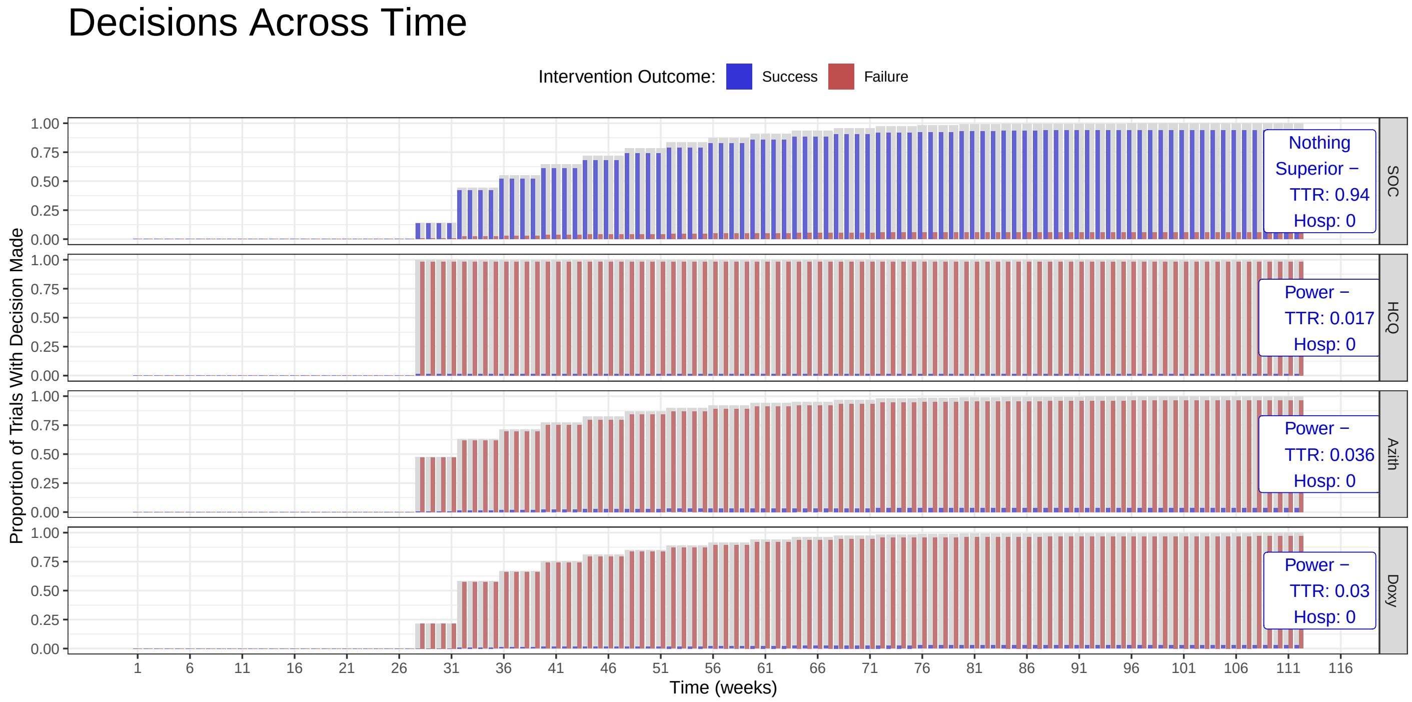

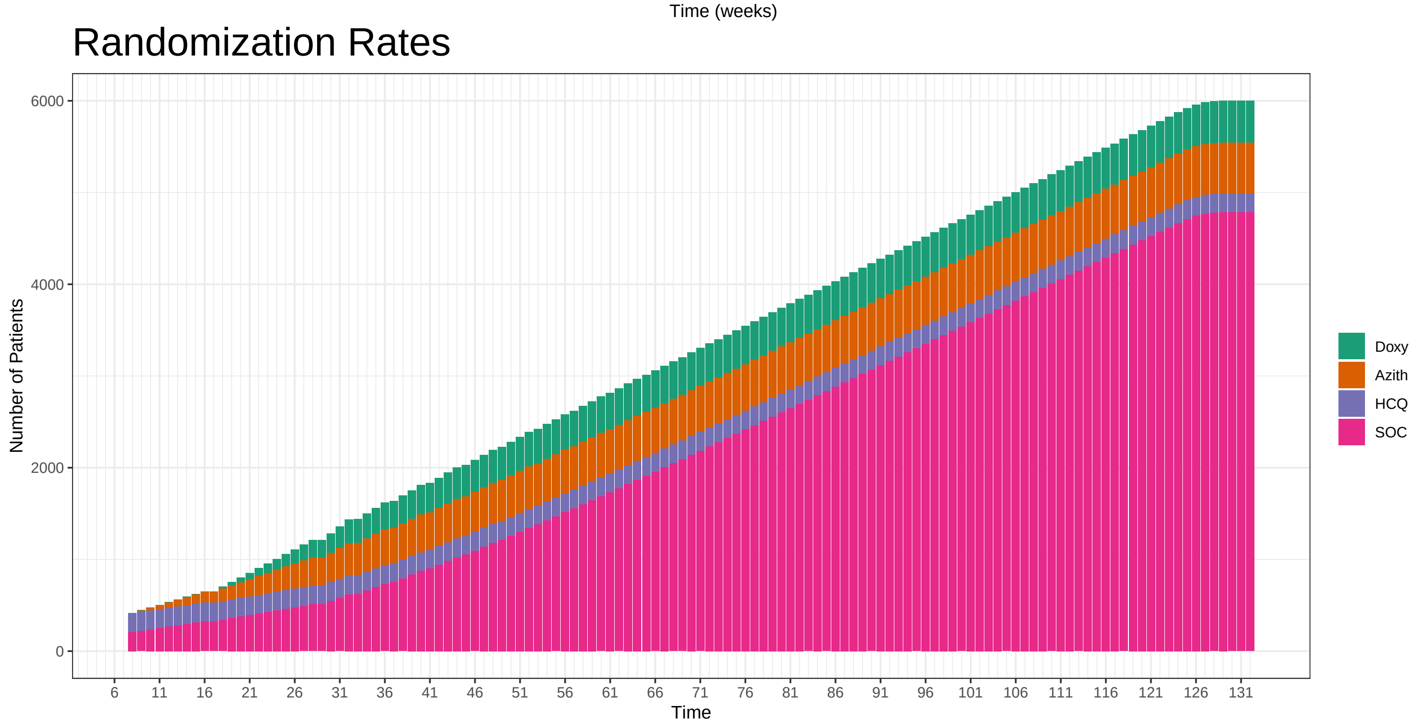

Hazard Ratios: HCQ: 1 Azith: 1 Doxy: 1.1

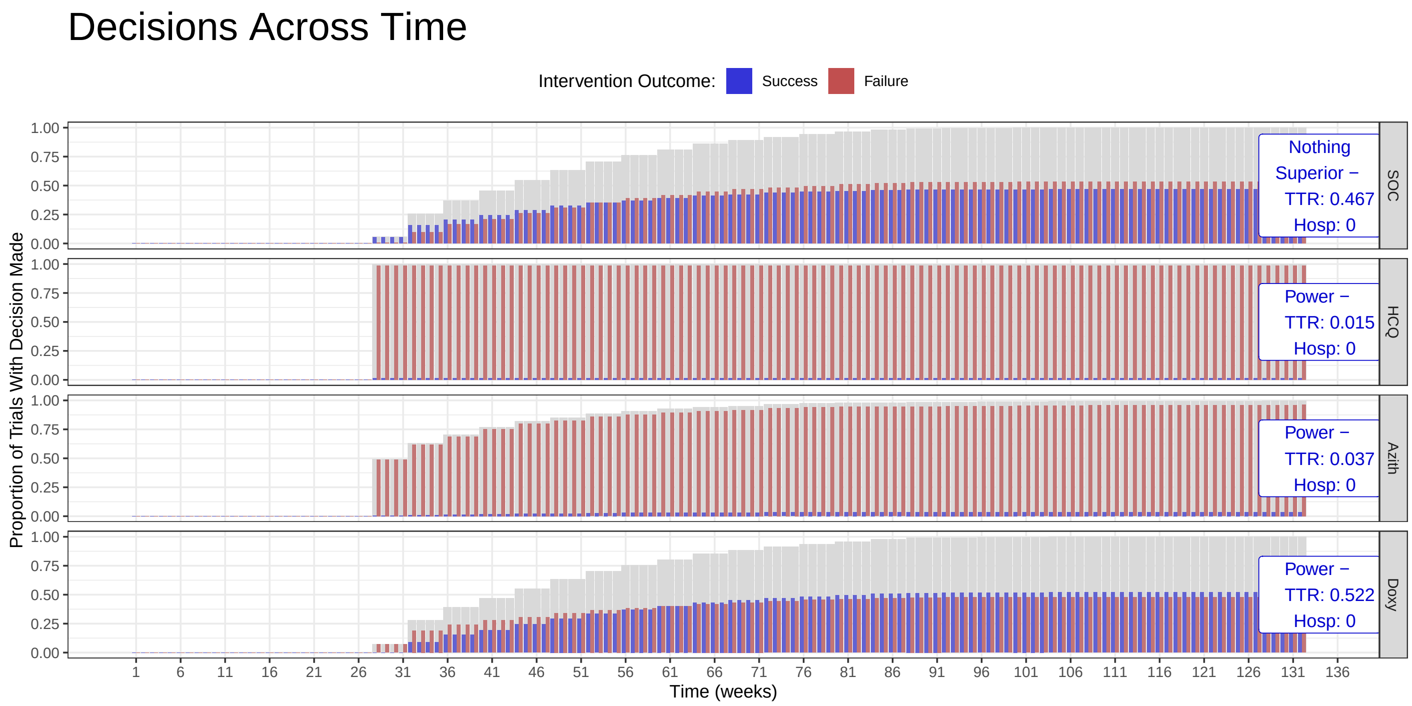

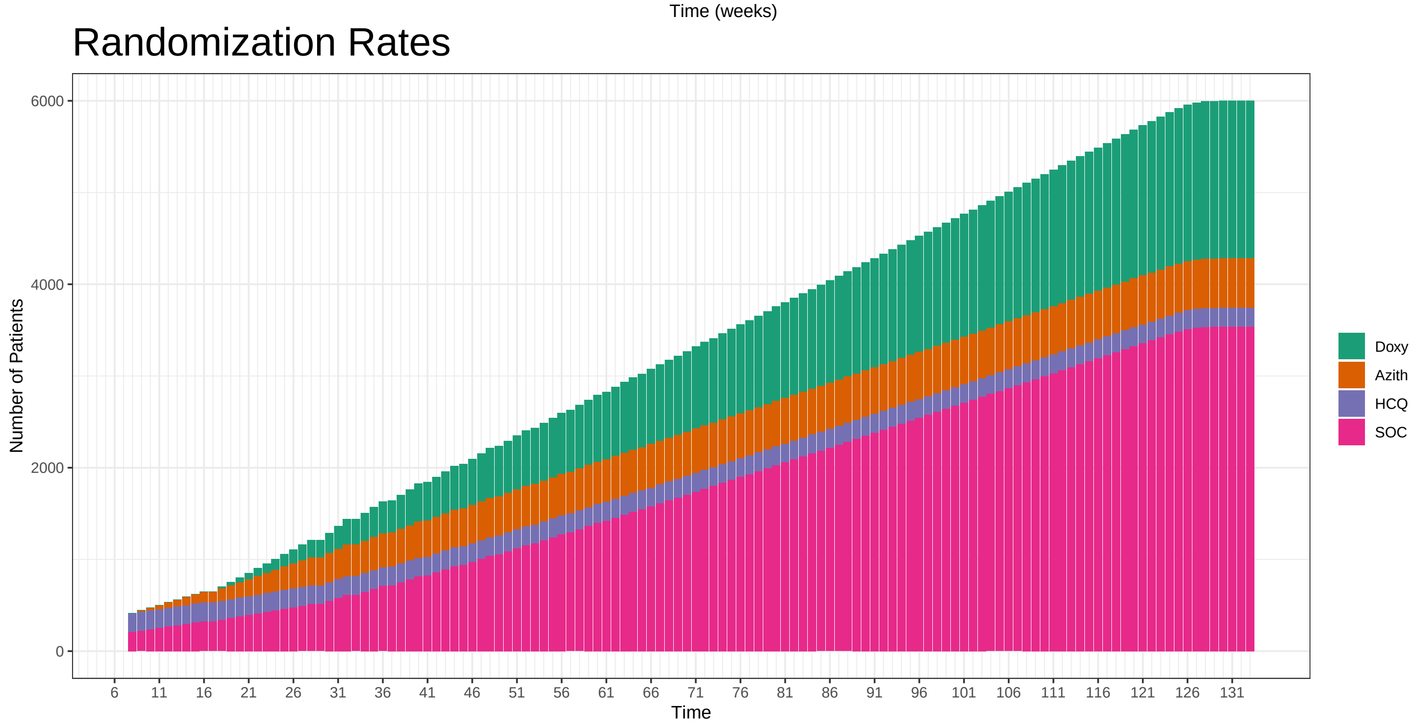

Hazard Ratios: HCQ: 1 Azith: 1 Doxy: 1.2

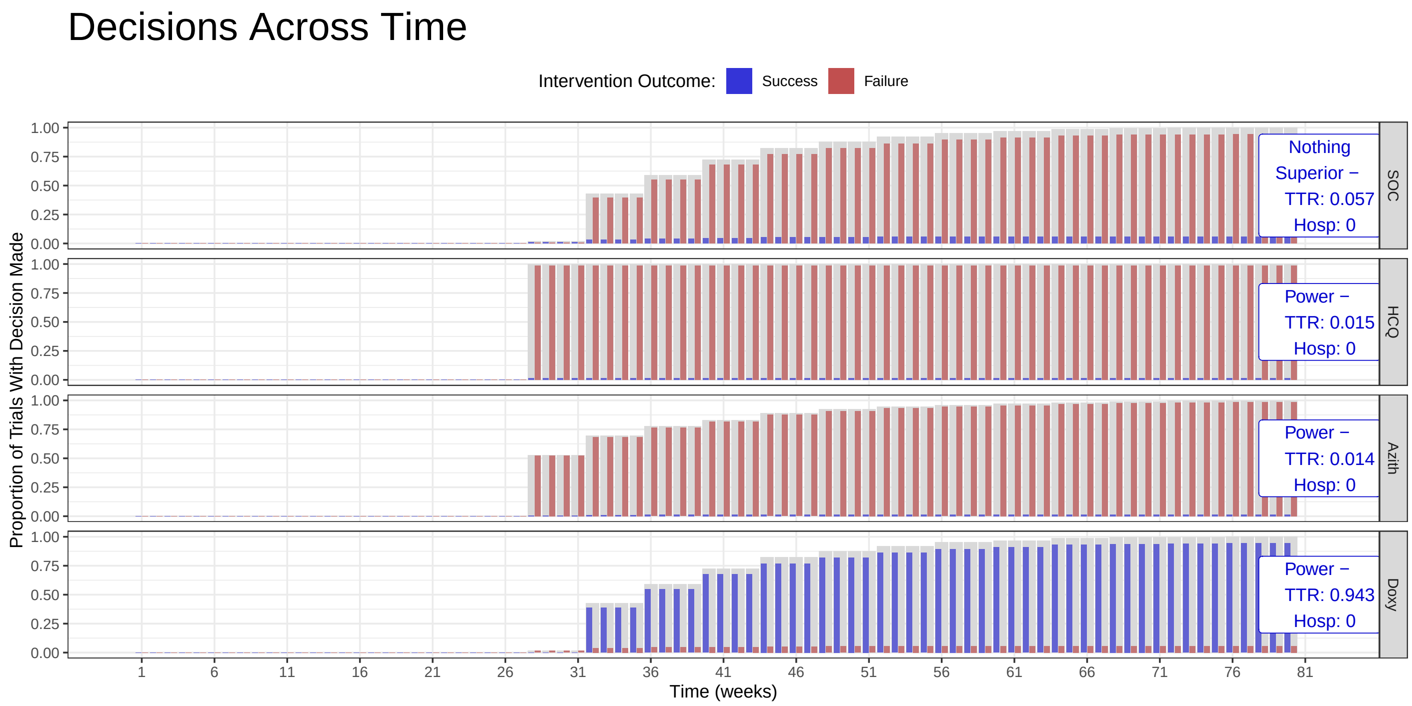

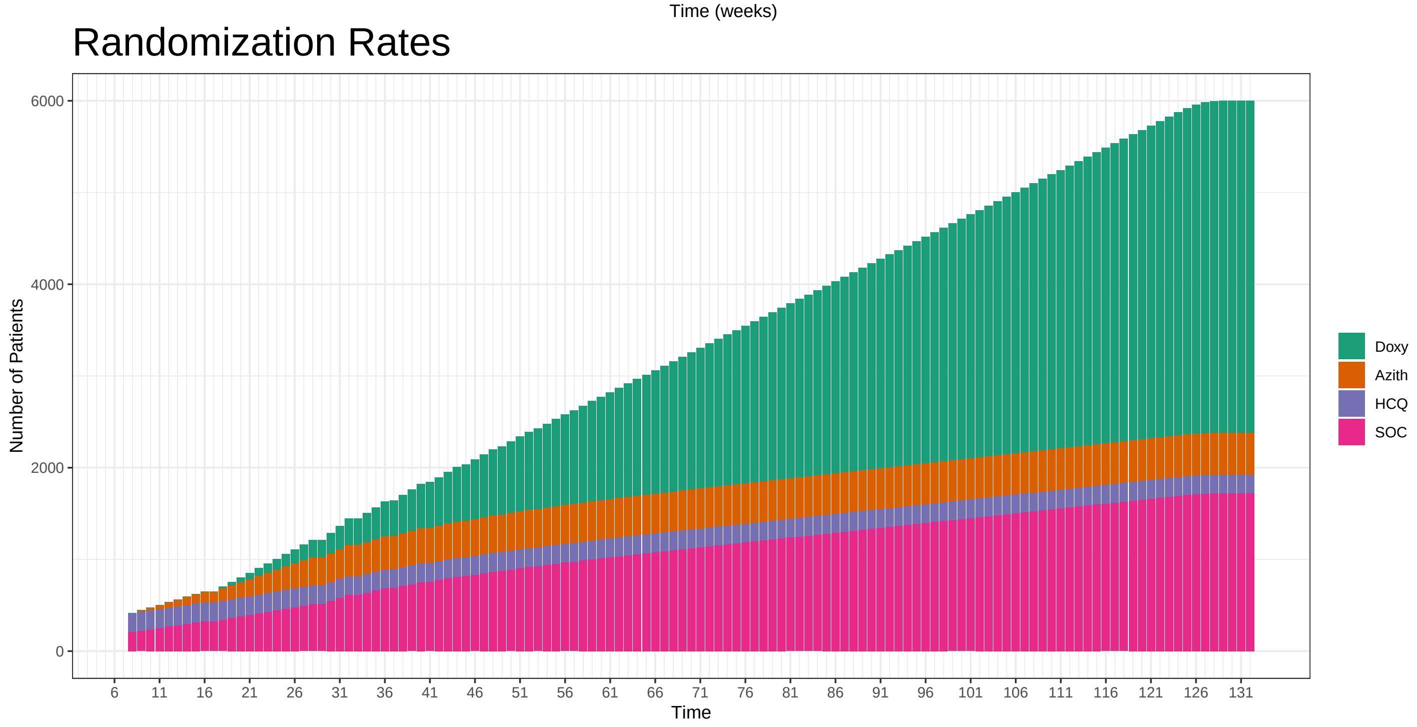

Hazard Ratios: HCQ: 1 Azith: 1 Doxy: 1.3

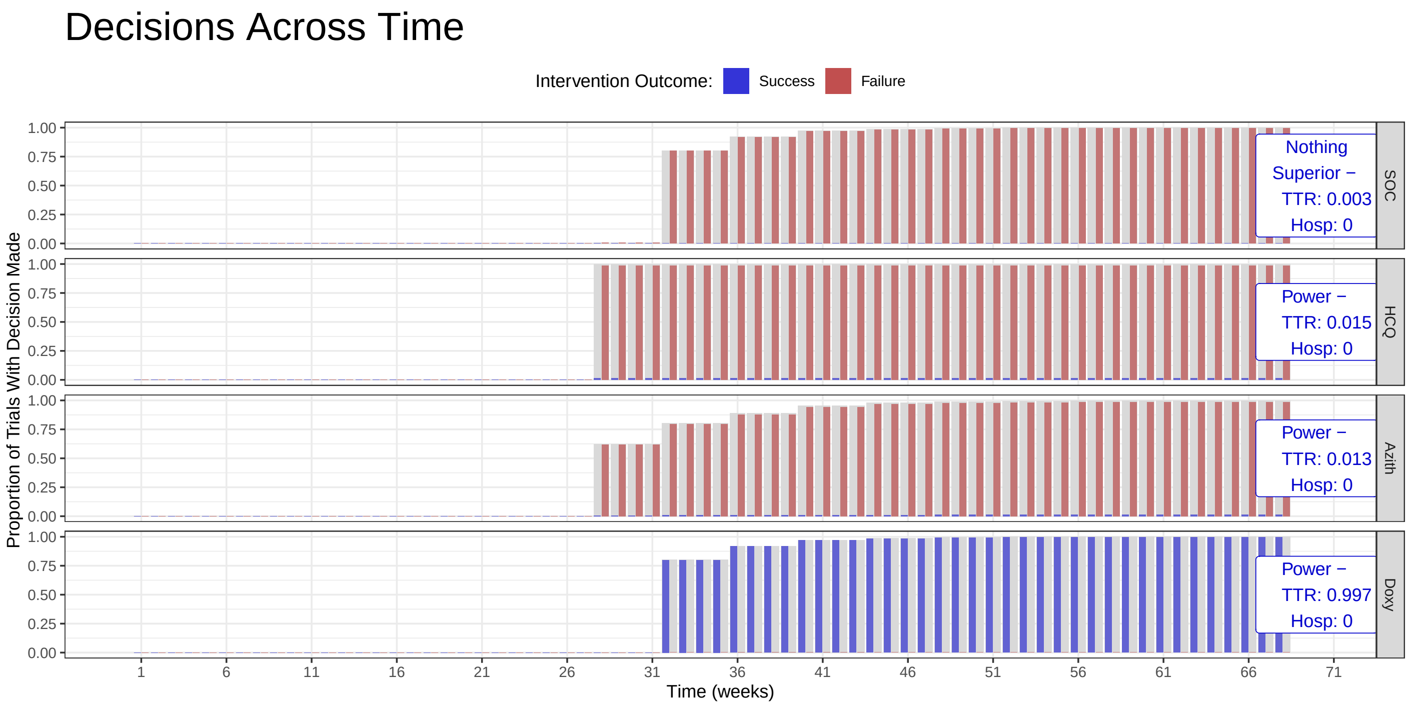

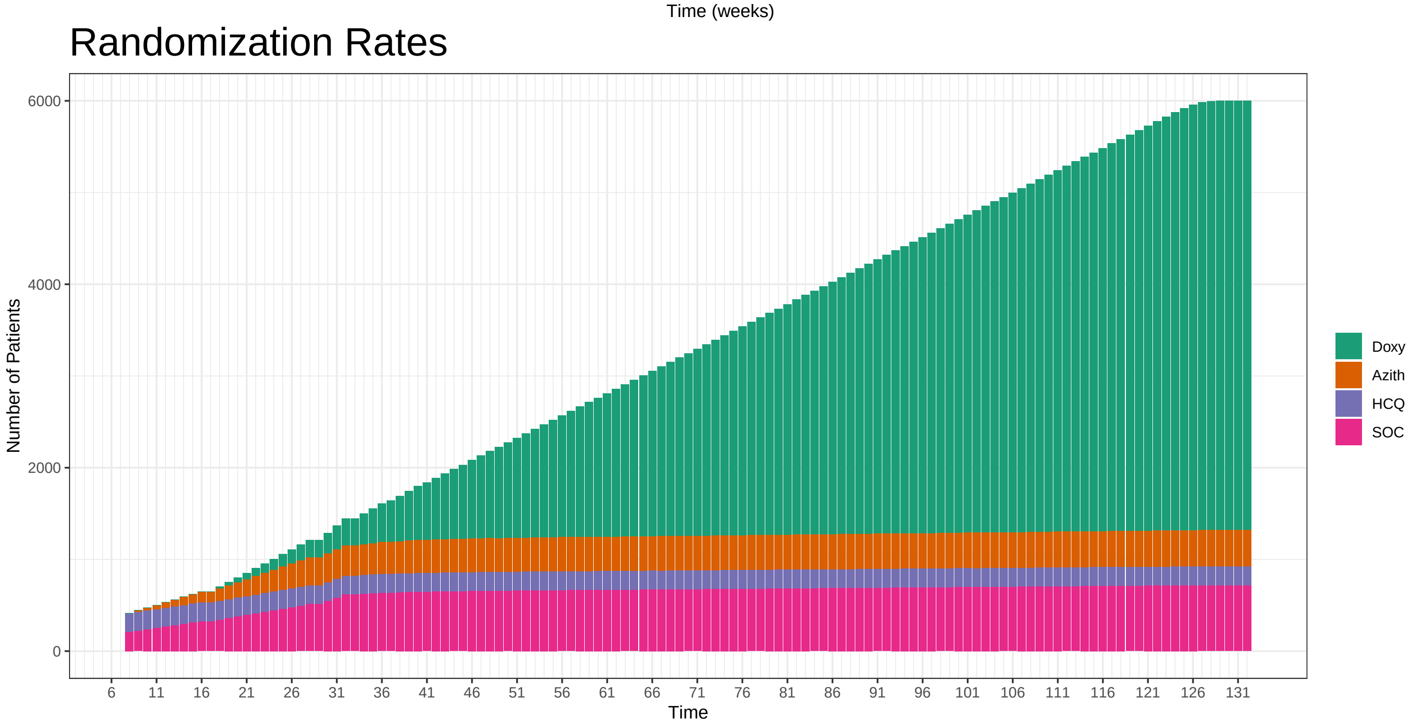

5.5 OCs Additional Scenarios: Fast Accrual

Principle OCs

Berry Consultants

10/26/2020

These operating characteristics are based on 1000 simulations per scenario across a variety of assumed treatment effects for experimental arms in the trial. The true number of patients randomized per day from April 1, 2020 to October 19, 2020. After this period is over, the simulations assume a faster than expected 100 patient per week accrual rate. In absence of a definitive rule, these simulations conduct an interim every 28 days. In addition, the true day of enrollment stopping of HCQ is used as well as the days for opening enrollment to the Azithromycin and Doxycycline arms. As such, up to at least October 19, 2020 (day 199) of the trial, the enrollment rates to each arm in these simulations are expected to align closely with the true enrollment rates to the arms in the trial.

Median TTR	Hazard Ratio			Power for TTR				Hospitalization Rate Benefit (%)			Power for TTR & Hosp		
SOC	HCQ	Azith	Doxy	SOC	HCQ	Azith	Doxy	HCQ	Azith	Doxy	HCQ	Azith	Doxy
9	1	1	1.0	0.962	0.014	0.023	0.016	0	0	0	0	0	0.000
9	1	1	1.1	0.613	0.015	0.020	0.378	0	0	1	0	0	0.067
9	1	1	1.2	0.120	0.015	0.016	0.880	0	0	2	0	0	0.588
9	1	1	1.3	0.011	0.014	0.009	0.989	0	0	3	0	0	0.975

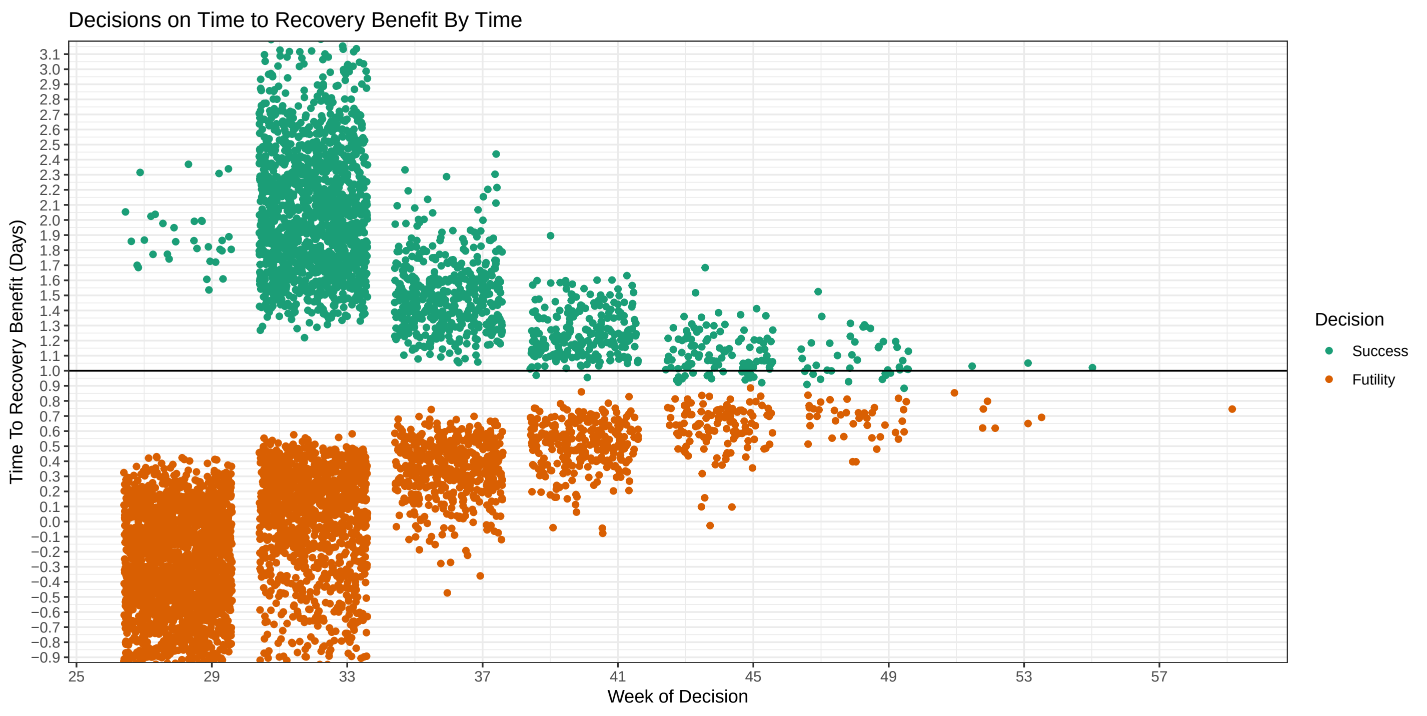

Decisions on Time to Recovery Benefit By Time
Time To Recovery Benefit (Days)
3.1
3.0
2.9
2.8
2.7
2.6
2.5
2.4
2.3
2.2
2.1
2.0
1.9
1.8
1.7
1.6
1.5
1.4
1.3
1.2
1.1
1.0
0.9
0.8
0.7
0.6
0.5
0.4
0.3
0.2
0.1
0.0
−0.1
−0.2
−0.3
−0.4
−0.5
−0.6
−0.7
−0.8
−0.9
25
29
33
37
41
45
49
53
57
Week of Decision
Decision
Success
Futility

Hazard Ratios: HCQ: NA Azith: NA Doxy: NA

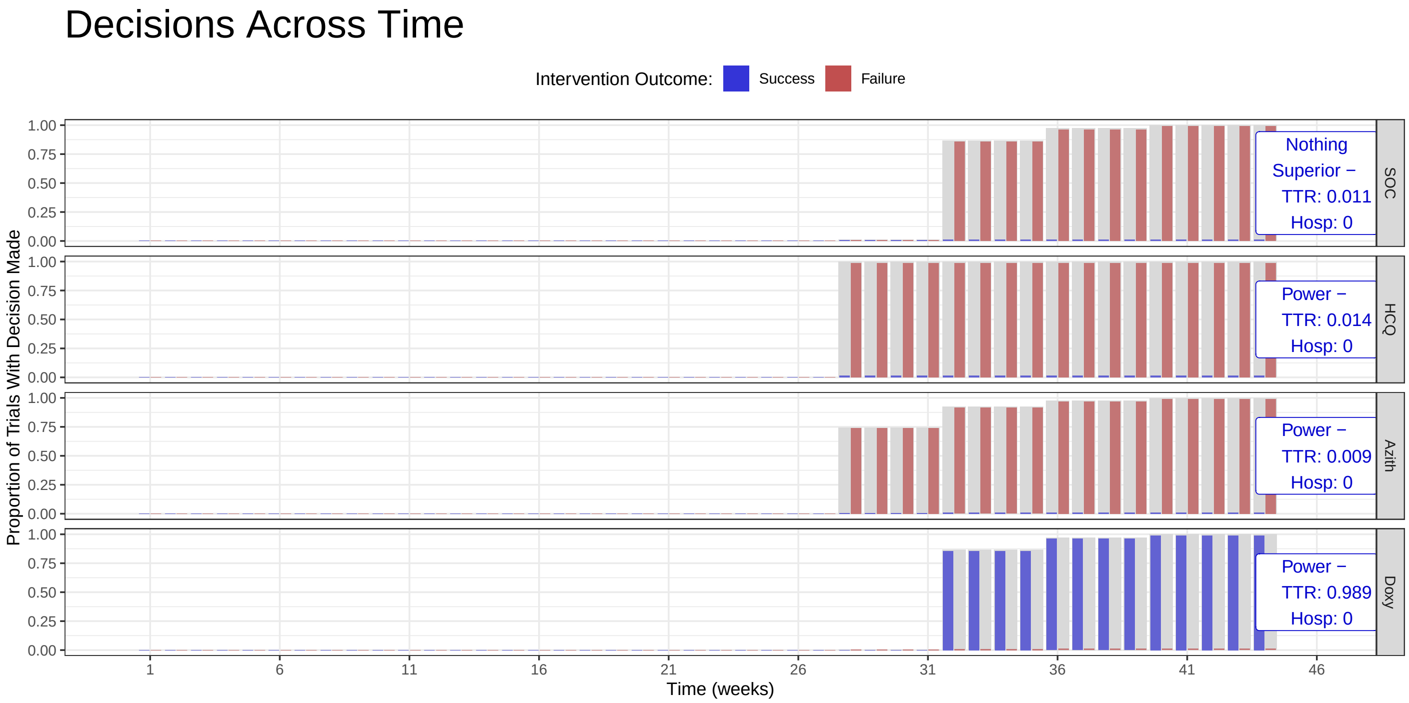

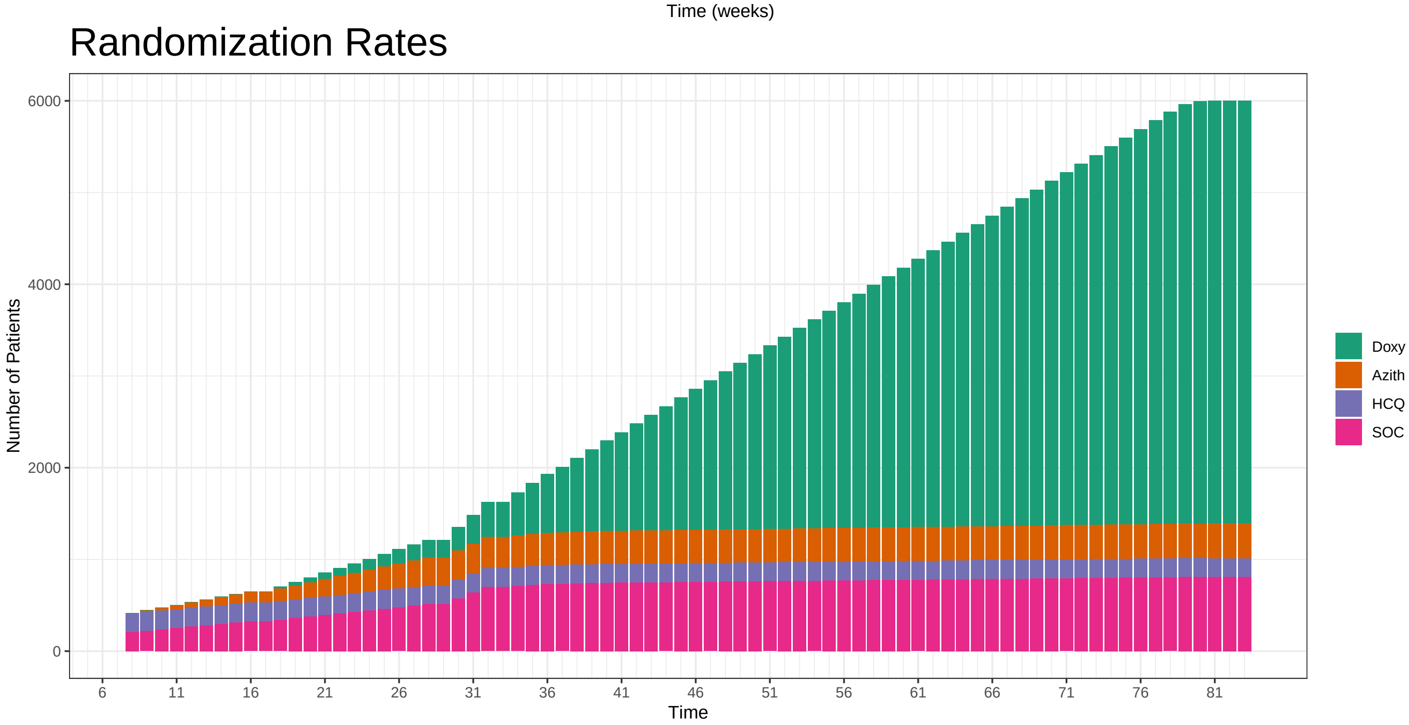

Hazard Ratios: HCQ: NA Azith: NA Doxy: NA

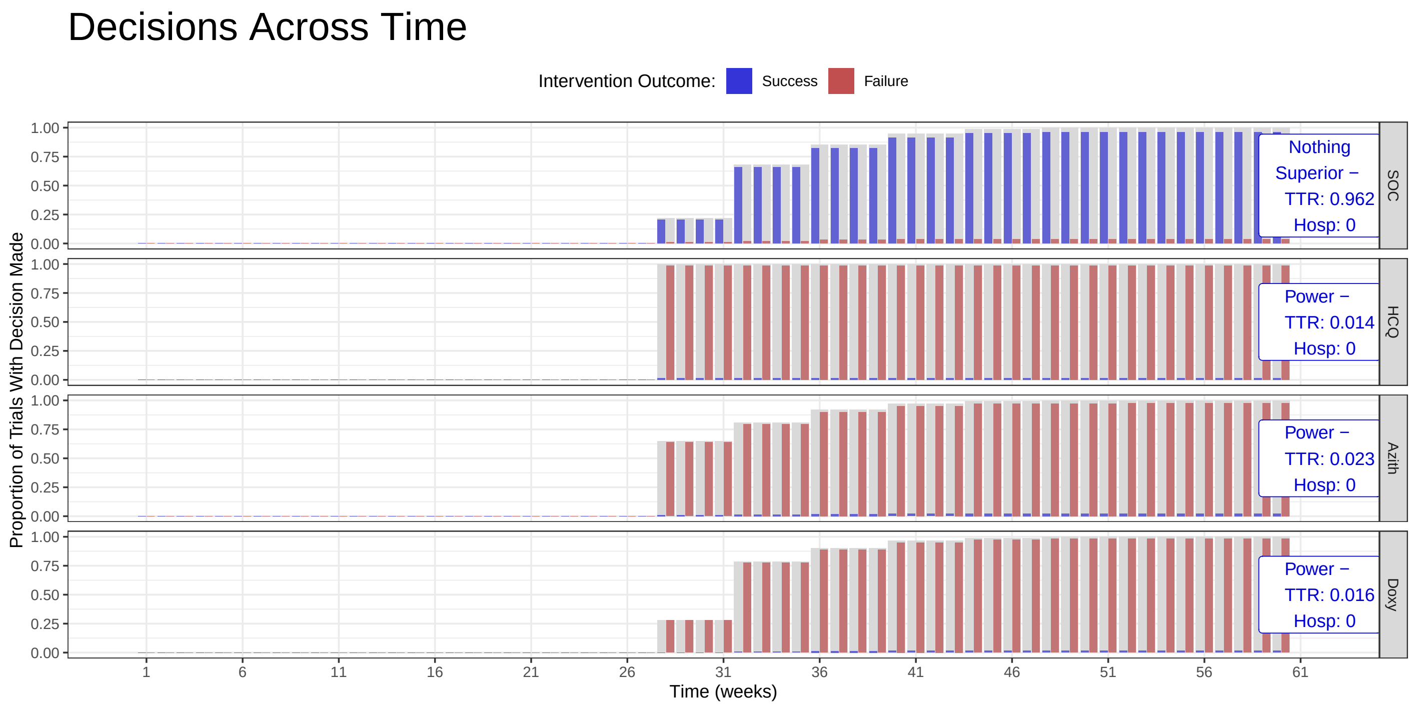

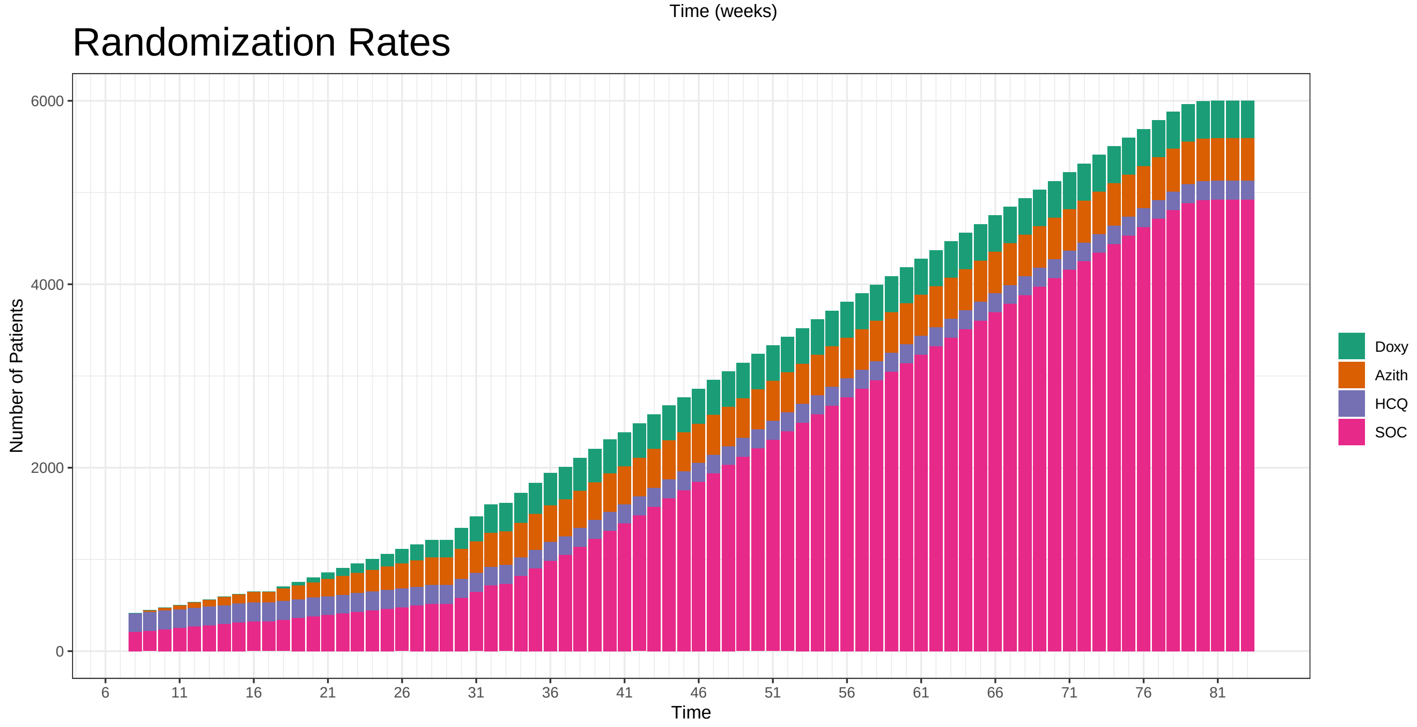

Hazard Ratios: HCQ: NA Azith: NA Doxy: NA

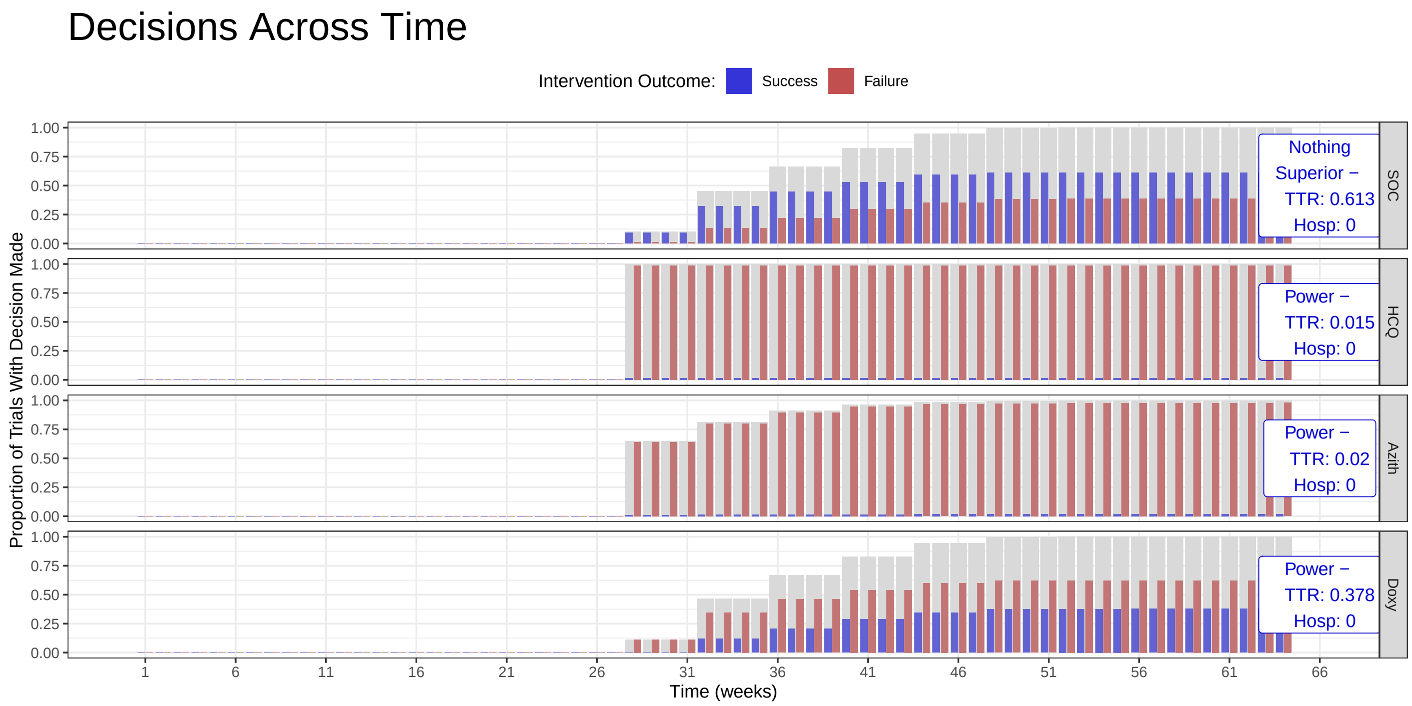

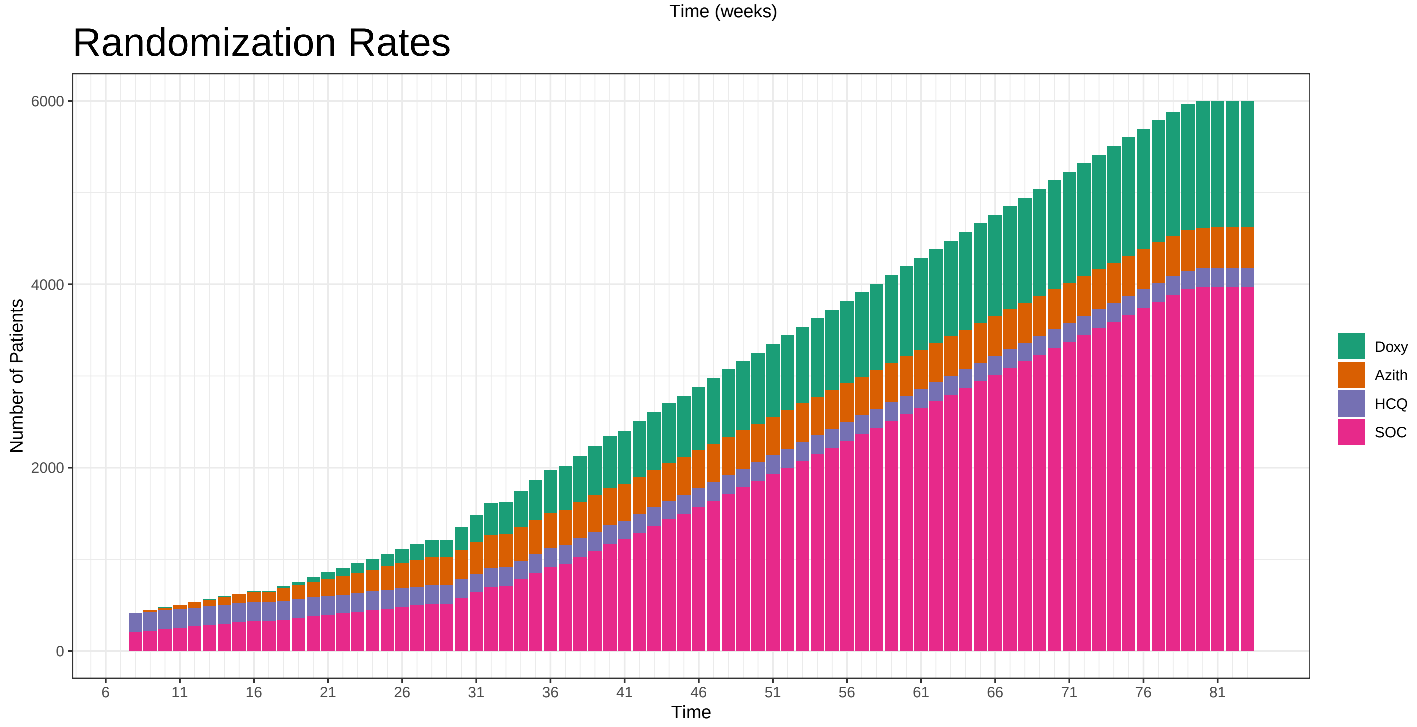

Hazard Ratios: HCQ: NA Azith: NA Doxy: NA

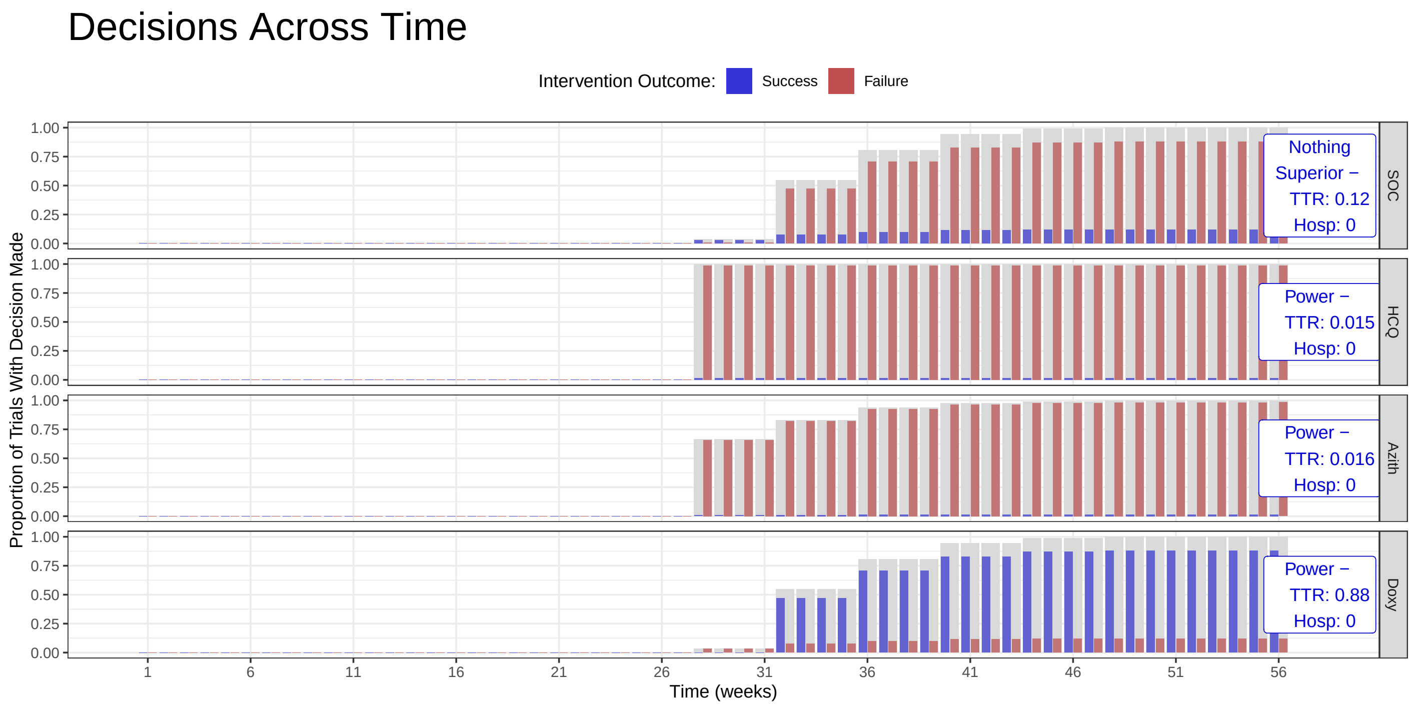

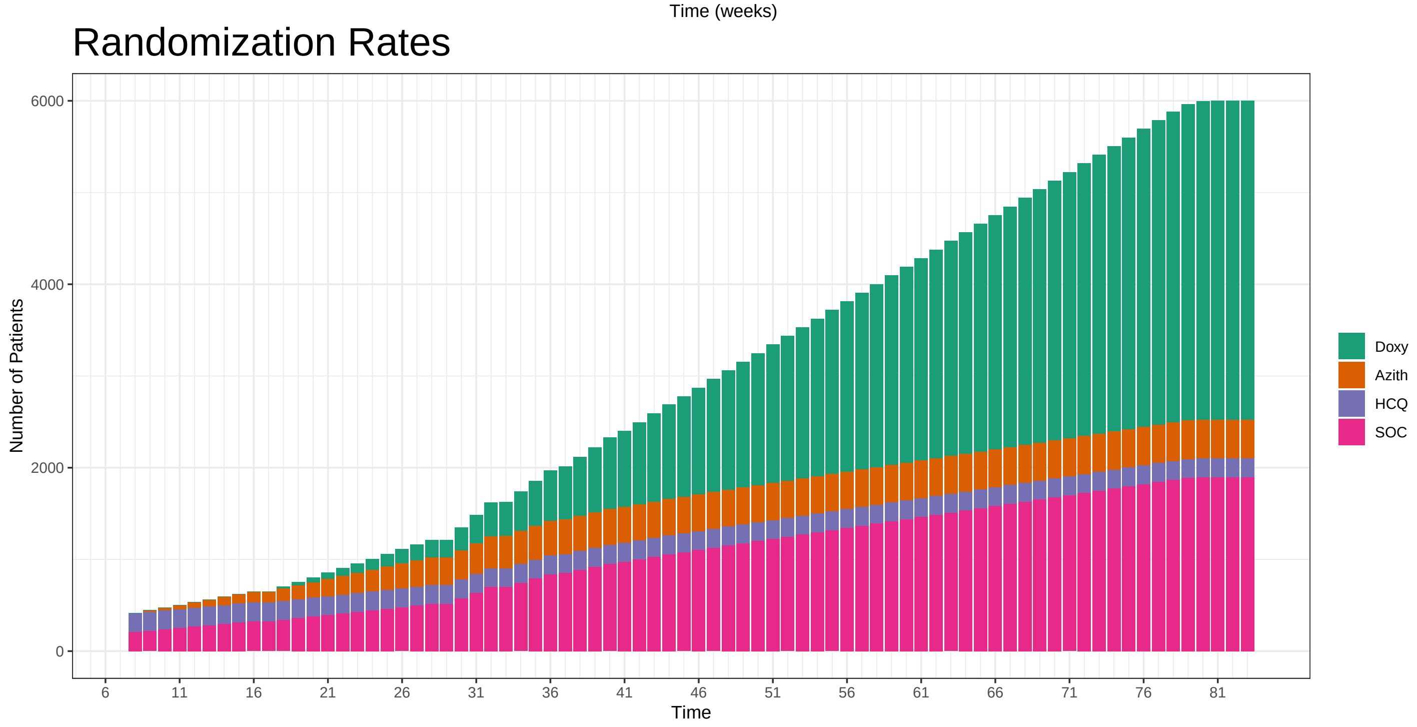

6 Simulated Example Trials

6.1 Simulated Example Trials: Three Interventions

This section explores the virtual simulations for the first three interventions versus Usual Care, with staggered entry (and HCQ suspension) as observed in the trial. We simulate virtual example trials and apply the adaptive algorithm exactly as described in the preceding sections. At each interim analysis, we perform an analysis of the data available at the time of the interim. For the purposes of the simulations, each of the trials continues to 4,000 participants, but in reality the end of the platform trial will be determined by available resources and the state of the pandemic. The calculations and statistical inferences using these data are summarized in an interim dashboard. The interim number and timing of the interim are provided in the upper left hand corner of the dashboard.

The table provides quantities calculated from the raw data as well as model estimated quantities. The columns denote the following.

Recovery Data

- Enrolled: The number of participants randomized to each arm at the time of the interim analysis
- Complete: The number of randomized participants with the opportunity to complete 28-days of follow-up
- Recovered: The number of randomized participants recovered, per primary endpoint definition (i.e. first recovery)
- Exposure Days: The total number of days of observed follow-up prior to a subjects's reported recovery across all participants
- Recoveries Per Day: The number of recoveries divided by the number of exposure days, interpreted as the number of recoveries per day per participant
- Estimated Hazard: The probability of recovery on day d given no recovery up to day d, with 95% Bayesian credible interval, averaged across all time segments
- Estimated HR: The estimated hazard ratio comparing the hazard of recovery for intervention j versus the Usual Care, with 95% Bayesian credible interval
- Estimated Median Time to Recovery: The model-based estimated median time to recovery for each treatment arm

Hospitalization Data:

- Hospitalizations: Total number of participants with hospitalization or death
- 28 Day Completers: Number of participants with opportunity to complete 28 days of follow-up
- Observed Hosp. Rate: Proportion of participants with hospitalization or death among those with the opportunity to complete 28 days of follow-up
- Est. Hosp. Rate: Model-based estimate for probability of hospitalization with corresponding 95% credible interval

Recovery Inferences:

- Pr(Superiority): The model-based estimated probability of superiority with respect to recovery endpoint for intervention j versus Usual Care
- Pr(Meaningful Effect): The model-based estimated probability that the benefit in median time to recovery for intervention j compared to Usual Care is at least 1.5 days
- Pr(Best): The model-based estimated probability that intervention j is superior to all other active interventions with respect to the recovery endpoint
- Randomization Probability: The randomization probabilities to be used for treatment allocation until the next interim analysis

Hospitalization Inferences:

- Pr(Superiority): The model-based estimated probability of superiority for intervention j versus Usual Care with respect to the hospitalization/death endpoint
- Pr(Meaningful Effect): The model-based estimated probability that the reduction in proportion of persons with hospitalization/death for intervention j relative to Usual Care 0.02 or greater (i.e., at least 2 percentage points)

The plot and table in the lower left corner of the dashboard provide a more detailed look at the observed data for the recovery endpoint. The plot is a Kaplan-Meier plot showing the proportion of subjects recovered for days 0-28. The table below the plot provides the number not yet recovered by 0, 7, 14, 21, and 28 days for each arm. Both of these summaries are showing observed data that contribute to the statistical modeling.

The two plots on the lower right show summaries of the statistical modeling of the recovery endpoint. The first plot shows the Bayesian posterior distributions of the hazard ratios corresponding to each intervention. Hazard ratios greater than one indicate faster recovery. The second plot (on far right) shows the Bayesian posterior distribution of the median time to recovery in days, with the center of each distribution shown in text above the peak of the distribution.

Below we show four virtual trials to illustrate the adaptive platform trial.

6.2 Example 1

April 1, 2020

Before Patient Enrollment Begins

Recovery Data

	Enrolled	Complete	Recovered	Exposure Days	Recoveries Per Day	Estimated Hazard	Estimated HR (95% interval)	Estimated Median Survival Time
SOC	0	0	0	0				
HCQ	0	0	0	0				
Azith	0	0	0	0				
Doxy	0	0	0	0				
Total	0	0	0	0				

Hospitalization Data

Hospitalizations	28 Day Completers	Observed Hosp. Rate	Est. Hosp. Rate (95% interval)
0	0		
0	0		
0	0		
0	0		
0	0		

Intervention Status
Enrolling
Enrolling
Unopened
Unopened

Observed Patient Recoveries

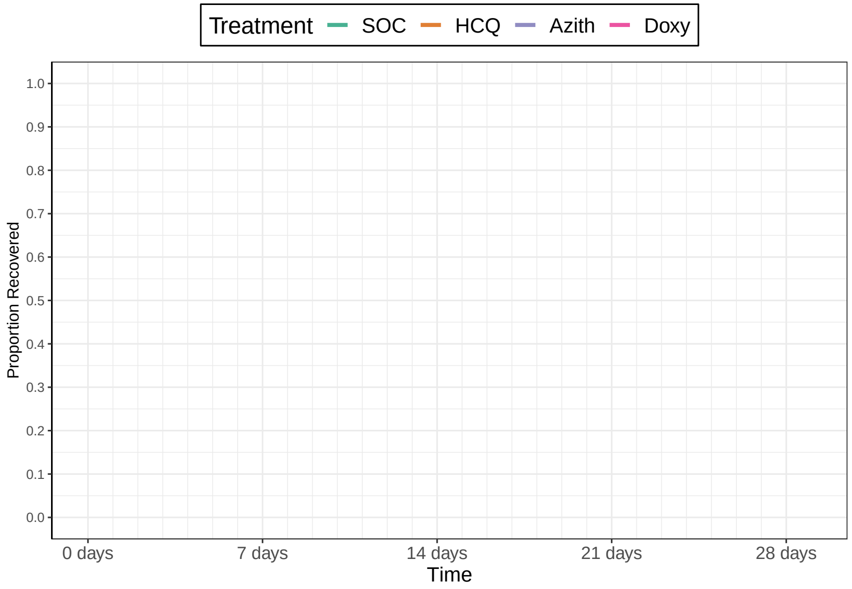

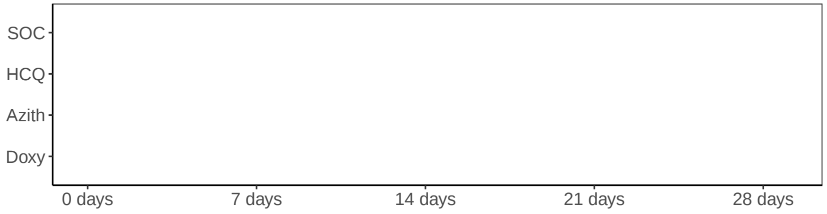

Recovery Inferences

	Pr(Superiority)	Pr(Meaningful Effect)	Pr(Best)	Randomization Probability
SOC				0.5
HCQ				0.5
Azith				0
Doxy				0

Hospitalization Inferences

Pr(Superiority)	Pr(Meaningful Effect)
0	0
0	0
0	0
0	0

Hazard Ratio Estimates

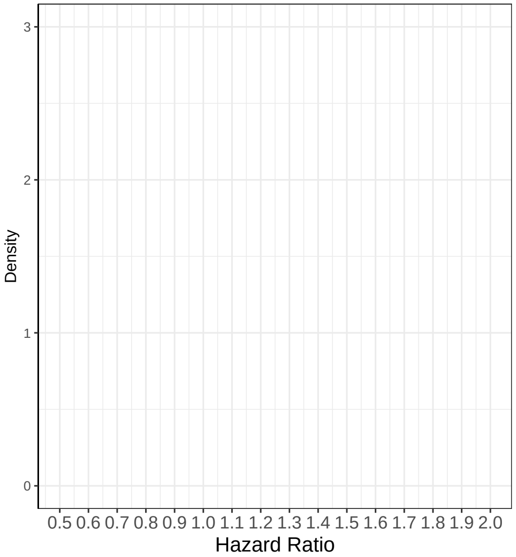

Median Time To Recovery Estimates

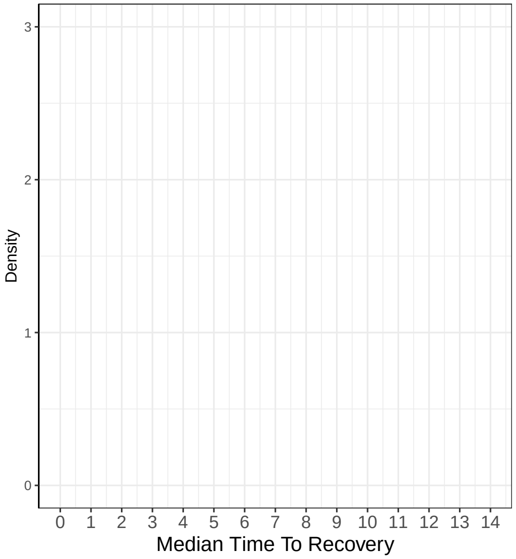

Remove Drug on May 22, 2020

Recovery Data

	Enrolled	Complete	Recovered	Exposure Days	Recoveries Per Day	Estimated Hazard	Estimated HR (95% interval)	Estimated Median Time to Recovery
SOC	207	143	134	1817	0.074			
HCQ	206	147	140	1861	0.075			
Azith	0	0	0	0				
Doxy	0	0	0	0				
Total	413	290	274	3679	0.074			

Hospitalization Data

Hospitalizations	28 Day Completers	Observed Hosp. Rate	Est. Hosp. Rate (95% interval)
2	79	0.0253	
6	78	0.0769	
0	0	NA	
0	0	NA	
8	157	0.0510	

Intervention Status
Enrolling
Removing
Unopened
Unopened

Observed Patient Recoveries

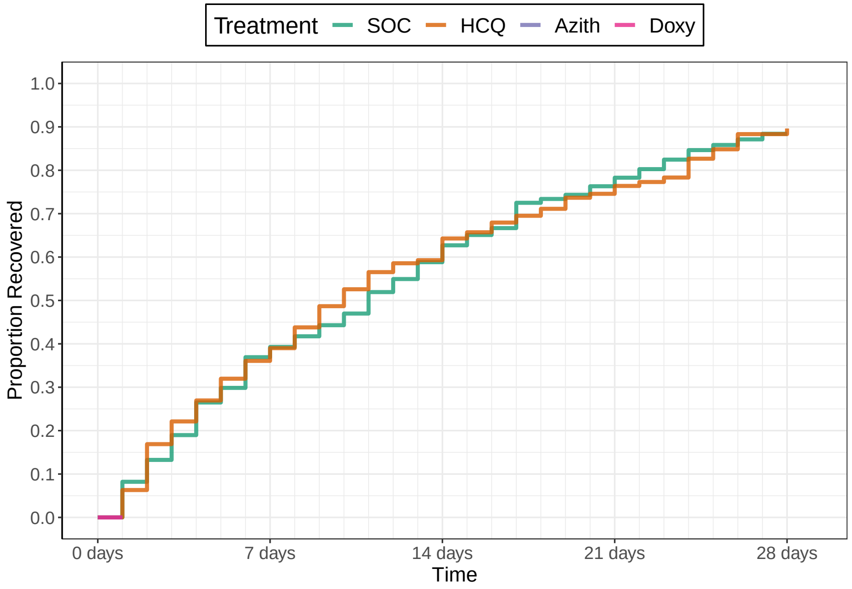

Number Un−recovered

	0 days	7 days	14 days	21 days	28 days
SOC	207	107	53	24	9
HCQ	206	109	57	28	9
Azith					
Doxy					

Recovery Inferences

	Pr(Superiority)	Pr(Meaningful Effect)	Pr(Best)	Randomization Probability
SOC				1
HCQ			0	0
Azith			0	0
Doxy			0	0

Hospitalization Inferences

Pr(Superiority)	Pr(Meaningful Effect)

Hazard Ratio Estimates

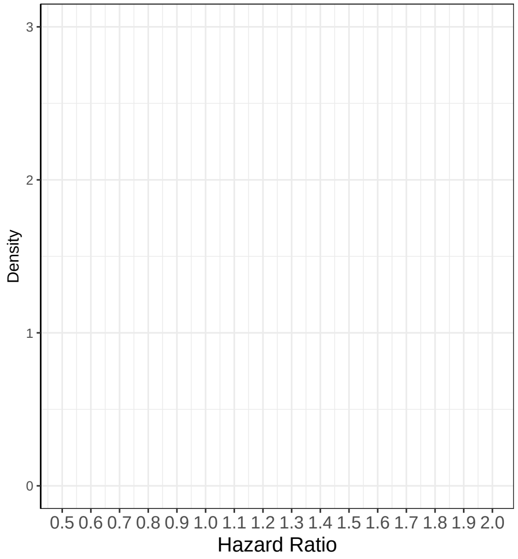

Median Time To Recovery Estimates

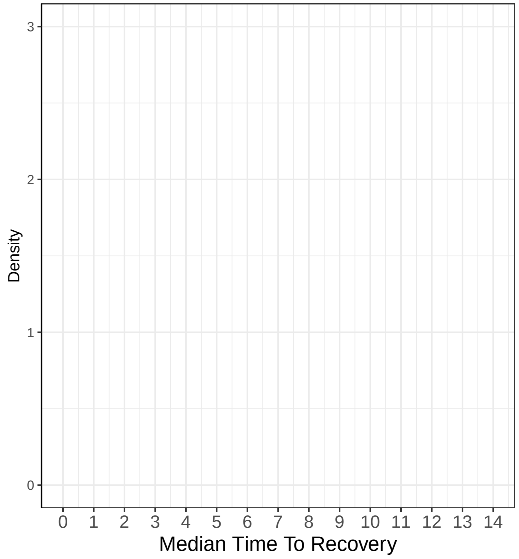

Add Drug on May 23, 2020

Recovery Data

	Enrolled	Complete	Recovered	Exposure Days	Recoveries Per Day	Estimated Hazard	Estimated HR (95% interval)	Estimated Median Time to Recovery
SOC	209	147	138	1881	0.073			
HCQ	206	151	143	1918	0.075			
Azith	0	0	0	0				
Doxy	0	0	0	0				
Total	415	298	281	3799	0.074			

Hospitalization Data

Hospitalizations	28 Day Completers	Observed Hosp. Rate	Est. Hosp. Rate (95% interval)
2	80	0.025	
6	80	0.075	
0	0	NA	
0	0	NA	
8	160	0.050	

Intervention Status
Enrolling
Paused
Introducing
Unopened

Observed Patient Recoveries

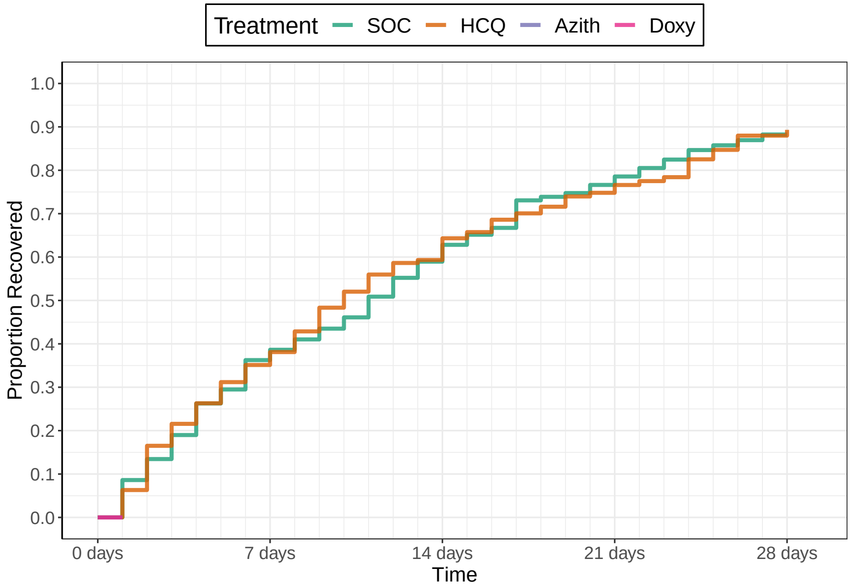

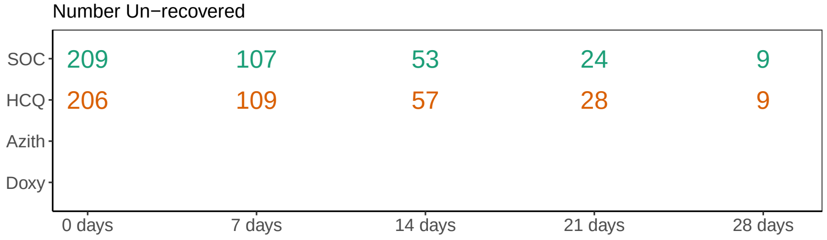

Recovery Inferences

	Pr(Superiority)	Pr(Meaningful Effect)	Pr(Best)	Randomization Probability
SOC				0.5
HCQ			0	0
Azith			1	0.5
Doxy			0	0

Hospitalization Inferences

Pr(Superiority)	Pr(Meaningful Effect)

Hazard Ratio Estimates

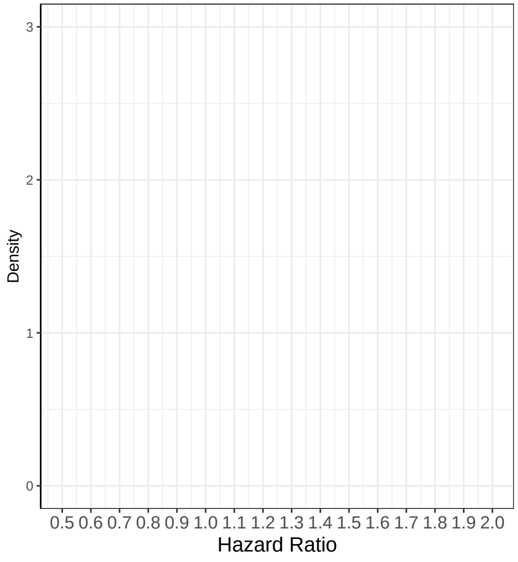

Median Time To Recovery Estimates

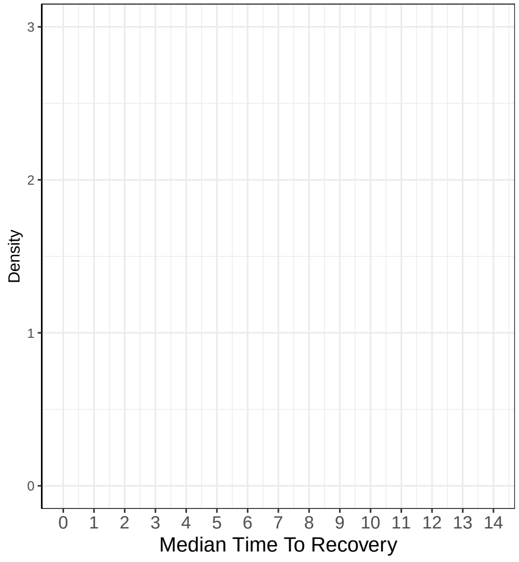

Add Drug on July 23, 2020

Recovery Data

	Enrolled	Complete	Recovered	Exposure Days	Recoveries Per Day	Estimated Hazard	Estimated HR (95% interval)	Estimated Median Time to Recovery
SOC	327	308	272	3615	0.075			
HCQ	206	206	176	2546	0.069			
Azith	118	103	94	1175	0.08			
Doxy	0	0	0	0				
Total	651	617	542	7336	0.074			

Hospitalization Data

Hospitalizations	28 Day Completers	Observed Hosp. Rate	Est. Hosp. Rate (95% interval)
3	286	0.0105	
6	206	0.0291	
8	77	0.1039	
0	0	NA	
17	569	0.0299	

Intervention Status
Enrolling
Paused
Introducing
Introducing

Observed Patient Recoveries

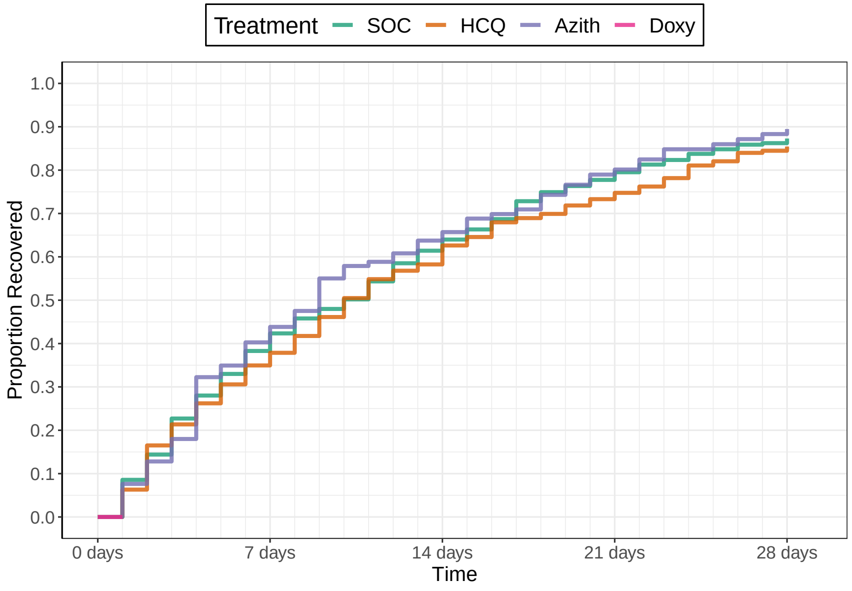

Number Un−recovered

	0 days	7 days	14 days	21 days	28 days
SOC	327	198	120	63	39
HCQ	206	134	86	55	32
Azith	118	67	37	18	10
Doxy					

Recovery Inferences

	Pr(Superiority)	Pr(Meaningful Effect)	Pr(Best)	Randomization Probability
SOC				0.33
HCQ			0	0
Azith			1	0.33
Doxy			0	0.33

Hospitalization Inferences

Pr(Superiority)	Pr(Meaningful Effect)

Hazard Ratio Estimates

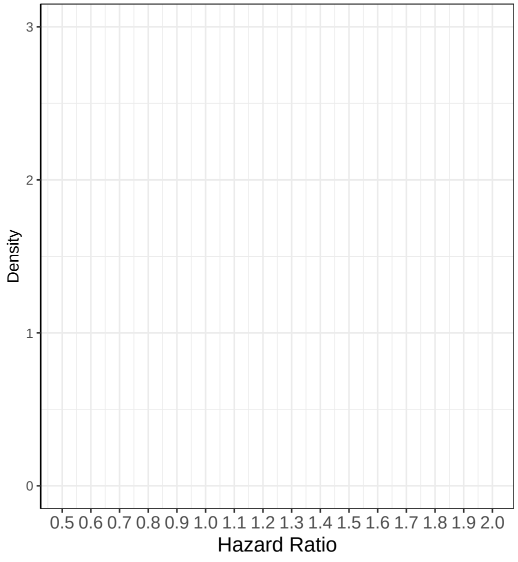

Median Time To Recovery Estimates

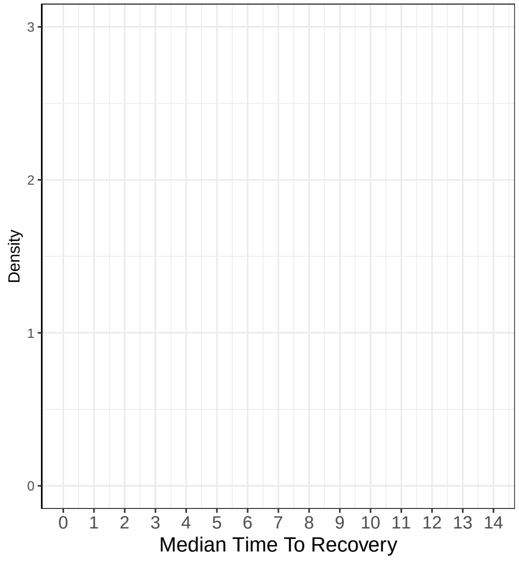

Interim 1 on October 15, 2020

Recovery Data

	Enrolled	Complete	Recovered	Exposure Days	Recoveries Per Day	Estimated Hazard	Estimated HR (95% interval)	Estimated Median Time to Recovery
SOC	496	437	388	5313	0.073	0.071	1	9.61
HCQ	206	206	176	2546	0.069	0.067	0.937 (0.795, 1.113)	10.27
Azith	313	268	252	2762	0.091	0.085	1.202 (1.03, 1.4)	8.01
Doxy	195	151	147	1353	0.109	0.097	1.368 (1.131, 1.625)	7.04
Total	1210	1062	963	11975	0.08			

Hospitalization Data

Hospitalizations	28 Day Completers	Observed Hosp. Rate	Est. Hosp. Rate (95% interval)
9	395	0.0228	0.02 (0.0095, 0.0339)
6	206	0.0291	0.0334 (0.0135, 0.061)
12	198	0.0606	0.0413 (0.0219, 0.0666)
7	74	0.0946	0.0407 (0.0179, 0.0713)
34	873	0.0389	

Intervention Status
LostOnTTR
Futility
SuccessOnTTRFutilityOnHosp Not Announced
Enrolling

Observed Patient Recoveries

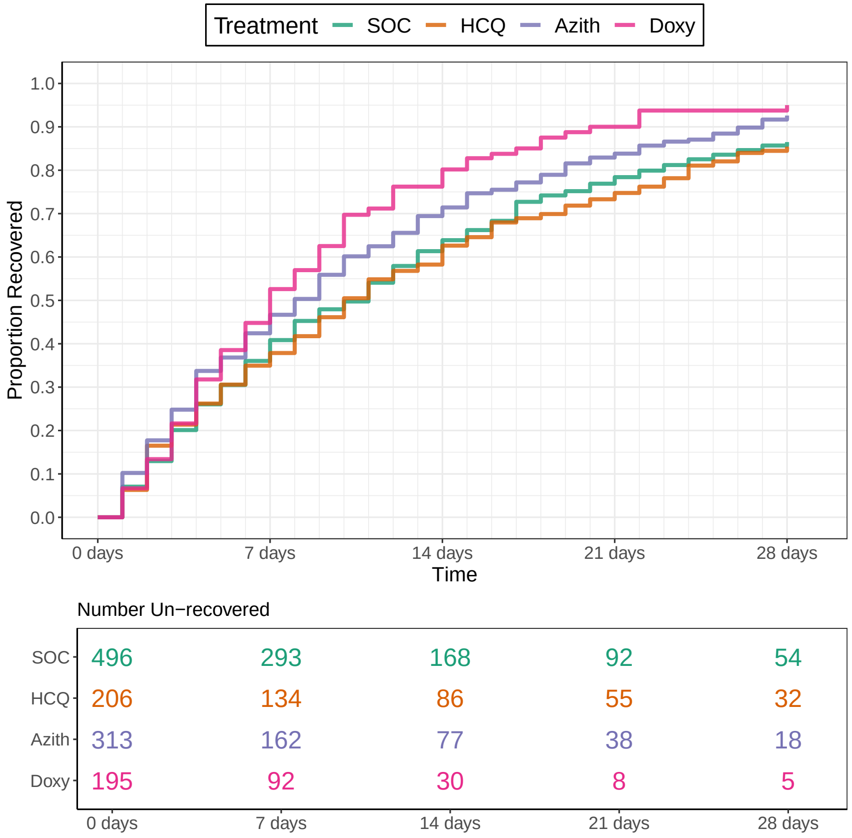

Recovery Inferences

	Pr(Superiority)	Pr(Meaningful Effect)	Pr(Best)	Randomization Probability
SOC				0.25
HCQ	0.2183	0.009	0	0
Azith	0.9903	0.5687	0.0788	0.25
Doxy	0.9993	0.9257	0.6712	0.5

Hospitalization Inferences

Pr(Superiority)	Pr(Meaningful Effect)
0.1652	0.0038
0.0455	5e−04
0.0712	0

Hazard Ratio Estimates

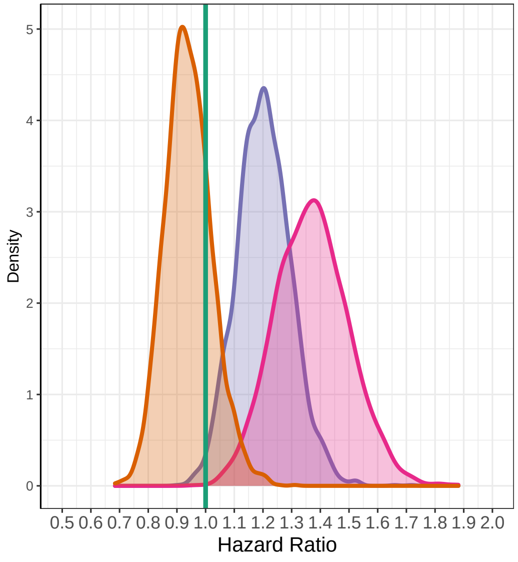

Median Time To Recovery Estimates

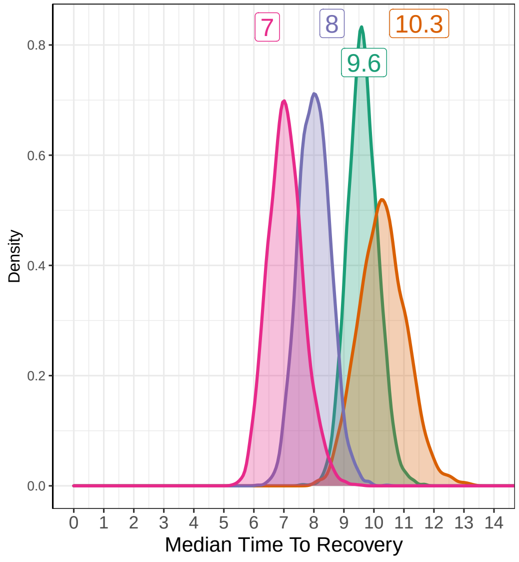

Interim 2 on November 12, 2020

Recovery Data

	Enrolled	Complete	Recovered	Exposure Days	Recoveries Per Day	Estimated Hazard	Estimated HR (95% interval)	Estimated Median Time to Recovery
SOC	550	536	472	6252	0.075	0.073	1	9.28
HCQ	206	206	176	2546	0.069	0.066	0.915 (0.768, 1.081)	10.17
Azith	377	352	327	3592	0.091	0.085	1.173 (1.022, 1.343)	7.89
Doxy	294	275	265	2468	0.107	0.098	1.348 (1.156, 1.548)	6.85
Total	1427	1369	1240	14858	0.083			

Hospitalization Data

Hospitalizations	28 Day Completers	Observed Hosp. Rate	Est. Hosp. Rate (95% interval)
14	496	0.0282	0.0272 (0.0153, 0.0422)
6	206	0.0291	0.034 (0.0137, 0.0619)
15	313	0.0479	0.0423 (0.024, 0.0654)
9	195	0.0462	0.0339 (0.0162, 0.057)
44	1210	0.0364	

Intervention Status
LostOnTTR
Dropped
SuccessOnTTRFutilityOnHosp Not Announced
SuccessOnTTRFutilityOnHosp *Announced*

Observed Patient Recoveries

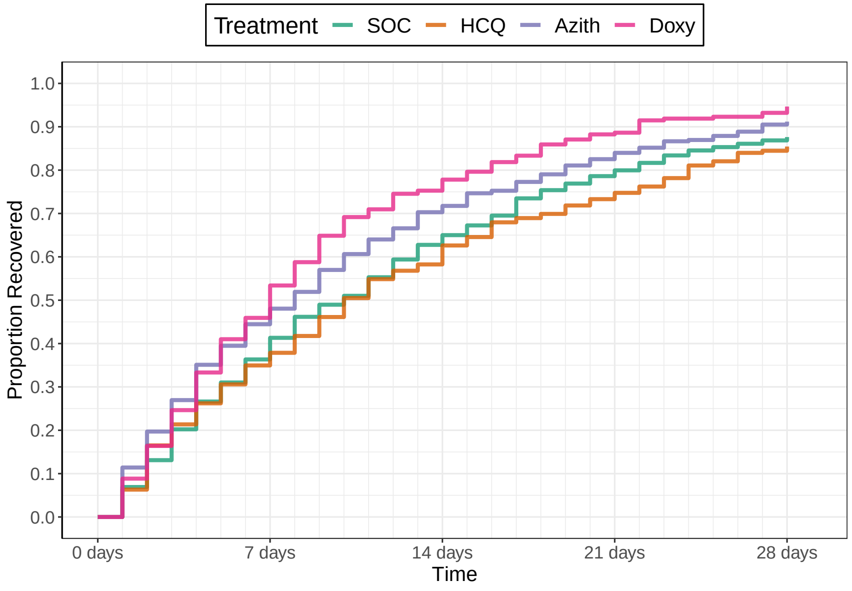

Number Un−recovered

	0 days	7 days	14 days	21 days	28 days
SOC	550	345	199	112	68
HCQ	206	134	86	55	32
Azith	377	201	102	60	28
Doxy	294	152	68	30	14

Recovery Inferences

	Pr(Superiority)	Pr(Meaningful Effect)	Pr(Best)	Randomization Probability
SOC				0.33
HCQ	0.1457	0.002	0	0
Azith	0.987	0.442	0.0415	0.33
Doxy	1	0.937	0.7085	0.33

Hospitalization Inferences

Pr(Superiority)	Pr(Meaningful Effect)
0.3375	0.02
0.1098	0.002
0.3078	0.0132

Hazard Ratio Estimates

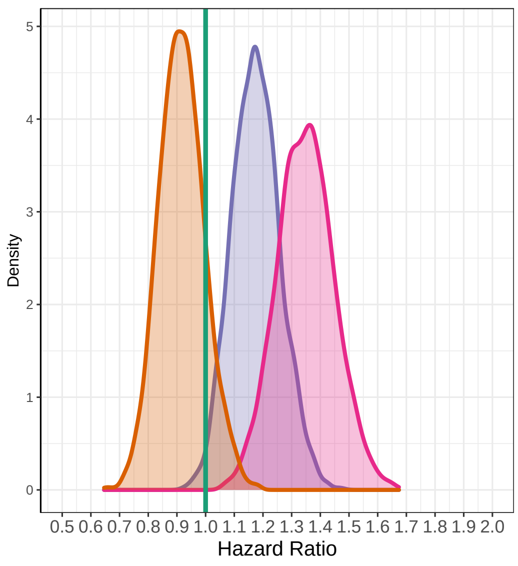

Median Time To Recovery Estimates

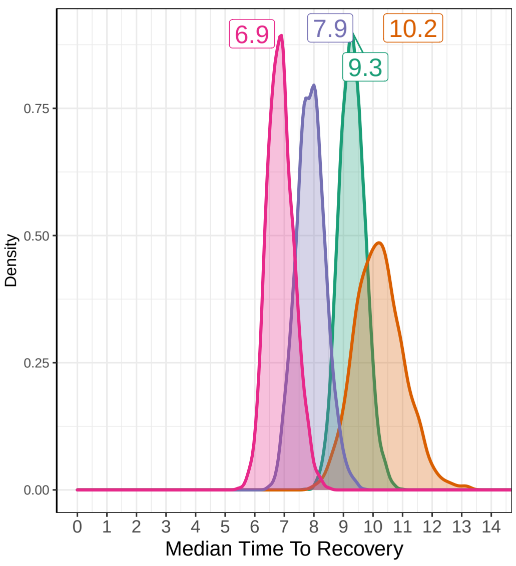

Interim 3 on September 05, 2022

Recovery Data

	Enrolled	Complete	Recovered	Exposure Days	Recoveries Per Day	Estimated Hazard	Estimated HR (95% interval)	Estimated Median Time to Recovery
SOC	2078	2078	1838	24211	0.076	0.074	1	9.53
HCQ	206	206	176	2546	0.069	0.067	0.913 (0.784, 1.056)	10.41
Azith	1918	1918	1769	19480	0.091	0.087	1.185 (1.11, 1.266)	8.08
Doxy	1798	1798	1703	16353	0.104	0.099	1.347 (1.261, 1.443)	7.13
Total	6000	6000	5486	62591	0.088			

Hospitalization Data

Hospitalizations	28 Day Completers	Observed Hosp. Rate	Est. Hosp. Rate (95% interval)
51	2078	0.0245	0.0251 (0.019, 0.0322)
6	206	0.0291	0.0334 (0.0137, 0.0612)
60	1918	0.0313	0.0318 (0.0244, 0.0399)
40	1798	0.0222	0.0227 (0.0164, 0.0299)
157	6000	0.0262	

Intervention Status
Complete
Dropped
SuccessOnTTRFutilityOnHosp Not Announced
SuccessOnTTRFutilityOnHosp *Announced*

Observed Patient Recoveries

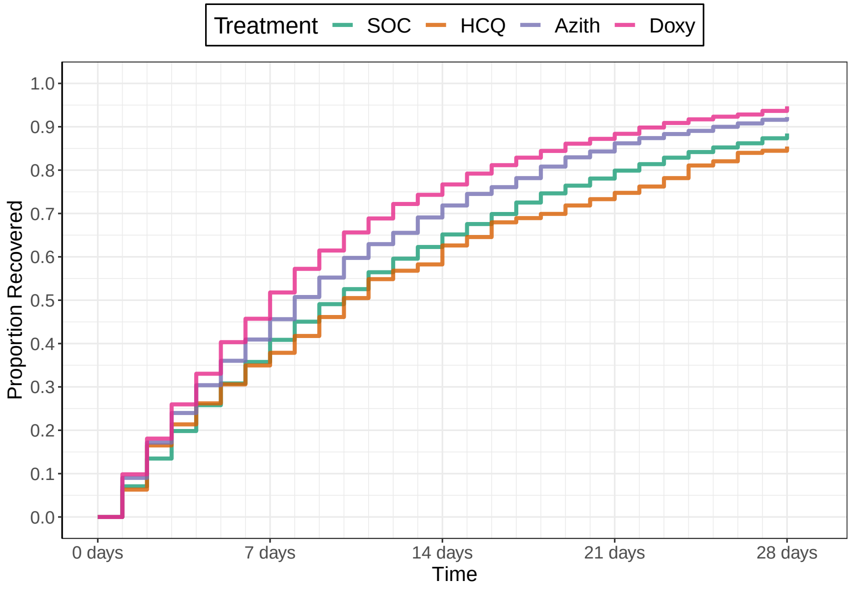

Number Un−recovered

	0 days	7 days	14 days	21 days	28 days
SOC	2078	1335	784	456	263
HCQ	206	134	86	55	32
Azith	1918	1133	593	301	161
Doxy	1798	976	462	230	114

Recovery Inferences

	Pr(Superiority)	Pr(Meaningful Effect)	Pr(Best)	Randomization Probability
SOC				
HCQ	0.1137	3e−04	0	
Azith	1	0.438	2e−04	
Doxy	1	0.9997	0.7498	

Hospitalization Inferences

Pr(Superiority)	Pr(Meaningful Effect)
0.2758	0.0012
0.095	0
0.6968	2e−04

Hazard Ratio Estimates

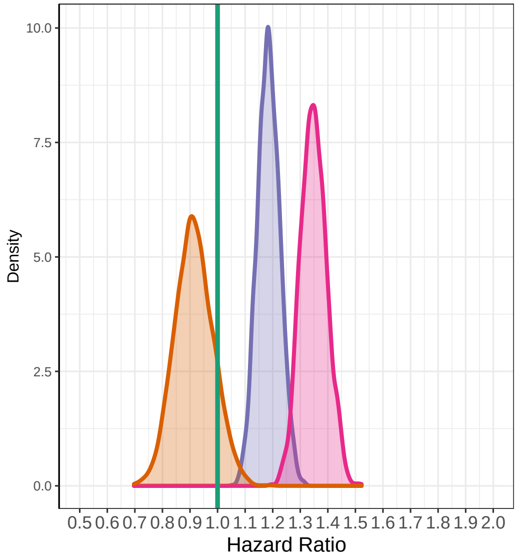

Median Time To Recovery Estimates

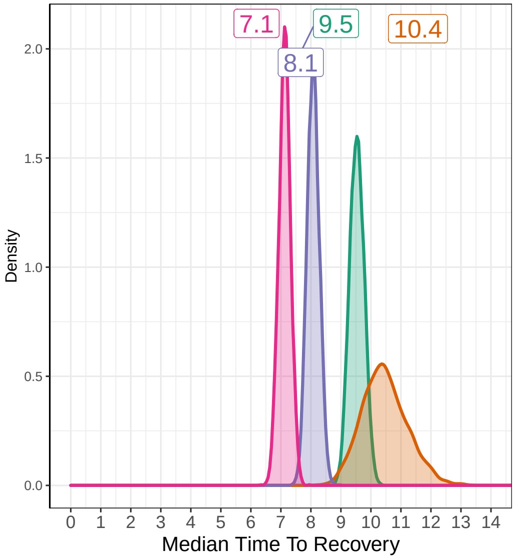

6.3 Example 2

April 1, 2020

Before Patient Enrollment Begins

Recovery Data

	Enrolled	Complete	Recovered	Exposure Days	Recoveries Per Day	Estimated Hazard	Estimated HR (95% interval)	Estimated Median Survival Time
SOC	0	0	0	0				
HCQ	0	0	0	0				
Azith	0	0	0	0				
Doxy	0	0	0	0				
Total	0	0	0	0				

Hospitalization Data

Hospitalizations	28 Day Completers	Observed Hosp. Rate	Est. Hosp. Rate (95% interval)
0	0		
0	0		
0	0		
0	0		
0	0		

Intervention Status
Enrolling
Enrolling
Unopened
Unopened

Observed Patient Recoveries

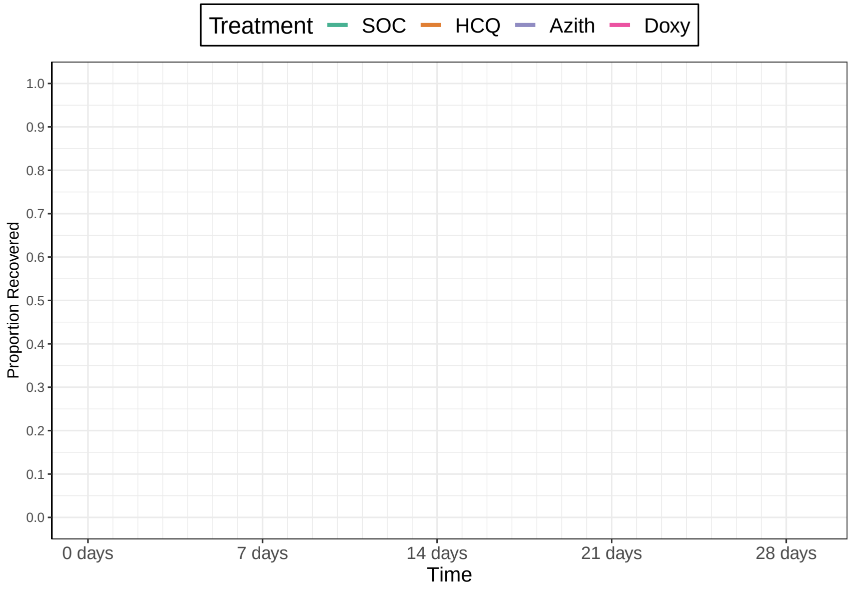

Number Un−recovered

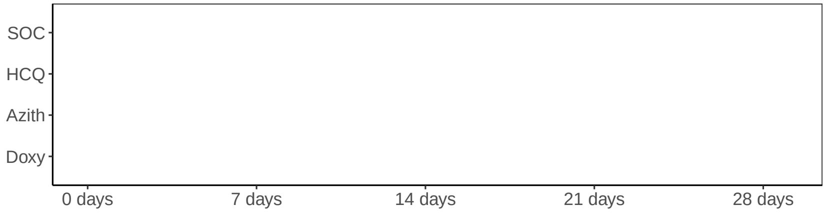

Recovery Inferences

	Pr(Superiority)	Pr(Meaningful Effect)	Pr(Best)	Randomization Probability
SOC				0.5
HCQ				0.5
Azith				0
Doxy				0

Hospitalization Inferences

Pr(Superiority)	Pr(Meaningful Effect)
0	0
0	0
0	0
0	0

Hazard Ratio Estimates

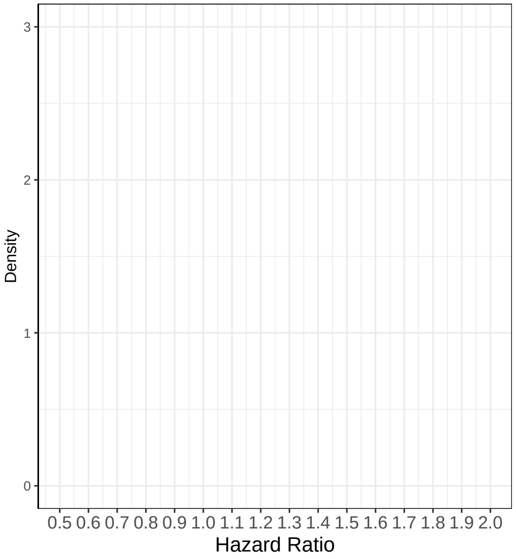

Median Time To Recovery Estimates

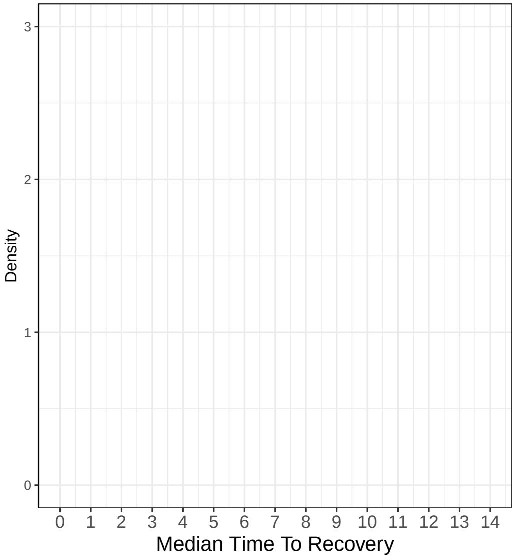

Remove Drug on May 22, 2020

Recovery Data

	Enrolled	Complete	Recovered	Exposure Days	Recoveries Per Day	Estimated Hazard	Estimated HR (95% interval)	Estimated Median Time to Recovery
SOC	206	146	136	1807	0.075			
HCQ	207	149	143	1737	0.082			
Azith	0	0	0	0				
Doxy	0	0	0	0				
Total	413	295	279	3544	0.079			

Hospitalization Data

Hospitalizations	28 Day Completers	Observed Hosp. Rate	Est. Hosp. Rate (95% interval)
1	78	0.0128	
10	79	0.1266	
0	0	NA	
0	0	NA	
11	157	0.0701	

Intervention Status
Enrolling
Removing
Unopened
Unopened

Observed Patient Recoveries

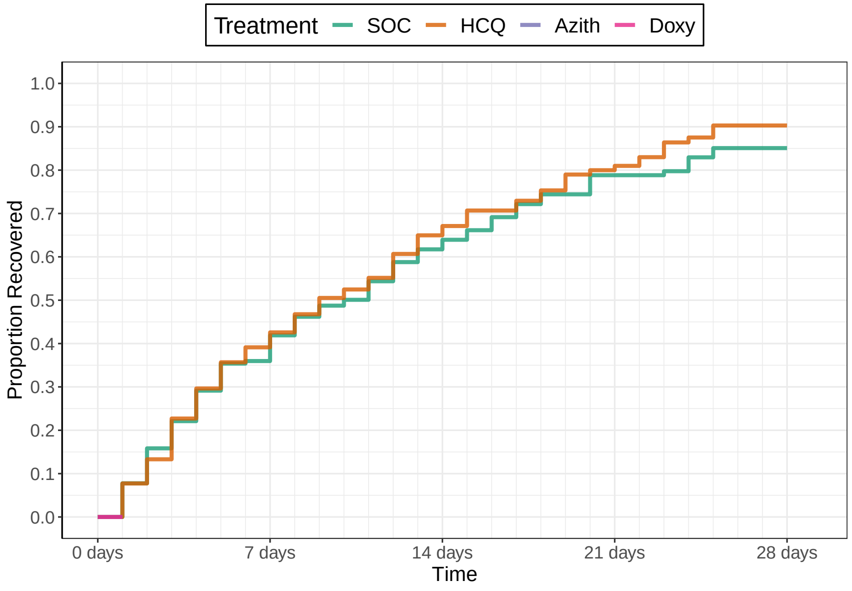

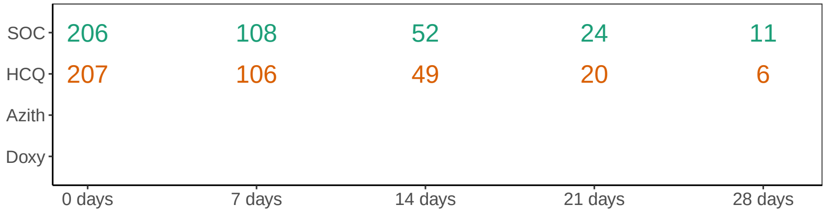

Recovery Inferences

	Pr(Superiority)	Pr(Meaningful Effect)	Pr(Best)	Randomization Probability
SOC				1
HCQ			0	0
Azith			0	0
Doxy			0	0

Hospitalization Inferences

Pr(Superiority)	Pr(Meaningful Effect)

Hazard Ratio Estimates

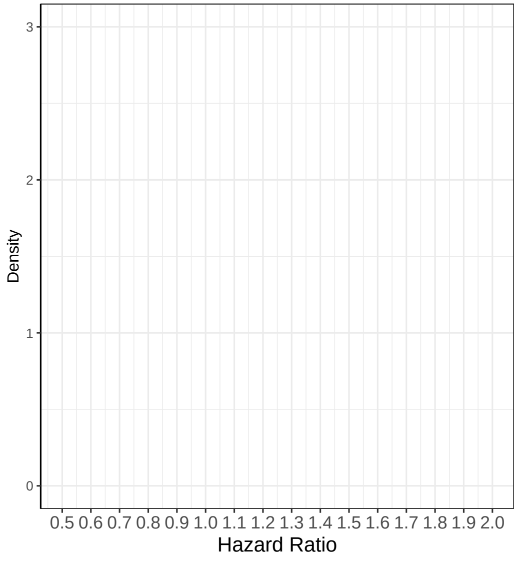

Median Time To Recovery Estimates

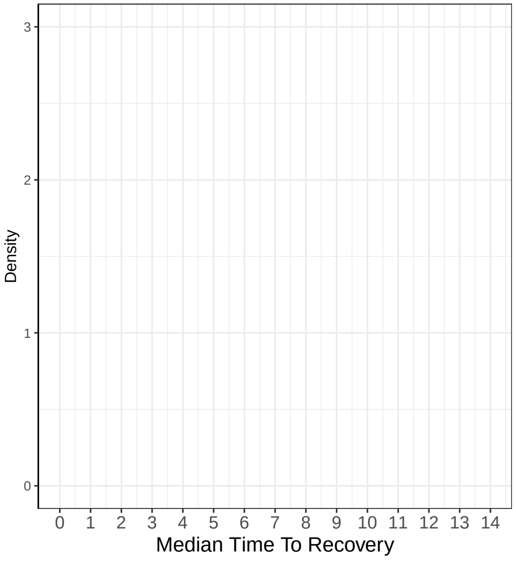

Add Drug on May 23, 2020

Recovery Data

	Enrolled	Complete	Recovered	Exposure Days	Recoveries Per Day	Estimated Hazard	Estimated HR (95% interval)	Estimated Median Time to Recovery
SOC	208	151	140	1866	0.075			
HCQ	207	154	148	1793	0.083			
Azith	0	0	0	0				
Doxy	0	0	0	0				
Total	415	305	288	3659	0.079			

Hospitalization Data

Hospitalizations	28 Day Completers	Observed Hosp. Rate	Est. Hosp. Rate (95% interval)
1	80	0.0125	
10	80	0.1250	
0	0	NA	
0	0	NA	
11	160	0.0688	

Intervention Status
Enrolling
Paused
Introducing
Unopened

Observed Patient Recoveries

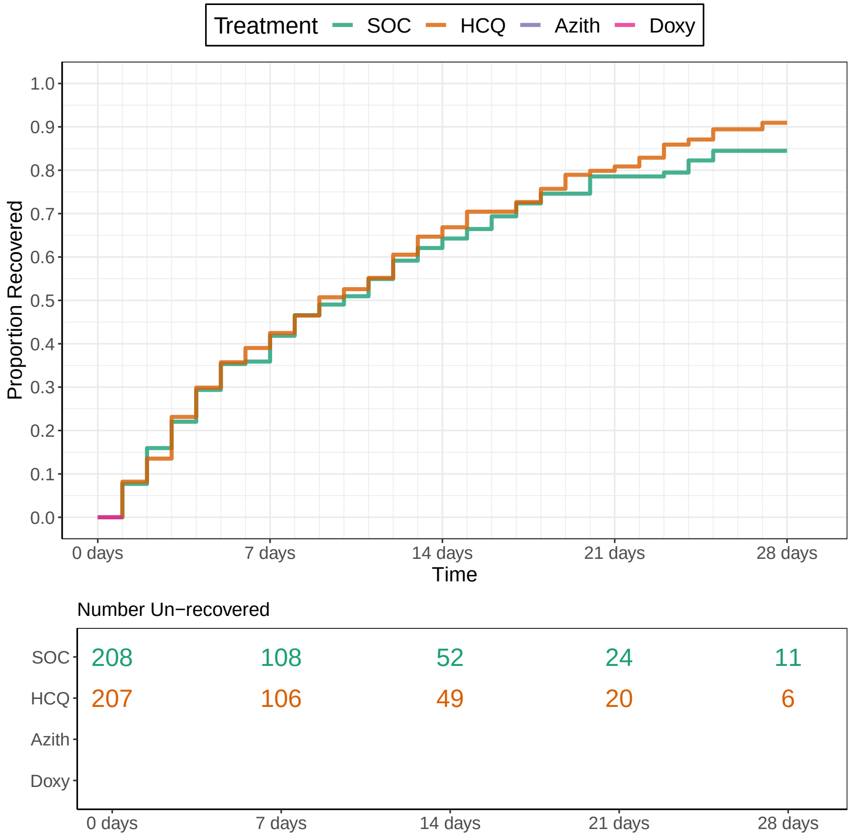

Recovery Inferences

	Pr(Superiority)	Pr(Meaningful Effect)	Pr(Best)	Randomization Probability
SOC				0.5
HCQ			0	0
Azith			1	0.5
Doxy			0	0

Hospitalization Inferences

Pr(Superiority)	Pr(Meaningful Effect)

Hazard Ratio Estimates

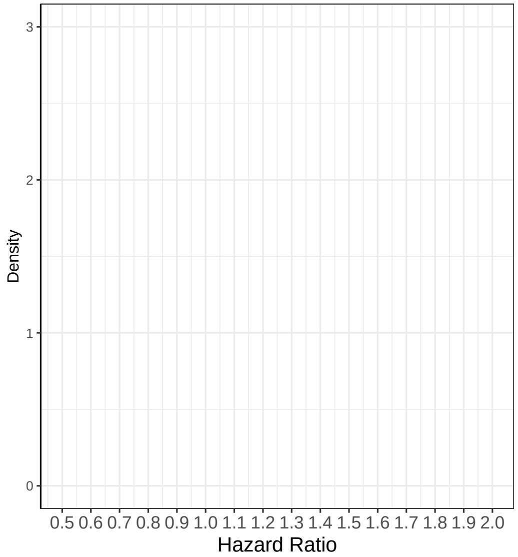

Median Time To Recovery Estimates

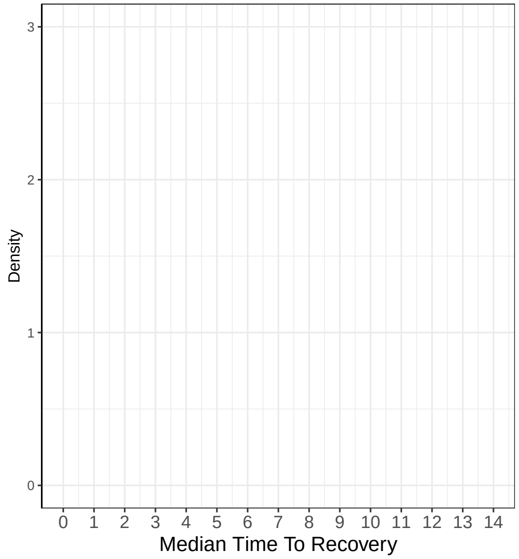

Add Drug on July 23, 2020

Recovery Data

	Enrolled	Complete	Recovered	Exposure Days	Recoveries Per Day	Estimated Hazard	Estimated HR (95% interval)	Estimated Median Time to Recovery
SOC	326	308	270	3608	0.075			
HCQ	207	207	181	2375	0.076			
Azith	118	106	97	1148	0.084			
Doxy	0	0	0	0				
Total	651	621	548	7132	0.077			

Hospitalization Data

Hospitalizations	28 Day Completers	Observed Hosp. Rate	Est. Hosp. Rate (95% interval)
7	285	0.0246	
10	207	0.0483	
4	77	0.0519	
0	0	NA	
21	569	0.0369	

Intervention Status
Enrolling
Paused
Introducing
Introducing

Observed Patient Recoveries

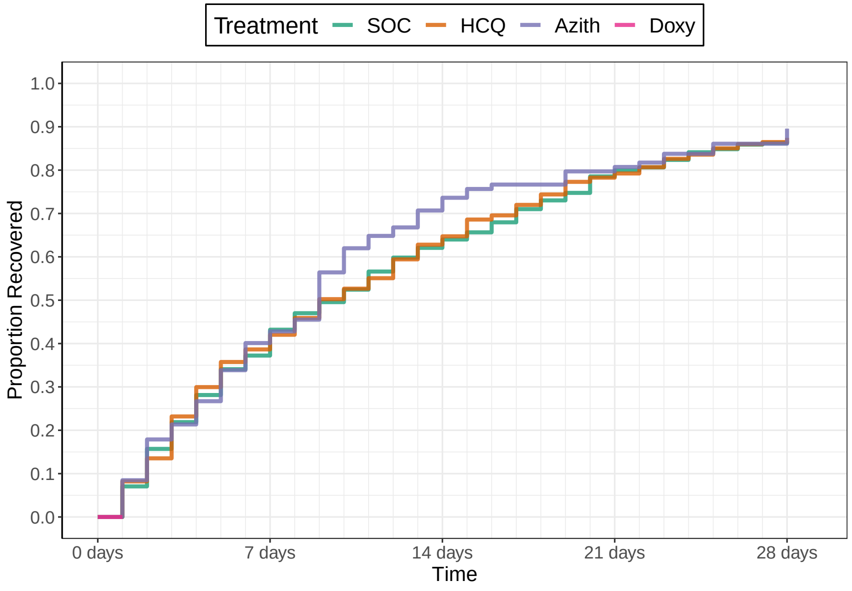

Number Un−recovered

	0 days	7 days	14 days	21 days	28 days
SOC	326	200	118	62	39
HCQ	207	127	77	45	28
Azith	118	67	30	20	12
Doxy					

Recovery Inferences

	Pr(Superiority)	Pr(Meaningful Effect)	Pr(Best)	Randomization Probability
SOC				0.33
HCQ			0	0
Azith			1	0.33
Doxy			0	0.33

Hospitalization Inferences

Pr(Superiority)	Pr(Meaningful Effect)

Hazard Ratio Estimates

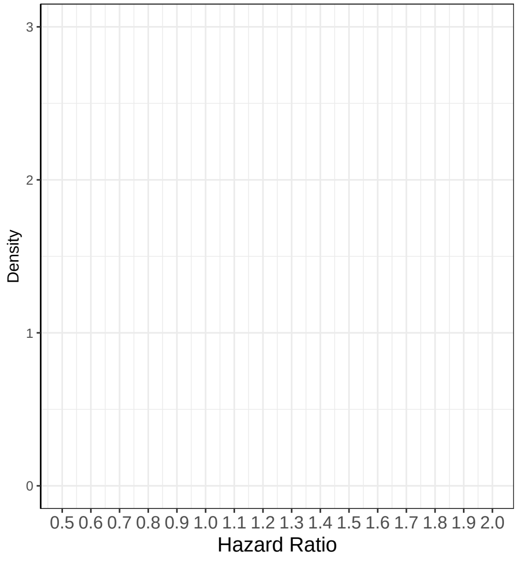

Median Time To Recovery Estimates

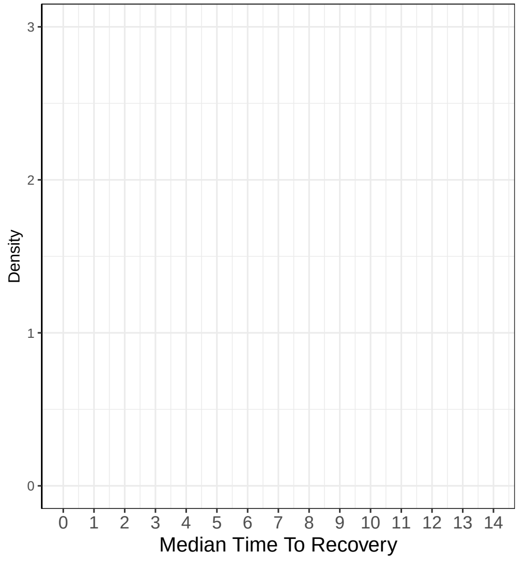

Interim 1 on October 15, 2020

Recovery Data

	Enrolled	Complete	Recovered	Exposure Days	Recoveries Per Day	Estimated Hazard	Estimated HR (95% interval)	Estimated Median Time to Recovery
SOC	515	461	415	5407	0.077	0.074	1	9.22
HCQ	207	207	181	2375	0.076	0.073	0.985 (0.835, 1.162)	9.36
Azith	309	256	234	2846	0.082	0.077	1.045 (0.907, 1.206)	8.81
Doxy	179	138	133	1309	0.102	0.092	1.246 (1.029, 1.497)	7.35
Total	1210	1062	963	11937	0.081			

Hospitalization Data

	Hospitalizations	28 Day Completers	Observed Hosp. Rate	Est. Hosp. Rate (95% interval)
SOC	19	400	0.0475	0.0387 (0.0238, 0.0573)
HCQ	10	207	0.0483	0.0528 (0.0274, 0.0866)
Azith	6	192	0.0312	0.0223 (0.0089, 0.0407)
Doxy	12	74	0.1622	0.0713 (0.0387, 0.1129)
Total	47	873	0.0538	

	Intervention Status
SOC	Enrolling
HCQ	Futility
Azith	Enrolling
Doxy	Enrolling
Total	

Observed Patient Recoveries

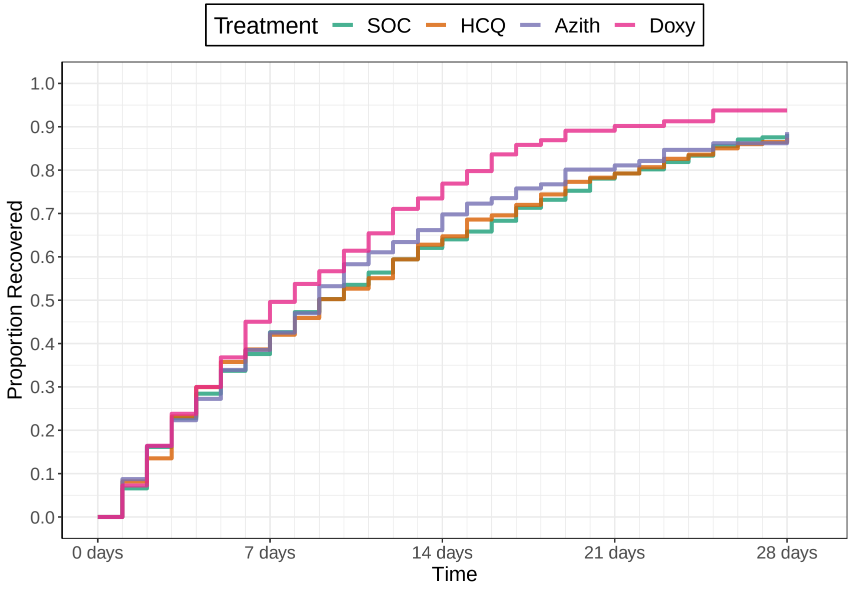

Number Un−recovered

	0 days	7 days	14 days	21 days	28 days
SOC	515	297	173	93	49
HCQ	207	127	77	45	28
Azith	309	169	84	41	27
Doxy	179	84	31	10	5

Recovery Inferences

	Pr(Superiority)	Pr(Meaningful Effect)	Pr(Best)	Randomization Probability
SOC				0.33
HCQ	0.4313	0.0207	0	0
Azith	0.7207	0.057	0.0362	0.28
Doxy	0.9853	0.6817	0.7138	0.39

Hospitalization Inferences

	Pr(Superiority)	Pr(Meaningful Effect)
SOC		
HCQ	0.2122	0.0148
Azith	0.9192	0.3777
Doxy	0.0478	0.0018

Hazard Ratio Estimates

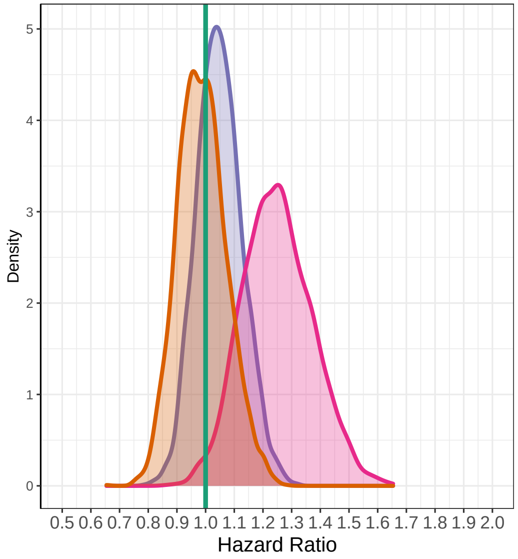

Median Time To Recovery Estimates

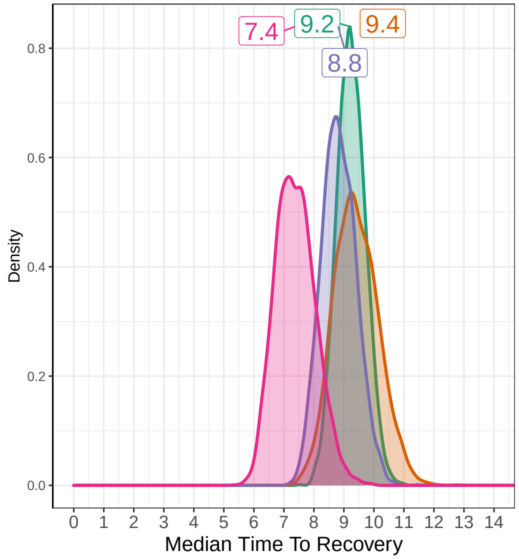

Interim 2 on November 12, 2020

Recovery Data

	Enrolled	Complete	Recovered	Exposure Days	Recoveries Per Day	Estimated Hazard	Estimated HR (95% interval)	Estimated Median Time to Recovery
SOC	593	575	512	6654	0.077	0.074	1	9.23
HCQ	207	207	181	2375	0.076	0.073	0.981 (0.83, 1.152)	9.43
Azith	375	351	321	3888	0.083	0.078	1.053 (0.923, 1.206)	8.77
Doxy	273	243	233	2182	0.107	0.098	1.323 (1.143, 1.53)	7.01
Total	1448	1376	1247	15100	0.083			

Hospitalization Data

Hospitalizations	28 Day Completers	Observed Hosp. Rate	Est. Hosp. Rate (95% interval)
22	515	0.0427	0.0387 (0.0251, 0.0548)
10	207	0.0483	0.0526 (0.0266, 0.0875)
7	309	0.0227	0.0212 (0.0092, 0.0381)
14	179	0.0782	0.054 (0.0308, 0.0841)
53	1210	0.0438	

Intervention Status
LostOnTTR
Dropped
InferiorToDoxy
SuccessOnTTRFutilityOnHosp *Announced*

Observed Patient Recoveries

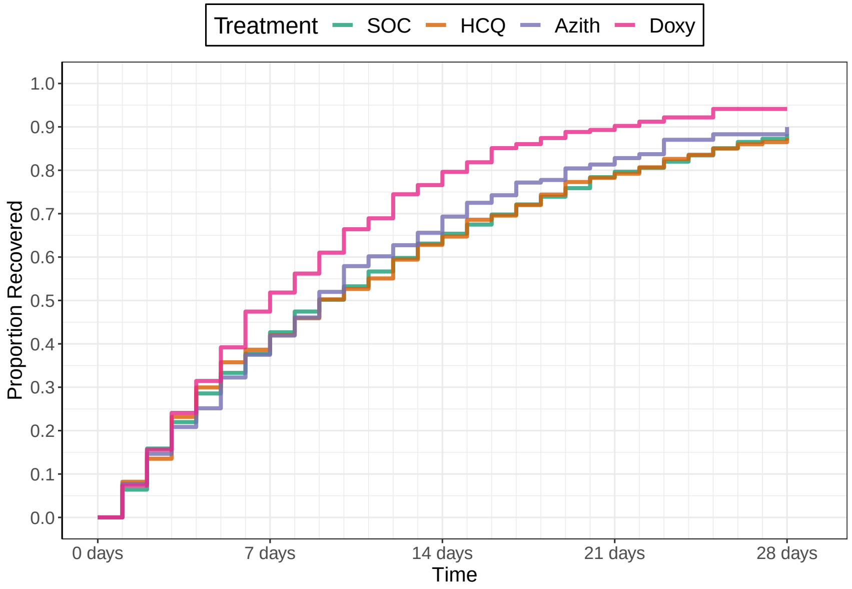

Number Un−recovered

	0 days	7 days	14 days	21 days	28 days
SOC	593	365	210	120	69
HCQ	207	127	77	45	28
Azith	375	226	120	63	35
Doxy	273	132	54	23	12

Recovery Inferences

	Pr(Superiority)	Pr(Meaningful Effect)	Pr(Best)	Randomization Probability
SOC				0.5
HCQ	0.4057	0.0127	0	0
Azith	0.772	0.0437	0	0
Doxy	0.9997	0.9037	0.75	0.5

Hospitalization Inferences

Pr(Superiority)	Pr(Meaningful Effect)
0.2158	0.0158
0.9422	0.4105
0.1645	0.0045

Hazard Ratio Estimates

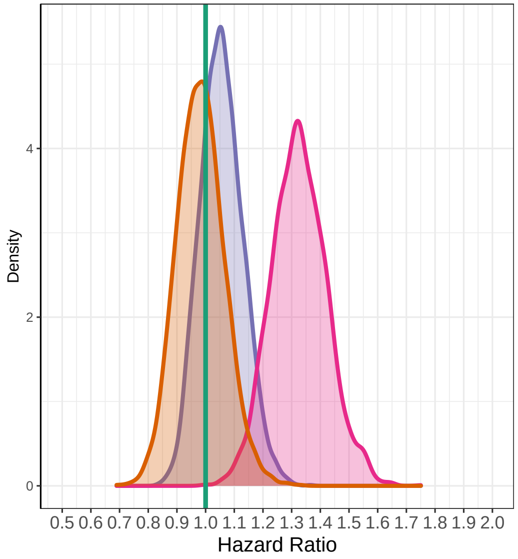

Median Time To Recovery Estimates

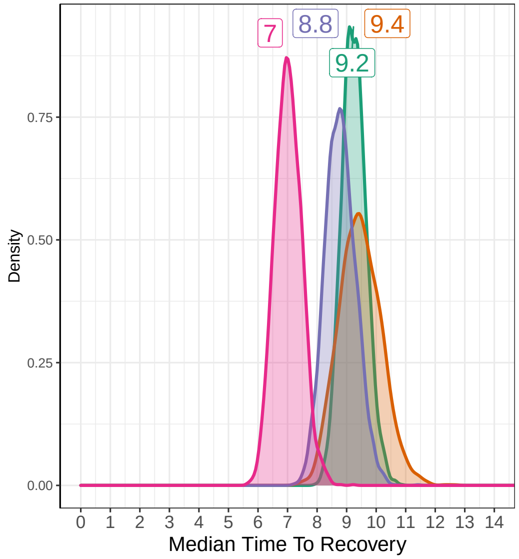

Interim 3 on August 30, 2022

Recovery Data

	Enrolled	Complete	Recovered	Exposure Days	Recoveries Per Day	Estimated Hazard	Estimated HR (95% interval)	Estimated Median Time to Recovery
SOC	2851	2851	2499	33636	0.074	0.072	1	9.69
HCQ	207	207	181	2375	0.076	0.073	1.021 (0.876, 1.173)	9.5
Azith	375	375	339	4072	0.083	0.08	1.11 (0.991, 1.238)	8.75
Doxy	2567	2567	2417	23633	0.102	0.097	1.354 (1.282, 1.431)	7.16
Total	6000	6000	5436	63716	0.085			

Hospitalization Data

Hospitalizations	28 Day Completers	Observed Hosp. Rate	Est. Hosp. Rate (95% interval)
83	2851	0.0291	0.0294 (0.0235, 0.0362)
10	207	0.0483	0.053 (0.0272, 0.0879)
7	375	0.0187	0.0212 (0.0093, 0.0381)
62	2567	0.0242	0.0246 (0.0188, 0.0306)
162	6000	0.0270	

Intervention Status
Complete
Dropped
Dropped
SuccessOnTTRFutilityOnHosp *Announced*

Observed Patient Recoveries

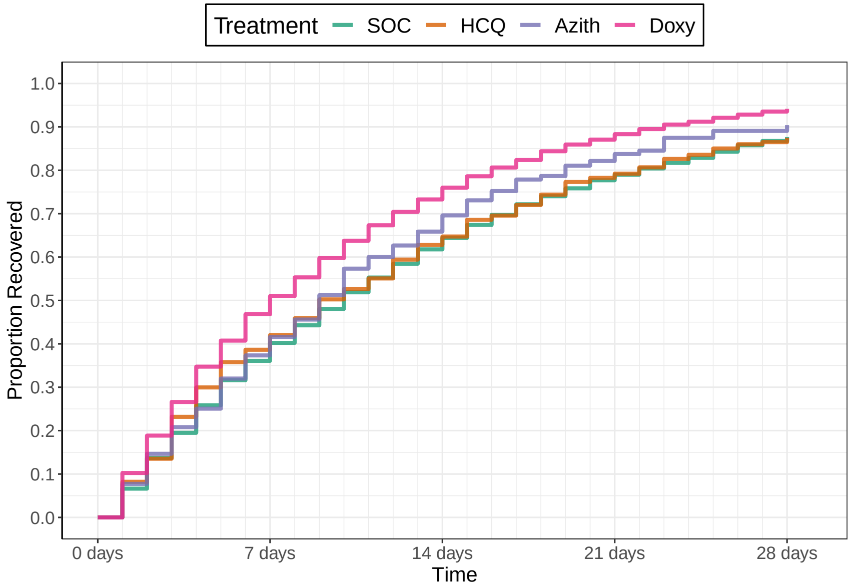

Number Un−recovered

	0 days	7 days	14 days	21 days	28 days
SOC	2851	1822	1090	636	378
HCQ	207	127	77	45	28
Azith	375	235	128	67	41
Doxy	2567	1365	686	332	166

Recovery Inferences

	Pr(Superiority)	Pr(Meaningful Effect)	Pr(Best)	Randomization Probability
SOC				
HCQ	0.6043	0.021	0	
Azith	0.966	0.1283	0	
Doxy	1	1	0.75	

Hospitalization Inferences

Pr(Superiority)	Pr(Meaningful Effect)
0.0495	0
0.8518	0.056
0.866	2e−04

Hazard Ratio Estimates

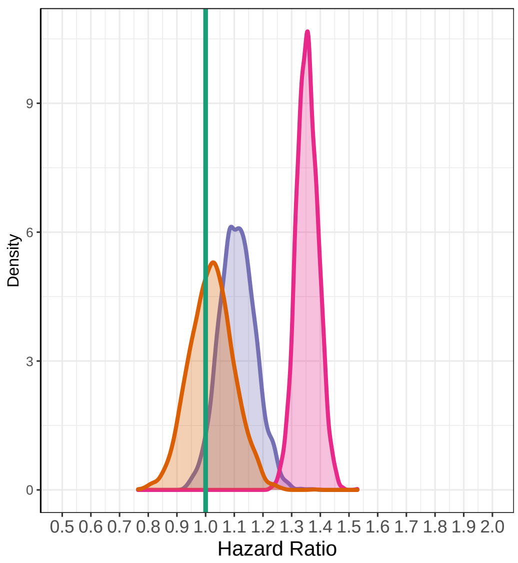

Median Time To Recovery Estimates

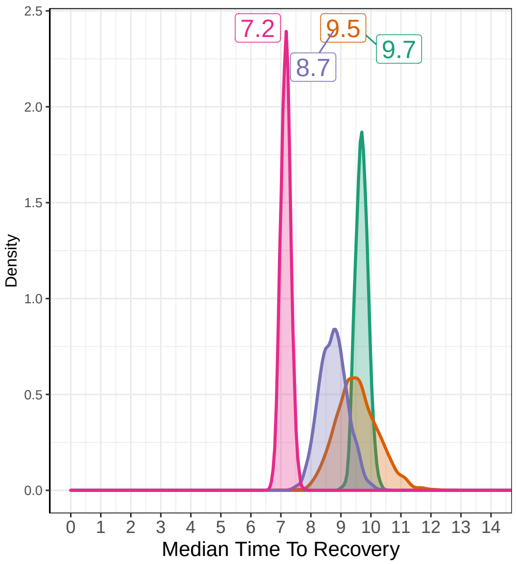

Berry Consultants
Statistical Innovation

6.4 Example 3

April 1, 2020

Before Patient Enrollment Begins

Recovery Data

	Enrolled	Complete	Recovered	Exposure Days	Recoveries Per Day	Estimated Hazard	Estimated HR (95% interval)	Estimated Median Survival Time
SOC	0	0	0	0				
HCQ	0	0	0	0				
Azith	0	0	0	0				
Doxy	0	0	0	0				
Total	0	0	0	0				

Hospitalization Data

Hospitalizations	28 Day Completers	Observed Hosp. Rate	Est. Hosp. Rate (95% interval)
0	0		
0	0		
0	0		
0	0		
0	0		

Intervention Status
Enrolling
Enrolling
Unopened
Unopened

Observed Patient Recoveries

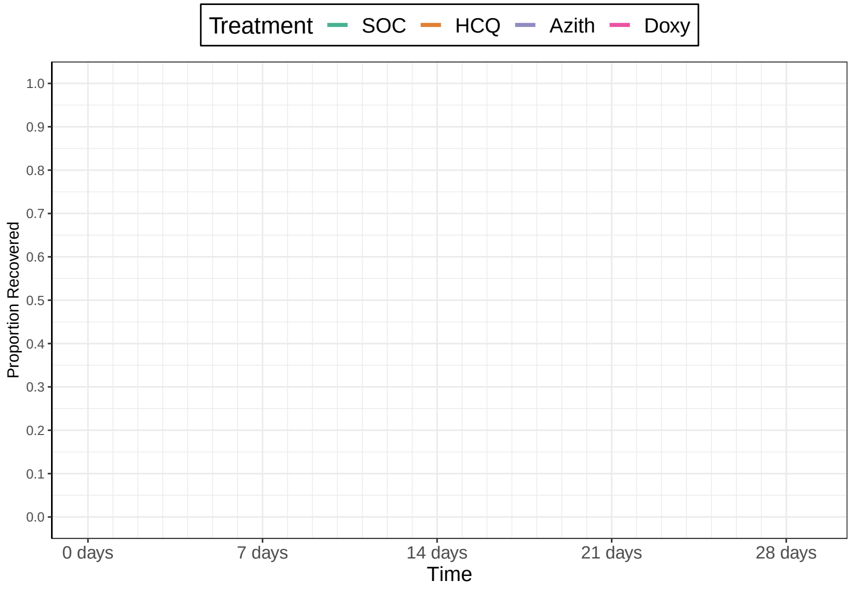

Number Un−recovered

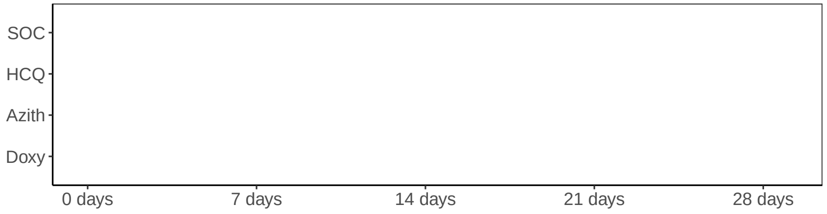

Recovery Inferences

	Pr(Superiority)	Pr(Meaningful Effect)	Pr(Best)	Randomization Probability
SOC				0.5
HCQ				0.5
Azith				0
Doxy				0

Hospitalization Inferences

Pr(Superiority)	Pr(Meaningful Effect)
0	0
0	0
0	0
0	0

Hazard Ratio Estimates

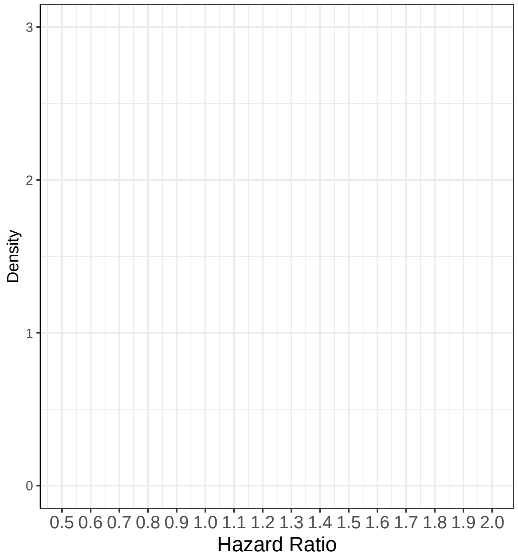

Median Time To Recovery Estimates

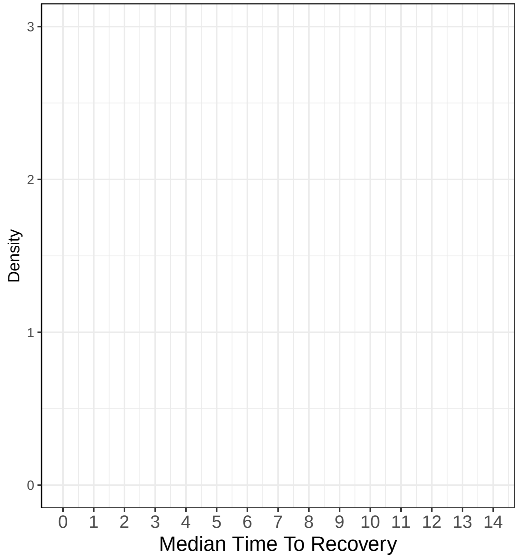

Remove Drug on May 22, 2020

Recovery Data

	Enrolled	Complete	Recovered	Exposure Days	Recoveries Per Day	Estimated Hazard	Estimated HR (95% interval)	Estimated Median Time to Recovery
SOC	206	150	141	1753	0.08			
HCQ	207	156	147	1684	0.087			
Azith	0	0	0	0				
Doxy	0	0	0	0				
Total	413	306	288	3437	0.084			

Hospitalization Data

Hospitalizations	28 Day Completers	Observed Hosp. Rate	Est. Hosp. Rate (95% interval)
5	78	0.0641	
7	79	0.0886	
0	0	NA	
0	0	NA	
12	157	0.0764	

Intervention Status
Enrolling
Removing
Unopened
Unopened

Observed Patient Recoveries

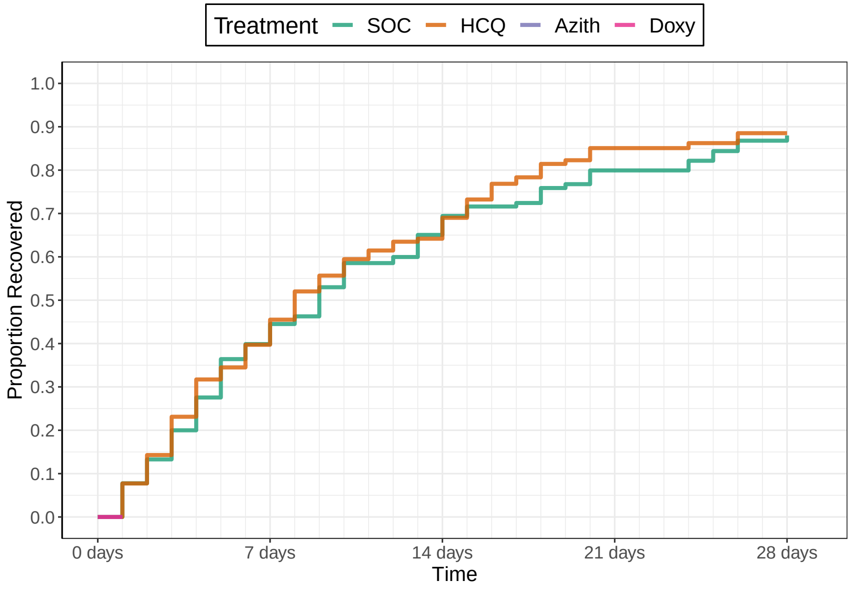

Number Un−recovered

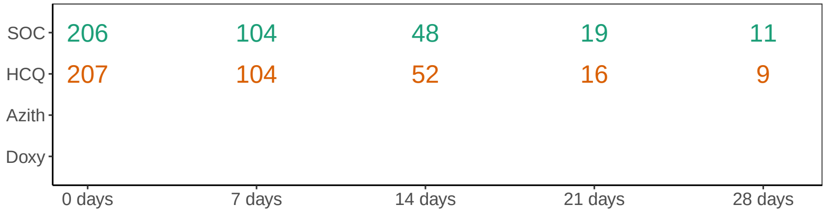

Recovery Inferences

	Pr(Superiority)	Pr(Meaningful Effect)	Pr(Best)	Randomization Probability
SOC				1
HCQ			0	0
Azith			0	0
Doxy			0	0

Hospitalization Inferences

Pr(Superiority)	Pr(Meaningful Effect)

Hazard Ratio Estimates

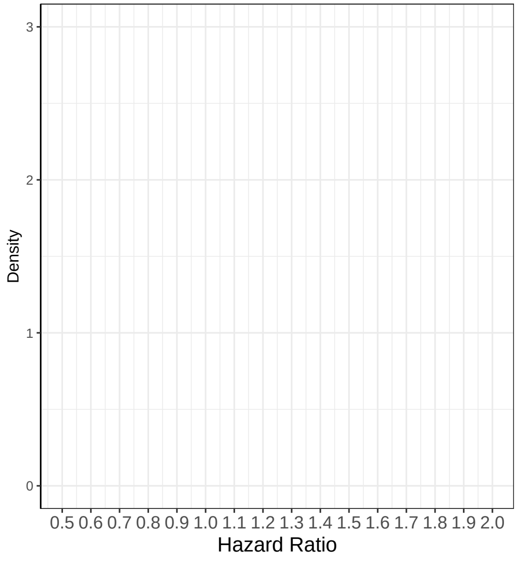

Median Time To Recovery Estimates

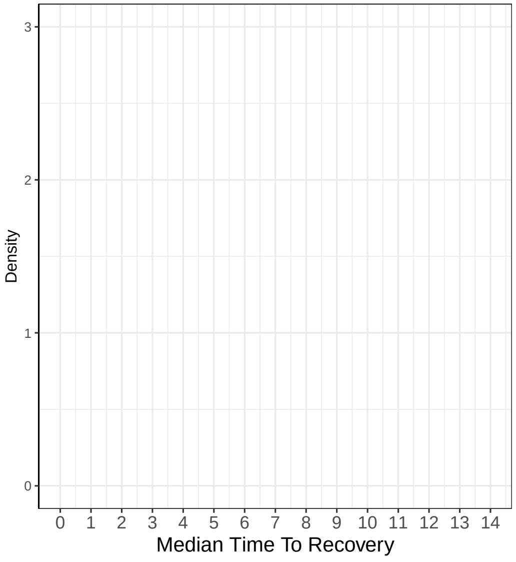

Add Drug on May 23, 2020

Recovery Data

	Enrolled	Complete	Recovered	Exposure Days	Recoveries Per Day	Estimated Hazard	Estimated HR (95% interval)	Estimated Median Time to Recovery
SOC	208	155	145	1808	0.08			
HCQ	207	166	157	1728	0.091			
Azith	0	0	0	0				
Doxy	0	0	0	0				
Total	415	321	302	3536	0.085			

Hospitalization Data

Hospitalizations	28 Day Completers	Observed Hosp. Rate	Est. Hosp. Rate (95% interval)
5	80	0.0625	
7	80	0.0875	
0	0	NA	
0	0	NA	
12	160	0.0750	

Intervention Status
Enrolling
Paused
Introducing
Unopened

Observed Patient Recoveries

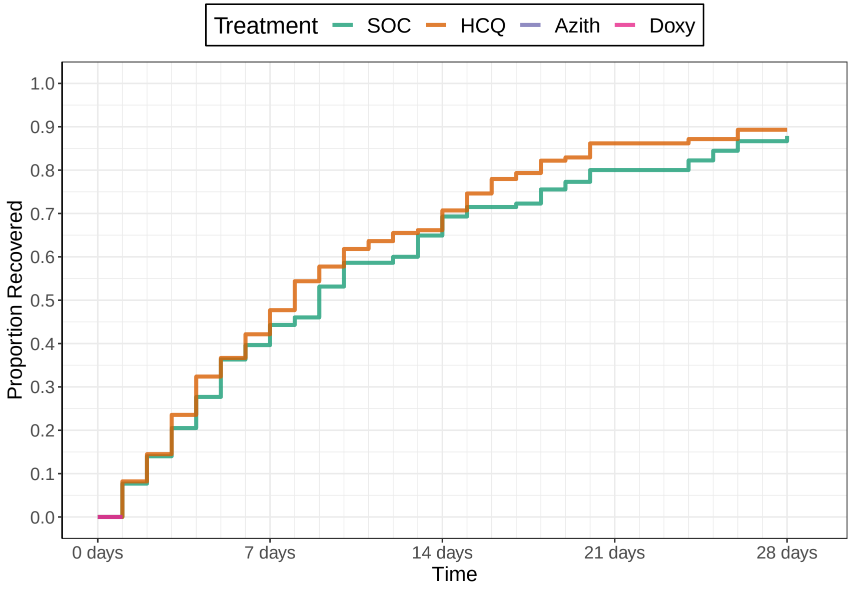

Number Un−recovered

	0 days	7 days	14 days	21 days	28 days
SOC	208	104	48	19	11
HCQ	207	104	52	16	9
Azith					
Doxy					

Recovery Inferences

	Pr(Superiority)	Pr(Meaningful Effect)	Pr(Best)	Randomization Probability
SOC				0.5
HCQ			0	0
Azith			1	0.5
Doxy			0	0

Hospitalization Inferences

Pr(Superiority)	Pr(Meaningful Effect)

Hazard Ratio Estimates

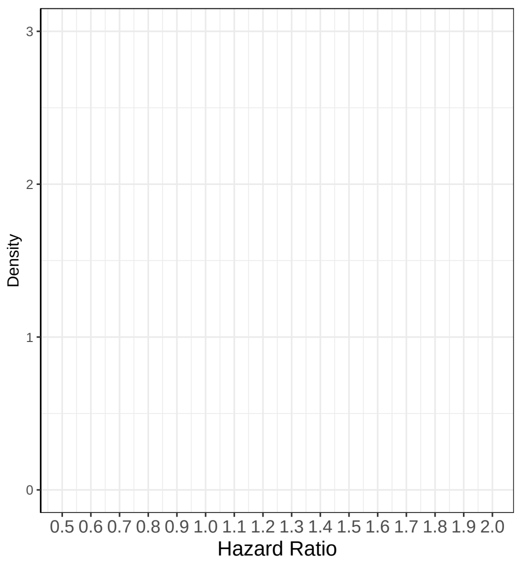

Median Time To Recovery Estimates

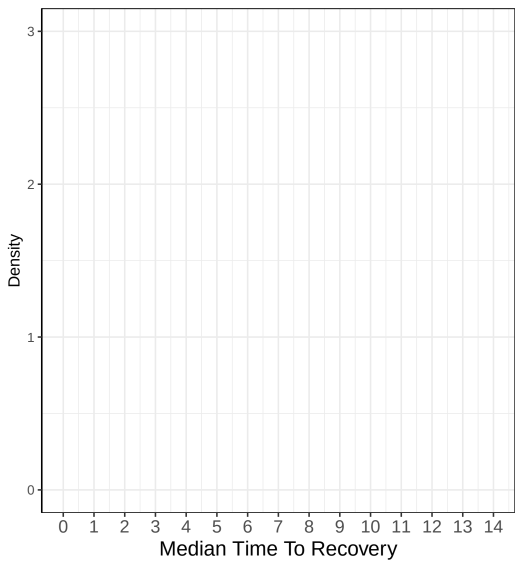

Add Drug on July 23, 2020

Recovery Data

	Enrolled	Complete	Recovered	Exposure Days	Recoveries Per Day	Estimated Hazard	Estimated HR (95% interval)	Estimated Median Time to Recovery
SOC	326	305	275	3462	0.079			
HCQ	207	207	188	2127	0.088			
Azith	118	105	97	1041	0.093			
Doxy	0	0	0	0				
Total	651	617	560	6630	0.084			

Hospitalization Data

Hospitalizations	28 Day Completers	Observed Hosp. Rate	Est. Hosp. Rate (95% interval)
6	285	0.0211	
7	207	0.0338	
3	77	0.0390	
0	0	NA	
16	569	0.0281	

Intervention Status
Enrolling
Paused
Introducing
Introducing

Observed Patient Recoveries

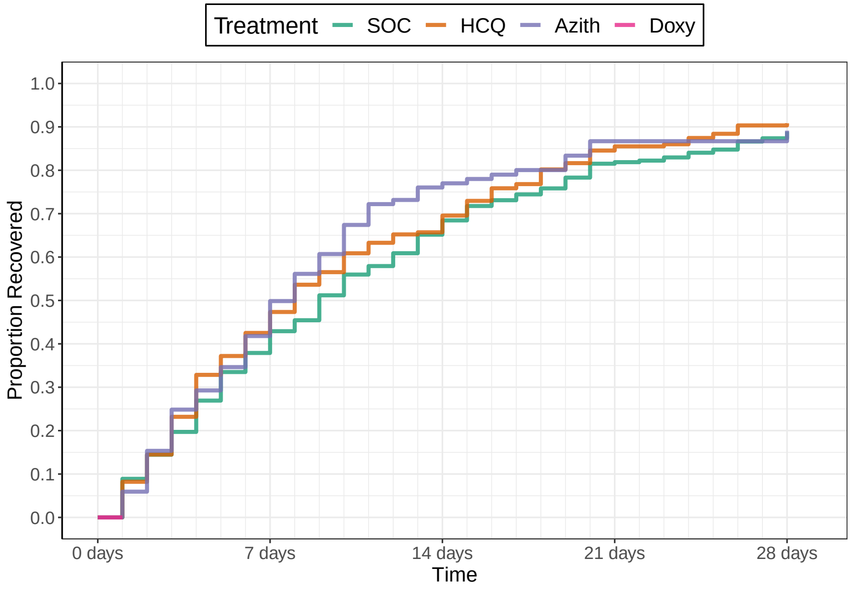

Number Un−recovered

	0 days	7 days	14 days	21 days	28 days
SOC	326	198	106	52	34
HCQ	207	119	71	32	20
Azith	118	65	25	12	11
Doxy					

Recovery Inferences

	Pr(Superiority)	Pr(Meaningful Effect)	Pr(Best)	Randomization Probability
SOC				0.33
HCQ			0	0
Azith			1	0.33
Doxy			0	0.33

Hospitalization Inferences

Pr(Superiority)	Pr(Meaningful Effect)

Hazard Ratio Estimates

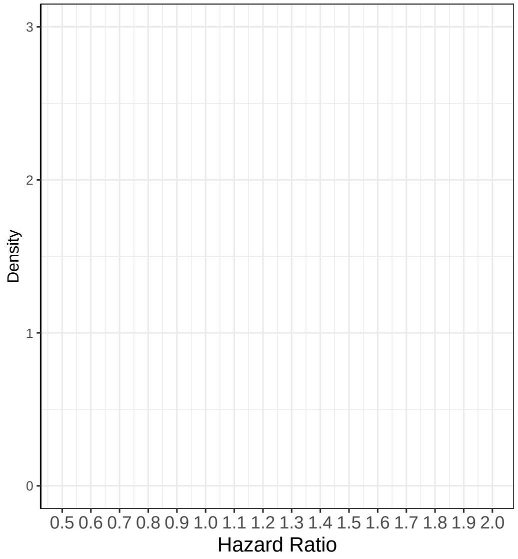

Median Time To Recovery Estimates

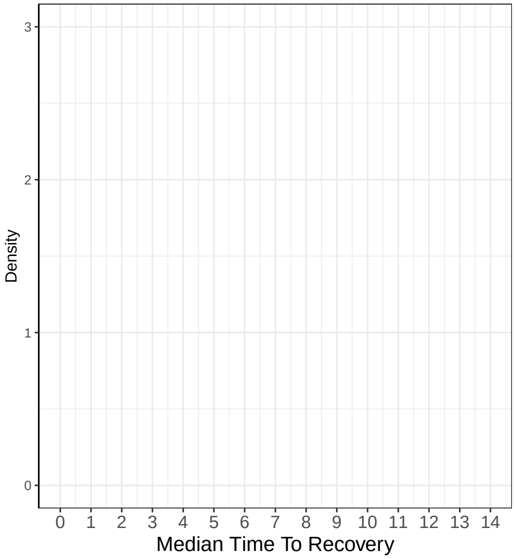

Interim 1 on October 15, 2020

Recovery Data

	Enrolled	Complete	Recovered	Exposure Days	Recoveries Per Day	Estimated Hazard	Estimated HR (95% interval)	Estimated Median Time to Recovery
SOC	519	477	433	5488	0.079	0.073	1	8.96
HCQ	207	207	188	2127	0.088	0.08	1.098 (0.937, 1.295)	8.18
Azith	317	262	242	2746	0.088	0.079	1.083 (0.928, 1.255)	8.28
Doxy	167	118	108	1301	0.083	0.075	1.019 (0.831, 1.239)	8.79
Total	1210	1064	971	11662	0.083			

Hospitalization Data

Hospitalizations	28 Day Completers	Observed Hosp. Rate	Est. Hosp. Rate (95% interval)
14	419	0.0334	0.0287 (0.0162, 0.0445)
7	207	0.0338	0.038 (0.0165, 0.0671)
9	185	0.0486	0.0314 (0.0155, 0.0526)
6	62	0.0968	0.0409 (0.017, 0.0745)
36	873	0.0412	

Intervention Status
Enrolling
Futility
Enrolling
Enrolling

Observed Patient Recoveries

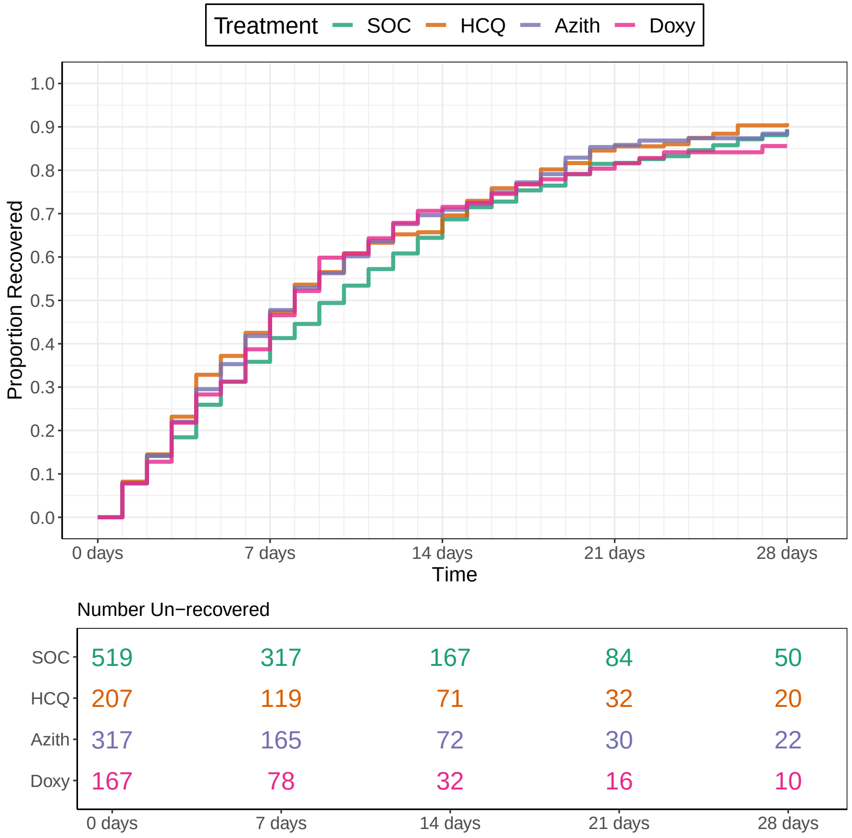

Recovery Inferences

	Pr(Superiority)	Pr(Meaningful Effect)	Pr(Best)	Randomization Probability
SOC				0.33
HCQ	0.8727	0.1377	0	0
Azith	0.8553	0.0917	0.525	0.4
Doxy	0.5723	0.0587	0.225	0.27

Hospitalization Inferences

Pr(Superiority)	Pr(Meaningful Effect)
0.2815	0.0148
0.4105	0.0232
0.2388	0.0127

Hazard Ratio Estimates

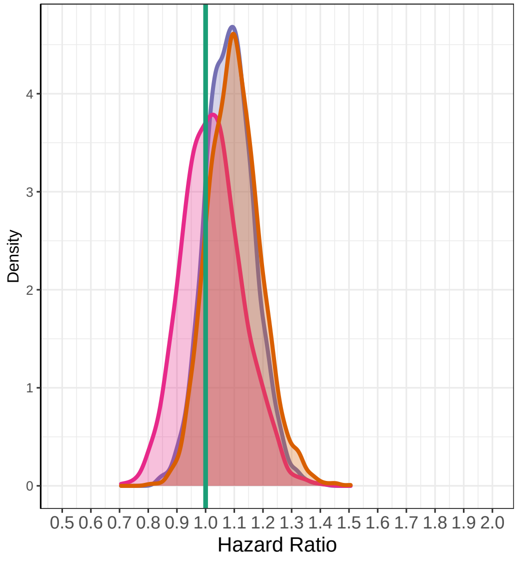

Median Time To Recovery Estimates

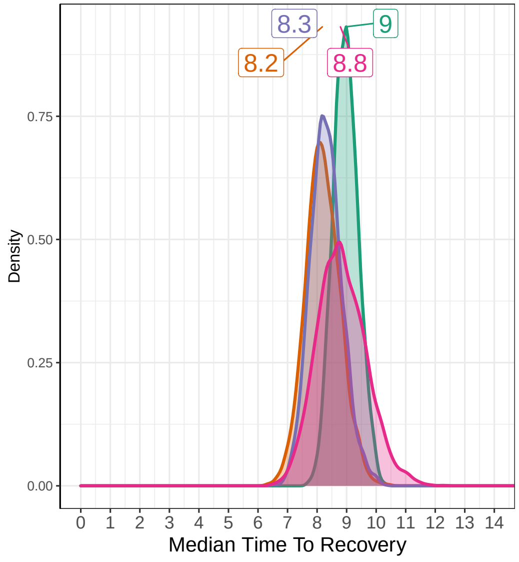

Interim 2 on November 12, 2020

Recovery Data

	Enrolled	Complete	Recovered	Exposure Days	Recoveries Per Day	Estimated Hazard	Estimated HR (95% interval)	Estimated Median Time to Recovery
SOC	605	574	522	6588	0.079	0.076	1	9.04
HCQ	207	207	188	2127	0.088	0.083	1.094 (0.932, 1.284)	8.3
Azith	417	392	362	3924	0.092	0.086	1.139 (1.001, 1.305)	7.98
Doxy	227	204	187	2176	0.086	0.08	1.065 (0.911, 1.25)	8.5
Total	1456	1377	1259	14815	0.085			

Hospitalization Data

Hospitalizations	28 Day Completers	Observed Hosp. Rate	Est. Hosp. Rate (95% interval)
18	519	0.0347	0.0311 (0.0189, 0.0462)
7	207	0.0338	0.0382 (0.0162, 0.0686)
12	317	0.0379	0.0309 (0.0164, 0.0497)
9	167	0.0539	0.0436 (0.0213, 0.0734)
46	1210	0.0380	

Intervention Status
Enrolling
Dropped
Enrolling
Enrolling

Observed Patient Recoveries

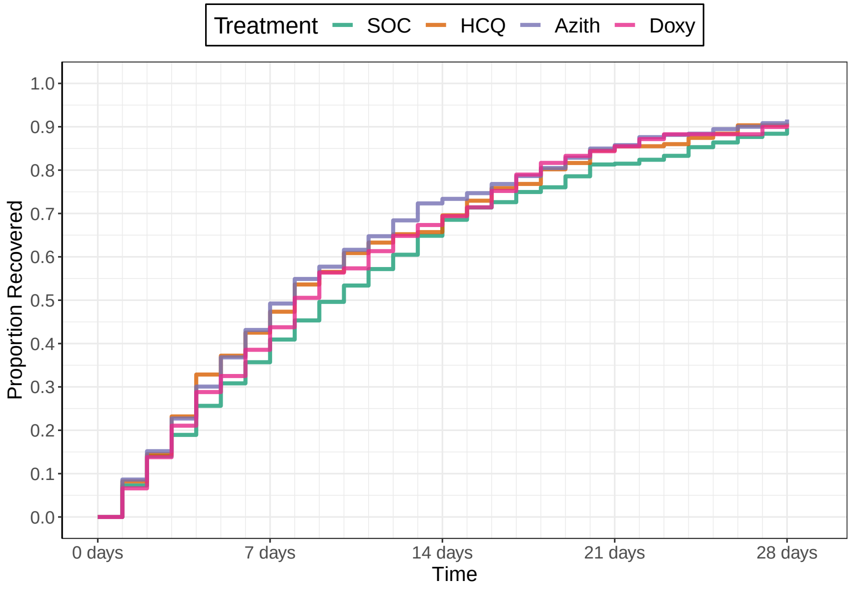

Number Un−recovered

	0 days	7 days	14 days	21 days	28 days
SOC	605	381	200	103	62
HCQ	207	119	71	32	20
Azith	417	224	106	57	33
Doxy	227	130	64	28	18

Recovery Inferences

	Pr(Superiority)	Pr(Meaningful Effect)	Pr(Best)	Randomization Probability
SOC				0.33
HCQ	0.8647	0.133	0	0
Azith	0.9757	0.22	0.5913	0.37
Doxy	0.7733	0.077	0.1588	0.29

Hospitalization Inferences

Pr(Superiority)	Pr(Meaningful Effect)
0.3355	0.018
0.5128	0.029
0.205	0.0102

Hazard Ratio Estimates

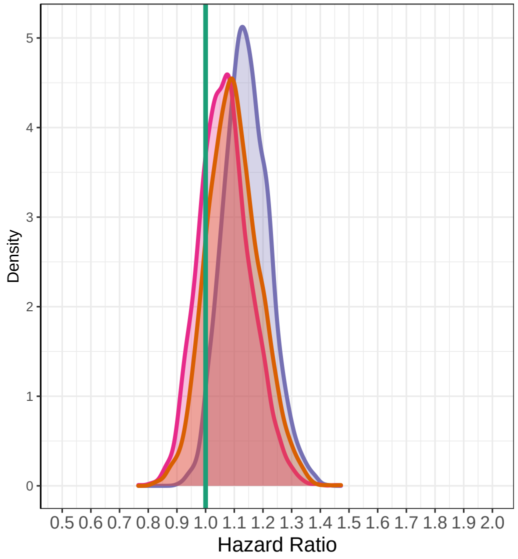

Median Time To Recovery Estimates

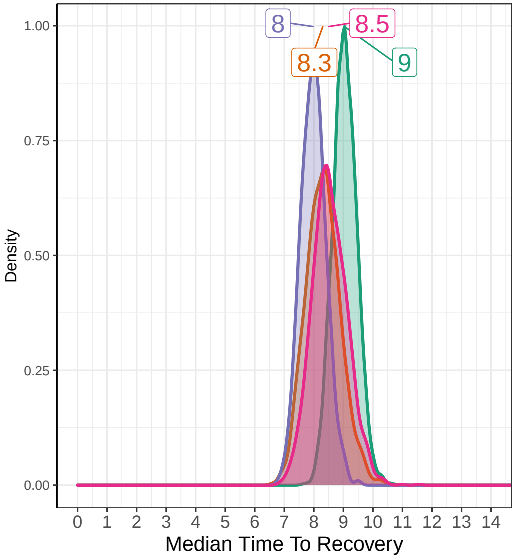

Interim 3 on December 10, 2020

Recovery Data

	Enrolled	Complete	Recovered	Exposure Days	Recoveries Per Day	Estimated Hazard	Estimated HR (95% interval)	Estimated Median Time to Recovery
SOC	668	636	573	7328	0.078	0.075	1	9.14
HCQ	207	207	188	2127	0.088	0.083	1.107 (0.945, 1.296)	8.29
Azith	472	453	419	4606	0.091	0.086	1.136 (1.01, 1.286)	8.07
Doxy	289	264	245	2946	0.083	0.079	1.042 (0.904, 1.204)	8.76
Total	1636	1560	1425	17008	0.084			

Hospitalization Data

Hospitalizations	28 Day Completers	Observed Hosp. Rate	Est. Hosp. Rate (95% interval)
22	605	0.0364	0.0344 (0.0222, 0.0488)
7	207	0.0338	0.0388 (0.0169, 0.0687)
15	417	0.0360	0.0337 (0.0194, 0.0518)
9	227	0.0396	0.0341 (0.0168, 0.0574)
53	1456	0.0364	

Intervention Status
Enrolling
Dropped
Enrolling
Futility

Observed Patient Recoveries

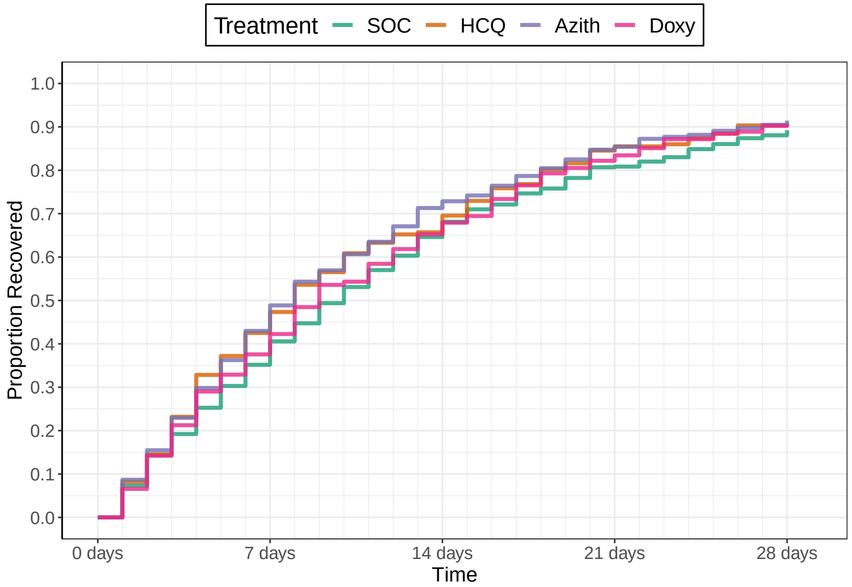

Number Un−recovered

	0 days	7 days	14 days	21 days	28 days
SOC	668	421	222	118	71
HCQ	207	119	71	32	20
Azith	472	262	129	68	40
Doxy	289	173	91	43	21

Recovery Inferences

	Pr(Superiority)	Pr(Meaningful Effect)	Pr(Best)	Randomization Probability
SOC				0.5
HCQ	0.8873	0.153	0	0
Azith	0.9803	0.2037	0.75	0.5
Doxy	0.7243	0.034	0	0

Hospitalization Inferences

Pr(Superiority)	Pr(Meaningful Effect)
0.4158	0.04
0.5435	0.0325
0.532	0.0452

Hazard Ratio Estimates

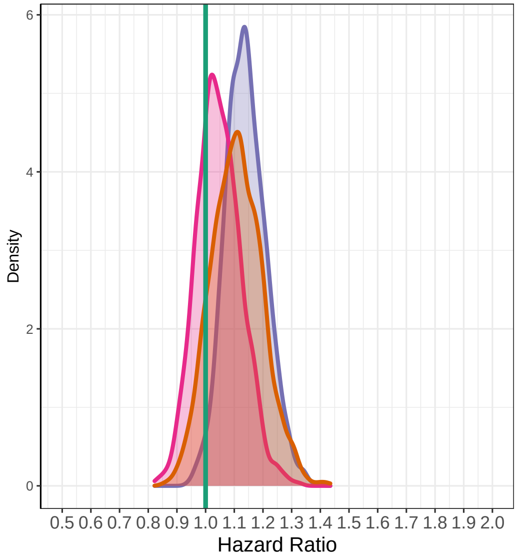

Median Time To Recovery Estimates

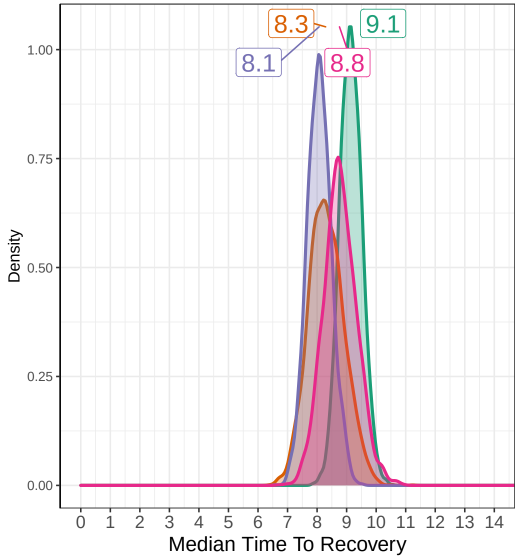

Interim 4 on January 07, 2021

Recovery Data

	Enrolled	Complete	Recovered	Exposure Days	Recoveries Per Day	Estimated Hazard	Estimated HR (95% interval)	Estimated Median Time to Recovery
SOC	770	724	650	8446	0.077	0.074	1	9.27
HCQ	207	207	188	2127	0.088	0.083	1.123 (0.964, 1.31)	8.27
Azith	583	540	497	5606	0.089	0.083	1.131 (1.008, 1.266)	8.23
Doxy	289	289	263	3158	0.083	0.079	1.069 (0.932, 1.219)	8.67
Total	1849	1760	1598	19337	0.083			

Hospitalization Data

	Hospitalizations	28 Day Completers	Observed Hosp. Rate	Est. Hosp. Rate (95% interval)
SOC	25	668	0.0374	0.0337 (0.0221, 0.047)
HCQ	7	207	0.0338	0.0384 (0.0173, 0.0679)
Azith	17	472	0.0360	0.0306 (0.0184, 0.046)
Doxy	9	289	0.0311	0.0344 (0.0162, 0.0593)
Total	58	1636	0.0355	

	Intervention Status
SOC	Enrolling
HCQ	Dropped
Azith	Enrolling
Doxy	Dropped
Total	

Observed Patient Recoveries

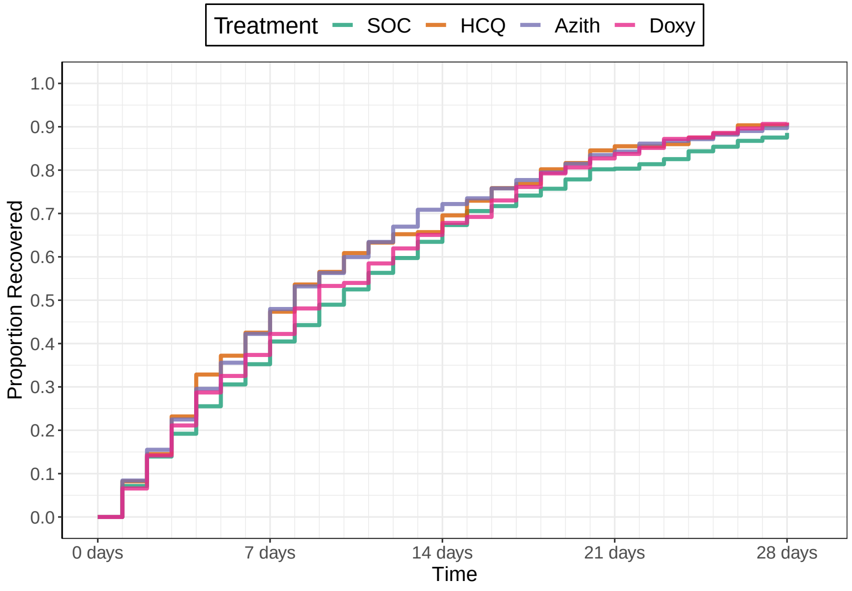

Number Un−recovered

	0 days	7 days	14 days	21 days	28 days
SOC	770	482	264	135	81
HCQ	207	119	71	32	20
Azith	583	322	155	82	48
Doxy	289	181	101	50	27

Recovery Inferences

	Pr(Superiority)	Pr(Meaningful Effect)	Pr(Best)	Randomization Probability
SOC				0.5
HCQ	0.9317	0.218	0	0
Azith	0.982	0.1753	0.75	0.5
Doxy	0.8383	0.0533	0	0

Hospitalization Inferences

	Pr(Superiority)	Pr(Meaningful Effect)
SOC		
HCQ	0.4008	0.032
Azith	0.6305	0.0352
Doxy	0.51	0.0415

Hazard Ratio Estimates

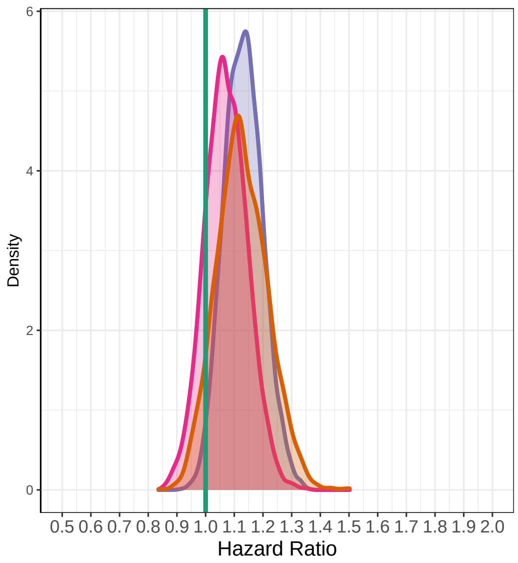

Median Time To Recovery Estimates

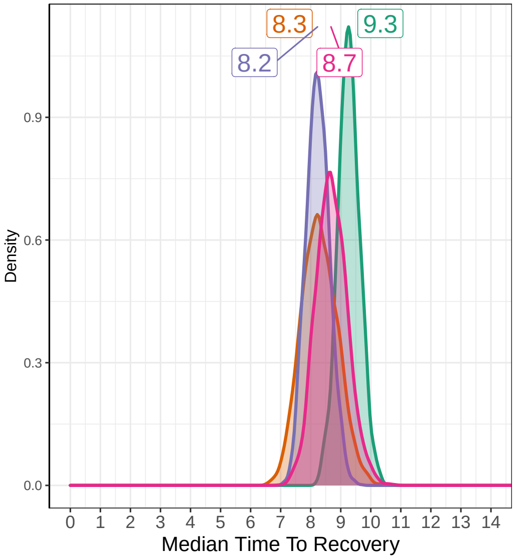

Interim 5 on February 04, 2021

Recovery Data

	Enrolled	Complete	Recovered	Exposure Days	Recoveries Per Day	Estimated Hazard	Estimated HR (95% interval)	Estimated Median Time to Recovery
SOC	881	832	747	9689	0.077	0.074	1	9.31
HCQ	207	207	188	2127	0.088	0.084	1.124 (0.958, 1.325)	8.31
Azith	682	643	590	6725	0.088	0.083	1.122 (1.005, 1.246)	8.34
Doxy	289	289	263	3158	0.083	0.079	1.069 (0.932, 1.219)	8.73
Total	2059	1971	1788	21700	0.082			

Hospitalization Data

Hospitalizations	28 Day Completers	Observed Hosp. Rate	Est. Hosp. Rate (95% interval)
28	770	0.0364	0.033 (0.0223, 0.0459)
7	207	0.0338	0.0383 (0.0167, 0.0678)
20	583	0.0343	0.0306 (0.019, 0.0447)
9	289	0.0311	0.0346 (0.017, 0.0578)
64	1849	0.0346	

Intervention Status
Enrolling
Dropped
Enrolling
Dropped

Observed Patient Recoveries

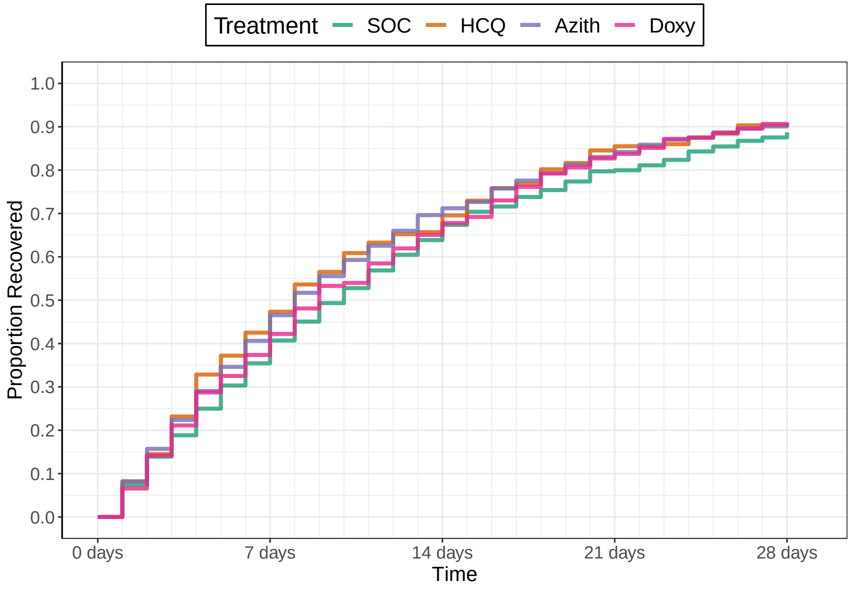

Number Un−recovered

	0 days	7 days	14 days	21 days	28 days
SOC	881	552	299	163	95
HCQ	207	119	71	32	20
Azith	682	392	192	102	57
Doxy	289	181	101	50	27

Recovery Inferences

	Pr(Superiority)	Pr(Meaningful Effect)	Pr(Best)	Randomization Probability
SOC				0.5
HCQ	0.928	0.2117	0	0
Azith	0.9803	0.125	0.75	0.5
Doxy	0.826	0.054	0	0

Hospitalization Inferences

Pr(Superiority)	Pr(Meaningful Effect)
0.38	0.023
0.607	0.0235
0.4732	0.025

Hazard Ratio Estimates

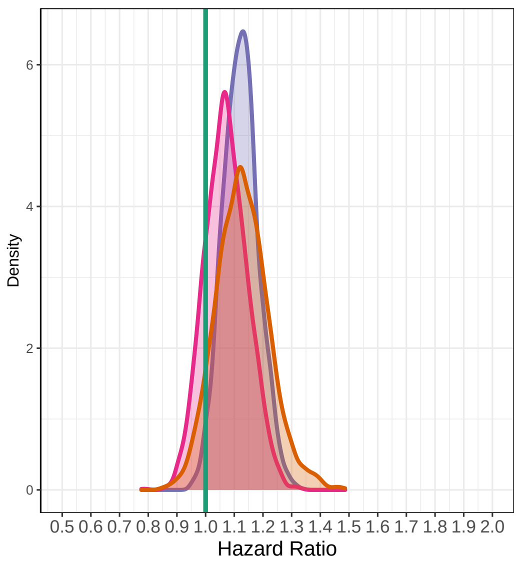

Median Time To Recovery Estimates

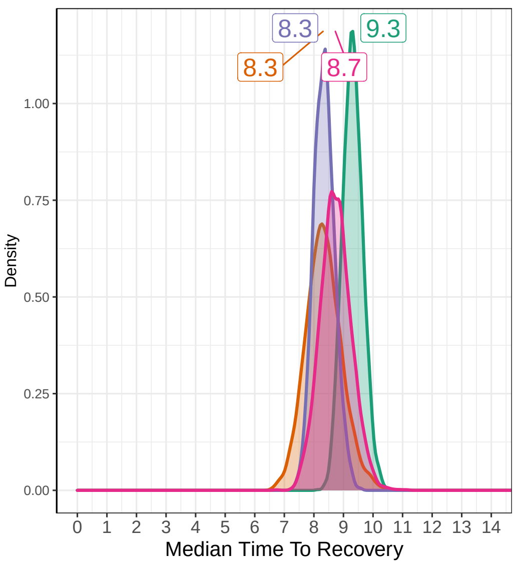

Interim 6 on March 04, 2021

Recovery Data

	Enrolled	Complete	Recovered	Exposure Days	Recoveries Per Day	Estimated Hazard	Estimated HR (95% interval)	Estimated Median Time to Recovery
SOC	1010	955	861	11070	0.078	0.075	1	9.3
HCQ	207	207	188	2127	0.088	0.084	1.113 (0.957, 1.299)	8.38
Azith	782	737	677	7695	0.088	0.084	1.115 (1.019, 1.232)	8.38
Doxy	289	289	263	3158	0.083	0.08	1.062 (0.926, 1.22)	8.78
Total	2288	2188	1989	24050	0.083			

Hospitalization Data

Hospitalizations	28 Day Completers	Observed Hosp. Rate	Est. Hosp. Rate (95% interval)
31	881	0.0352	0.0314 (0.0215, 0.0431)
7	207	0.0338	0.0384 (0.0174, 0.0679)
27	682	0.0396	0.0356 (0.0239, 0.0497)
9	289	0.0311	0.0341 (0.0164, 0.0578)
74	2059	0.0359	

Intervention Status
Enrolling
Dropped
Enrolling
Dropped

Observed Patient Recoveries

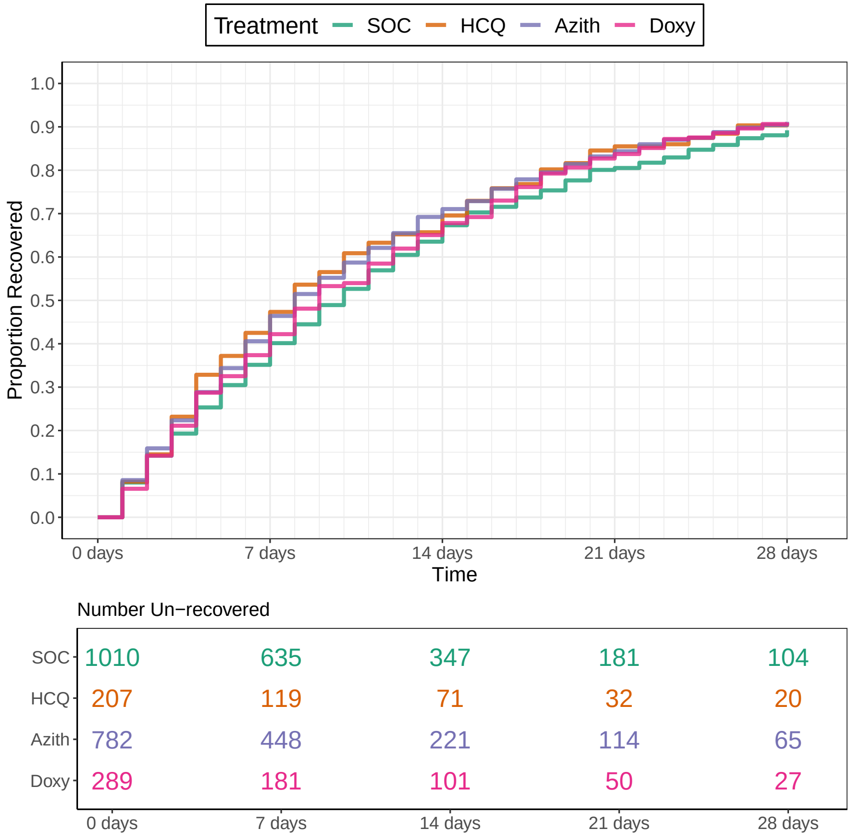

Recovery Inferences

	Pr(Superiority)	Pr(Meaningful Effect)	Pr(Best)	Randomization Probability
SOC				0.5
HCQ	0.9197	0.1733	0	0
Azith	0.9887	0.077	0.75	0.5
Doxy	0.8143	0.0377	0	0

Hospitalization Inferences

Pr(Superiority)	Pr(Meaningful Effect)
0.331	0.0162
0.3137	0.0028
0.4255	0.0168

Hazard Ratio Estimates

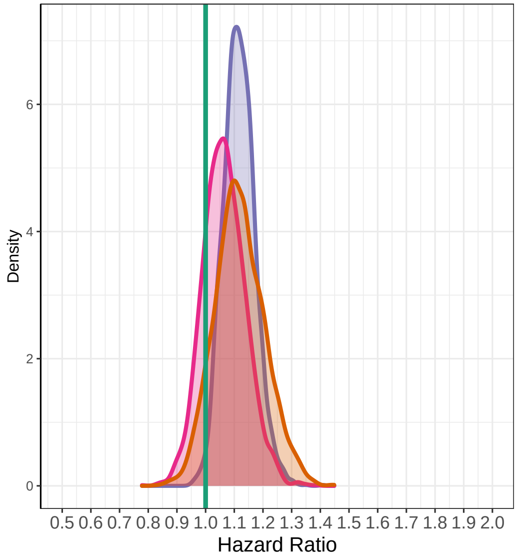

Median Time To Recovery Estimates

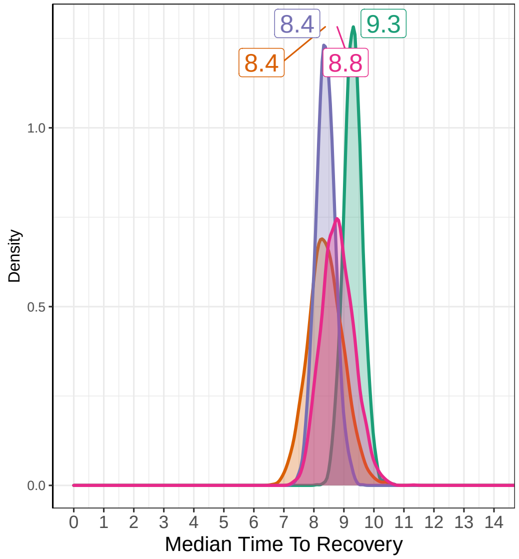

Interim 7 on April 01, 2021

Recovery Data

	Enrolled	Complete	Recovered	Exposure Days	Recoveries Per Day	Estimated Hazard	Estimated HR (95% interval)	Estimated Median Time to Recovery
SOC	1130	1085	975	12506	0.078	0.076	1	9.29
HCQ	207	207	188	2127	0.088	0.085	1.114 (0.96, 1.287)	8.38
Azith	884	848	784	8906	0.088	0.085	1.115 (1.015, 1.217)	8.37
Doxy	289	289	263	3158	0.083	0.081	1.059 (0.924, 1.206)	8.8
Total	2510	2429	2210	26697	0.083			

Hospitalization Data

Hospitalizations	28 Day Completers	Observed Hosp. Rate	Est. Hosp. Rate (95% interval)
33	1010	0.0327	0.03 (0.0209, 0.0405)
7	207	0.0338	0.0382 (0.0164, 0.0674)
31	782	0.0396	0.0361 (0.0249, 0.0494)
9	289	0.0311	0.0342 (0.0163, 0.0577)
80	2288	0.0350	

Intervention Status
Enrolling
Dropped
Enrolling
Dropped

Observed Patient Recoveries

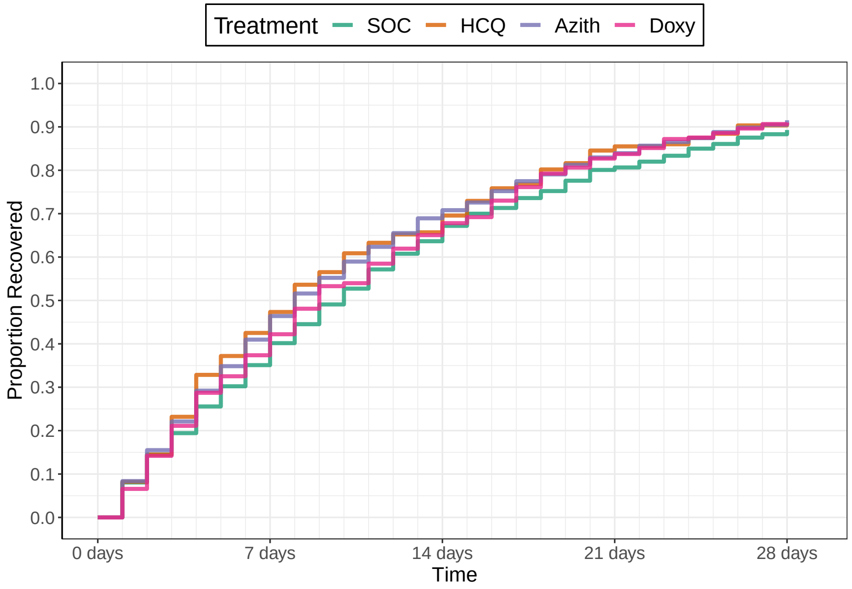

Number Un−recovered

	0 days	7 days	14 days	21 days	28 days
SOC	1130	717	390	209	120
HCQ	207	119	71	32	20
Azith	884	511	265	139	73
Doxy	289	181	101	50	27

Recovery Inferences

	Pr(Superiority)	Pr(Meaningful Effect)	Pr(Best)	Randomization Probability
SOC				0.5
HCQ	0.927	0.1657	0	0
Azith	0.9893	0.0693	0.75	0.5
Doxy	0.803	0.036	0	0

Hospitalization Inferences

Pr(Superiority)	Pr(Meaningful Effect)
0.3045	0.0082
0.2268	8e−04
0.3902	0.0092

Hazard Ratio Estimates

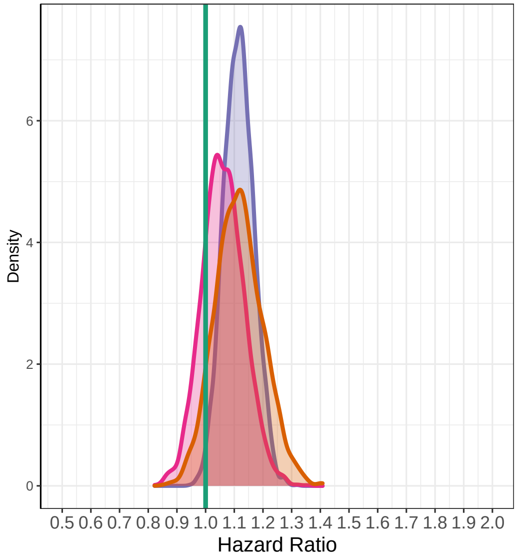

Median Time To Recovery Estimates

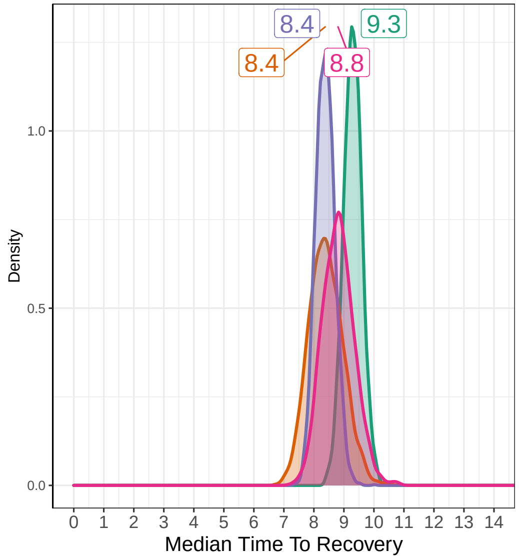

Interim 8 on April 29, 2021

Recovery Data

	Enrolled	Complete	Recovered	Exposure Days	Recoveries Per Day	Estimated Hazard	Estimated HR (95% interval)	Estimated Median Time to Recovery
SOC	1228	1194	1073	13572	0.079	0.076	1	9.12
HCQ	207	207	188	2127	0.088	0.084	1.1 (0.944, 1.283)	8.33
Azith	996	954	876	9989	0.088	0.084	1.097 (1.008, 1.198)	8.35
Doxy	289	289	263	3158	0.083	0.08	1.049 (0.921, 1.187)	8.72
Total	2720	2644	2400	28845	0.083			

Hospitalization Data

Hospitalizations	28 Day Completers	Observed Hosp. Rate	Est. Hosp. Rate (95% interval)
34	1130	0.0301	0.0285 (0.0199, 0.0385)
7	207	0.0338	0.0382 (0.0166, 0.0675)
35	884	0.0396	0.0361 (0.0254, 0.0481)
9	289	0.0311	0.0344 (0.0167, 0.0579)
85	2510	0.0339	

Intervention Status
Enrolling
Dropped
Futility
Dropped

Observed Patient Recoveries

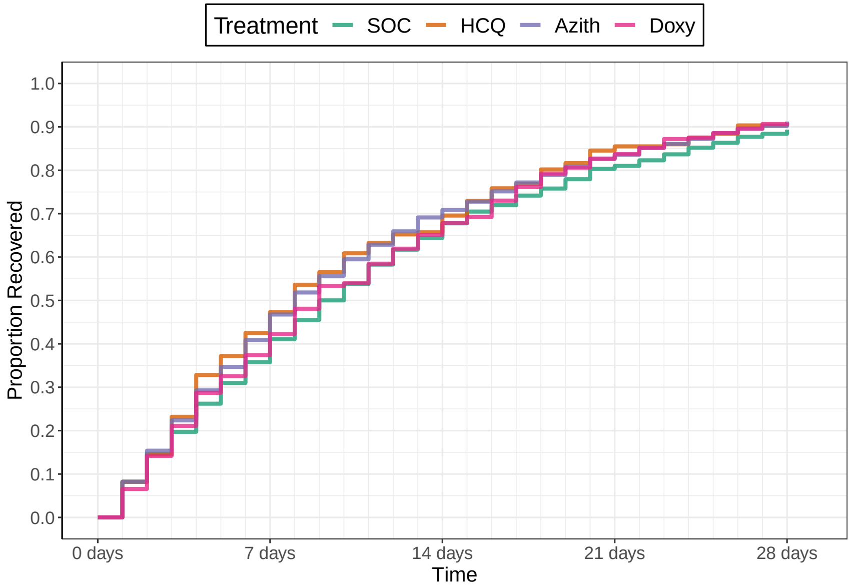

Number Un−recovered

	0 days	7 days	14 days	21 days	28 days
SOC	1228	777	421	230	133
HCQ	207	119	71	32	20
Azith	996	573	288	159	87
Doxy	289	181	101	50	27

Recovery Inferences

	Pr(Superiority)	Pr(Meaningful Effect)	Pr(Best)	Randomization Probability
SOC				1
HCQ	0.8877	0.1253	0	0
Azith	0.9853	0.027	0	0
Doxy	0.759	0.019	0	0

Hospitalization Inferences

Pr(Superiority)	Pr(Meaningful Effect)
0.254	0.0068
0.1568	0
0.3295	0.006

Hazard Ratio Estimates

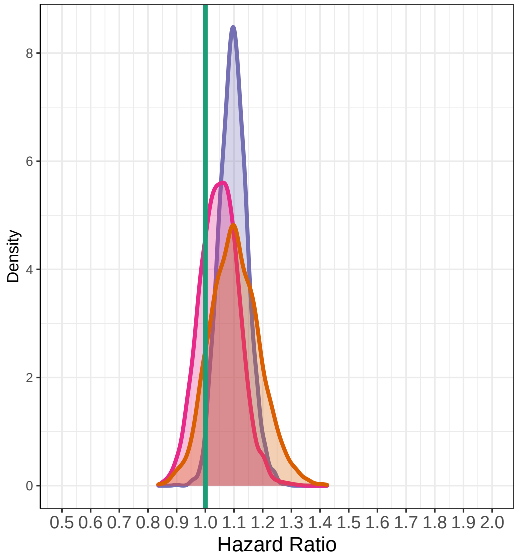

Median Time To Recovery Estimates

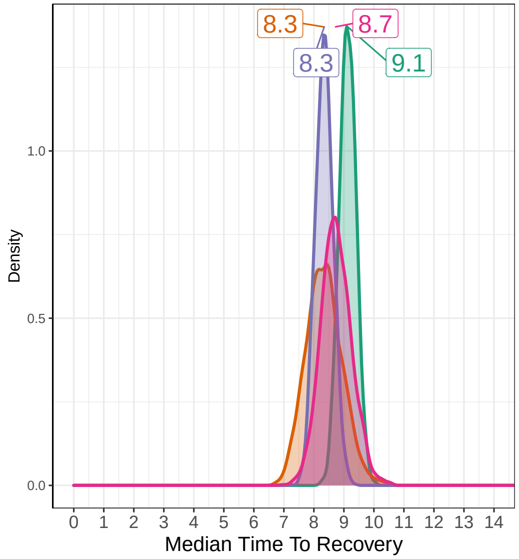

Interim 9 on August 26, 2022

Recovery Data

	Enrolled	Complete	Recovered	Exposure Days	Recoveries Per Day	Estimated Hazard	Estimated HR (95% interval)	Estimated Median Time to Recovery
SOC	4508	4508	3979	52829	0.075	0.073	1	9.64
HCQ	207	207	188	2127	0.088	0.085	1.154 (0.995, 1.322)	8.39
Azith	996	996	908	10382	0.087	0.084	1.153 (1.071, 1.235)	8.4
Doxy	289	289	263	3158	0.083	0.081	1.098 (0.97, 1.243)	8.79
Total	6000	6000	5338	68497	0.078			

Hospitalization Data

Hospitalizations	28 Day Completers	Observed Hosp. Rate	Est. Hosp. Rate (95% interval)
138	4508	0.0306	0.0308 (0.026, 0.0361)
7	207	0.0338	0.0387 (0.017, 0.0686)
35	996	0.0351	0.0361 (0.0253, 0.0486)
9	289	0.0311	0.0344 (0.0167, 0.0584)
189	6000	0.0315	

Intervention Status
Complete
Dropped
Dropped
Dropped

Observed Patient Recoveries

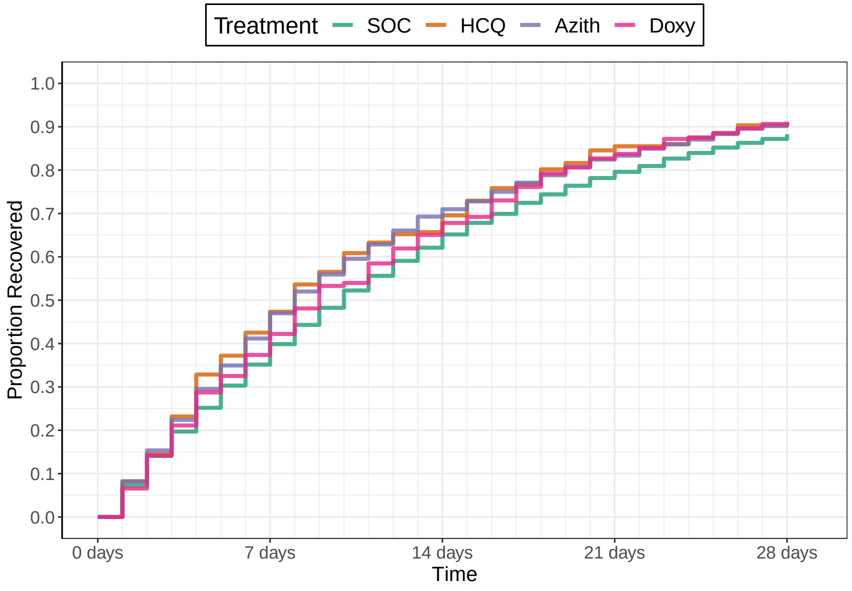

Number Un−recovered

	0 days	7 days	14 days	21 days	28 days
SOC	4508	2924	1708	984	577
HCQ	207	119	71	32	20
Azith	996	586	306	175	98
Doxy	289	181	101	50	27

Recovery Inferences

	Pr(Superiority)	Pr(Meaningful Effect)	Pr(Best)	Randomization Probability
SOC				
HCQ	0.9697	0.3407	0	
Azith	1	0.1867	0	
Doxy	0.928	0.1067	0	

Hospitalization Inferences

Pr(Superiority)	Pr(Meaningful Effect)
0.3088	0.005
0.2218	0
0.4058	0.0032

Hazard Ratio Estimates

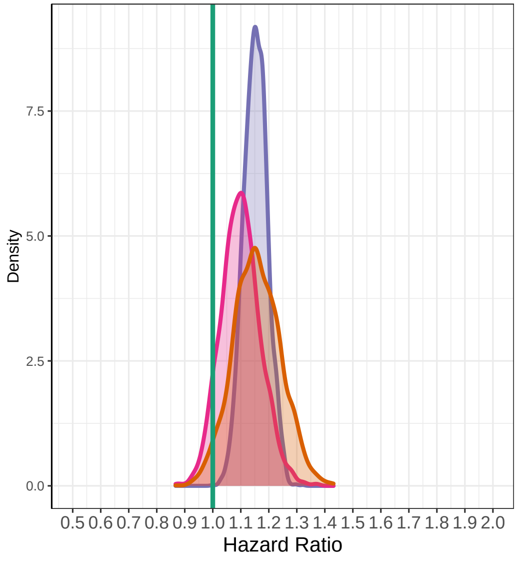

Median Time To Recovery Estimates

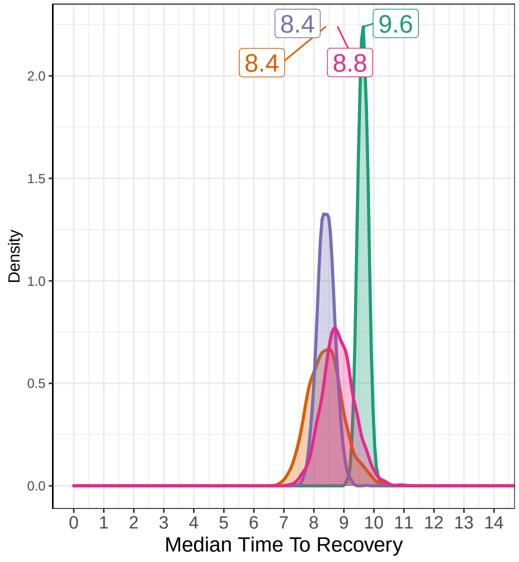

6.5 Example 4

April 1, 2020

Before Patient Enrollment Begins

Recovery Data

	Enrolled	Complete	Recovered	Exposure Days	Recoveries Per Day	Estimated Hazard	Estimated HR (95% interval)	Estimated Median Survival Time
SOC	0	0	0	0				
HCQ	0	0	0	0				
Azith	0	0	0	0				
Doxy	0	0	0	0				
Total	0	0	0	0				

Hospitalization Data

Hospitalizations	28 Day Completers	Observed Hosp. Rate	Est. Hosp. Rate (95% interval)
0	0		
0	0		
0	0		
0	0		
0	0		

Intervention Status
Enrolling
Enrolling
Unopened
Unopened

Observed Patient Recoveries

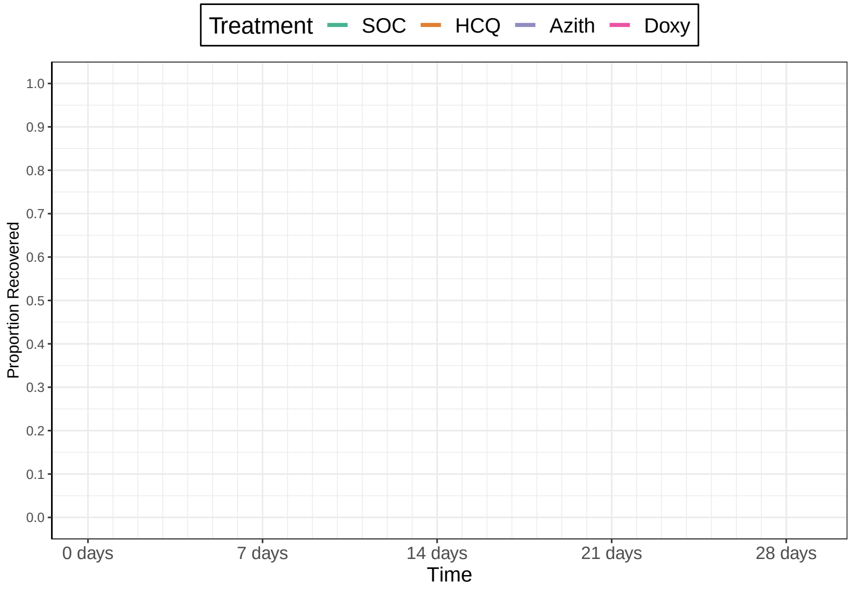

Number Un−recovered

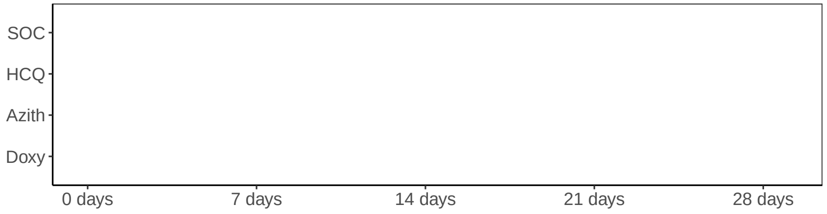

Recovery Inferences

	Pr(Superiority)	Pr(Meaningful Effect)	Pr(Best)	Randomization Probability
SOC				0.5
HCQ				0.5
Azith				0
Doxy				0

Hospitalization Inferences

Pr(Superiority)	Pr(Meaningful Effect)
0	0
0	0
0	0
0	0

Hazard Ratio Estimates

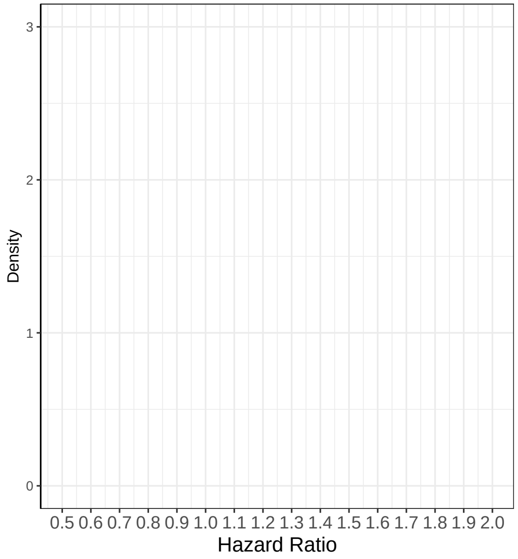

Median Time To Recovery Estimates

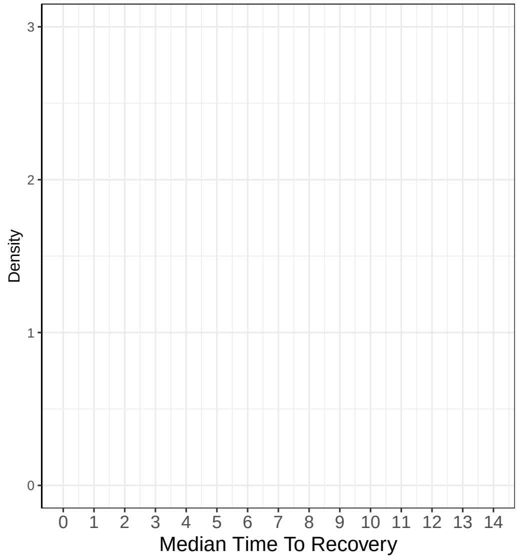

Remove Drug on May 22, 2020

Recovery Data

	Enrolled	Complete	Recovered	Exposure Days	Recoveries Per Day	Estimated Hazard	Estimated HR (95% interval)	Estimated Median Time to Recovery
SOC	206	152	146	1728	0.084			
HCQ	207	143	134	1821	0.074			
Azith	0	0	0	0				
Doxy	0	0	0	0				
Total	413	295	280	3549	0.079			

Hospitalization Data

Hospitalizations	28 Day Completers	Observed Hosp. Rate	Est. Hosp. Rate (95% interval)
4	79	0.0506	
5	78	0.0641	
0	0	NA	
0	0	NA	
9	157	0.0573	

Intervention Status
Enrolling
Removing
Unopened
Unopened

Observed Patient Recoveries

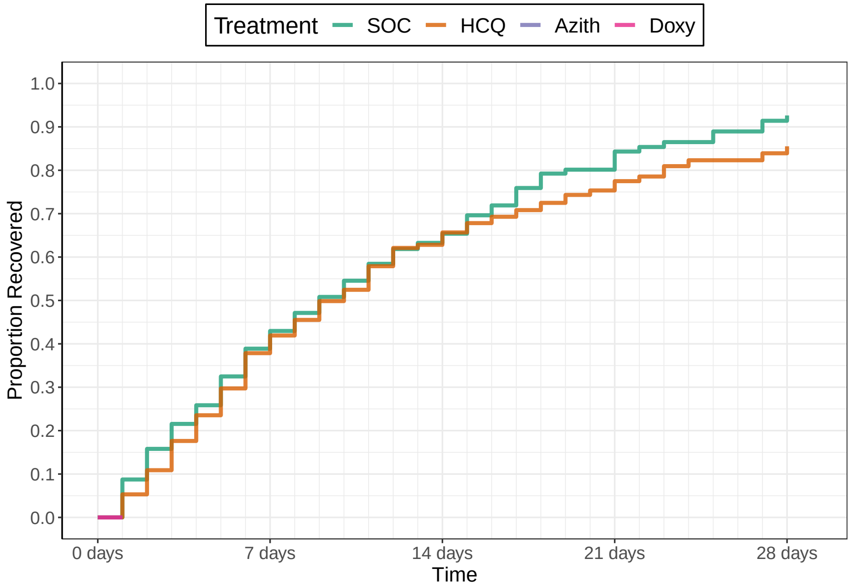

Number Un−recovered

	0 days	7 days	14 days	21 days	28 days
SOC	206	105	52	19	7
HCQ	207	107	52	23	10
Azith					
Doxy					

Recovery Inferences

	Pr(Superiority)	Pr(Meaningful Effect)	Pr(Best)	Randomization Probability
SOC				1
HCQ			0	0
Azith			0	0
Doxy			0	0

Hospitalization Inferences

Pr(Superiority)	Pr(Meaningful Effect)

Hazard Ratio Estimates

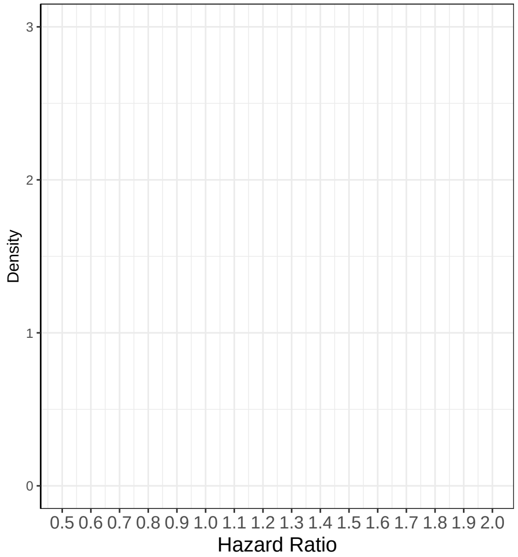

Median Time To Recovery Estimates

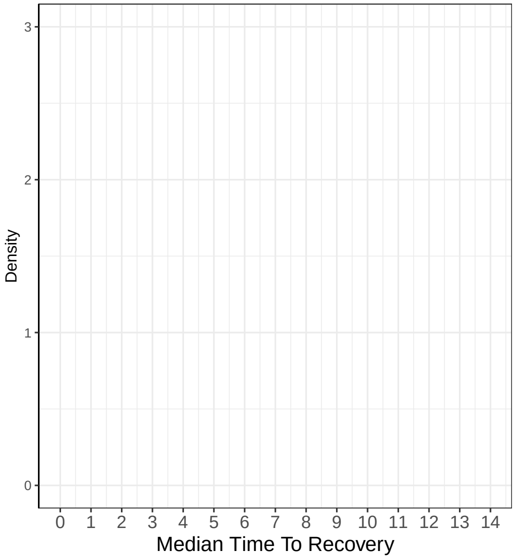

Add Drug on May 23, 2020

Recovery Data

	Enrolled	Complete	Recovered	Exposure Days	Recoveries Per Day	Estimated Hazard	Estimated HR (95% interval)	Estimated Median Time to Recovery
SOC	208	155	149	1782	0.084			
HCQ	207	147	138	1884	0.073			
Azith	0	0	0	0				
Doxy	0	0	0	0				
Total	415	302	287	3666	0.078			

Hospitalization Data

Hospitalizations	28 Day Completers	Observed Hosp. Rate	Est. Hosp. Rate (95% interval)
4	80	0.0500	
5	80	0.0625	
0	0	NA	
0	0	NA	
9	160	0.0562	

Intervention Status
Enrolling
Paused
Introducing
Unopened

Observed Patient Recoveries

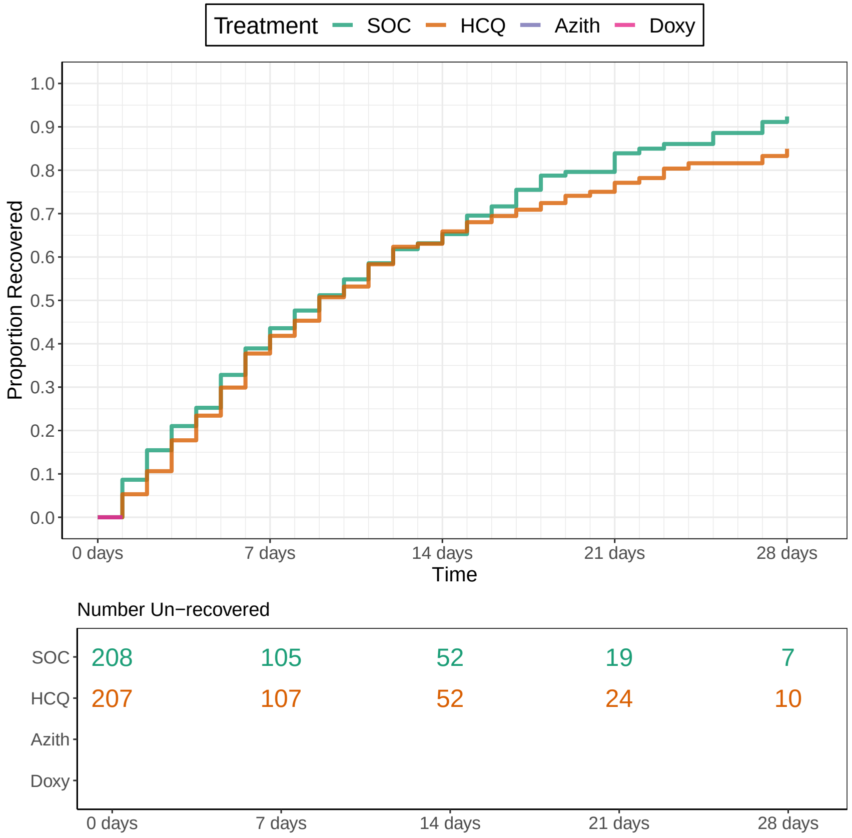

Recovery Inferences

	Pr(Superiority)	Pr(Meaningful Effect)	Pr(Best)	Randomization Probability
SOC				0.5
HCQ			0	0
Azith			1	0.5
Doxy			0	0

Hospitalization Inferences

Pr(Superiority)	Pr(Meaningful Effect)

Hazard Ratio Estimates

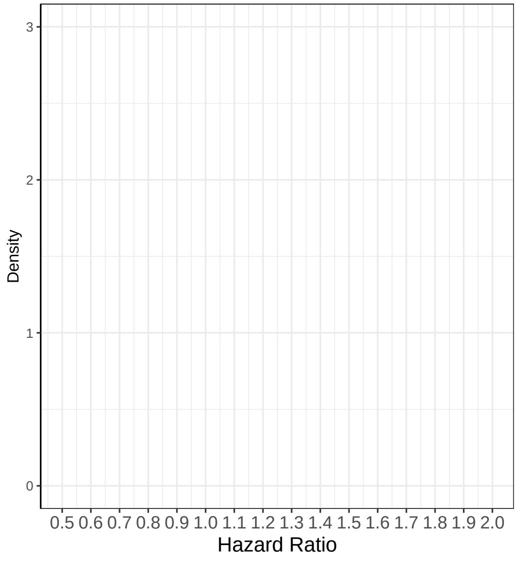

Median Time To Recovery Estimates

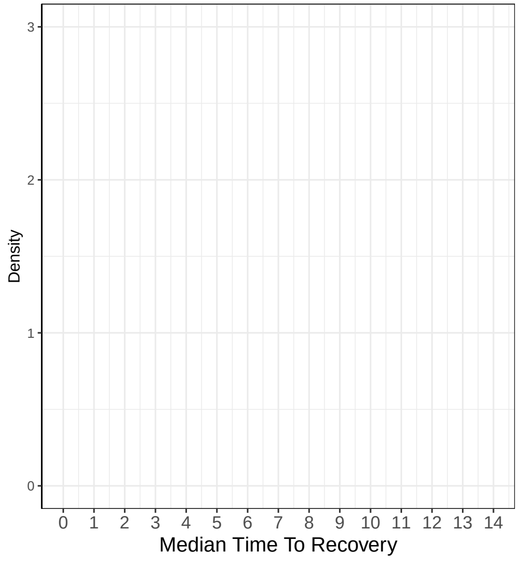

Add Drug on July 23, 2020

Recovery Data

	Enrolled	Complete	Recovered	Exposure Days	Recoveries Per Day	Estimated Hazard	Estimated HR (95% interval)	Estimated Median Time to Recovery
SOC	326	310	286	3499	0.082			
HCQ	207	207	179	2453	0.073			
Azith	118	104	100	1078	0.093			
Doxy	0	0	0	0				
Total	651	621	565	7030	0.08			

Hospitalization Data

Hospitalizations	28 Day Completers	Observed Hosp. Rate	Est. Hosp. Rate (95% interval)
7	285	0.0246	
5	207	0.0242	
4	77	0.0519	
0	0	NA	
16	569	0.0281	

Intervention Status
Enrolling
Paused
Introducing
Introducing

Observed Patient Recoveries

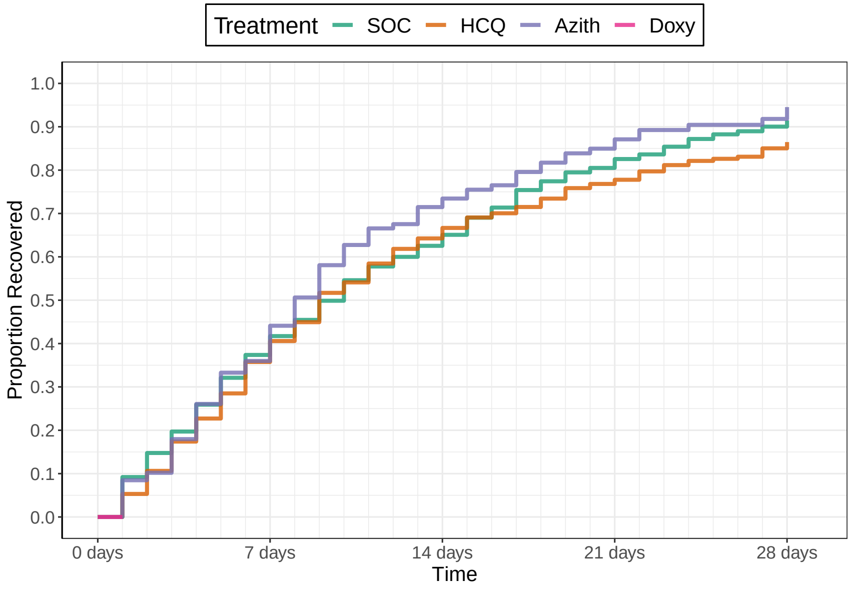

Number Un−recovered

	0 days	7 days	14 days	21 days	28 days
SOC	326	202	118	57	28
HCQ	207	133	74	48	31
Azith	118	71	29	14	6
Doxy					

Recovery Inferences

	Pr(Superiority)	Pr(Meaningful Effect)	Pr(Best)	Randomization Probability
SOC				0.33
HCQ			0	0
Azith			1	0.33
Doxy			0	0.33

Hospitalization Inferences

Pr(Superiority)	Pr(Meaningful Effect)

Hazard Ratio Estimates

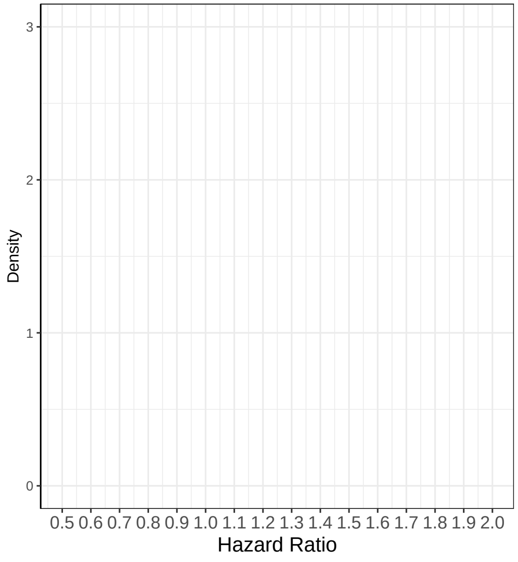

Median Time To Recovery Estimates

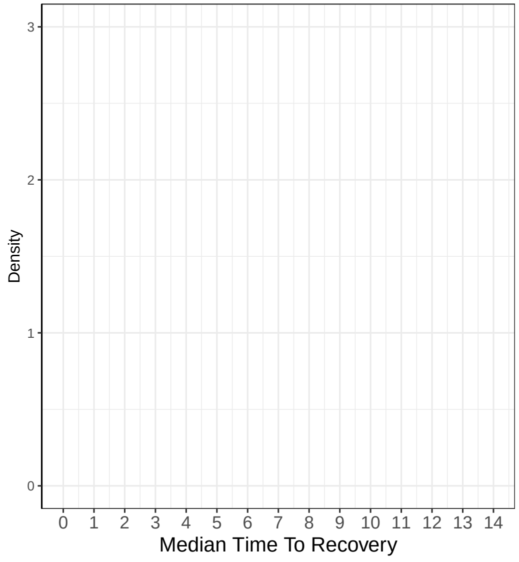

Interim 1 on October 15, 2020

Recovery Data

	Enrolled	Complete	Recovered	Exposure Days	Recoveries Per Day	Estimated Hazard	Estimated HR (95% interval)	Estimated Median Time to Recovery
SOC	482	440	405	4826	0.084	0.082	1	8.69
HCQ	207	207	179	2453	0.073	0.072	0.875 (0.742, 1.03)	9.88
Azith	312	270	260	2615	0.099	0.095	1.158 (1.004, 1.349)	7.53
Doxy	209	151	146	1571	0.093	0.088	1.08 (0.901, 1.302)	8.05
Total	1210	1068	990	11465	0.086			

Hospitalization Data

Hospitalizations	28 Day Completers	Observed Hosp. Rate	Est. Hosp. Rate (95% interval)
10	387	0.0258	0.0228 (0.0115, 0.0382)
5	207	0.0242	0.0286 (0.0108, 0.0541)
8	200	0.0400	0.0288 (0.0132, 0.05)
2	79	0.0253	0.0141 (0.003, 0.0346)
25	873	0.0286	

Intervention Status
Enrolling
Futility
Enrolling
Enrolling

Observed Patient Recoveries

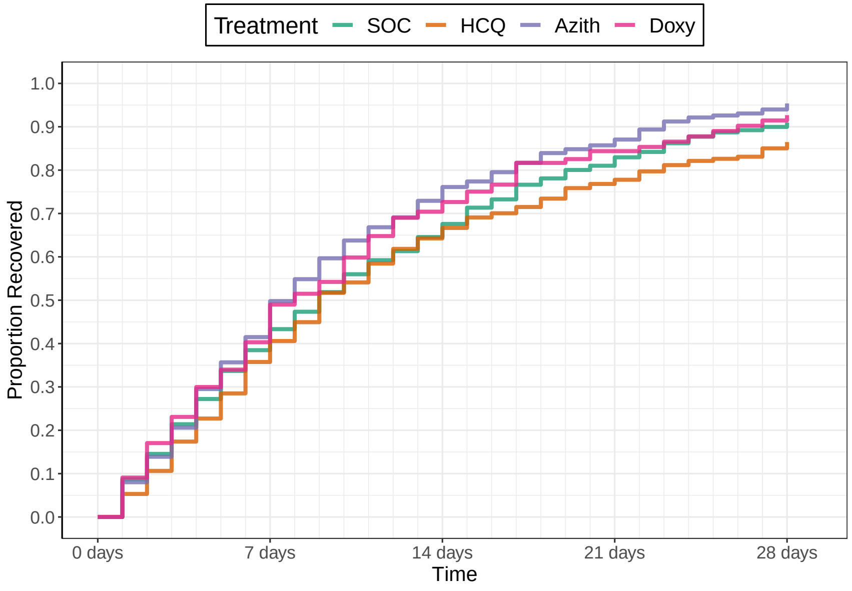

Number Un−recovered

	0 days	7 days	14 days	21 days	28 days
SOC	482	279	152	78	40
HCQ	207	133	74	48	31
Azith	312	162	68	32	13
Doxy	209	103	40	16	7

Recovery Inferences

	Pr(Superiority)	Pr(Meaningful Effect)	Pr(Best)	Randomization Probability
SOC				0.33
HCQ	0.0513	0	0	0
Azith	0.9793	0.28	0.567	0.37
Doxy	0.8027	0.116	0.183	0.3

Hospitalization Inferences

Pr(Superiority)	Pr(Meaningful Effect)
0.3352	0.0142
0.3022	0.0122
0.806	0.1338

Hazard Ratio Estimates

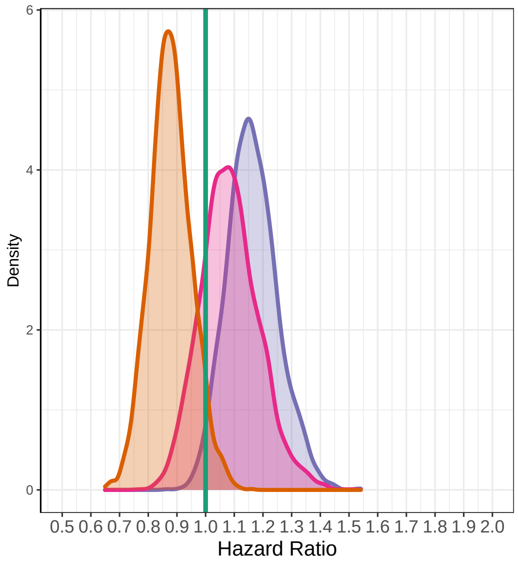

Median Time To Recovery Estimates

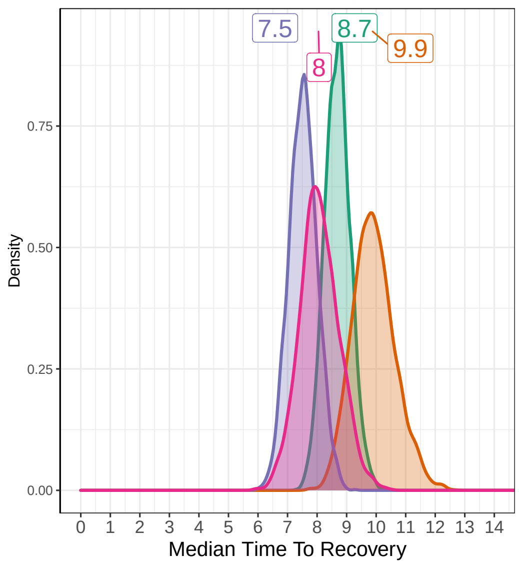

Interim 2 on November 12, 2020

Recovery Data

	Enrolled	Complete	Recovered	Exposure Days	Recoveries Per Day	Estimated Hazard	Estimated HR (95% interval)	Estimated Median Time to Recovery
SOC	573	535	488	5941	0.082	0.079	1	8.83
HCQ	207	207	179	2453	0.073	0.071	0.891 (0.756, 1.049)	9.85
Azith	405	373	354	3728	0.095	0.09	1.132 (0.989, 1.296)	7.85
Doxy	263	246	231	2421	0.095	0.09	1.134 (0.977, 1.32)	7.83
Total	1448	1361	1252	14543	0.086			

Hospitalization Data

Hospitalizations	28 Day Completers	Observed Hosp. Rate	Est. Hosp. Rate (95% interval)
15	482	0.0311	0.0279 (0.0162, 0.0424)
5	207	0.0242	0.0285 (0.0109, 0.054)
10	312	0.0321	0.0274 (0.014, 0.0455)
4	209	0.0191	0.019 (0.0062, 0.0387)
34	1210	0.0281	

Intervention Status
Enrolling
Dropped
Enrolling
Enrolling

Observed Patient Recoveries

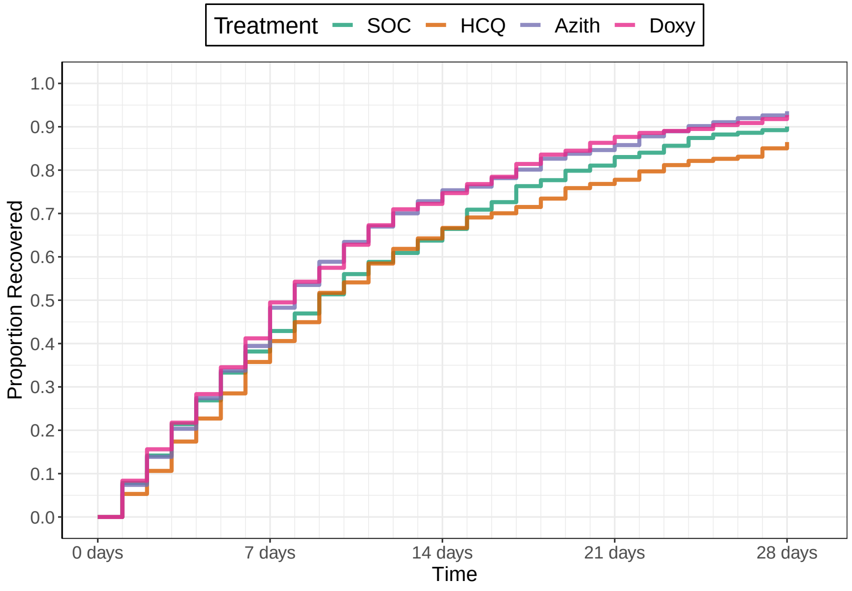

Number Un−recovered

	0 days	7 days	14 days	21 days	28 days
SOC	573	340	188	96	52
HCQ	207	133	74	48	31
Azith	405	233	97	54	23
Doxy	263	149	67	30	18

Recovery Inferences

	Pr(Superiority)	Pr(Meaningful Effect)	Pr(Best)	Randomization Probability
SOC				0.33
HCQ	0.0867	0	0	0
Azith	0.966	0.1797	0.3665	0.34
Doxy	0.944	0.1967	0.3835	0.33

Hospitalization Inferences

Pr(Superiority)	Pr(Meaningful Effect)
0.5152	0.042
0.5262	0.033
0.8028	0.1468

Hazard Ratio Estimates

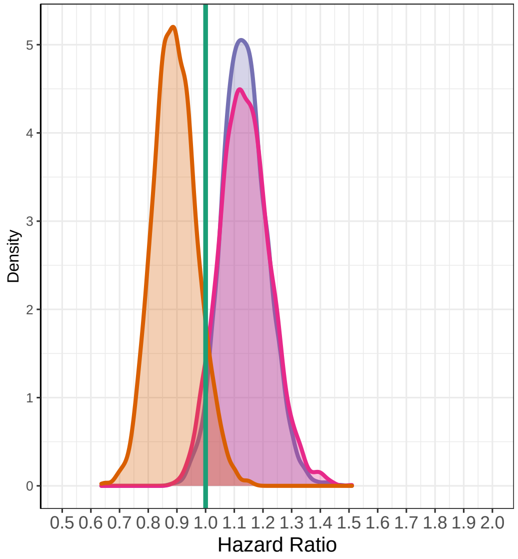

Median Time To Recovery Estimates

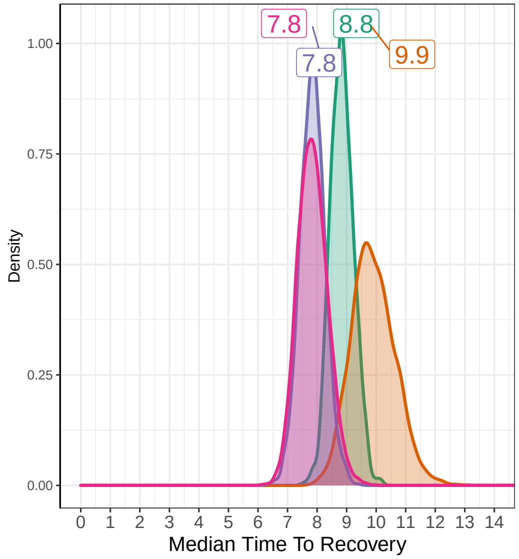

Interim 3 on December 10, 2020

Recovery Data

	Enrolled	Complete	Recovered	Exposure Days	Recoveries Per Day	Estimated Hazard	Estimated HR (95% interval)	Estimated Median Time to Recovery
SOC	638	615	559	6714	0.083	0.08	1	8.77
HCQ	207	207	179	2453	0.073	0.071	0.884 (0.749, 1.036)	9.85
Azith	470	452	422	4524	0.093	0.089	1.107 (0.983, 1.254)	7.99
Doxy	334	307	290	3140	0.092	0.088	1.093 (0.954, 1.259)	8.07
Total	1649	1581	1450	16831	0.086			

Hospitalization Data

Hospitalizations	28 Day Completers	Observed Hosp. Rate	Est. Hosp. Rate (95% interval)
18	573	0.0314	0.0295 (0.0181, 0.044)
5	207	0.0242	0.0288 (0.0108, 0.0568)
11	405	0.0272	0.0252 (0.0134, 0.0417)
7	263	0.0266	0.0241 (0.0103, 0.0436)
41	1448	0.0283	

Intervention Status
Enrolling
Dropped
Enrolling
Enrolling

Observed Patient Recoveries

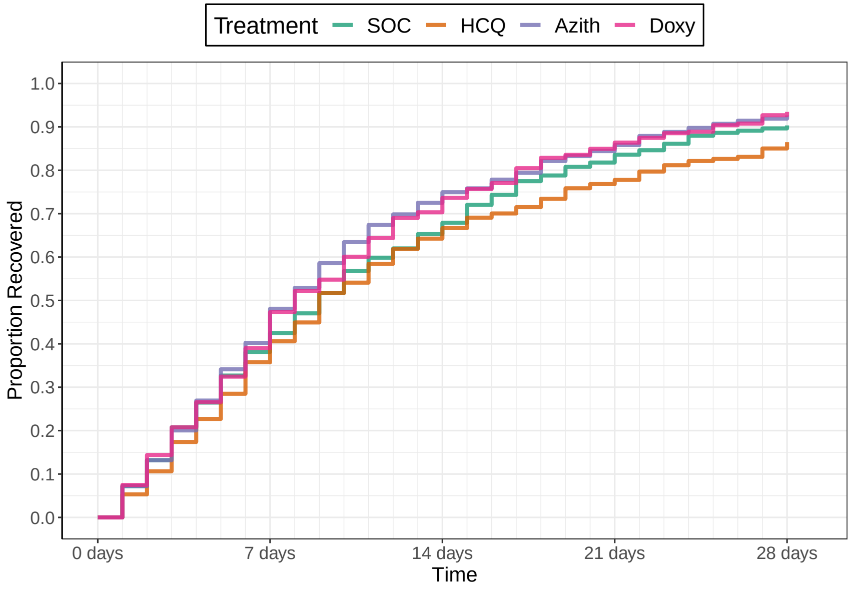

Number Un−recovered

	0 days	7 days	14 days	21 days	28 days
SOC	638	384	211	110	60
HCQ	207	133	74	48	31
Azith	470	274	124	67	33
Doxy	334	191	89	42	19

Recovery Inferences

	Pr(Superiority)	Pr(Meaningful Effect)	Pr(Best)	Randomization Probability
SOC				0.33
HCQ	0.064	0	0	0
Azith	0.9527	0.0717	0.4225	0.34
Doxy	0.9047	0.0707	0.3275	0.32

Hospitalization Inferences

Pr(Superiority)	Pr(Meaningful Effect)
0.5562	0.06
0.6805	0.0505
0.7072	0.0848

Hazard Ratio Estimates

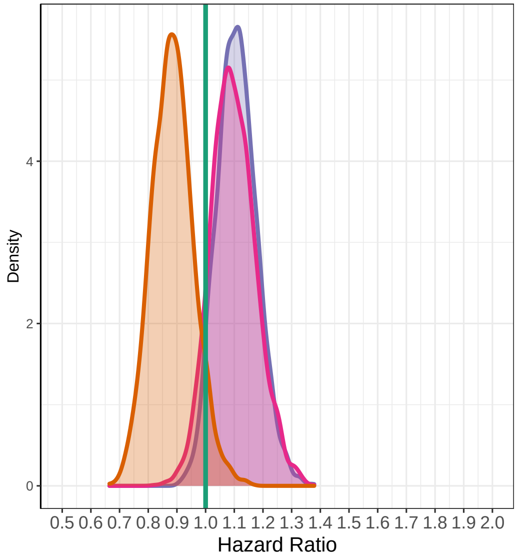

Median Time To Recovery Estimates

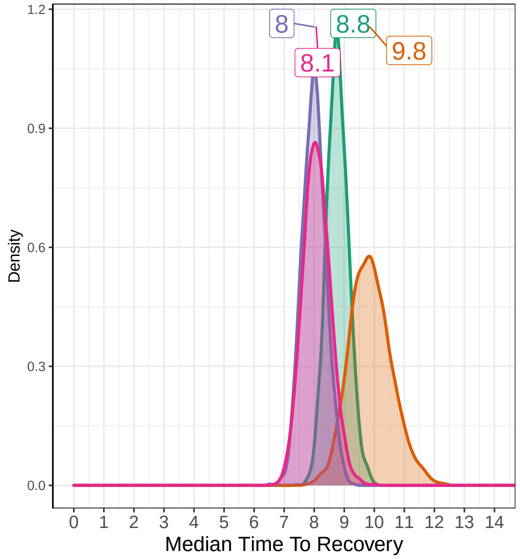

Interim 4 on January 07, 2021

Recovery Data

	Enrolled	Complete	Recovered	Exposure Days	Recoveries Per Day	Estimated Hazard	Estimated HR (95% interval)	Estimated Median Time to Recovery
SOC	709	684	620	7506	0.083	0.08	1	8.89
HCQ	207	207	179	2453	0.073	0.071	0.886 (0.759, 1.029)	9.92
Azith	553	516	484	5173	0.094	0.09	1.118 (1, 1.254)	8.04
Doxy	388	367	347	3635	0.095	0.091	1.138 (1.003, 1.285)	7.92
Total	1857	1774	1630	18768	0.087			

Hospitalization Data

Hospitalizations	28 Day Completers	Observed Hosp. Rate	Est. Hosp. Rate (95% interval)
18	638	0.0282	0.0267 (0.0163, 0.0397)
5	207	0.0242	0.0289 (0.0106, 0.0564)
12	470	0.0255	0.0235 (0.0125, 0.0373)
10	334	0.0299	0.0283 (0.0143, 0.0466)
45	1649	0.0273	

Intervention Status
Enrolling
Dropped
Enrolling
Enrolling

Observed Patient Recoveries

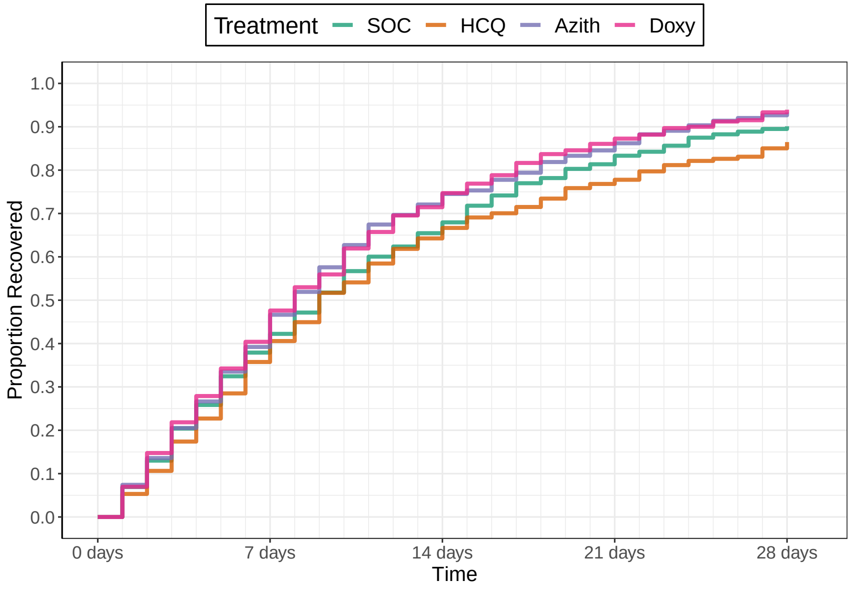

Number Un−recovered

	0 days	7 days	14 days	21 days	28 days
SOC	709	431	235	123	68
HCQ	207	133	74	48	31
Azith	553	319	138	75	35
Doxy	388	223	105	46	22

Recovery Inferences

	Pr(Superiority)	Pr(Meaningful Effect)	Pr(Best)	Randomization Probability
SOC				0.33
HCQ	0.0633	0	0	0
Azith	0.9743	0.0653	0.2918	0.33
Doxy	0.978	0.128	0.4582	0.33

Hospitalization Inferences

Pr(Superiority)	Pr(Meaningful Effect)
0.4572	0.0332
0.643	0.0288
0.461	0.0102

Hazard Ratio Estimates

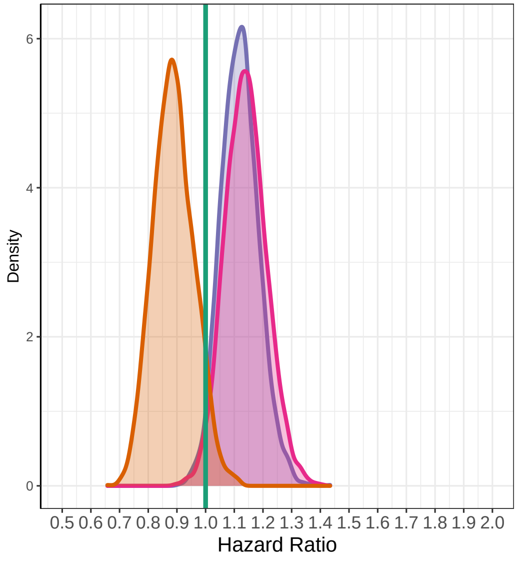

Median Time To Recovery Estimates

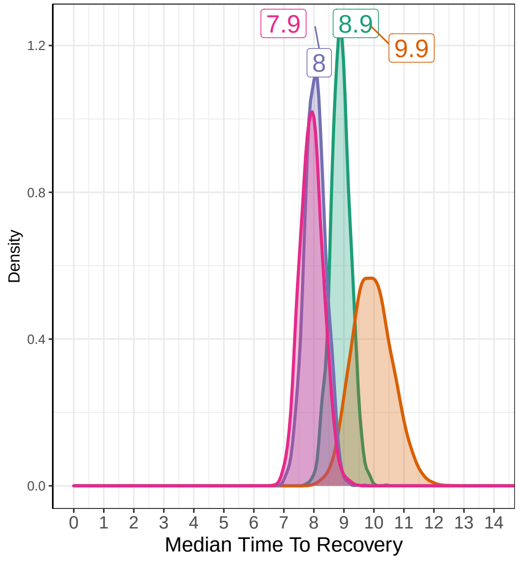

Interim 5 on February 04, 2021

Recovery Data

	Enrolled	Complete	Recovered	Exposure Days	Recoveries Per Day	Estimated Hazard	Estimated HR (95% interval)	Estimated Median Time to Recovery
SOC	770	742	672	8161	0.082	0.081	1	8.97
HCQ	207	207	179	2453	0.073	0.072	0.892 (0.762, 1.037)	9.94
Azith	631	597	561	6070	0.092	0.09	1.113 (0.996, 1.241)	8.14
Doxy	445	426	403	4196	0.096	0.093	1.154 (1.018, 1.302)	7.89
Total	2053	1972	1815	20881	0.087			

Hospitalization Data

Hospitalizations	28 Day Completers	Observed Hosp. Rate	Est. Hosp. Rate (95% interval)
18	709	0.0254	0.0245 (0.0146, 0.0368)
5	207	0.0242	0.0289 (0.0106, 0.0552)
14	553	0.0253	0.0238 (0.0135, 0.0369)
11	388	0.0284	0.0269 (0.0138, 0.0433)
48	1857	0.0258	

Intervention Status
Enrolling
Dropped
Enrolling
Enrolling

Observed Patient Recoveries

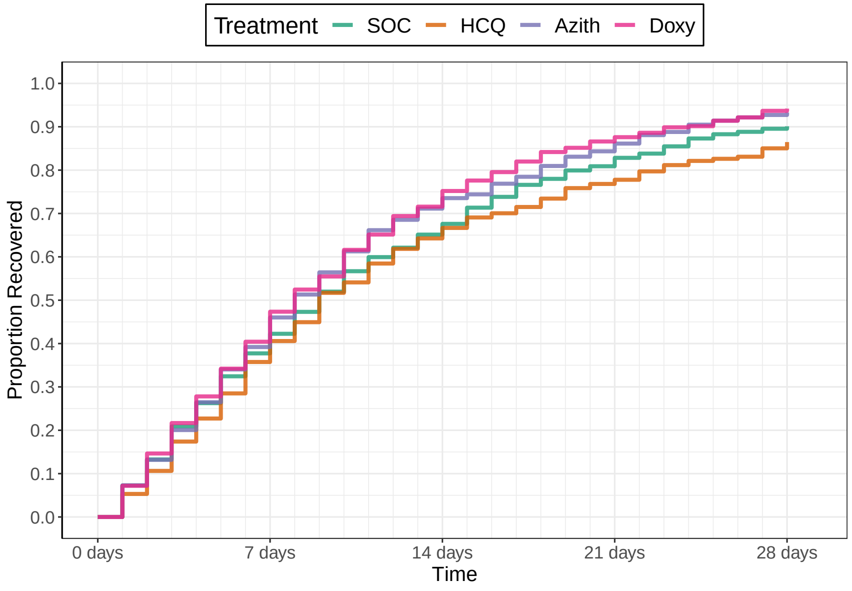

Number Un−recovered

	0 days	7 days	14 days	21 days	28 days
SOC	770	469	253	138	74
HCQ	207	133	74	48	31
Azith	631	374	167	88	39
Doxy	445	258	118	54	25

Recovery Inferences

	Pr(Superiority)	Pr(Meaningful Effect)	Pr(Best)	Randomization Probability
SOC				0.33
HCQ	0.069	0	0	0
Azith	0.9693	0.0623	0.2185	0.33
Doxy	0.9857	0.1877	0.5315	0.34

Hospitalization Inferences

Pr(Superiority)	Pr(Meaningful Effect)
0.3985	0.0142
0.5432	0.0082
0.4128	0.0068

Hazard Ratio Estimates

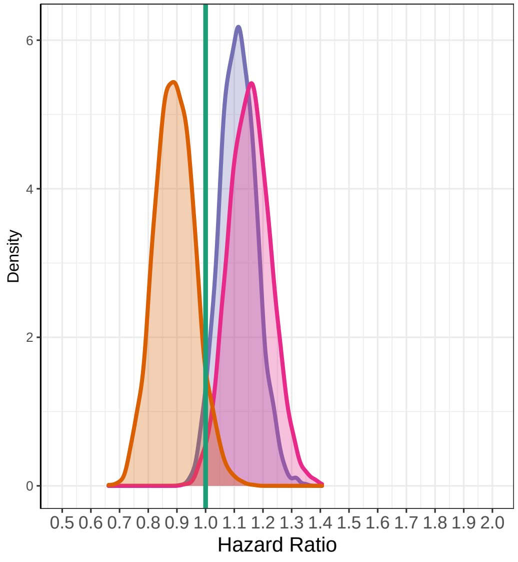

Median Time To Recovery Estimates

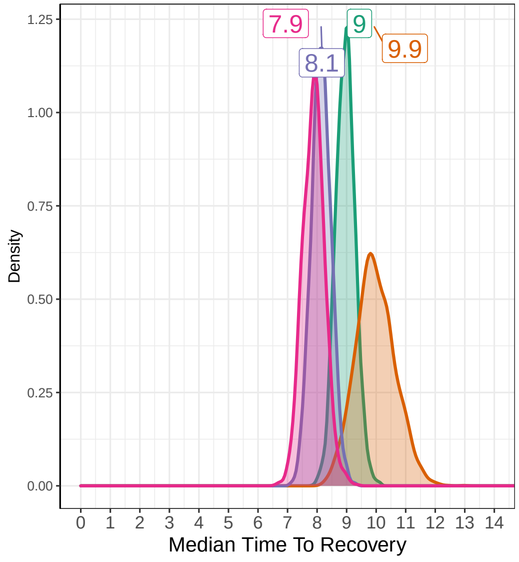

Interim 6 on March 04, 2021

Recovery Data

	Enrolled	Complete	Recovered	Exposure Days	Recoveries Per Day	Estimated Hazard	Estimated HR (95% interval)	Estimated Median Time to Recovery
SOC	847	807	733	8965	0.082	0.08	1	9.02
HCQ	207	207	179	2453	0.073	0.072	0.895 (0.764, 1.043)	9.97
Azith	695	673	629	6919	0.091	0.089	1.102 (0.995, 1.216)	8.26
Doxy	506	482	458	4790	0.096	0.093	1.156 (1.037, 1.296)	7.91
Total	2255	2169	1999	23127	0.086			

Hospitalization Data

Hospitalizations	28 Day Completers	Observed Hosp. Rate	Est. Hosp. Rate (95% interval)
19	770	0.0247	0.0236 (0.0145, 0.0346)
5	207	0.0242	0.0287 (0.0106, 0.0557)
16	631	0.0254	0.0245 (0.0145, 0.0368)
14	445	0.0315	0.0296 (0.0168, 0.0463)
54	2053	0.0263	

Intervention Status
LostOnTTR
Dropped
Futility
SuccessOnTTRFutilityOnHosp Not Announced

Observed Patient Recoveries

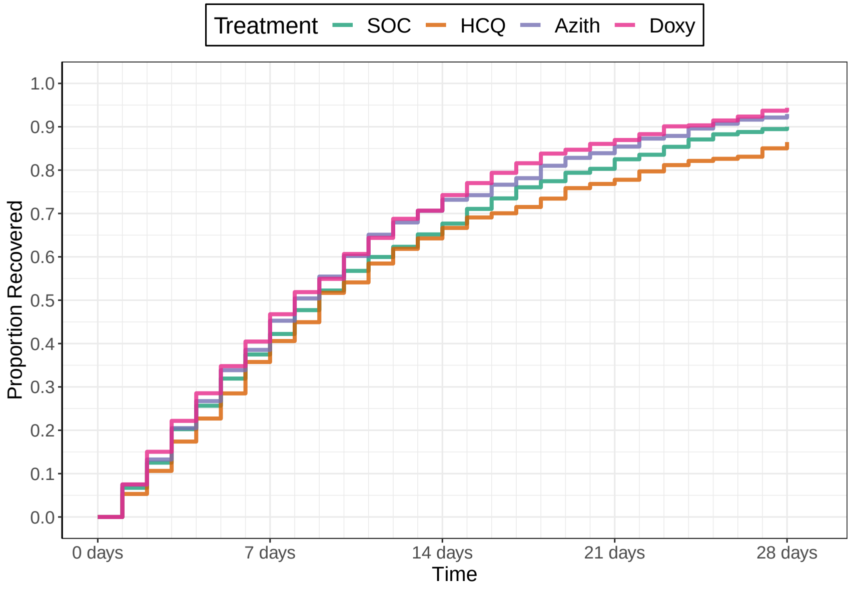

Number Un−recovered

	0 days	7 days	14 days	21 days	28 days
SOC	847	516	279	152	78
HCQ	207	133	74	48	31
Azith	695	420	196	105	49
Doxy	506	293	139	62	28

Recovery Inferences

	Pr(Superiority)	Pr(Meaningful Effect)	Pr(Best)	Randomization Probability
SOC				0.5
HCQ	0.0757	0	0	0
Azith	0.9667	0.0317	0	0
Doxy	0.9933	0.1847	0.75	0.5

Hospitalization Inferences

Pr(Superiority)	Pr(Meaningful Effect)
0.369	0.0078
0.464	0.0028
0.2502	0.0022

Hazard Ratio Estimates

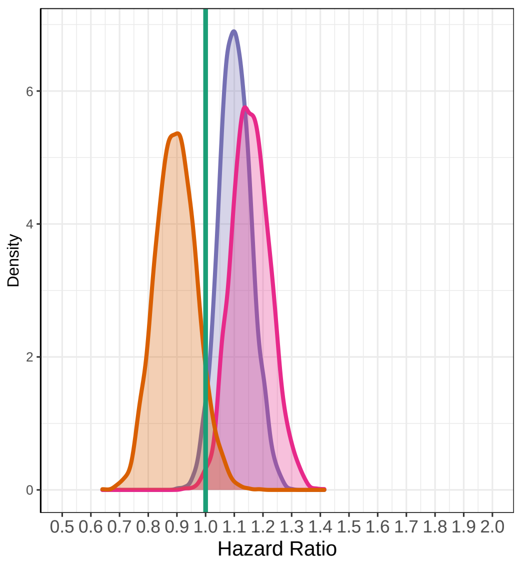

Median Time To Recovery Estimates

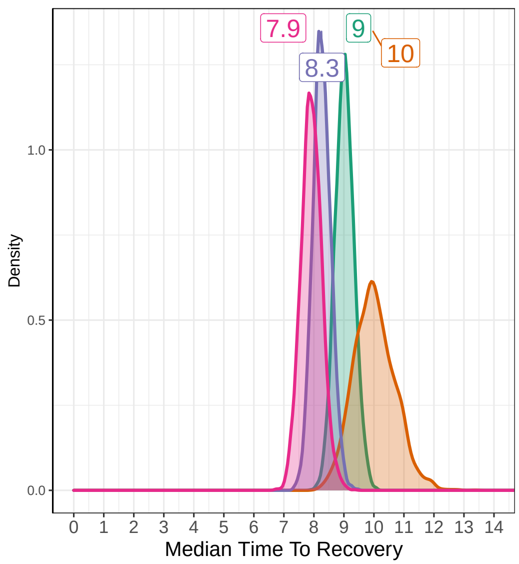

Interim 7 on August 27, 2022

Recovery Data

	Enrolled	Complete	Recovered	Exposure Days	Recoveries Per Day	Estimated Hazard	Estimated HR (95% interval)	Estimated Median Time to Recovery
SOC	2688	2688	2373	30880	0.077	0.074	1	9.39
HCQ	207	207	179	2453	0.073	0.07	0.948 (0.82, 1.088)	9.86
Azith	695	695	646	7084	0.091	0.087	1.17 (1.074, 1.275)	8.1
Doxy	2410	2410	2264	22450	0.101	0.096	1.293 (1.22, 1.368)	7.37
Total	6000	6000	5462	62867	0.087			

Hospitalization Data

Hospitalizations	28 Day Completers	Observed Hosp. Rate	Est. Hosp. Rate (95% interval)
88	2688	0.0327	0.0331 (0.0266, 0.0403)
5	207	0.0242	0.0284 (0.0105, 0.0557)
16	695	0.0230	0.0243 (0.0143, 0.037)
68	2410	0.0282	0.0286 (0.0224, 0.0355)
177	6000	0.0295	

Intervention Status
Complete
Dropped
Dropped
SuccessOnTTRFutilityOnHosp *Announced*

Observed Patient Recoveries

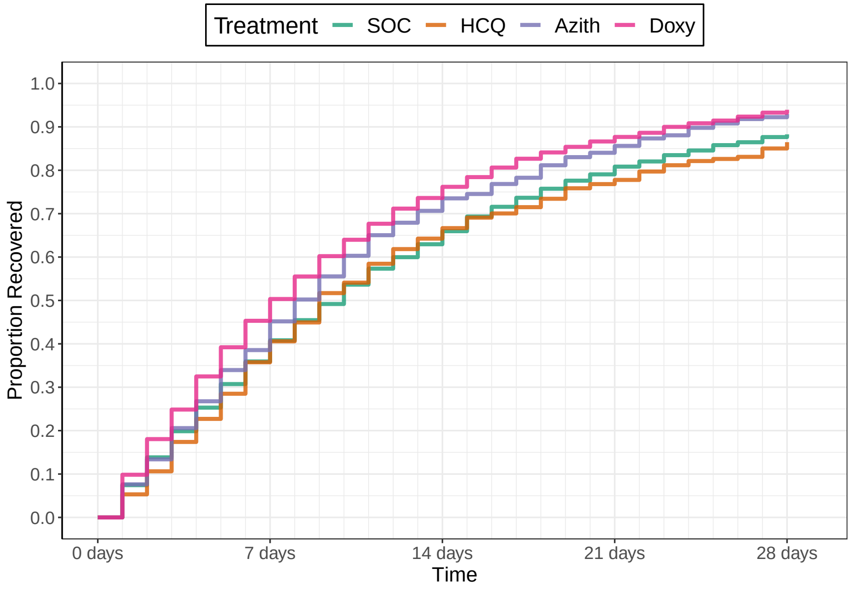

Number Un−recovered

	0 days	7 days	14 days	21 days	28 days
SOC	2688	1722	996	563	332
HCQ	207	133	74	48	31
Azith	695	427	204	111	54
Doxy	2410	1318	636	322	162

Recovery Inferences

	Pr(Superiority)	Pr(Meaningful Effect)	Pr(Best)	Randomization Probability
SOC				
HCQ	0.2353	3e−04	0	
Azith	1	0.271	0	
Doxy	1	0.9847	0.75	

Hospitalization Inferences

Pr(Superiority)	Pr(Meaningful Effect)
0.6968	0.0802
0.9035	0.0392
0.8315	8e−04

Hazard Ratio Estimates

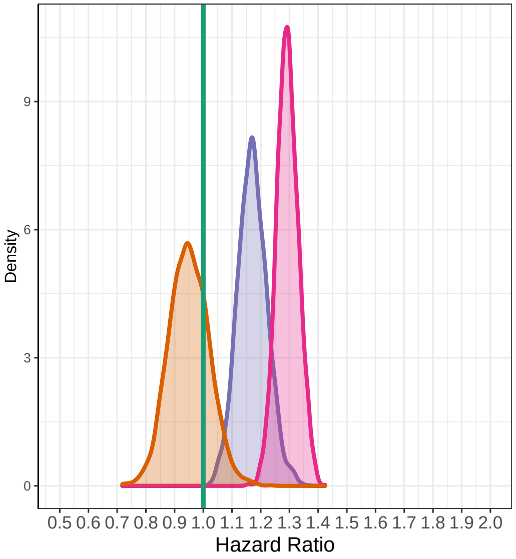

Median Time To Recovery Estimates

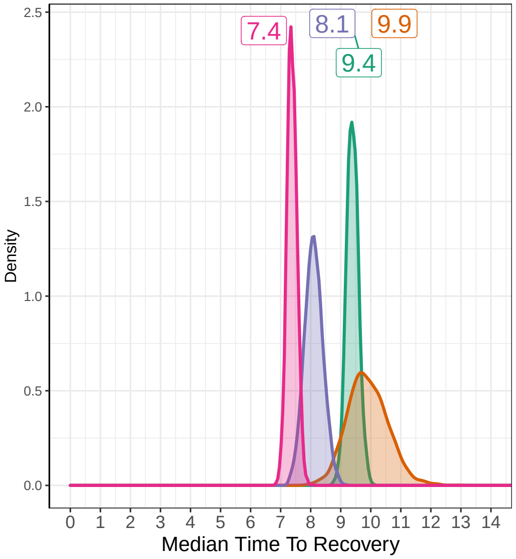

6.6 Computational Details

In the simulations, relevant covariates (age, comorbidity or high risk) are regarded as nuisance variables, and hence are not factored into the simulations. For computational efficiency, the primary analysis for the hospitalization endpoint was simulated using beta-binomial posterior distributions; however logistic regression will be used during execution to account for the covariates (as specified in Section 3.2). Operating characteristics are based on a minimum of 1,000 simulations per scenario. Each Bayesian MCMC fit includes at least 4,000 posterior samples with a burn-in of at least 1,000 samples. In trial execution, at least 10,000 MCMC samples will be taken from the Bayesian posterior distribution after an appropriate burn-in. The R software package was used to summarize the simulation output and to create graphics and tables for this report. This document was typeset with LaTeX.

Appendix 3

Primary Care Clinical Trials Unit

PRINCIPLE
Platform Randomised trial of INterventions against COVID-19 In older peoPLE

MASTER STATISTICAL ANALYSIS PLAN

Platform Randomised trial of INterventions against COVID-19 In older peoPLE

Internal Reference Number / Short title: PRINCIPLE

Ethics Ref: 20/SC/0158

IRAS Project ID: 281958

EudraCT Number: 2020-001209-22

Based on protocol version 6.1 3 30th December 2020 3rd 2020

Version 1.1 Date: 5 January 2021

Version History

	Version Date:	Signature	Date
Written by	Nicola Williams		6 Jan 2021
Reviewed by	Ly-Mee Yu		6 Jan 2021
Reviewed by	Ben Saville		6 Jan 2021
Approved by:	Professor Chris Butler		6 Jan 2020

Version History

Version:	Version Date:	Changes:
0.1	25th March 2020	MS initial draft
0.2	31st March 2020	Updated post JM initial comments
0.3	12 May 2020	JM/UG writing SAP
0.4	10 Aug<br>4th August 2020	Updated to protocol version 4.0<br>NW updating SAP
0.5	11th August 2020	Merging of 2 0.4 versions
0.6	1st October 2020	Updates following comments from JG, ST and CB<br>Update primary outcomes and sample size<br>Remove references to RCGP data as this is stored in the notes review
0,7	15th October 2020	Updates following meetings with JG, ST, LMY<br>Remove much of text from introduction/DMC sections and refer to protocol/DMC charter<br>Update analysis of binary outcomes to logistic regression
0.8	29th October 2020	Updates following review from LMY

		Health service use split into participant reported and GP reported health service use<br>Addition of sustained recovery outcome<br>Addition of appendix III – analysis of HYDROXYCHLOROQUINE arm
0.9	30th October 2020	Further update to definition of sustained recovery. Also changed the definition of duration of severe symptoms.
0.10	3rd November 2020	Update following comments from CB
0.11	4th November 2020	Incorporated Jienchi Dorward & Ben Saville's comments
0,12	6th November 2020	Incorporated Richard Hobbs' comments and made changes following meeting with LMY, RH, CB, BS, JD:<br><br>Updated definition of sustained recovery<br>Change 'consumption' to 'prescription' for antibiotics<br>Addition of WHO ordinal scale
0.13	18th November 2020	Amending typos<br><br>Addition of appendices detailing analysis of azithromycin and doxycycline<br><br>Addition of subgroup analyses
0.14	24th November 2020	Update to protocol v6.0<br>New outcome: new infections in the household<br>Addition of appendix detailing analysis of inhaled corticosteroid arm<br><br>Update subgroup analysis section

		Accept changes from BS and CB from previous version
0.15	26th November 2020	Updates following comments from Philip Hannaford – update to ICS dose and subgroup analyses to include deaths and deaths/hospitalisation combined
1.0	1st December 2020	Change to version 1.0 for signing off
1.1	5th January 2021	Remove viral shedding outcome<br>Update timing of swab results<br>Clarification of censoring for time to event outcomes<br>Addition of subgroup analyses

UNIVERSITY OF OXFORD

TABLE OF CONTENTS

1 INTRODUCTION 8

1.1 PREFACE 8
1.2 PURPOSE AND SCOPE OF THE PLAN 8
1.3 TRIAL OVERVIEW 9
1.4 OBJECTIVES 10

2 TRIAL DESIGN 13

2.1 ADAPTIVE DESIGN 13
2.2 DEFINITIONS 13
2.2.1 Follow-up period 13
2.2.2 Sources of data 13
2.3 OUTCOMES 14
2.3.1 Primary outcome 14
2.3.2 Secondary outcomes 14
2.4 TARGET POPULATION 16
2.5 TREATMENTS 16
2.6 SAMPLE SIZE 16
2.6.1 Virtual trial simulations 17
2.7 RANDOMISATION AND BLINDING IN THE ANALYSIS STAGE 17

3 DATA MANAGEMENT AND DERIVATION OF OUTCOMES 18

3.1 SOURCES OF DATA 18
3.2 PRIMARY OUTCOMES 18
3.2.1 Time to recovery 18
3.2.2 Hospital admission or death 19
3.3 SECONDARY OUTCOMES 20
3.3.1 Patient reported illness severity 20
3.3.2 Duration of severe symptoms 20
3.3.3 Time to Sustained Recovery 20
3.3.4 Time to Sustained symptoms alleviation 21
3.3.5 Contacts with health services between randomisation and day 28 of follow-up. 21
3.3.6 Contacts with health services 21
3.3.7 Prescribing of antibiotics between randomisation and day 28 of FU 22
3.3.8 Hospital assessment without admission 22
3.3.9 Oxygen administration 23
3.3.10 Intensive care unit admission 23
3.3.11 Mechanical ventilation. 24
3.3.12 Duration of hospital admission 24
*3.3.13 Viral shedding .... **Error! Bookmark not defined.***
3.3.14 Negative effect on well-being (WHO-5) 25

3.3.15 WHO Ordinal Scale of Clinical Progression 25
3.3.16 New infections in the household 25
3.3.17 Derivation of subgroups for addressing Objective 12 25

4 ANALYSIS – GENERAL CONSIDERATIONS 26

4.1 PARTICIPANT THROUGHPUT 26
4.2 DESCRIPTIVE STATISTICS 26
4.3 CHARACTERISTICS OF PARTICIPANTS 26
4.4 DESCRIPTION OF AVAILABLE DATA....................27
4.5 COMPLIANCE WITH MEDICATION 27
4.6 DEFINITION OF POPULATION FOR ANALYSIS....................27
4.7 COMPARATIVE ANALYSIS 27
4.8 POOLING OF INVESTIGATIONAL SITES 27
4.9 DATA MONITORING COMMITTEE AND INTERIM ANALYSES 28

5 PRIMARY ANALYSIS 28

5.1 PRIMARY OUTCOMES 28

6 SECONDARY ANALYSIS 29

6.1 PRIMARY OUTCOME ON SUBSET OF PATIENTS WITH CONFIRMED COVID-19 29
6.2 SECONDARY OUTCOMES 29
6.2.1 Patient reported illness severity 30
6.2.2 Duration of severe symptoms 30
6.2.3 Time to Sustained recovery 30
6.2.4 Time to Sustained symptoms alleviation....................30
6.2.5 Contacts with health care services 30
6.2.6 Prescribing of antibiotics 31
6.2.7 Hospital assessment without admission 31
6.2.8 Oxygen administration 31
6.2.9 Intensive care unit admission 31
6.2.10 Mechanical ventilation 31
6.2.11 Duration of hospital admission 32
6.2.12 Viral Shedding .... ***Error! Bookmark not defined.***
6.2.13 Well-being 32
6.2.14 WHO Ordinal Scale of Clinical Progression....................32
6.2.15 New infections in the Household....................32
6.3 HANDLING MISSING DATA....................32
6.4 MULTIPLE COMPARISONS AND MULTIPLICITY 32
6.5 MODEL ASSUMPTIONS 32

7 SENSITIVITY ANALYSIS....................33

8 SUBGROUP ANALYSES....................33

9 SAFETY ANALYSIS....................33

9.1 ADVERSE EVENTS 33

10 VALIDATION **34**

11 CHANGES TO THE PROTOCOL OR PREVIOUS VERSIONS OF SAP **34**

12 APPENDICES **34**

12.1 APPENDIX I. SCHEDULE OF PROCEDURES 34
12.2 APPENDIX II. FLOW DIAGRAM OF TRIAL PARTICIPANTS 36
12.3 APPENDIX III: PLANS FOR ANALYSIS OF HYDROXYCHLOROQUINE 37
Population of Analysis *37*
Descriptive statistics *37*
Primary Outcome Analysis *37*
Secondary Outcome Analysis *37*
Analysis of Safety Data *37*
12.4 APPENDIX IV: PLANS FOR ANALYSIS OF AZITHROMYCIN 38
Population of Analysis *38*
Descriptive statistics *38*
Primary Outcome Analysis *38*
Secondary Outcome Analysis *38*
Analysis of Safety Data *38*
12.5 APPENDIX V: PLANS FOR ANALYSIS OF DOXYCYCLINE 39
Population of Analysis *39*
Descriptive statistics *39*
Primary Outcome Analysis *39*
Secondary Outcome Analysis *39*
Analysis of Safety Data *39*
12.6 APPENDIX VI: PLANS FOR ANALYSIS OF INHALED CORTICOSTEROID BUDESONIDE 40
Population of Analysis *40*
Descriptive statistics *40*
Primary Outcome Analysis *40*
Secondary Outcome Analysis *40*
Analysis of Safety Data *40*

UNIVERSITY OF OXFORD

1 Introduction

1.1 Preface

Chief Investigator: Professor Chris Butler

Co-study lead: Professor Richard Hobbs

Senior Trial Manager(s): Dr Hannah Swayze, Dr Emma Ogburn, Dr Emily Bongard, Julie Allen

Trial Manager: Dr Sharon Tonner

Data manager: Jenna Grabey

Lead Trial Statistician(s): Dr Ly-Mee Yu and Dr Ben Saville

There are two teams of unblinded statisticians involved in the PRINCIPLE trial. The unblinded statisticians in Oxford Primary Care Clinical Trials Unit (PC-CTU) are responsible for data management, derivation of outcomes, transfer of data to the Statistical Analysis Committee (SAC) of Berry Consultants and analysis of the secondary and safety outcomes. The unblinded SAC is responsible for the interim analysis and to provide a summary of results for the Data Monitoring Safety Monitoring Committee (DMSC) members in an interim analysis report. The SAC will be responsible for the co-primary analyses and any related sensitivity and subgroup analyses requiring a similar Bayesian framework.

This version of the Statistical Analysis Plan was written based on protocol version 6.0 2 November 2020, and may be updated in the light of further amendments to the study protocol.

1.2 Purpose and scope of the plan

This Master Statistical Analysis Plan (M-SAP) will detail the statistical design and methods of the PRINCIPLE trial. It will include an appendix titled "Adaptive Design Report" (ADR), which will provide complete specifications for the primary analyses and pre-specified adaptive algorithm. In addition, the M-SAP will be accompanied by arm-specific appendices to describe any planned deviations from the M-SAP. Plans for the analysis of qualitative outcomes is beyond the scope of this statistical analysis plan, and therefore will not be covered in this M-SAP.

Analyses-related decisions may need to be made based on the observed data, such as a review of the distribution of outcome data. These decisions will be made prior to the proposed statistical analyses.

The plan draws on statistical guidance ICH Harmonised Tripartite Guideline: Statistical Principles for Clinical Trials and PSI Guidelines for the Standard Operating Procedures (SOP) for Good Statistical Practice in Clinical Research, the CONSORT statement for operating trials and PC-CTU statistical SOPs.

Analyses will be carried out in accordance with the M-SAP and corresponding appendices. Any additional analysis that is not specified in the M-SAP/appendices or any unplanned deviation(s) from the M-

SAP/appendices will be specified in the Statistical Report. Reasons for these changes will be documented and authorised by the Chief Investigator.

Due to the nature of the design of this trial, results for specific treatments will be analysed while the trial is ongoing. This will be done with prior agreement from the Trial Steering Committee (TSC) and DMSC, and the trial team will remain blind to these analyses until such time as the TSC, informed by data and advice from the DMSC, advise that findings should be declared.

1.3 Trial overview

PRINCIPLE is an open, adaptive, platform trial to evaluate emerging treatments for Covid-like-illness. A “platform trial” is a trial in which multiple treatments for the same disease are tested simultaneously. The backbone of the trial is an adaptive clinical trial design. Pre-specified decision criteria allow for dropping a treatment for futility, declaring a treatment superior, or adding a new treatment to be tested. If at any point a treatment is deemed superior to the Usual Care arm, the superior treatment may replace the Usual Care arm as the new standard of care within the trial. However, the primary analysis of subsequent interventions will correspond to the comparison versus the original Usual Care arm. Because the process of dropping and adding treatments may be on-going for an indefinite period of time, platform trials may be better conceived of as a process rather than a single clinical trial. In the context of the COVID-19 pandemic, the trial may continue as long as the pandemic persists.

The PRINCIPLE trial began as a two arm, 1:1 randomised trial but with the capability to add additional interventions over time. The evaluation of any new interventions is governed by the master protocol and M-SAP (including adaptive algorithm and decision criteria), with any planned deviations from the master protocol and M-SAP to be specified in arm-specific appendices. The inclusion of any new interventions will require additional arm-specific appendices to the master protocol and M-SAP.

The primary analysis population is defined as all randomized participants according to the groups they were randomly allocated to, regardless of deviation from protocol and irrespective of their COVID-19 status.

Further analyses of the primary and secondary outcomes will be carried out based only on the subset of participants with confirmed COVID-19.

1.4 OBJECTIVES

The primary and secondary objectives as well as time points to evaluate these outcome measures as stated in the protocol.

	Objectives	Outcome Measures	Timepoint (s)
Primary	To assess the effectiveness of trial treatments in reducing<br><br>1) Time to recovery, for patients aged ≥50 years with comorbidity, and aged ≥65 with or without comorbidity and possible COVID-19 during a time of prevalent COVID-19 disease	1) Time to recovery, defined as the first instance that a participant reports feeling recovered from suspected COVID-19.	Within 28 days of randomisation<br><br>Patient report, Study Partner report, daily online symptom scores
	2) Hospitalisation and/or death.	2) Hospitalisation and/or death	Within 28 days of randomisation<br><br>Patient report, Study Partner report, medical records
Secondary	To explore whether trial treatment reduces<br><br>1) Patient-reported illness severity<br>2) Duration of severe symptoms<br>3) Time to sustained recovery<br>4) Time to sustained symptom alleviation	Patient reports daily symptoms.	Daily online symptom scores.<br><br>Telephone call or text on days 2, 7, 14 and 28 if data is not obtained through the online diary
	5) Contacts with the health services	Contacts with health services reported by patients and/or captured by reports in patients' medical records	GP notes review if available through Oxford RCGP RSC network; otherwise, other sources of

	Objectives	Outcome Measures	Timepoint (s)
	6) Prescription of antibiotics	primary care medical records	routinely collected data after 28 days<br><br>HES/ONS/EMIS/Medical record data linkage after 28 days if patients have been assessed in hospital for 6 months
	7) Hospital assessment without admission<br>8) Oxygen administration<br>9) Intensive Care Unit admission<br>10) Mechanical ventilation<br>11) Duration of hospital admission	Patient report/carer report/medical record in primary and secondary care	
	12) To determine if treatment effects on the primary and secondary endpoints are specific to those with a positive test for SARS-CoV-2	Swab test results will indicate an "Intention to Treat Infected" group within the overall cohort for sub analysis. Blood test results on recovery (optional) for evidence of historic COVID-19	Swab result from medical records, the supporting laboratory and/or convalescent blood test for evidence of historic COVID-19
	13) Negative effects on well being	WHO-5 Well Being Index	WHO 5 Well Being Index at baseline, day 14, and day 28, either via online diary or telephone
	14) WHO Ordinal scale of clinical progression	Patient report/carer report/medical record in primary and secondary care	Days 7, 14 and 28
	15) New infections in household	Reports of new infections in the household (from daily questionnaire)	Within 28 days of randomisation

	Objectives	Outcome Measures	Timepoint (s)
Qualitative sub-study<br><br>(not covered in this M-Plan)	1. To explore patients' experiences of consulting, being tested and taking (trial) medication for suspected COVID-19.<br><br>2. To explore healthcare professionals' views of taking part in research during pandemics.	1. Telephone interviews with patients.<br><br>2. Telephone interviews with healthcare professionals.	1. After 28 days.<br><br>2. Once practice has completed recruitment.
Intervention(s)	All trial interventions are detailed in the Appendices of the protocol. Further interventions may be added or replaced during the course of the trial, subject to suitable interventions becoming available and all necessary approvals being obtained.		
Comparator	PRINCIPLE began as a two-arm trial, with the intervention arm being Usual Care without the addition of a trial drug. Additional arms may be added as the trial progresses. These will be detailed in the Appendices of the protocol. If an intervention arm is shown to be superior on both co-primary endpoints, then this will be incorporated in the new standard of care. However, the primary analysis of subsequent interventions will correspond to the comparison versus the original Usual Care arm.		

2 Trial design

PRINCIPLE is an open, adaptive, platform trial to evaluate emerging treatments of COVID-like-illness. A “platform trial” is a trial in which multiple treatments for the same disease are tested simultaneously. The backbone of the trial is an adaptive clinical trial design. Pre-specified decision criteria allow for dropping a treatment for futility, declaring a treatment superior, or adding a new treatment to be tested. If at any point a treatment is deemed superior to the control arm, the superior treatment will replace the control arm as the new standard of care, and all subsequent treatments may be compared to the new standard of care within the trial. Because the process of dropping and adding treatments may be on-going for an indefinite period of time, platform trials may be better conceived of as a process rather than a single clinical trial. In the context of the COVID-19 pandemic, the trial may continue as long as the pandemic persists, and while there is a need to evaluate treatments for acute respiratory tract infections in the community.

The PRINCIPLE trial began as a 1:1 randomised trial of standard care versus standard care plus hydroxychloroquine but with the capability to add additional interventions over time. The evaluation of any new interventions is governed by the master protocol, including adaptive and decision criteria. In addition, the inclusion of any new interventions will require amendments and/or supplements to the protocol and SAP.

2.1 Adaptive design

The pre-specified design will allow adaptations to the trial based on the observed data. These adaptations include the declaration of success or futility of an intervention at an interim analysis, the addition or removal of treatment arms, and changes in the randomisation probabilities. Adaptations will occur at a given interim analysis if pre-specified conditions are satisfied. The adaptive algorithm will be documented in the Adaptive Design Report, including pre-specified criteria for decisions regarding futility or effectiveness of interventions and/or replacing interventions in the trial.

2.2 Definitions

2.2.1 Follow-up period

For the primary outcomes, patients will be followed from date of randomisation to 28 days by daily symptom diary and/or telephone, and review of their medical records. Participant records will be accessed up to 3 months following randomisation to ascertain outcome data up to 28 days from randomisation.

2.2.2 Sources of data

There are multiple sources of data

(i) Daily online diaries for 28 days
(ii) Telephone call data collected at 7, 14 and 28 days, if daily online diaries are not completed (participants are called if they miss 2 or more days of their diary)
(iii) Notes review CRF (including data from medical records, primary care clinical records and RCGP surveillance data)

(iv) Death and hospitalisation CRF
(v) Lab results CRF

The derivation of each outcome (See section 3.0) will ensure that all sources of data are considered where appropriate.

Where diary data and telephone data are inconsistent, daily diary data will be used in the derivation of the outcome.

2.3 Outcomes

2.3.1 Primary outcome

There are two co-primary outcomes as listed below:

- Time to recovery from suspected COVID-19 infection within 28 days from randomisation, where recovery is defined as the first instance that a participant reports feeling recovered.
- Hospital admission or death related to suspected or confirmed COVID-19 within 28 days from randomisation

2.3.2 Secondary outcomes

2.3.2.1 Patient reported illness severity

Participants are asked to rate how well they are feeling overall each day on a scale of 1-10 (1 being the worst and 10 being the best). This is captured on the patients' daily diaries and the Call CRF.

2.3.2.2 Duration of severe symptoms

Participants are asked to rate their symptoms of fever, cough, shortness of breath, muscle ache, nausea/vomiting, diarrhoea, and generally unwell on a four point scale from 0=no problem, 1=mild problem, 2=moderate problem and 3=major problem.

This is captured on patients' daily diaries and Call CRF

The outcomes to be analysed will be time to alleviation of symptoms and time to initial reduction in severe symptoms.

2.3.2.3 Time to Sustained recovery

Whether a patient has reached 'sustained recovery' will be determined from the participant diaries and the telephone call CRFs based on the question "do you feel recovered today?"

2.3.2.4 Time to Sustained alleviation of symptoms

Participants are asked to rate their symptoms of fever, cough, shortness of breath, muscle ache, nausea/vomiting, diarrhoea, and generally unwell on a four point scale from 0=no problem, 1=mild problem, 2=moderate problem and 3=major problem.

This is captured on patients' daily diaries and Call CRF

2.3.2.5 Contacts with health services between randomisation and day 28 of follow-up (FU).

This is captured on patients' daily diaries, telephone call CRFs, and medical notes review

2.3.2.6 Consumption of antibiotics between randomisation and day 28 of FU

Notes review at 28 days will record information regarding prescription of antibiotics.

2.3.2.7 Hospital assessment without admission over night

This is captured on patients' daily diaries, telephone call CRFs, notes review and hospitalisation and death CRF.

2.3.2.8 Oxygen Administration

This is captured in patients' daily diaries, telephone call CRF, Notes review and hospitalisation and death CRF.

2.3.2.9 Intensive care unit admission

This is captured in patients' daily diaries, telephone call CRF, Notes review, and hospitalisation and death CRF.

2.3.2.10 Mechanical ventilation

This is captured in patients' daily diaries, telephone call CRF, Notes review and hospitalisation and death CRF.

2.3.2.11 Duration of hospital admission

Patient report/carer report/medical record in primary care and hospital care in relation to duration of hospital admission between date of randomisation and day 28 of follow-up.

This is captured in patients' daily diaries, Call CRF, Notes review and hospitalisation and death CRF.

2.3.2.12 Negative effects on well-being (WHO-5)

The WHO-5 well-being index is collected from daily diaries or telephone call at baseline, day 14 and day 28.

2.3.2.13 WHO Ordinal Scale of Clinical Progression

This is a score based on a number of factors including hospitalisation, use of oxygen, ventilation and death. It ranges from 1 (not hospitalised) to 6 (dead).

2.3.2.14 New infections in the Household

This is collected in the daily diary and telephone call CRF through the question "has anybody else in your house become unwell today with a respiratory illness?"

2.4 TARGET POPULATION

The trial aims to include patients aged ≥50 years with comorbidity, and patients aged ≥65 with or without comorbidity with confirmed or suspected COVID-19 who meet the UK case definition for possible COVID-19, and who are well enough to remain in the community. This definition can be found here:

https://www.gov.uk/government/publications/wuhan-novel-coronavirus-initial-investigation-of-possible-cases/investigation-and-initial-clinical-management-of-possible-cases-of-wuhan-novel-coronavirus-wn-cov-infection

The study is for people who have ongoing symptoms.

See protocol for detailed inclusion and exclusion criteria.

2.5 TREATMENTS

Based on version 6.0 of the protocol the main randomisation will be between the following treatment arms (although not all treatments may be available at any one time and not all participants are eligible for all treatments). However, due to the nature of the trial design, treatments can be added and dropped as appropriate.

- Usual care
- Usual care plus hydroxychloroquine, 200mg twice daily for 3 days (discontinued)
- Usual care plus azithromycin, 500mg once daily for 3 days
- Usual care plus doxycycline, 200mg on day 1 followed by 100mg daily for 6 days
- Usual care plus inhaled corticosteroid budesonide, 400mcg daily (as two puffs bd) for 14 days

Subsequent reference to a treatment group refers to treatment plus usual care, and subsequent reference to usual care group refers to the usual care without a study drug.

2.6 SAMPLE SIZE

Given the open perpetual trial structure, the trial does not have a prespecified end based on sample size. Rather, the trial will continue until either superiority or futility is claimed for each intervention, or until the pandemic expires in the population. We estimate that approximately 400 participants per arm (800 participants total if only a single intervention vs. Usual Care) will be required to provide 90% power for detecting a hazard ratio of 1.3 (approximate difference of 2 days in median recovery time). This calculation is based on the assumption of an exponential distribution for time to recovery with a median of 9 days in the Usual Care arm, with some adjustments for missing data and multiple interim analyses. On average, we expect fewer participants to be required when there is a large treatment benefit or complete lack of benefit. For example, if the true hazard ratio is 1.5 (3 day benefit in median time to recovery), on average only 150 subjects per arm are required to provide sufficient power. The primary advantage of the adaptive design is the ability to adapt the sample size to the observed data, thus addressing the primary hypothesis as quickly and as efficiently as possible.

We estimate that approximately 1500 participants per arm (3000 participants total if only a single intervention vs. usual care) will be required to provide 90% power for detecting a 50% reduction in the relative risk of hospitalisation and/or death. This calculation is based on the assumption of an underlying 5% combined hospitalisation and/or death rate in the Usual Care arm, with an intervention lowering the hospitalisation and/or death rate to 2.5%, with some adjustments for the multiple interim analyses. We expect fewer participants to be required to detect a 50% reduction if the event rate in the Usual Care arm is greater than 5%.

2.6.1 Virtual trial simulations

Because of the adaptive platform trial structure, there exists no simple formula(s) to calculate power and Type I error (false positive rate). Hence, virtual trial simulations will be used to fully characterize and quantify the power and Type I error of the design. These simulations will be conducted prior to the first interim analysis (with results described in the Adaptive Design Report), and will be used to optimize the adaptive decision criterion and RAR parameters. The simulations will include a comprehensive evaluation of trial performance across a wide range of assumptions (e.g. underlying distribution of outcome in Usual Care arm, treatment effect, accrual rates, etc.). This will include summaries regarding the number of subjects required to make a success or futility conclusions for each intervention. Complete details of the simulations will be provided in the Adaptive Design Report.

2.7 Randomisation and blinding in the analysis stage

Participants will be randomised using a fully validated and compliant web-based randomisation system called Sortition. Once deemed eligible, the clinician or a member of the trial team will randomise the participant. The randomisation process will take only a few moments via the online system and will not delay trial participation. Participants will be randomised to the arm/arms they are eligible for (at least two arms), automatically by Sortition.

Initially, randomisation will be fixed 1:1 for a comparison between two trial arms, with stratification by age (less than 65, greater than or equal to 65), and comorbidity (yes/no). If a second intervention arm is added to the study, randomisation allocation will be modified and the additional intervention will be included in the interim analyses (with evaluation for success and futility) as detailed in the Adaptive Design Report. If there are at least 3 arms (2) treatment and Usual Care) in the study, each interim analysis may incorporate modified randomisation probabilities via response adaptive randomisation (RAR). Full details for implementing RAR will be provided in the Adaptive Design Report; the general idea is to allocate more participants to the intervention arms that have the best observed outcomes.

PRINCIPLE is an open-label trial. The participant, a person guiding the participant through the randomisation process, and the participants primary care clinician will know the participant's allocation. Therefore, no unblinding or code breaking is required in the event of a relevant emergency. However, those managing the data will be blind to participant allocation.

The trial team and recruiting clinicians will be blinded to emerging results. During the course of the trial, only those on the Data Safety & Monitoring Committee will have access to the unblinded interim results.

3 Data management and Derivation of outcomes

3.1 Sources of Data

Data is collected from multiple sources. For the derivation of outcomes, should data be collected from more than one source, we will specify which source should be utilised. For example, if data for an outcome are obtained from both daily diary and telephone calls, the daily diary data will be utilised first.

(i) Death and hospitalisation CRF
(ii) Online daily diaries for 28 days
(iii) Telephone call CRF at day 7, 14 and 28
(iv) Lab results CRF
(v) Notes review CRF
(vi) When available, the Secondary Uses Services data, which is a collection of healthcare data in England provided by NHS Digital, and other sources of hospital data.

Data management for derivation of primary and secondary outcomes will be carried out by PC-CTU statisticians, and primary outcomes (and any data that are relevant to the analysis of the primary outcomes) data transferred to SAC.

3.2 Primary outcomes

3.2.1 Time to recovery

The first primary outcome is time to recovery from suspected COVID-19 infection within 28 days from randomisation, where recovery is defined as the first instance that a participant reports feeling recovered.

Time (in days) taken to self-reported recovery will be computed as time to reported "Yes" to the question "Do you feel recovered today (I.e. symptoms associated with illness are no longer a problem)?". The variable is recorded as (**WELLYN**=1). This will be calculated as date from randomisation to date (VISDAT_P) of participant self-report of recovery if using the patient diary. If the call CRF is being used then the date to feeling fully recovered is '**WELLDAT**'.

If a participant has more than one date for when they reported feeling recovered (i.e. relapse or more than one data source completed), the date of first reported recovery will be taken.

If the participant is in hospital on the date of feeling fully recovered then this will still be counted as a recovery (hospitalisation recorded in any 1 or more of the following sources: notes review; death and hospitalisation CRF; daily diaries; call CRF).

3.2.2 Hospital admission or death

The second primary outcome is hospital admission or mortality related to suspected or confirmed COVID-19 measured within 28 days from randomisation.

3.2.2.1 Derivation of mortality

Mortality related to suspected COVID-19 within 28 days of randomisation

Data collected via the death and hospitalisation CRF and the notes review will be used to derive the primary outcome. If the following is true from either source then the patient will be recorded as having death related to suspected COVID-19 within 28 days of randomisation.

DDYN =1 AND date of death (**DDDAT**) ≤28 days from randomisation AND COVID-19 contributed to death [**DD_CV19=1**].

If death is recorded as "Yes" but the COVID-19 related variable is missing, then the outcome will be cross checked with the SAE data. Any death not related to suspected COVID-19 should be recorded as an SAE.

3.2.2.2 Derivation of hospital admission

Hospital admission within 28 days will be defined as an overnight stay in hospital and likely to be related to COVID-19. This can be recorded from the daily diaries, calls at days 7, 14 and 28, notes review CRF and death and hospitalisation CRF. If any of the sources indicate a hospital admission suspected to be related to COVID-19 within 28 days of randomisation then this variable will be coded as "Yes".

3.2.2.2.1 Death and hospitalisation CRF

Participant attended hospital [**HOSP_HOOCCUR = 1**] AND attendance likely related to COVID-19 [**CVYN** = 1] AND [number of days in hospital > 1 or not yet discharged [**HOSP_HOENYN=0**] AND date they were admitted to hospital (**HOSP_HOSTDAT**) is ≤28 days from date of randomisation.

3.2.2.2.2 Daily diary data

Have you attended hospital in the last 24 hours [**HOSP_HOOCCUR** = 1] AND attendance likely related to COVID-19 [**CVYN** = 1] AND admitted overnight [**HOSP_NIGHTYN** = 1] AND first date of overnight hospital stay (**HOSP_HOSTDAT**) is ≤28 days from date of randomisation.

3.2.2.2.3 Calls at 7, 14, 28 days

Admitted to hospital [**HOSP_HOOCCUR** = 1] AND was the admission related to suspected COVID-19 infection [**CVYN** = 1] AND admitted overnight [**HOSP_NIGHTYN** = 1] AND first date of overnight hospital stay (**HOSP_HOSTDAT**) is ≤28 days from date of randomisation.

3.2.2.2.4 Notes review

Participant attended hospital [**HOSP_HOOCCUR** = 1] AND attendance likely related to COVID-19 [**CVYN** = 1] AND admitted overnight [**HOSP_NIGHTYN**=1] AND date they went to hospital is ≤28 days from date of randomisation.

If either hospital admission (as derived above) OR mortality (as derived above) occurs then this primary outcome has occurred (Primary outcome = 1).

Early versions of online daily diaries and call CRF did not include a qualification for suspected COVID-19 infection so these will be cross checked with the SAE data. Any hospitalisation or death not thought to be related to suspected COVID-19 should be recorded as an SAE

3.3 Secondary outcomes

3.3.1 Patient reported illness severity

Participants are asked to rate how well they are feeling each day on a scale of 1-10 (1 being the worst and 10 being the best). This is captured on the participants' daily diaries and the Call CRF.

Data from each available day will be included in the analysis, with day 7, 14 and 28 data obtained from the call CRF if not available in the daily diary. Participants will have the potential for a maximum of 28 responses.

3.3.2 Duration of severe symptoms

Participants are asked to rate their symptoms of fever, cough, shortness of breath, muscle ache, nausea/vomiting, diarrhoea, and generally feeling unwell on a four point scale from 0=no problem, 1=mild problem, 2=moderate problem and 3=major problem until they feel recovered.

'Severe' symptoms will be defined as a score of 3 (major) on the four point rating scale.

3.3.2.1 Time to alleviation of symptoms

Time to alleviation of symptoms will be defined as the time from randomisation to all symptoms being rated as mild or none. For those who have call data only, the time to alleviation will be defined as the day the call was made. For those who only had symptoms rated at baseline as mild or none they will be censored at time 0. Participants who withdraw or are lost to follow-up will be censored at their last contact date.

3.3.2.2 Time to initial reduction of severity of symptoms

Time to initial reduction of severity of symptoms will be defined as time to reduction in severity of each individual symptoms to at least one grade lower. For those who have call data only, the time that the symptoms reduced to at least one grade lower will be defined as the day the call was made. This will be calculated for each symptom and also for overall symptoms. Participants who withdraw or are lost to follow-up will be censored at their last contact date. Participants with symptoms rated as none at baseline will be censored at time 0.

3.3.3 Time to Sustained Recovery

Time to sustained recovery will be defined as the time to first reported recovery on the question 'Do you feel recovered today?' with no subsequent responses of 'no' to this question up to 28 days. For

those who have call data only, the time to sustained recovery will be defined as the day the call was made. Participants who withdraw or are lost to follow-up will be censored at their last contact date.

3.3.4 Time to Sustained symptoms alleviation

This will follow the same principle as time to initial reduction in severity of symptoms but there must be no subsequent symptom severity recorded as moderate or major. This will be calculated for individual symptoms separately and also for overall symptoms.

3.3.5 Contacts with health services between randomisation and day 28 of follow-up.

This will be split into participant reported health service use and health service use from GP records.

3.3.6 Contacts with health services

3.3.6.1 Participant reported health service use

Sources of data for this outcome are participant daily diaries and telephone call CRF (day 7, day 14 and day 28).

This will be presented as 2 outcomes:

1. A binary outcome indicating whether the participant has had any contact with health services during 28 days of follow-up. This will be 'yes' if any of the following are recorded as yes in the daily diary or call CRF:

    - GP (**GP_HOOCCUR**)
    - Other primary care services (**PCS_HOOCCUR**)
    - NHS 111 and other central advice resources (**NHS_HOOCCUR**)
    - A&E (**AE_HOOCCUR**)
    - Hospital (**HOSP_HOOCCUR**)
    - Other (**OTH_HOOCCUR, OTH_HOOCCUR_DEF**– free text)

2. A continuous variable of the number of health service contacts whilst alive during 28 days of FU.

    The number of health service contacts from the diary data will be the total number of times the participant has responded 'yes' to any of the following:

    GP_HOOCCUR, PCS_HOOCCUR, NHS_OOCCUR, AE_OOCUR, OTH_OOCCUR

    If the diary data is missing then the call CRF data will be utilised. The number of health service contacts from the call CRF will be the total of the following for each of the 7, 14 and 28 day calls:

GP_HOOCCURNUM, PCS_HOOCCURNUM, NHS_HOOCCURNUM, AE_HOOCCURNUM, OTH_HOOCCURNUM

3.3.6.2 Health Service Use From GP Records

Data for this outcome will come from the GP notes review after 28 days.

This will be presented as 2 outcomes:

1. A binary outcome indicating whether the participant has had any contact with health services during 28 days of FU. This will be 'yes' if any of the following are recorded as yes in the notes review:
    - GP (**GP_HOOCCUR**)
    - Other primary care services (**PCS_HOOCCUR**)
    - NHS 111 and other national resources (**NHS_HOOCCUR**)
    - A&E (**AE_HOOCCUR**)
    - Hospital (**HOSP_HOOCCUR**)
    - Other (**OTH_HOOCCUR, OTH_HOOCCUR_DEF**– free text)

2. A continuous variable of the number of health service contacts whilst alive during 28 days of FU. This will be derived by totalling the following for those with a notes review:

    GP_NUM, PCS_NUM, NHS_NUM, AE_NUM, OTH_NUM

3.3.7 Prescribing of antibiotics between randomisation and day 28 of FU

Notes review at 28 days will record information regarding prescription of antibiotics.

- Antibiotic prescribed (Yes = 1, No=1) (**ATBYN**)

This outcome will consider consumption of antibiotics whilst alive to account for truncation by death.

3.3.7.1 Derivation of outcome

Antibiotic prescribed = Yes, IF prescribed antibiotic [**ATBYN**=1] AND start date of antibiotic ≤28 days from data of randomisation. If case note review is available for a participant but no information is provided regarding antibiotics, it will be assumed that NO antibiotic was prescribed. If no notes review is available for a participant this will be recorded as missing with respect to antibiotic prescribing.

3.3.8 Hospital assessment without admission

This is captured on patients' daily diaries, Call CRF, notes review and the death and hospitalisation CRF. All sources will be considered and if any indicates a hospital assessment without admission it will be coded as such. If there are discrepancies between the sources of data the diary data will be assumed to be correct.

3.3.8.1 Derivation

Notes review, patient diary and call CRF:

Hospital assessment without admission = YES if on any day the participant reports going to hospital [**HOSP_HOOCUR** = Yes] AND on that date they were not admitted overnight [**HOSP_NIGHTYN** = No]

Death and hospitalisation CRF:

Hospital assessment without admission = YES if the patient has been admitted to hospital since they joined the trial (**HOSP_HOOCCUR**=1) and Date of admission (**HOSP_HOSTDAT**) = date of discharge (**HOSP_HOENDAT_DD**)

3.3.9 Oxygen administration

This is captured in patients' daily diaries, Call CRF, Notes review and death and hospitalisation CRF.

This outcome will reflect oxygen administration whilst alive to account for truncation due to death

3.3.9.1 Derivation

Oxygen administration = yes if patient reports receiving oxygen whilst in hospital [**OXY_HOOCCUR**=Yes] AND date of admission to hospital is ≤28 days from date of randomisation.

If oxygen use is reported via any data source then it will be counted as yes.

3.3.10 Intensive care unit admission

This is captured in patients' daily diaries, Call CRF, Notes review and the death and hospitalisation CRF. It will be calculated in 2 ways:

1. To reflect ICU admission whilst alive to account for truncation due to death prior to ICU admission. This definition of the outcome would give an estimate of the requirement for ICU between the randomised groups from a healthcare resource use perspective. This is derived as:

   Intensive care unit admission = yes if report of participant staying in ICU [ICUYN=Yes or ICU_HOOCCUR=Yes] AND date of admission to ICU is ≤28 days from date of randomisation. If intensive care unit admission is reported via any data source then it will be counted as an ICU admission.

2. To consider this outcome from a patient benefit perspective the outcome will be defined as a composite by assigning a "poor" outcome (i.e. ICU admission) to participants who die before requiring ICU admission. This is derived as:

Intensive care unit admission = yes if report of participant staying in ICU [**ICUYN**=Yes or **ICU_HOOCCUR**=Yes] AND date of admission to ICU is ≤28 days from date of randomisation) OR participant has died.

If an ICU admission is recorded on any data source then it will be coded as an ICU admission.

3.3.11 Mechanical ventilation.

a. This outcome will reflect mechanical ventilation whilst alive to account for truncation due to death. This definition of the outcome would give an estimate of the requirement for ventilation between the randomised groups from a healthcare resource use perspective.
b. To consider this outcome from a patient benefit perspective the outcome will be defined as a composite by assigning a "poor" outcome to participants who die before requiring ventilation.

This is captured in patients' daily diaries, Call CRF, Notes review and death and hospitalisation CRF and captured from the question related to receiving mechanical ventilation.

3.3.11.1 Derivation

(a) Mechanical ventilation = yes if report of participant on mechanical ventilation [**MV_HOOCCUR**=Yes] AND date of admission to hospital is ≤28 days from date of randomisation.
(b) Mechanical ventilation = yes if participant dies OR (participant on mechanical ventilation AND date of admission to hospital is ≤28 days from date of randomisation [**MV_HOOCCUR**=Yes])

If mechanical ventilation is recorded on any data source then it will be coded as mechanical ventilation=YES.

3.3.12 Duration of hospital admission

This is captured in patient diaries, Call CRF, notes review and death and hospitalisation CRF.

The duration of hospital admission is calculated in the following ways from the different data sources:

Notes review: **HOSP_DUR**

Death and hospitalisation CRF: Difference between date of admission and date of discharge

Participant diaries: **HOSP_DUR**

Call CRF: **HOSP_NONIGHTS**

If a participant has been admitted with suspected COVID-19 more than once in the FU period of 28 days, the duration of hospital stay will be the sum of all admissions during FU, truncated at day 28.

All sources of data will be considered and if any indicates a hospital stay it will be coded as such. If there are discrepancies between the sources of data the diary data will be assumed to be correct.

3.3.13 Negative effect on well-being (WHO-5)

Well-being is measured using the WHO well-being index which includes 5 items relating to well-being measured on a five point scale (scale of 5 =all of the time, 4=most of the time, 3=more than half the time, 2=less than half the time, 1=some of the time, 0=at no time). A total score is computed by summing the scores to the five individual questions to give a raw score ranging from 0 to 25 which is then multiplied by 4 to give the final score from 0 representing the worst imaginable well-being to 100 representing the best imaginable well-being. Negative effect on well-being is collected at baseline, 14 days and 28 days via the daily diary and call CRF.

From a patient benefit perspective participants who die before the measured time point will be given a score of 0 for that time point and included in the analysis.

3.3.14 WHO Ordinal Scale of Clinical Progression

There are various versions of this scale. We will use a score based on a number of factors including hospitalisation, use of oxygen, ventilation and death. It ranges from 1 (not hospitalised) to 6 (dead) and is defined as follows:

1 = Not hospitalised

2 = Hospitalised without need for supplemental oxygen

3 = Hospitalised with need for supplemental oxygen

4 = Hospitalised with need for non-invasive positive pressure ventilation or high-flow nasal cannula

5 = Hospitalised with need for mechanical ventilation or extracorporeal membrane oxygenation

6 = Death

This outcome will be derived at days 7, 14 and 28.

3.3.15 New infections in the household

This will be coded as 'yes' if the answer to the question "has anybody else in your house become unwell today with a respiratory illness?" is yes in the daily diary and/or the telephone call CRF at any time during the 28 days after randomisation.

3.3.16 Derivation of subgroups for addressing Objective 12

Participants will be categorised as being infected or not, based on any swab result between randomisation and 28 days. Swab results data is contained in the baseline CRF, Lab results CRF and notes review CRF.

Any swab result obtained between randomisation and 28 days after the date of randomisation, will be used to classify participants into three groups. A positive test result at any time will put them into group 1, regardless of other negative results;

1. COVID-19 positive
2. COVID-19 Negative
3. No swab result available

4 Analysis – General considerations

The following sections detail the final analysis for any treatment comparison within 28 days of randomisation. Methods for the primary analyses, including interim analyses, are specified in the Adaptive Design Report.

4.1 Participant throughput

The flow of participants through the trial will be reported following CONSORT and will include number of participants randomly assigned, receiving allocated treatment, followed up, withdrawn and analysed for primary outcome. Protocol deviations and information regarding screening information and number of ineligible participants randomised will be reported.

4.2 Descriptive statistics

Baseline characteristics of participants will be summarised both overall and by randomised group, including stratification factors and important prognostic, demographic and clinical characteristics. Binary and unordered categorical variables will be summarised using number, number missing and proportions. Continuous variables that are approximately normally distributed will be summarised using number, number missing, mean and standard deviation. Continuous variables that are not normally distributed or ordered categorical variables will be summarised using number, number missing, median and interquartile range.

There will be no tests of statistical significance nor confidence intervals for differences between randomised groups with respect to any baseline variable.

4.3 Characteristics of participants

Characteristics of participants to be described include age, sex (male/female/other), presence of comorbidities (Asthma COPD or other lung disease, Diabetes, Heart problems, High blood pressure, Liver disease, Stroke or other neurological problem), duration of symptoms prior to randomisation, symptoms (fever, cough shortness of breath, muscle ache and nausea/vomiting, other) rated as no problem, mild, moderate or major, medications, use of antibiotics , contact with health care services (GP, other primary care services, NHS 111, A&E, Hospital and other), test results for SARS-CoV-2 infection (not tested, missing, positive, negative), care home residency and ethnicity (collected at 28 days in addition to baseline in case these data were missed at baseline). Stratification factors will be described.

4.4 DESCRIPTION OF AVAILABLE DATA

The number of participants with available data for primary and secondary outcomes for the final analysis will be reported by treatment group.

Details describing methods for dealing with missing data with respect to the primary outcome will be described within the Adaptive Design Report (ADR).

4.5 COMPLIANCE WITH MEDICATION

Participants are asked in the online daily diaries to record whether they have taken their medication and if not, the reason why. The call CRF records the number of days they took the trial medication. For participants in randomised groups receiving medication, compliance with medication will be reported. The number of days that the allocated medication was taken will be reported along with withdrawals from treatment.

4.6 DEFINITION OF POPULATION FOR ANALYSIS

The primary analysis population is defined as all randomised participants for whom data were available with participants analysed according to the groups they were randomly allocated to, regardless of deviation from protocol and irrespective of their COVID-19 status.

A second analysis population will be the covid-19 positive population, on which all primary and secondary outcomes will also be analysed.

Safety analysis will be conducted on the as treated population (i.e the treatment that participants have received).

4.7 COMPARATIVE ANALYSIS

For all outcomes the primary analysis will be performed on the primary analysis population at 28 days after randomisation.

Each treatment arm will be compared with the usual care arm. If a treatment is deemed superior to usual care on both co-primary endpoints and replaces the usual care arm as the new standard of care, subsequent treatments will be compared with the original usual care arm.

There will be no formal adjustment for multiple comparisons.

4.8 POOLING OF INVESTIGATIONAL SITES

Data from all sites will be combined and analysed collectively. A sensitivity analysis of the primary outcomes may be carried out adjusting for geographical clinical research network (CRN) if deemed necessary.

4.9 Data Monitoring Committee And Interim Analyses

Details of the data monitoring committee and interim analyses can be found in the interim analysis report and the DMC charter.

5 PRIMARY ANALYSIS

5.1 Primary outcomes

Unless otherwise specified in the intervention-specific appendices, the co-primary outcomes will be analysed using a "gate-keeping" strategy. For a given treatment, time to recovery will be analysed first, and if the first null hypothesis is rejected, the second co-primary endpoint of hospitalization/death will be subsequently analysed. This gate-keeping strategy preserves the overall Type I error of the primary endpoints without additional adjustments for multiple hypotheses. In addition, the gate-keeping structure reflects the clinical belief that an intervention is unlikely to demonstrate benefit on the hospitalization/death endpoint without first demonstrating benefit on the time to recovery endpoint.

The first primary analysis is a Bayesian piecewise exponential of time to recovery regressed on treatment and stratification covariates (age, comorbidity or high risk). Complete details of the model are described in the adaptive design report. Let θ_j for $j > 0$ denote the log hazard ratio for time to recovery for persons on intervention j versus the Usual Care arm ($j = 0$), where $\theta_j > 0$ corresponds to faster recovery. Based on a Bayesian posterior distribution of θ_j, the primary analysis for intervention j will test the following hypothesis:

$$H_0: \theta_j \leq 0 \qquad (1)$$

$$H_1: \theta_j > 0$$

If the Bayesian posterior probability of beneficial treatment effect (ψ_{1j}) in equation (2) is greater than or equal to 0.99,

$$\psi_{1j} = \Pr(\theta_j > 0) \qquad (2)$$

the null hypothesis will be rejected and the intervention will be deemed superior to Usual Care. The decision criteria controls the one-sided Type I error of each intervention at approximately 0.025.

The second co-primary analysis is a Bayesian generalized linear model of the primary outcome regressed on treatment and stratification covariates (age, comorbidity or high risk). Complete details

of the model are described in the adaptive design report. Let p_j denotes the probability of hospitalization/death for persons in treatment group j, where $j = 0$ denotes the Usual Care arm and $j = 1$ denotes an intervention arm. A Bayesian posterior distribution will be derived for the estimated difference in probability of hospitalization/death between treatment groups. Let δ_j denote the log odds ratio of hospitalization/death comparing intervention j to Usual Care. The primary analysis for intervention j will test the following hypothesis:

$$H_0: \delta_j \geq 0 \qquad (3)$$

$$H_1: \delta_j < 0$$

If the Bayesian posterior probability of beneficial treatment effect ψ_{2j} is greater than or equal to 0.975, with ψ_{2j} given in (4),

$$\psi_{2j} = \Pr(\delta_j < 0) \qquad (4)$$

the null hypothesis will be rejected and the intervention will be deemed superior to Usual Care with respect to Hospitalization/Death. Note the decision criterion is lower than the recovery endpoint decision criterion (due to the gate-keeping structure), and controls the one-sided Type I error of each intervention at approximately 0.025 for plausible scenarios.

The primary analysis population is defined as all randomized participants for whom data are available, and are analysed according to the groups they are randomized to. Secondary analyses will conduct the primary analysis on the subset of participants with confirmed COVID-19.

6 SECONDARY ANALYSIS

6.1 PRIMARY OUTCOME ON SUBSET OF PATIENTS WITH CONFIRMED COVID-19

The primary outcomes will be analysed using the same method as detailed in the adaptive design report, but using only those patients with confirmed COVID-19. Modifications to the model (e.g. fewer parameters and/or covariates) may be necessary if sample sizes are insufficient.

6.2 SECONDARY OUTCOMES

For all secondary outcomes, the analysis will compare each treatment arm with the usual care arm, in a pairwise comparison. For each analysis only concurrent controls who meet the same eligibility criteria as the intervention will be included. Regression models (appropriate for each endpoint) will include randomised group (treatment/usual care) and stratification factors (age (continuous), comorbidity (Yes/no)). They will also include duration of symptoms at randomisation. Should it be necessary to compare more than one intervention with control at the same time, a covariate indicating which arms of the trial the participant was eligible to be randomised to will also be included. For binary outcomes with a low event rate, results will be reported descriptively by treatment group and

a Chi-square test or Fisher's exact test may be used instead of the analysis detailed below. For continuous outcomes where the data are skewed, alternative non-parametric methods will be considered.

6.2.1 Patient reported illness severity

A linear regression model will be used to analyse this outcome. The illness severity at each time point (1-28 days) will be included as the response variable, along with randomised group, age, presence of comorbidity and duration of symptoms as fixed effects. Time will be included as a continuous variable.

6.2.2 Duration of severe symptoms

6.2.2.1 Time to alleviation of symptoms

Time to alleviation of symptoms will be compared between each treatment arm with the usual care arm using Cox proportional hazards model, adjusting for randomised group, age and presence of comorbidity at baseline. The model will also be adjusted for duration of symptoms (days) prior to randomisation. This will be calculated as the date of randomisation minus the start date of symptoms as reported on the screening CRF. If this date is unavailable then the date the patient registered with Principle will be used. For those with no symptoms recorded as moderate or severe at baseline they will be considered censored at time 0. The adjusted hazard ratio and 95% CI will be estimated from the model. A Kaplan Meier plot will also be presented. If the assumption of proportionality is not met, then an alternative survival model such as restricted mean survival method will be used.

Separate analyses will be carried out for overall and for each symptom separately.

6.2.2.2 Time to initial reduction of severity of symptoms

This will be analysed using the method described for time to alleviation of symptoms.

6.2.3 Time to Sustained recovery

This will be analysed using the method described for time to alleviation of symptoms.

6.2.4 Time to Sustained symptoms alleviation

This will be analysed using the method described for time to alleviation of symptoms.

6.2.5 Contacts with health care services

The number and percentage of participants with at least one contact with health services will be presented for treatment and usual care groups. This outcome will be analysed using a logistic regression model. Randomised group, age and presence of comorbidity will be included covariates. The adjusted odds ratios will be reported for each pairwise comparison along with their associated

95% confidence interval and P-value. In addition, the adjusted absolute risk differences will be reported.

The number of contacts with health care services over 28 days will be analysed using a Poisson model. Randomised group, age and presence of comorbidity will be included as covariates. Adjusted incidence ratios will be presented with their 95% confidence intervals and related P value.

6.2.6 Prescribing of antibiotics

The count and percentage of participants with an antibiotic prescription will be presented for each treatment group. This outcome will be analysed using a logistic regression model. Randomised group, age and presence of comorbidity will be included covariates. The adjusted odds ratios will be reported for each pairwise comparison along with their associated 95% confidence interval and P-value. In addition, the adjusted absolute risk differences will be reported.

6.2.7 Hospital assessment without admission

The count and percentage of participants with hospital assessment without admission will be presented for each treatment group. This outcome will be analysed using a logistic regression model. Randomised group, age and presence of comorbidity will be included covariates. The adjusted odds ratios will be reported for each pairwise comparison along with their associated 95% confidence interval and P-value. In addition, the adjusted absolute risk differences will be reported.

6.2.8 Oxygen administration

The count and percentage of participants with oxygen administration will be presented for each treatment group. This outcome will be analysed using a logistic regression model. Randomised group, age and presence of comorbidity will be included covariates. The adjusted odds ratios will be reported for each pairwise comparison along with their associated 95% confidence interval and P-value. In addition, the adjusted absolute risk differences will be reported.

6.2.9 Intensive care unit admission

The count and percentage of participants with intensive care unit administration will be presented for each treatment group. This outcome will be analysed using a logistic regression model. Randomised group, age and presence of comorbidity will be included covariates. The adjusted odds ratios will be reported for each pairwise comparison along with their associated 95% confidence interval and P-value. In addition, the adjusted absolute risk differences will be reported.

6.2.10 Mechanical ventilation

The count and percentage of participants with mechanical ventilation will be presented for each treatment group. This outcome will be analysed using a logistic regression model. Randomised group, age and presence of comorbidity will be included covariates. The adjusted odds ratios will be reported for each pairwise comparison along with their associated 95% confidence interval and P-value. In addition, the adjusted absolute risk differences will be reported.

6.2.11 Duration of hospital admission

The mean duration of hospital admission will only be considered for those with a hospital admission and will be compared between each treatment arm with the usual care arm using a multiple linear regression model. The model will include outcome as the response variable, randomised group, age and presence of comorbidity as covariates. The mean (SD) duration will be presented in each group and the adjusted difference in means and 95% CI for each pairwise treatment arm comparison with the usual care group.

6.2.12 Well-being

The distribution of the WHO well-being index will be considered. Assuming there are not a large number of deaths or hospitalisations, a linear mixed effect model will be fitted to the data. Baseline well-being score will be fitted as a covariate in the model. Fixed effects will include randomised group, age, presence of comorbidity, time and a time x randomised group interaction. The mean (SD) well-being score at 14 and 28 days will be reported for each group and the adjusted difference in means (95% CI) for each pairwise treatment comparison with the usual care group will be presented.

6.2.13 WHO Ordinal Scale of Clinical Progression

If the data are available to calculate this scale then the following analysis will be carried out. The number and percentage of participants on each level of the scale will be presented by treatment group at days 7, 14 and 28. The outcome will be analysed using an ordinal logistic regression model, including the following covariates: randomised group; age; presence of comorbidity.

6.2.14 New infections in the Household

The count and percentage of participants with a new infection in the household will be presented for each treatment group. This outcome will be analysed using a logistic regression model. Randomised group, age and presence of comorbidity will be included covariates. The adjusted odds ratios will be reported for each pairwise comparison along with their associated 95% confidence interval and P-value. In addition, the adjusted absolute risk differences will be reported.

6.3 Handling missing data

Subjects with missing data for a given endpoint will not contribute data to the respective primary analysis. Sensitivity analyses will be conducted comparing data of persons with and without complete 28-days follow-up. Bayesian multiple imputation strategies of the primary analysis will be considered if there are characteristics or outcomes found to be associated with the primary endpoint.

6.4 Multiple comparisons and multiplicity

There will be no adjustment for multiplicity in the analysis of secondary outcomes.

6.5 Model assumptions

For the analysis of the secondary outcomes, model diagnostics will be checked.

7 SENSITIVITY ANALYSIS

Upon conclusion of the study or intervention (and at interims as needed), a stand-alone sensitivity analysis of each co-primary analysis will be conducted for each completed intervention, in which each intervention is compared Usual Care using only concurrent randomizations who meet the eligibility criteria of the intervention. These analyses will take the same form as the primary analysis models but may require modified priors/parameters for the temporal adjustment. For some interventions (e.g. hydroxychloroquine), temporal adjustment may not be necessary for this sensitivity analysis.

8 SUBGROUP ANALYSES

Model-based estimated treatment group differences in median time to recovery will be provided for each of the covariate subgroups (50-65 years old with comorbidities, ≥65 years old with comorbidities, and ≥65 years old without comorbidities), with 95% Bayesian credible intervals from the first co-primary analysis model. Similarly, model-based estimated differences in hospitalization rates will be provided for each of the covariate subgroups with 95% Bayesian credible intervals from the second co-primary analysis model.

Five further subgroup analyses of the time to recovery and death or hospitalisation outcomes will be carried out. Time to recovery will be analysed using the model specified for the analysis of time to alleviation of symptoms (section 6.2.2.1) and death or hospitalisation will be carried out using the same model used for other secondary binary outcomes (logistic regression model with randomised group, age and presence of comorbidity included as covariates). In addition the models will include an interaction between treatment group and the subgroup of interest. The P-values for the interaction effects will be presented and forest plots presented to show the effects in each subgroup and overall.

- Age group (<65/≥65 years)
- Presence of comorbidity at baseline (yes/no)
- Swab result (positive/negative/no result). This will be defined as described in section 3.3.16)
- Duration of symptoms prior to randomisation (this will be assessed as both a continuous outcome and using a cut off of ≤7 days vs. >7 days)
- Severity of symptoms score at baseline (using cut off at ≤ 5 and > 5)

9 SAFETY ANALYSIS

9.1 ADVERSE EVENTS

Number and severity of serious adverse events (SAE) will be summarised across treatment arms using numbers and proportions.

10 VALIDATION

The analysis of the primary outcome will be validated by a second statistician from the SAC. The final analysis of the secondary and safety outcomes will be validated by a Senior Trial Statistician or suitably qualified delegate from Oxford PC-CTU.

11 CHANGES TO THE PROTOCOL OR PREVIOUS VERSIONS OF SAP

The outcome 'WHO ordinal scale of clinical progression' is not in the current protocol (V6.0).

The current protocol (V6.0) has an outcome of 'consumption of antibiotics'. Information regarding antibiotics has only been collected in the notes review and therefore relates to prescriptions of antibiotics rather than patient reported consumption.

12 APPENDICES

12.1 APPENDIX I. SCHEDULE OF PROCEDURES

Procedures	Visits					

	Visit timing<br><br>Day 0	Day 0	Day 0	Day 0	Daily Day 1-28 incl	Day 29-12mths
	Screening completed by participant online/phone	Eligibility completed by participant online/phone	Baseline completed by participant online/phone	Eligibility completed by Clinician online/phone	Symptom Diaries completed by participant online/phone	Retrospective data collection by study team
Informed consent	X	X	X	X	X	
Demographics	X	X	X			X
Medical history	X	X	X	X		X
Concomitant medications		X				X
Eligibility assessment	X	X				
Randomisation				X		
Dispensing of trial drugs				X	X	
Daily Questionnaire					X	
Compliance					X	
Adverse event assessments					X	X

12.2 Appendix II. Flow diagram of trial participants

CONSORT
TRANSPARENT REPORTING of TRIALS

CONSORT 2010 Flow Diagram

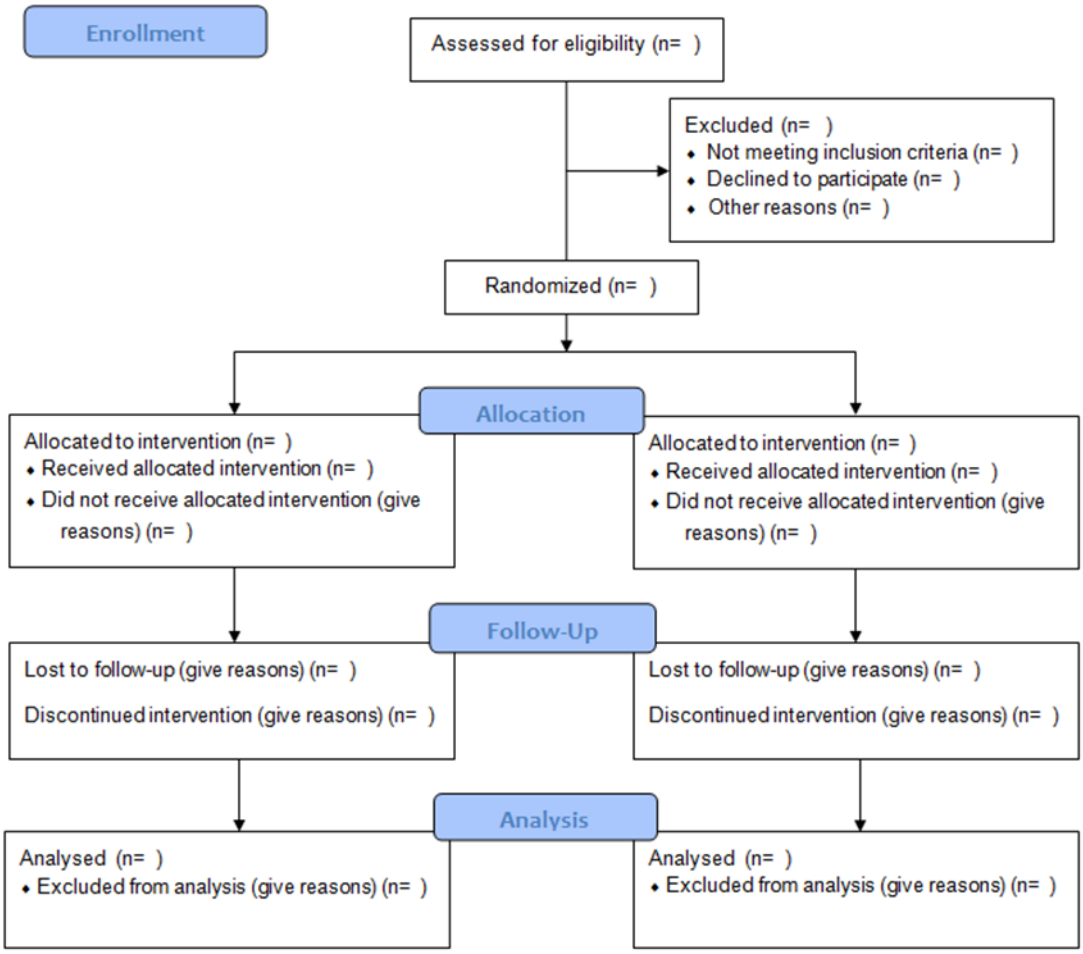

12.3 APPENDIX III: Plans for analysis of Hydroxychloroquine

Population of Analysis

The primary analysis population for the co-primary outcomes is defined as all randomised participants for whom data were available with participants analysed according to the groups they were randomly allocated to, at the time of the first trial interim analysis regardless of deviation from protocol and irrespective of their COVID-19 status. Although, participants randomised to other treatment arms were included in this interim analysis, only results of the hydroxychloroquine intervention will be reported.

Sensitivity and secondary analyses will be based on all participants randomised up to the point when hydroxychloroquine stopped recruitment.

Descriptive statistics

Baseline characteristics of participants will be summarised both overall and by randomised group, for all participants up to the point when randomisation to hydroxychloroquine was stopped. Data will be summarised as described in the M-SAP.

Primary Outcome Analysis

Analysis of the co-primary outcomes, i.e. time-to-recovery from randomisation and hospitalisation or death at 28 days from randomisation, are detailed in the Adaptive Design Report. Data from participants allocated to the hydroxychloroquine arm, and all control participants' data available at the time of the first interim analysis will be used, so there will be more control participants in the primary analysis than participants allocated to the hydroxychloroquine arm contributing to the primary analyses. However, due to the general lack of availability of swab tests during the Hydroxychloroquine testing phase, secondary analyses on the subset of participants with confirmed COVID-19 will not be performed.

Secondary Outcome Analysis

Secondary outcomes will be analysed as described in the M-SAP. All secondary outcomes will be analysed as detailed in the M-SAP.

Analysis of Safety Data

All serious adverse events will be reported as described in the M-SAP.

12.4 APPENDIX IV: PLANS FOR ANALYSIS OF AZITHROMYCIN

POPULATION OF ANALYSIS

The primary analysis population for the co-primary outcomes is defined as all randomised participants for whom data were available with participants analysed according to the groups they were randomly allocated to, at the time of the interim analysis regardless of deviation from protocol and irrespective of their COVID-19 status. Although, participants randomised to other treatment arms were included in this interim analysis, only results of the azithromycin intervention will be reported.

Sensitivity and secondary analyses will be based on all participants randomised between the first and last dates a patient was randomised to receive azithromycin. Only concurrent controls eligible for the azithromycin arm will be included.

DESCRIPTIVE STATISTICS

Baseline characteristics of participants will be summarised both overall and by randomised group, for all participants included in the population described above for the secondary analyses. Data will be summarised as described in the M-SAP.

PRIMARY OUTCOME ANALYSIS

Analysis of the co-primary outcomes, i.e. time-to-recovery from randomisation and hospitalisation or death at 28 days from randomisation, are detailed in the Adaptive Design Report. All relevant data available at the time of the interim will be used in the primary analysis.

SECONDARY OUTCOME ANALYSIS

Secondary outcomes will be analysed as described in the M-SAP. All secondary outcomes will be analysed as detailed in the M-SAP.

ANALYSIS OF SAFETY DATA

All serious adverse events will be reported as described in the M-SAP.

12.5 APPENDIX V: PLANS FOR ANALYSIS OF DOXYCYCLINE

POPULATION OF ANALYSIS

The primary analysis population for the co-primary outcomes is defined as all randomised participants for whom data were available with participants analysed according to the groups they were randomly allocated to, at the time of the interim analysis regardless of deviation from protocol and irrespective of their COVID-19 status. Although, participants randomised to other treatment arms were included in this interim analysis, only results of the doxycycline intervention will be reported.

Sensitivity and secondary analyses will be based on all participants randomised between the first and last dates a patient was randomised to receive doxycycline. Only concurrent controls eligible for the doxycycline arm will be included.

DESCRIPTIVE STATISTICS

Baseline characteristics of participants will be summarised both overall and by randomised group, for all participants included in the population described above for the secondary analyses. Data will be summarised as described in the M-SAP.

PRIMARY OUTCOME ANALYSIS

Analysis of the co-primary outcomes, i.e. time-to-recovery from randomisation and hospitalisation or death at 28 days from randomisation, are detailed in the Adaptive Design Report. All relevant data available at the time of the interim will be used in the primary analysis.

SECONDARY OUTCOME ANALYSIS

Secondary outcomes will be analysed as described in the M-SAP. All secondary outcomes will be analysed as detailed in the M-SAP.

ANALYSIS OF SAFETY DATA

All serious adverse events will be reported as described in the M-SAP.

12.6 APPENDIX VI: PLANS FOR ANALYSIS OF INHALED CORTICOSTEROID BUDESONIDE

POPULATION OF ANALYSIS

The primary analysis population for the co-primary outcomes is defined as all randomised participants for whom data were available with participants analysed according to the groups they were randomly allocated to, at the time of the interim analysis regardless of deviation from protocol and irrespective of their COVID-19 status. Although, participants randomised to other treatment arms were included in this interim analysis, only results of the inhaled corticosteroid intervention will be reported.

Sensitivity and secondary analyses will be based on all participants randomised between the first and last dates a patient was randomised to receive inhaled corticosteroid. Only concurrent controls eligible for the inhaled corticosteroid arm will be included.

DESCRIPTIVE STATISTICS

Baseline characteristics of participants will be summarised both overall and by randomised group, for all participants included in the population described above for the secondary analyses. Data will be summarised as described in the M-SAP.

PRIMARY OUTCOME ANALYSIS

Analysis of the co-primary outcomes, i.e. time-to-recovery from randomisation and hospitalisation or death at 28 days from randomisation, are detailed in the Adaptive Design Report. All relevant data available at the time of the interim will be used in the primary analysis.

SECONDARY OUTCOME ANALYSIS

Secondary outcomes will be analysed as described in the M-SAP. All secondary outcomes will be analysed as detailed in the M-SAP.

ANALYSIS OF SAFETY DATA

All serious adverse events will be reported as described in the M-SAP.

ADDENDUM TO PRINCIPLE M-SAP VERSION 1.1 12th Jan 2021

The purpose of this Addendum to version 1.0 of the PRINCIPLE M-SAP is to clarify the analysis population, identify patients to be included in the Hydroxychloroquine, Azithromycin and Doxycycline treatment comparisons and specify the datalock dates for each analysis.

DEFINITION OF ANALYSIS POPULATION

The analysis population will include all participants as defined by the protocol eligibility criteria.

As per ICH E9 guidance the following participants will be excluded from the analysis population;

(a) Participants randomised but subsequently found to be not eligible for randomization
(b) Participants previously randomised to an arm in the PRINCIPLE trial (subsequent randomisations will be excluded)

In addition, the following participants will be excluded;

(c) Participants who withdraw consent for data linkage and notes review and for whom no outcome data has been collected.

Tables of baseline characteristics will include only participants in the analysis population (i.e. excluded participants post randomisation will not be included). The number of participants excluded will be reported.

The PC-CTU unblinded statistician will ensure that the data transfer to the SAC for each treatment comparison only includes participants that meet the definition of the analysis population as specified above.

ANALYSIS POPULATIONS

Primary Analysis Population

The primary analysis population is based on all randomised participants for whom data are available, with participants analysed according to the groups they were randomly allocated to, regardless of deviation from protocol and irrespective of their COVID-19 status. In the setting of an adaptive platform trial with sequential overlap of treatment arms, the primary analysis for each intervention may be based on a distinct population. For each intervention that stops randomisation (due to futility) or becomes standard of care (due to superiority) based on interim analysis criteria, the date of the implementation of change in randomisation per interim decision will determine the intervention primary analysis population. For example, if randomisation is stopped for an intervention due to interim futility criteria, the final primary analysis includes all trial participants enrolled up to the date when randomisation was stopped to that intervention. Generally speaking, the primary analysis for a given intervention will be based on complete 28-

day follow-up of these participants, but the duration of follow-up may be impacted by public disclosure of interim results as determined by the Trial Management Group.

Concurrent Randomization Analysis Population

Sensitivity and secondary outcome analyses for a given intervention will be based on participants randomised during the same time frame when the intervention was actively being randomised, i.e. a concurrent randomization analysis population.

Infected Analysis Population for Primary Analysis

A secondary analysis population, "Infected Analysis Population for Primary Analysis", is defined as the Primary Analysis Population but will only include participants who are COVID-19 positive.

Infected Analysis Population for Concurrent Randomization Analysis

An additional secondary analysis population, "Infected Analysis Population for Concurrent Randomization Analysis", is defined as the Concurrent Randomization Analysis Population but will only include participants who are COVID-19 positive.

Safety Analysis Population

Safety analysis will be conducted on the as treated population (i.e the treatment that participants have received).

Appendix 4

Psychiatric Research Unit
WHO Collaborating Centre in Mental Health

WHO (Five) Well-Being Index (1998 version)

Please indicate for each of the five statements which is closest to how you have been feeling over the last two weeks. Notice that higher numbers mean better well-being.

Example: If you have felt cheerful and in good spirits more than half of the time during the last two weeks, put a tick in the box with the number 3 in the upper right corner.

Over the last two weeks:	All the time	Most of the time	More than half of the time	Less than half of the time	Some of the time	At no time
**1. I have felt cheerful and in good spirits**	5	4	3	2	1	0
**2. I have felt calm and relaxed**	5	4	3	2	1	0
**3. I have felt active and vigorous**	5	4	3	2	1	0
**4. I woke up feeling fresh and rested**	5	4	3	2	1	0
**5. My daily life has been filled with things that interest me**	5	4	3	2	1	0

Scoring:

The raw score is calculated by totaling the figures of the five answers. The raw score ranges from 0 to 25, 0 represent- ing worst possible and 25 representing best possible quality of life.

To obtain a percentage score ranging from 0 to 100, the raw score is multiplied by 4. A percentage score of 0 represents worst possible, whereas a score of 100 represents best possible quality of life.

Interpretation:

It is recommended to administer the Major Depression (ICD-10) Inventory if the raw score is below 13 or if the patient has answered 0 to 1 to any of the five items. A score below 13 indicates poor wellbeing and is an indication for testing for depression under ICD-10.

Monitoring change:

In order to monitor possible changes in wellbeing, the percentage score is used. A 10% difference indicates a significant change (ref. John Ware, 1995).

© Psychiatric Research Unit, WHO Collaborating Center for Mental Health, Frederiksborg General Hospital, DK-3400 Hillerød

Appendix 5

PRINCIPLE Trial Collaborative

Writing committee

Christopher C Butler C[1†], Jienchi Dorward [1,2], Ly-Mee Yu[1], Oghenekome Gbinigie[1], Gail Hayward[1], Benjamin R Saville[3,4], Oliver Van Hecke[1], Nick Berry[3], Michelle Detry[3], Christina Saunders[3], Mark Fitzgerald[3], Victoria Harris[1], Mahendra G Patel[1,5], Simon de Lusignan[1], Emma Ogburn[1], Philip H Evans[6,7], Nicholas PB Thomas[7,8], FD Richard Hobbs[1†], on behalf of the PRINCIPLE Collaborative Group

Writing Committee affiliations

1. Nuffield Department of Primary Care Health Sciences, University of Oxford, Oxford, UK (Prof CC Butler FMedSci, J Dorward MBChB, LM Yu DPhil, G Hayward DPhil, O Gbinigie MB BChir, O Van Hecke DPhil, E Ogburn PhD, Prof FDR Hobbs FMedSci)
2. Centre for the AIDS Programme of Research in South Africa (CAPRISA), University of KwaZulu–Natal, Durban, South Africa (J Dorward).
3. Berry Consultants, Texas, USA (BR Saville PhD, N Berry PhD, M Detry PhD, C Saunders PhD, M Fitzgerald PhD)
4. Department of Biostatistics, Vanderbilt University School of Medicine, Tennessee, USA (BR Saville)
5. School of Pharmacy and Medical Sciences, University of Bradford, Bradford, UK (Prof MG Patel, FRPharmS)
6. College of Medicine and Health, University of Exeter (Prof PH Evans FRCGP)
7. National Institute for Health Research, Clinical Research Network (PH Evans, NPB Thomas PhD)
8. Royal College of General Practitioners, London, UK (NPB Thomas)

Principle Trial Management Group

Julie Allen[1], Monique Andersson[2], Nick Berry[3], Emily Bongard[1], Aleksandra Borek[1], Christopher C Butler[1] (Chair), Simon de Lusignan[1], Jienchi Dorward[1,4], Philip H Evans[5,6], Filipa Ferreira[1], Oghenekome Gbinigie[1], Jenna Grabey[1], Gail Hayward[1], FD Richard Hobbs[1], Susan Hopkins[7], David Judge[1], Mona Koshkouei[1], Martin J Llewelyn[8], Emma Ogburn[1], Mahendra G. Patel[9], Dan Richards-Doran[1], Heather Rutter[1], Benjamin R. Saville[2,10], Hannah Swayze[1], Nicholas PB Thomas[5,11], Manasa Tripathy[1], Sarah Tonkin-Crine[1,12], Sharon Tonner[1], Ly-Mee Yu[1], Oliver Van Hecke[1]

Trial Management Group affiliations

1. Nuffield Department of Primary Care Health Sciences, University of Oxford, Oxford, UK
2. Department of Microbiology, Oxford University Hospitals NHS Trust, Oxford, UK
3. Berry Consultants, Texas, USA,
4. Centre for the AIDS Programme of Research in South Africa (CAPRISA), University of KwaZulu–Natal, Durban, South Africa.
5. National Institute for Health Research, Clinical Research Network

6. College of Medicine and Health, University of Exeter
7. Public Health England, London, UK
8. Brighton and Sussex Medical School, University of Sussex, Brighton, UK
9. School of Pharmacy and Medical Sciences, University of Bradford, Bradford, UK
10. Department of Biostatistics, Vanderbilt University School of Medicine, Tennessee, USA
11. Royal College of General Practitioners, London, UK
12. National Institute for Health Protection Research Unit in Healthcare Associated Infections and Antimicrobial Resistance, Oxford, UK

Statistical Analysis Committee
Nick Berry, Michelle Detry (Chair), Christina Saunders, Mark Fitzgerald

Trial Steering Committee
Carol Green, Phil Hannaford, Paul Little (Chair), Tim Mustill, Matthew Sydes

Data Monitoring and Safety Committee
Deborah Ashby (Chair), Nick Francis, Simon Gates, Gordon Taylor, Patrick White

Principle Trial Coordinating Office

Joint Chief Investigators: CC Butler, FDR Hobbs

Trial management: E Ogburn (coordinator), H Swayze, E Bongard, J Allen, S Tonner, R Edeson, J Brooks, R Edwards, N Maeder, S Barrett, S Brann, A Maloney, K Dempster, J de Henau, J Robinson, N Begum

Clinical Team: H Rutter (Coordinator), K Madronal, B Mundy, B Ianson, I Noel, B Thompson, O Gbinigie, J Dorward, G Hayward, O van Hecke, N Jones, H van der Westhuizen, K Kotze

Data and Programming Team: D Judge, J Grabey, L Castello, D Watt, R Zhao

Statistics: B Saville, L-M Yu, N Berry, M Detry, V Harris, C Saunders, M Fitzgerald, J Mollison, U Galal

Royal College of General Practitioner's Research Support Centre: S de Lusignan (coordinator) M Tripathy, F Ferreira

National Institute for Health Research Clinical Research Network Coordinating Centre:
PH Evans, NPB Thomas, H Collins, Katherine Priddis, Lydia Owen, Kate Hannaby, Ben Drew

Table S1: Baseline characteristics of all eligible, randomised participants by study arm (Concurrent Randomised Analysis population)

	**Azithromycin (N=500)**	**Usual Care (N=629)**	**OVERALL (N=1129)**
Age (years), mean(SD) [min,max]	60.9 (7.8) [50.0 to 90.0]	60.6 (7.7) [50.0 to 85.0]	60.7 (7.8) [50.0 to 90.0]
Age category, n(%)			
Greater than or equal to 65 years	326 (65.2%)	417 (66.3%)	743 (65.8%)
Less than 65 years	174 (34.8%)	212 (33.7%)	386 (34.2%)
Sex, n(%)			
Female	284 (56.8%)	357 (56.8%)	641 (56.8%)
Male	215 (43.0%)	271 (43.1%)	486 (43.0%)
Missing, n(%)	1 (0.2%)	1 (0.2%)	2 (0.2%)
Ethnicity*, n(%)			
White	418 (83.6%)	539 (85.7%)	957 (84.8%)
Mixed background	9 (1.8%)	13 (2.1%)	22 (1.9%)
South Asian	20 (4.0%)	24 (3.8%)	44 (3.9%)
Black	0 (0.0%)	3 (0.5%)	3 (0.3%)
Other	9 (1.8%)	5 (0.8%)	14 (1.2%)
Missing, n(%)	44 (8.8%)	45 (7.2%)	89 (7.9%)
Duration of illness prior to randomization (days), median(IQR) [range]	6 (4 to 10) [0 to 28]	7 (5 to 10) [0 to 26]	7 (4 to 10) [0.0 to 28.0]
Missing, n(%)	24 (2.1%)	29 (2.6%)	53 (4.7%)
Smoking_status, n(%)			
Current smoker	57 (11.4%)	72 (11.4%)	129 (11.4%)
Former smoker	175 (35.0%)	245 (39.0%)	420 (37.2%)
Never smoker	255 (51.0%)	293 (46.6%)	548 (48.5%)
Missing, n(%)	13 (2.6%)	19 (3.0%)	32 (2.8%)
Swab result within 28 days of randomisation, n(%)			
Negative	261 (52.2%)	323 (51.4%)	584 (51.7%)
Positive	186 (37.2%)	225 (35.8%)	411 (36.4%)
No result	1 (0.2%)	5 (0.8%)	6 (0.5%)
Missing, n(%)	52 (10.4%)	76 (12.1%)	128 (11.3%)
Comorbidities, n(%)			
No	63 (12.6%)	79 (12.6%)	142 (12.6%)
Yes	437 (87.4%)	550 (87.4%)	987 (87.4%)
Asthma, COPD or lung disease, n(%)	181 (36.2%)	224 (35.6%)	405 (35.9%)
Diabetes, n(%)	85 (17.0%)	119 (18.9%)	204 (18.1%)
Heart problems†, n(%)	82 (16.4%)	82 (13.0%)	164 (14.5%)
High blood pressure for which you are taking medications, n(%)	198 (39.6%)	250 (39.7%)	448 (39.7%)
Liver disease, n(%)	13 (2.6%)	16 (2.5%)	29 (2.6%)
Comorbidities			
Stroke or other neurological problem, n(%)	27 (5.4%)	32 (5.1%)	59 (5.2%)
Taking ACE inhibitor‡, n(%)	98 (19.6%)	119 (18.9%)	217 (19.2%)
Missing, n(%)	2 (0.4%)	3 (0.5%)	5 (0.4%)
Fever, n(%)			
No problem	212 (42.4%)	279 (44.4%)	491 (43.5%)
Minor problem	158 (31.6%)	215 (34.2%)	373 (33.0%)
Moderate problem	114 (22.8%)	116 (18.4%)	230 (20.4%)
Major problem	16 (3.2%)	19 (3.0%)	35 (3.1%)
Cough, n(%)			
No problem	85 (17.0%)	116 (18.4%)	201 (17.8%)
Minor problem	210 (42.0%)	255 (40.5%)	465 (41.2%)
Moderate problem	168 (33.6%)	225 (35.8%)	393 (34.8%)
Baseline symptoms			
Major problem	37 (7.4%)	33 (5.2%)	70 (6.2%)
Shortness of breath, n(%)			
No problem	196 (39.2%)	224 (35.6%)	420 (37.2%)
Minor problem	203 (40.6%)	265 (42.1%)	468 (41.5%)
Moderate problem	85 (17.0%)	130 (20.7%)	215 (19.0%)
Major problem	16 (3.2%)	10 (1.6%)	26 (2.3%)
Muscle ache, n(%)			
No problem	155 (31.0%)	191 (30.4%)	346 (30.6%)
Minor problem	184 (36.8%)	251 (39.9%)	435 (38.5%)
Moderate problem	127 (25.4%)	141 (22.4%)	268 (23.7%)
Major problem	34 (6.8%)	46 (7.3%)	80 (7.1%)
Nausea, n(%)			
No problem	375 (75.0%)	470 (74.7%)	845 (74.8%)
Minor problem	94 (18.8%)	133 (21.1%)	227 (20.1%)
Moderate problem	25 (5.0%)	22 (3.5%)	47 (4.2%)
Major problem	6 (1.2%)	4 (0.6%)	10 (0.9%)
Feeling generally unwell (malaise), n(%)			
No problem	28 (5.6%)	40 (6.4%)	68 (6.0%)

	Azithromycin (N=500)	Usual Care (N=629)	OVERALL (N=1129)
Minor problem	203 (40.6%)	281 (44.7%)	484 (42.9%)
Moderate problem	195 (39.0%)	252 (40.1%)	447 (39.6%)
Major problem	49 (9.8%)	34 (5.4%)	83 (7.4%)
Missing, n(%)	25 (5.0%)	22 (3.5%)	47 (4.2%)
Diarrhea, n(%)			
No problem	341 (68.2%)	454 (72.2%)	795 (70.4%)
Minor problem	87 (17.4%)	106 (16.9%)	193 (17.1%)
Moderate problem	34 (6.8%)	37 (5.9%)	71 (6.3%)
Major problem	13 (2.6%)	10 (1.6%)	23 (2.0%)
Missing, n(%)	25 (5.0%)	22 (3.5%)	47 (4.2%)
Have you taken antibiotics since your illness started, n(%)	15 (3.0%)	21 (3.3%)	36 (3.2%)
Missing, n(%)	1 (0.2%)	1 (0.2%)	2 (0.2%)
GP, n(%)	141 (28.2%)	152 (24.2%)	293 (26.0%)
Other primary care services, n(%)	25 (5.0%)	32 (5.1%)	57 (5.0%)
NHS 111, n(%)	81 (16.2%)	99 (15.7%)	180 (15.9%)
A&E, n(%)	6 (1.2%)	11 (1.7%)	17 (1.5%)
Other healthcare services, n(%)	43 (8.6%)	55 (8.7%)	98 (8.7%)
Use of healthcare services			
WHO5 well-being score§, mean(SD)	50.3 (25.3)	50.5 (24.4)	50.4 (24.8)

* Data on ethnicity were collected retrospectively via notes review.
† E.g. angina, heart attack, heart failure, atrial fibrillation, valve problems
‡ Such as Ramipril, Lisinopril, Perindopril, Captopril or Enalapril
§Well-being is measured using the WHO well-being index which includes 5 items relating to well-being measured on a five point scale. A total score is computed by summing the scores to the five individual questions to give a raw score ranging from 0 to 25 which is then multiplied by 4 to give the final score from 0 representing the worst imaginable well-being to 100 representing the best imaginable well-being.

Table S2: Sensitivity analysis of primary and secondary outcomes

	Azithromycin	Usual Care	Estimated treatment effect (95% BCI)	Pr(Meaningful effect)	Pr(Superiority)
**Primary outcomes (Concurrent Randomised Analysis population)**					
First reported recovery, n/N (%)	402/500 (80·4%)	631/823 (76·7%)			
Time to first reported recovery, median (IQR)	7 (3 – 17)	8 (2 – 23)	1·08 (0·95 – 1·23)*	0·23*	0·89*
Hospitalisation/death at 28 days, n/N (%)	16/500 (3·2%)	22/629 (3·5%)	·4% (-2·4%, 3·6%)†	0·22†	0·66†
**Secondary outcomes (Concurrent Randomised Analysis population for SARS-CoV-2 positive population)** ‡	**Azithromycin**	**Usual Care**	**Estimated treatment effect (95% CI)**	**P-value**	
Sustained recovery, n/N (%)	98/186 (52·7%)	131/225 (58·2%)			
Time to sustained recovery, median (IQR)	28 (10 – ··)	23 (12 – ··)	0·89 (0·68 – 1·16)§	0·40	
Alleviation of all symptom, n/N (%)	153/157 (97·5%)	163/178 (91·6%)			
Time to alleviation of all symptom, median (IQR)	3 (2 – 7)	4 (2 – 9)	1·19 (0·95 – 1·49)§	0·12	
Sustained alleviation of all symptom, n/N (%)	126/154 (81·8%)	142/174 (81·6%)			
Time to sustained alleviation of all symptom, median (IQR)	8 (3 – 24)	10 (4 – 21)	1.01 (0·79 – 1·29)§	0·93	
Initial reduction of severity of symptom, n/N (%)	163/186 (87·6%)	196/225 (87·1%)			
Time to initial reduction of severity of symptom, median (IQR)	5 (2 – 11)	6 (3 – 14)	1·05 (0·85 – 1·30)§	0·63	
Rating of how well participant feels (1 worst, 10 best), mean (SD) [n]					
Day 7	7·1 (1·9) [179]	6·8 (1·8) [217]	0·25 (-0·11 to 0·62)ll	0·18	
Day 14	7·9 (1·7) [185]	7·7 (1·7) [220]	0·13 (-0·27 to 0·53)ll	0·52	
Day 21	8·2 (1·7) [160]	8·0 (1·6) [195]	00·09 (-0·37 to 0·55)ll	0·71	
Day 28	8·2 (1·7) [187]	8·3 (1·5) [219]	--0·07 (-0·59 to 0·45)ll	0·80	
Well-being (WHO5 Questionnaire), mean (SD)[n]					
Day 14	45·0 (25·1) [182]	42·2 (24·3) [217]	1·18 (-3·21 to 5·56)ll	0·60	
Day 28	56·1 (24·6) [180]	56·0 (24·5) [213]	-0·15 (-4·55 to 4·25)ll	0·95	
Self-reported contact with ≥1 healthcare service	105/186 (56·5%)	128/225 (56·9%)	1·03 (0·87 to 1·22)¶	0·76	
GP reported contact with ≥1 healthcare service	63/107 (58·9%)	72/139 (51·8%)	1·14 (0·91 to 1·43)¶	0·25	
Prescription of antibiotics	7/101 (6·9%)	7/126 (5·6%)	1·25 (0·45 to 3·44)**	0·78	
Hospital assessment without admission	3/186 (1·6%)	4/225 (1·8%)	0·91 (0·21 to 4·00)**	>0·99	
Oxygen Administration	6/184 (3·3%)	9/222 (4·1%)	0·80 (0·29 to 2·22)**	0·79	
Mechanical ventilation	2/183 (1·1%)	4/222 (1·8%)	0·61 (0·11 to 3·27)**	0·69	
ICU admission	2/182 (1·1%)	4/222 (1·8%)	0·61 (0·11 to 3·29)**	0·69	

* Estimated hazard ratio derived from a Bayesian piecewise exponential model adjusted for age and comorbidity at baseline, with 95% Bayesian credible interval. Hazard ratio > 1 favors azithromycin. Pr(Meanginful effect) is the model-based estimated probability that the benefit in median time to recovery compared to Usual Care is at least 1.5 days. Pr(Superiority) is the probability of superiority and treatment superiority is declared if Pr(superiority) ≥ 0.99 versus usual care.

† Estimated absolute benefit in percentage of hospitalisation/death derived from a Bayesian logistic regression model adjusted for age and comorbidity at baseline, with 95% Bayesian credible interval. A positive value favors azithromycin. Pr(Meanginful effect) is the model-based estimated probability that the benefit in hospitalisation/death compared to Usual Care is at least 2% days. Pr(Superiority) is the probability of superiority and treatment superiority is declared if Pr(superiority) ≥ 0.975 versus usual care

‡ All secondary outcome analyses were conducted on the concurrent randomization analysis population, but restricted to those in the azithromycin and usual care group only.

§ Estimated hazard ratio derived from a Cox proportional hazard model adjusted for age, comorbidity at baseline, duration of illness, and eligible for azithromycin at baseline, with 95% confidence interval.

ll Mixed effect model adjusting age, comorbidity, duration of illness, eligible for azithromycin at baseline, and time. Participant was fitted as a random effect. WHO well-being score was also adjusted for the score at baseline

¶ Relative risk adjusted for age, comorbidity at baseline, duration of illness, and eligible for azithromycin at baseline

** Unadjusted relative risk due to low event rate.

Figure S1: GP practices that have recruited at least one participant to PRINCIPLE (n=1406)

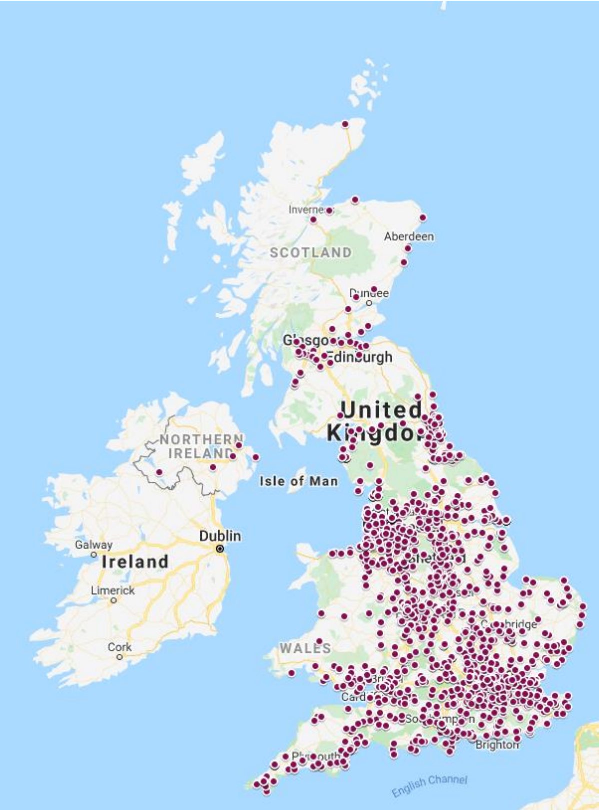

Figure S2: Summary and results of the time to first self-reported recovery for Concurrent Randomisation Analysis Population

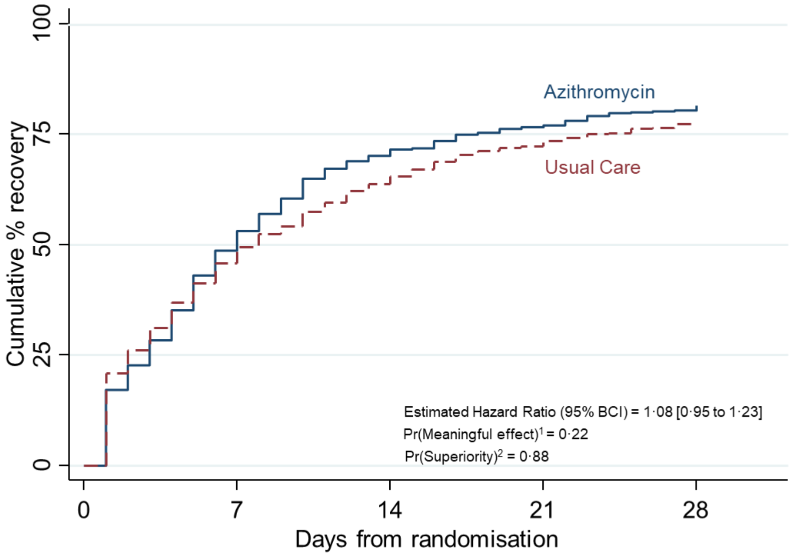

Number not yet recovered (recovered)

	0	7	14	21	28
Azithromycin	500 (0)	254 (265)	144 (355)	111 (381)	93 (402)
Usual Care	629 (0)	337 (310)	219 (407)	163 (455)	133 (477)

[1] Bayesian model-based estimated probability that the benefit in median time to recovery compared to Usual Care is at least 1.5 days

[2] Probability of superiority, treatment superiority is declared if Pr(superiority) ≥ 0.99 versus Usual Care

Figure S3 Estimated mean and 95% confidence interval of daily rating of feeling well over the 28 days follow-up by treatment arm

(a) Concurrent Randomisation Analysis population but restricted to those in the azithromycin and usual care group only

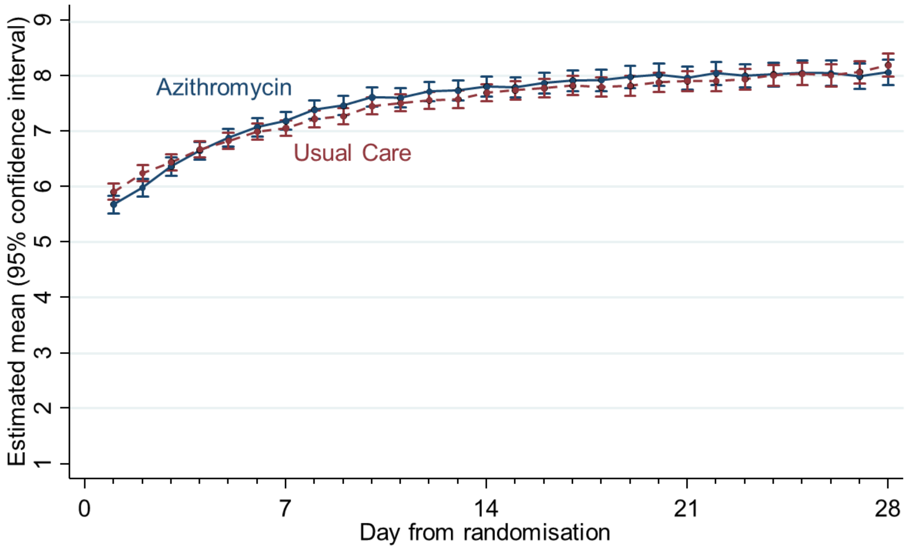

(b) Concurrent Randomisation Analysis population for SARS-CoV-2 positive population

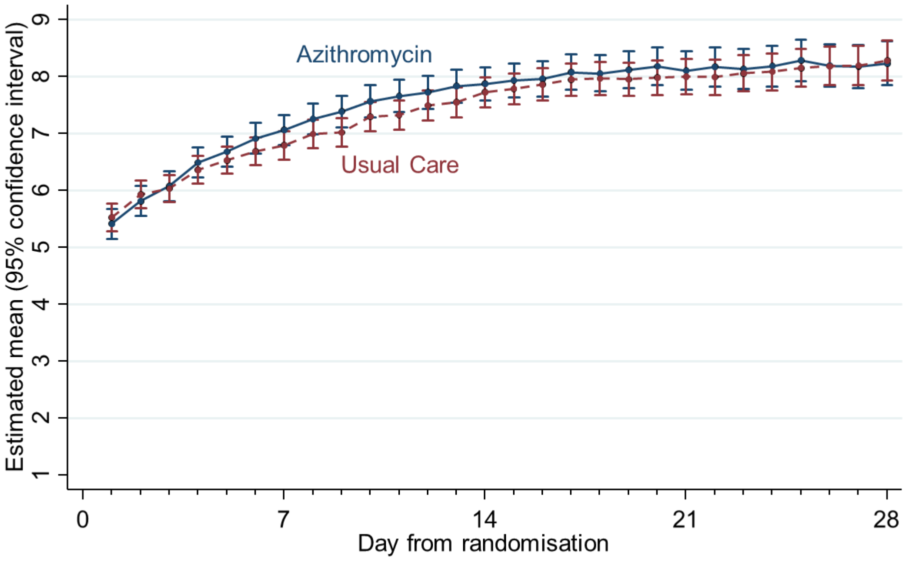

Figure S4: Effect of allocation to azithromycin on time to event outcomes Concurrent Randomisation Analysis population but restricted to those in the azithromycin and usual care group only

(a) Time to alleviations of all symptoms and alleviation of each symptom

Time to Alleviation of Symptom[1]	Azithromycin	Usual Care	Median time (days) Azithromycin	Median time (days) Usual Care	Adjusted HR (95% CI)	P-value
All symptoms	401/420	473/505	3	3	1·04 (0·91, 1·19)	0·57
Fever	195/198	217/224	2	2	1·11 (0·91, 1·36)	0·30
Cough	267/274	336/348	3	3	1·08 (0·91, 1·27)	0·38
Shortness of breath	180/193	242/259	4	5	1·08 (0·88, 1·31)	0·46
Muscle ache	244/252	288/302	3	3	1·12 (0·94, 1·33)	0·22
Nausea-vomiting	80/81	80/85	3	5	1·24 (0·90, 1·72)	0·19
Diarrhoea	139/144	129/142	4	5	1·20 (0·93, 1·53)	0·16
Generally unwell (malaise)	334/356	404/425	3	3	0·98 (0·84, 1·14)	0·77

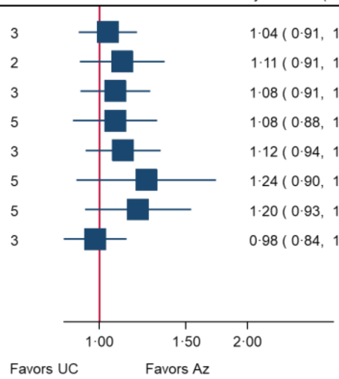

[1]Time to alleviation of symptoms is defined as the time from randomisation to all symptoms or each symptom being rated as mild or none.

(b) Time to sustained alleviation of all symptoms and alleviation of each symptom

Time to Sustained Alleviation[1]	Azithromycin	Usual Care	Median time (days) Azithromycin	Median time (days) Usual Care	Adjusted HR[2] (95% CI)	P-value
All symptoms	338/422	425/510	8	10	0·94 (0·81, 1·09)	040
Fever	191/198	213/224	3	4	1·13 (0·93, 1·38)	0·23
Cough	245/274	318/348	7	7	0·97 (0·82, 1·15)	0·70
Shortness of breath	155/193	216/259	12	11	0·95 (0·77, 1·17)	0·62
Muscle ache	210/252	265/302	7	8	0·94 (0·78, 1·12)	0·47
Nausea-vomiting	78/81	75/85	5	8	1·45 (1·04, 2·01)	0·028
Diarrhoea	137/144	124/142	6	7	1·32 (1·03, 1·71)	0·031
Generally unwell (malaise)	301/356	369/425	8	8	0·95 (0·81, 1·11)	0·52

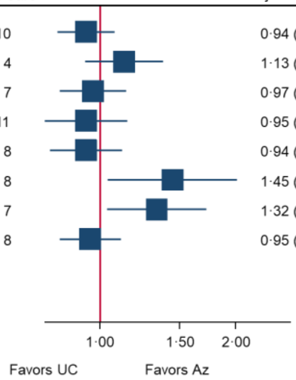

[1]Time to sustained alleviation of symptoms is defined as time to alleviation of symptoms with no subsequent symptom being rated as mild or none.

(c) Time to initial reduction of severity of all symptoms and alleviation of each symptom

Time to Initial Reduction of Severity of Symptom[1]	Azithromycin	Usual Care	Median time (days) Azithromycin	Median time (days) Usual Care	Adjusted HR (95% CI)	P-value
All symptoms	449/494	554/622	4	4	0·99 (0·87, 1·13)	0·91
Fever	285/288	343/350	1	1	1·04 (0·89, 1·22)	0·62
Cough	401/415	482/513	3	2	1·06 (0·92, 1·21)	0·42
Shortness of breath	278/304	363/405	3	4	1·01 (0·86, 1·18)	0·94
Muscle ache	329/345	419/438	2	2	0·95 (0·82, 1·11)	0·53
Nausea-vomiting	125/125	155/159	1	1	1·04 (0·82, 1·33)	0·74
Diarrhoea	132/159	149/175	2	2	1·01 (0·79, 1·28)	0·95
Generally unwell (malaise)	427/472	534/589	3	3	0·96 (0·85, 1·10)	0·56

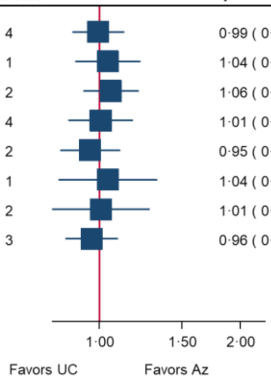

[1]Time to initial reduction of severity of symptoms is defined as time to reduction in severity of each individual symptoms to at least one grade lower.

Figure S5 Effect of allocation to azithromycin on time to event outcomes (Concurrent Randomisation Analysis population for SARS-CoV-2 positive population)

(a) Time to alleviations of all symptoms and alleviation of each symptom

Time to Alleviation of Symptom[1]	Azithromycin	Usual Care	Median time (days) Azithromycin	Median time (days) Usual Care	Adjusted HR (95% CI)	P-value
All symptoms	153/157	163/178	3	4	1·19 (0·95, 1·49)	0·12
Fever	78/79	82/85	2	3	0·97 (0·70, 1·33)	0·84
Cough	83/84	102/104	3	3	0·97 (0·72, 1·30)	0·83
Shortness of breath	58/64	84/91	5	6	0·85 (0·61, 1·20)	0·37
Muscle ache	97/99	117/124	3	3	1·13 (0·86, 1·49)	0·38
Nausea-vomiting	29/30	36/41	3	4	1·34 (0·79, 2·27)	0·28
Diarrhoea	50/51	40/43	4	5	1·40 (0·89, 2·18)	0·14
Generally unwell (malaise)	136/141	158/163	3	3	0·99 (0·78, 1·25)	0·92

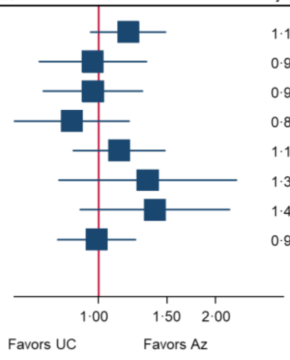

[1]Time to alleviation of symptoms is defined as the time from randomisation to all symptoms or each symptom being rated as mild or none.

(b) Time to sustained alleviation of all symptoms and alleviation of each symptom

Time to Sustained Alleviation	Azithromycin	Usual Care	Median time (days) Azithromycin	Median time (days) Usual Care	Adjusted HR (95% CI)	P-value
All symptoms	126/154	142/174	8	10	1·01 (0·79, 1·29)	0·93
Fever	78/79	81/85	3	3	1·05 (0·77, 1·45)	0·74
Cough	79/84	99/104	6	7	0·94 (0·70, 1·27)	0·68
Shortness of breath	51/64	77/91	11	10	0·79 (0·55, 1·13)	0·20
Muscle ache	87/99	110/124	6	5	0·98 (0·74, 1·31)	0·90
Nausea-vomiting	29/30	35/41	3	6	1·68 (0·98, 2·87)	0·060
Diarrhoea	50/51	39/43	6	7	1·39 (0·88, 2·19)	0·16
Generally unwell (malaise)	125/141	142/163	7	7	0·98 (0·77, 1·25)	0·86

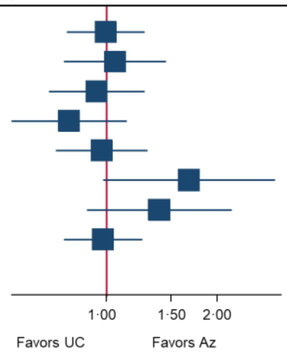

[1]Time to sustained alleviation of symptoms is defined as time to alleviation of symptoms with no subsequent symptom being rated as mild or none.

(c) Time to initial reduction of severity of all symptoms and alleviation of each symptom

Time to Initial Reduction of Severity of Symptom	Azithromycin	Usual Care	Median time (days) Azithromycin	Median time (days) Usual Care	Adjusted HR (95% CI)	P-value
All symptoms	163/186	196/225	5	6	1·05 (0·85, 1·30)	0·63
Fever	110/110	115/118	1	1	1·07 (0·82, 1·40)	0·61
Cough	145/152	165/179	3	4	1·09 (0·87, 1·37)	0·46
Shortness of breath	76/90	118/134	5	5	0·88 (0·66, 1·18)	0·41
Muscle ache	131/136	161/168	2	3	1·15 (0·91, 1·46)	0·25
Nausea-vomiting	48/48	58/61	1	2	1·08 (0·72, 1·62)	0·69
Diarrhoea	60/60	58/60	1	2	1·24 (0·85, 1·81)	0·27
Generally unwell (malaise)	175/181	197/211	4	4	1·10 (0·90, 1·36)	0·36

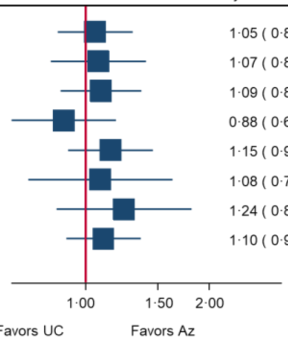

[1]Time to initial reduction of severity of symptoms is defined as time to reduction in severity of each individual symptoms to at least one grade lower.
